# Re-Exploring the Anthracycline Chemical Space for Better Anti-Cancer Compounds

**DOI:** 10.1021/acs.jmedchem.3c00853

**Published:** 2023-08-10

**Authors:** Merle A. van Gelder, Sabina Y. van der Zanden, Merijn B. L. Vriends, Roos A. Wagensveld, Gijsbert A. van der Marel, Jeroen D. C. Codée, Herman S. Overkleeft, Dennis P. A. Wander, Jacques J. C. Neefjes

**Affiliations:** † Department of Cell and Chemical Biology, ONCODE Institute, Leiden University Medical Center, Einthovenweg 20, 2333 ZC Leiden, The Netherlands; ‡ Leiden Institute of Chemistry, Leiden University, Einsteinweg 55, 2333 CC Leiden, The Netherlands

## Abstract

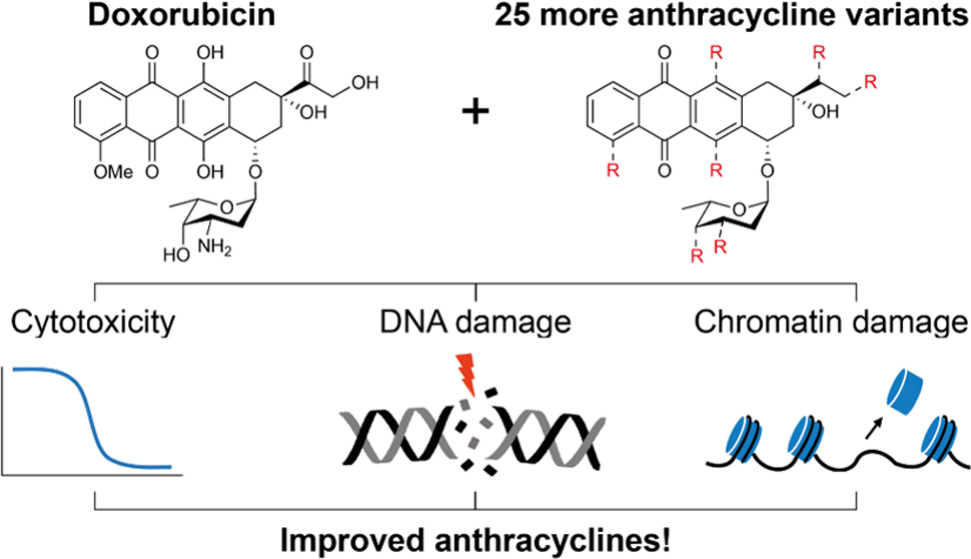

The anthracycline anti-cancer drugs are intensely used in the clinic to treat a wide variety of cancers. They generate DNA double strand breaks, but recently the induction of chromatin damage was introduced as another major determinant of anti-cancer activity. The combination of these two events results in their reported side effects. While our knowledge on the structure–activity relationship of anthracyclines has improved, many structural variations remain poorly explored. Therefore, we here report on the preparation of a diverse set of anthracyclines with variations within the sugar moiety, amine alkylation pattern, saccharide chain and aglycone. We assessed the cytotoxicity *in vitro* in relevant human cancer cell lines, and the capacity to induce DNA- and chromatin damage. This coherent set of data allowed us to deduce a few guidelines on anthracycline design, as well as discover novel, highly potent anthracyclines that may be better tolerated by patients.

## Introduction

Anthracyclines have been used extensively as chemotherapeutics in the treatment of various hematological cancers and solid tumors since their discovery in the 1960s.^1^ Because of their broad anti-cancer effectivity they are considered “essential medicines” by the WHO,^2^ and their remarkable potency has inspired the development of thousands of variants.^3^ Only few of these analogues have been approved for clinical use,^4^ of which only doxorubicin, daunorubicin, epirubicin and idarubicin have been adopted for worldwide use. While these anthracyclines are among the most effective anti-cancer drugs, their clinical application is hampered by treatment-limiting side effects and drug resistance.^5,6^ The side effects of anthracycline treatment are severe: cardiotoxicity, secondary tumor formation and infertility affect the quality of life and survival of patients, regardless of the cancer prognosis.^7−10^ Of these, cardiotoxicity is the main adverse effect, which emerges in a cumulative manner and is restricting treatment regimens as a consequence.^8^

**Figure 1:**
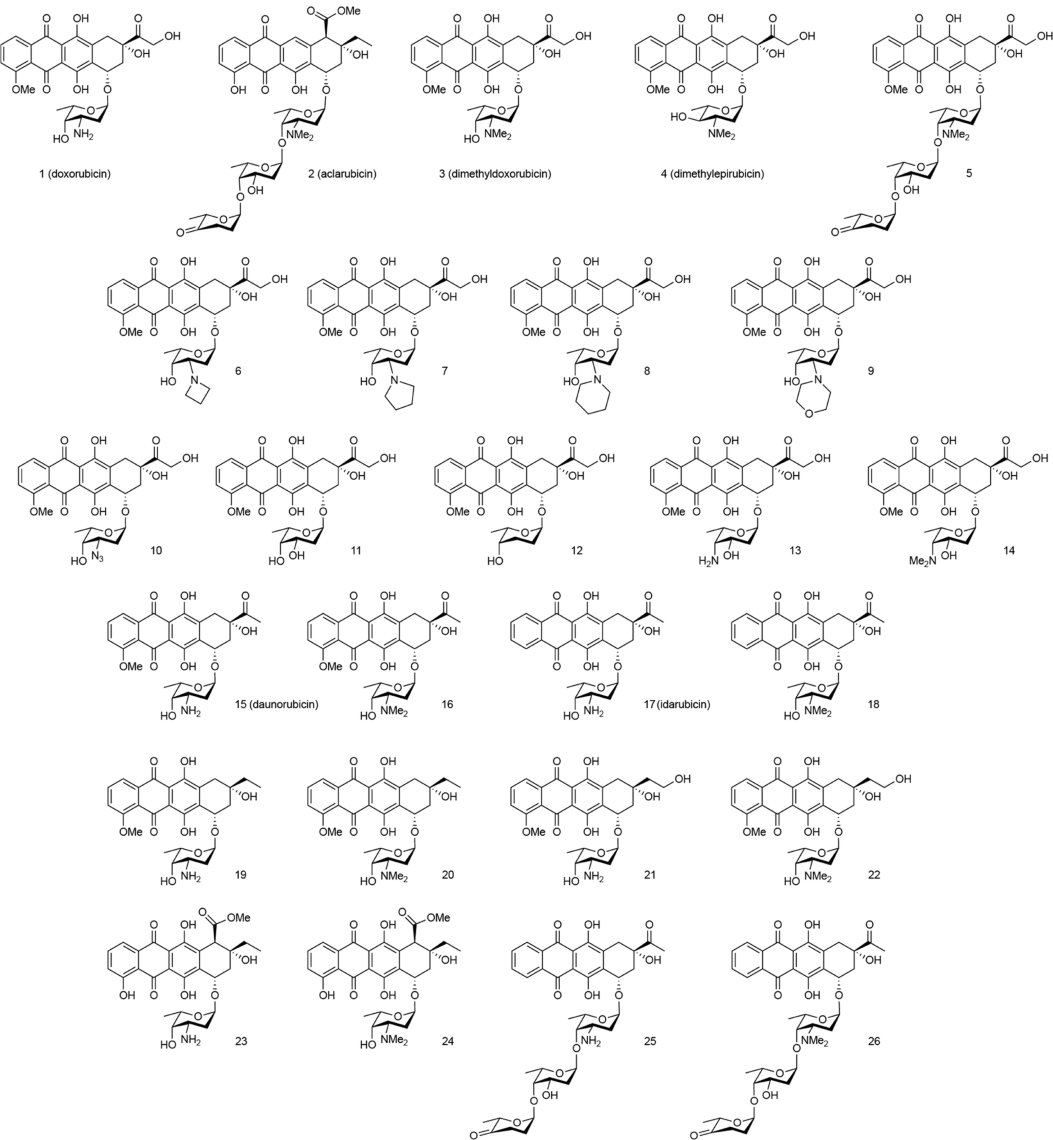
Chemical structures of compounds **1**–**26**, evaluated in this study. Clinically used anthracyclines doxorubicin (**1**), aclarubicin (**2**), daunorubicin (**15**), idarubicin (**17**); the most potent anthracyclines from our previous work (**3**–**5**); doxorubicin derivatives differing in the sugar moiety (**6**–**14**) and (*N,N*-dimethyl) derivatives differing in the aglycone part (**16**, **18**–**24**) and idarubicin-trisaccharides (**25** and **26**).

It has long been appreciated that anthracycline drugs are able to cause DNA double-strand breaks by inhibition or poisoning of topoisomerase II.^11^ For decades, this mode of action was thought to be the main reason for the remarkable effectiveness of these drugs. However, we revealed that DNA damage is not the only mode of action for most anthracycline variants. All clinically used anthracyclines induce chromatin damage upon DNA intercalation and subsequent eviction of histones.^12,13^ Furthermore, we recently showed that the combination of DNA damage and chromatin damage, as exerted by doxorubicin, results in the major side effects reported for this compound.^13^ In contrast, aclarubicin solely induces chromatin damage and is neither cardiotoxic nor induces therapy-related malignancies. Comparison of the structural similarities and differences of doxorubicin and aclarubicin inspired the design of *N,N*-dimethyldoxorubicin (**3**, Figure 1). This variant showed adequate anticancer effectivity *in vitro* and in various *in vivo* models, without accompanying (cardio)toxicity.^13^ These results suggest that separating DNA damage from chromatin damage activities may guide the development of novel variants that lack the major long-term side effects that are associated with the anthracycline variants currently in clinical use.

In a follow-up study with the aim to better understand the molecular mode of action of these anthracycline drugs, we synthesized a focused library of diastereomers of doxorubicin in the 1,2-amino alcohol arrangement of the 2,3-dideoxy-3-amino-l-fucoside. This yielded *N,N*-dimethylepirubicin (**4**, Figure 1), a compound slightly more potent than *N,N*-dimethyldoxorubicin.^14^ In addition, the evaluation of doxorubicin/aclarubicin hybrid structures, varying in the tetracyclic aglycone, the sugar moiety and the *N*-alkylation pattern generated the doxorubicin trisaccharide (**5**, Figure 1) that is nearly 20-fold more cytotoxic than doxorubicin.^15^ Building onto these studies, we here present a further systematic expansion of our anthracycline library through the synthesis and evaluation of 19 additional anthracyclines. These constitute variations in amine alkylation (**6**–**9**), replacement/removal of the basic amine (**10**–**12**) and regio-isomers in the sugar moiety (**13** and **14**). Additionally, exploration of the chemical space in the aglycone yielded (*N,N*-dimethyl-)amine bearing monosaccharides **15**–**24** and trisaccharides **25** and **26**. We determined the cytotoxic potency of these new variants in relevant cancer cell line models as well as their ability to induce both DNA- and chromatin damage, and compared these to the clinically used variants doxorubicin (**1**), aclarubicin (**2**), daunorubicin (**15**) and idarubicin (**17**) and our most effective variants from previous studies (**3–5**).^13−15^ Small modifications in the aglycone moiety lead to marked changes in the cytotoxicity of our compounds. Furthermore, our results underline our earlier findings that a tertiary amine on the first saccharide commonly improves the cytotoxicity of the compounds.

In summary, our endeavors to explore the chemical space of anthracycline variants resulted in a total of ten compounds that were more effective in K562 cells than doxorubicin (**1**), the clinically foremost used anthracycline. Of this list, compound **26**, composed of the idarubicin aglycone and the aclarubicin trisaccharide proved to be the most cytotoxic agent of the series with an IC_50_ towards K562 tumor cells in the low nanomolar range. This analogue does not induce DNA damage and is the fastest histone evictor we have identified to date. Consequently, this compound is likely to have a favorable toxicity profile, similar to aclarubicin (**2**) and *N,N*-dimethyl doxorubicin (**3**), and would therefore be of high interest for further evaluation.

## Results and Discussion

The 26-compound anthracycline library subject of the here-presented studies is depicted in Figure 1. It is comprised of five compounds (**1**–**5**) we have reported on earlier,^13−15^ which we compare to 21 structural analogues (**6**–**26**). One distinguishing feature that determined (the lack of) DNA damage induction in our previously reported studies on doxorubicin analogues is the addition of two methyl groups to the amine group in the daunosamine moiety: while doxorubicin (**1**) induces DNA double strand breaks, its *N,N*-dimethylated analogue **3** does not.^13^ To further probe the relevance of the tertiary amine in the daunosamine moiety of these structures on DNA damage efficiency (and by extension, on toxic side effects) we prepared tertiary amines **6**–**9** featuring a cyclic azetidine (**6**), a pyrrolidine (**7**), a piperidine (**8**) and a morpholine moiety (**9**), respectively. Compounds **10**–**12** are included to examine whether the basic amine is required at all for any of the three biological activities (DNA damage, chromatin damage and cytotoxicity), with the amine either masked as an azide (**10**), substituted for an alcohol (**11**) or removed altogether (**12**). Compounds **13** and **14** are regio-isomers of doxorubicin (**1**) and *N,N*-dimethyldoxorubicin (**3**), respectively, featuring a 2,3-dideoxy-3-aminofucose (*N,N*-dimethylated in **14**) and have been designed to establish the relevance of the location of the basic (alkylated) amine within the glycan moiety of doxorubicin (**1**). The clinically used drugs daunorubicin (**15**) and idarubicin (**17**), differ from doxorubicin (**1**) in the nature of the aglycone while they feature the same daunosamine sugar moiety. To establish whether dimethylation of the amine removes DNA damaging activity, we included their respective *N,N*-dimethyl analogues **16** and **18** in this work. Compounds **19**–**24** comprise daunosamine/rhodosamine pairs featuring a number of alternative tetracyclic aglycones. Compounds **25** and **26** are composed of the idarubicin aglycone and the aclarubicin trisaccharide, with the latter again dimethylated at the daunosamine nitrogen. Compounds **8**,^16^**9**,^17^**10**,^18^**11**,^19^**13**,^20^**16**,^16^**21**,^21^**23**^22,23^ and **24**(24) have been described previously, compounds **1**, **2**, **15** and **17** are commercially available and **6**, **7**, **12**, **14**, **18**, **19**, **20**, **22**, **25**, **26**, **27** and **28** were newly synthesized (syntheses are detailed in the Supporting Information). Many of these compounds have had their cytotoxicity evaluated in past studies, and at times the DNA damage capacity has been included. However, these data are fragmented, because of the use of different methods, cell lines or (animal) models. Additionally, the induction of histone eviction has been shown by us to be a better determinant of cytotoxicity than DNA damage, and this had not yet been evaluated for **6**–**14**, **16** and **18**–**26**. As such, this work presents the assessment of compounds **1**–**26** for their potency to effect three biological processes: the cytotoxicity in three relevant cancer cell lines, DNA double strand break formation and chromatin damage via histone eviction.

### Cytotoxicity of Anthracycline Derivatives

**Figure 2:**
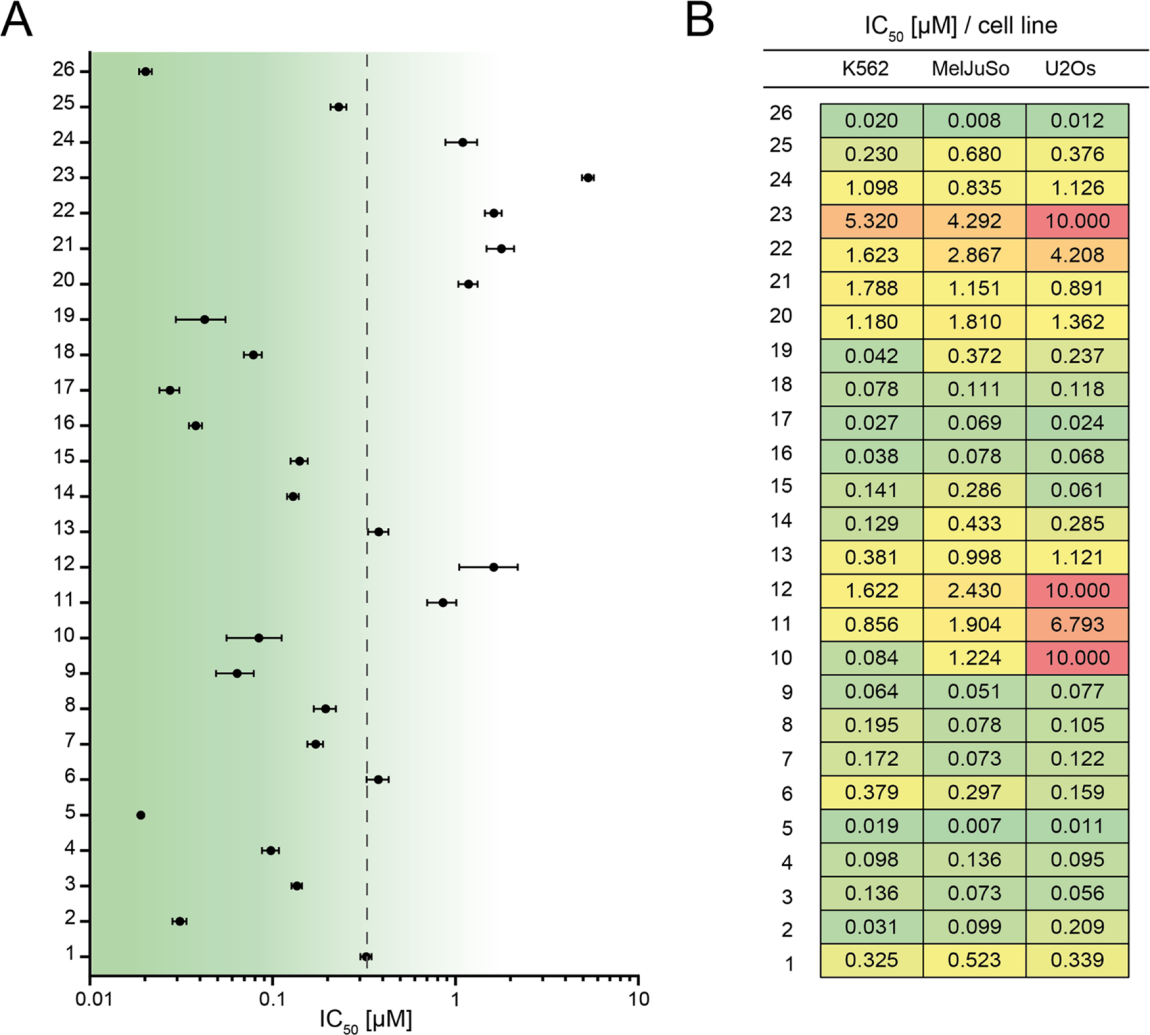
Cytotoxic potency of anthracycline derivatives **1–26** to three different tumor cell lines. (A) IC_50_ values are plotted for all derivatives tested in the human myelogenous leukemia cell line K562. The *Y*-axis shows the numbers of the structures shown in Figure 1. The dotted line indicates the IC_50_ for doxorubicin. (B) IC_50_ values for the 26 anthracycline variants tested in human myelogenous leukemia cell line K562, human melanoma cell line MelJuSo and human osteosarcoma cell line U2OS.

Anthracyclines are often used in the treatment of acute myeloid leukemia and other hematological malignancies. Therefore, the human myelogenous leukemia cell line K562 was used to determine the cytotoxicity of our set of anthracycline variants *in vitro*. The cytotoxicity of all variants (**1**–**26**) was tested using a short-term cell viability assay. Briefly, cells were treated for 2 h with the different anthracycline variants at the indicated concentrations, and cell viability was determined 72 h after treatment. The IC_50_ values for all analogues are plotted (Figure 2A). Within the set of cyclic (tertiary) amines, azetidine (**6**) proved equally effective when compared to the parental drug (**1**), of which the IC_50_ is depicted with a dotted line. The other three cyclic amines (**7**–**9**) were more effective than doxorubicin (**1**), with an IC_50_ similar to that of *N,N*-dimethyldoxorubicin (**3**). This is in line with our earlier observation that *N,N*-dimethylated anthracyclines, such as **3** and **4**, are more cytotoxic than their free-amine counterparts.^14,15^ Of the three doxorubicin derivates not containing a basic amine, variants **11** and **12** are considerably less cytotoxic than doxorubicin (**1**), while azido-doxorubicin (**10**) proved to be almost 4-fold more potent. Relocation of the amine moiety from the 3′- to the 4′-position in the sugar, as in **13** and **14**, did not markedly change the IC_50_ for these compounds compared to their original counterparts **1** and **3**, respectively. Removal of the aglycone carbonyl function (as in **19**–**22**) generally did not improve cytotoxicity when compared to the parent compounds. A notable exception is 13-deoxydaunorubicin (**19**), which is nearly equipotent to the most cytotoxic free amine anthracycline in our hands—idarubicin (**17**). Compounds bearing an aglycone with three phenol groups (**23** and **24**) turned out to be poorly cytotoxic. However, they were both more cytotoxic than their aclarubicin-aglycone bearing counterparts described before.^15^ The idarubicin-derived trisaccharides in this set (**25** and **26**) were significantly more cytotoxic than doxorubicin (**1**), and *N,N*-dimethylated-idarubicin trisaccharide (**26**) was the most effective compound of this set; active at low nanomolar concentrations. In fact, with an IC_50_ of 20 nM in K562 cells this variant is 16 times more cytotoxic than doxorubicin (**1**). In general, the observed cytotoxic activity appeared consistent across cell types, since similar cytotoxicity profiles were observed in cell lines from different cancer origins (Figure 2B), however with some exceptions (for instance: **10**–**12** and **23**).

Overall, evaluation of the cytotoxic activity of the full set of new anthracycline derivatives produced seven compounds that were less effective (**11**, **12**, and **20**–**24**) than doxorubicin (**1**), and two compounds (**6** and **13**) with a similar IC_50_ as doxorubicin (**1**). Interestingly, ten newly synthesized compounds showed to be (far) more effective than doxorubicin in the three tested cell lines.

### Evaluation of DNA Damaging Activity

Anthracycline variants that are used in the clinic display two modes of action: the induction of DNA damage via targeting of topoisomerase II and/or chromatin damage through eviction of histones.^25^ DNA damage activity does contribute to the cytotoxicity of these (and other chemotherapeutics), however, we have shown that DNA damage conspires with chromatin damage to induce the severe therapy limiting side effects of this class of drugs.^13^ Therefore, it is imperative to assess the different mechanisms of action of each of the anthracycline variants.

**Figure 3:**
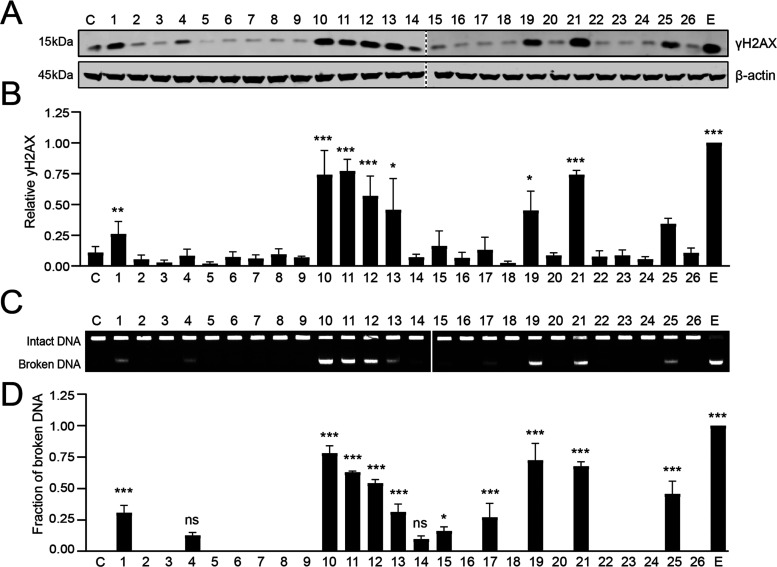
DNA damage capacity of the full set of anthracycline derivatives **1**–**26**, C; unmanipulated control. (A) K562 cells were treated for 2 h with 10 μM of the indicated compounds, etoposide [E] was used as positive control. γH2AX levels were examined by Western blot. Actin was used as a loading control and molecular weight markers are indicated. (B) Quantification of γH2AX signal normalized to the loading control. Results are presented as mean ± SD of three independent experiments. Ordinary one-way ANOVA with Dunnett’s multiple comparison test. *P < 0.05, **P < 0.01, ***P < 0.001. (C) DNA double strand breaks were directly visualized by CFGE. The position of intact and broken DNA is indicated. (D) Quantification of broken DNA relative to total DNA as analyzed by CFGE. Etoposide [E] was used as positive control. Results are presented as mean ± SD of three independent experiments. Ordinary one-way ANOVA with Dunnett’s multiple comparison test. *P < 0.05, ***P < 0.001, ns = not significant.

In response to DNA double strand break formation, histone H2AX is phosphorylated, then called γH2AX.^26^ The levels of γH2AX thus reflect the presence of DNA double strand breaks. Therefore, we determined the DNA-damaging capacity of this set of anthracyclines by assessing γH2AX protein levels using Western blot analysis. K562 cells were treated with the indicated compounds (**1**–**26**) at a concentration of 10 μM, corresponding to serum peak levels for doxorubicin in cancer patients at standard treatment.^27^ Etoposide (a podophyllotoxin-based topoisomerase II inhibitor) was included as positive control for DNA break formation (Figure 3A,B). Variants with a tertiary amine on the reducing fucose (**2**–**9**, **16**, **18**, **20**, **22**, **24** and **26**) did not induce DNA damage, in line with results obtained previously for aclarubicin (**2**) and *N,N*-dimethyldoxorubicin (**3**).^13^ Compound **4** and **14** may be exceptions as these compounds induce a slight increase in γH2AX level, similar to earlier observations.^14^ On the other hand, almost all compounds with a primary amine at this position are able to induce DNA double strand breaks. Specifically, the non-basic doxorubicin variants lacking the amine (**10**–**12**), doxorubicin regio-isomer **13**, 13-deoxy-daunorubicin (**19**), 13-deoxy-doxorubicin (**21**) and non-methylated idarubicin-aclarubicin hybrid (**25**) all proved to be very potent DNA damage inducers. Here, the poorly cytotoxic compound **23**, deviated from the rule lacking DNA damage activity, despite its primary amine. A similar trend in γH2AX protein levels was observed for all compounds (**1**–**26**) at lower drug concentrations (1 and 5 μM, Supporting Information Figures S1 and S2).

Some anthracycline variants also cause dissociation of histones from chromatin upon intercalation into DNA,^25^ including the histone variant H2AX. Therefore, the levels of γH2AX might not accurately represent DNA damage when compounds are efficient histone evictors. To determine DNA double strand break induction by the different anthracycline variants at the DNA level, we assessed the DNA damage capacity of our compounds using constant field gel electrophoresis (CFGE)^28^ a direct method to visualize intact and broken DNA (Figure 3C,D). This complementary assay to study DNA damage confirmed the observations on γH2AX protein levels, showing the same trend in the DNA damaging capacity of our series of compounds.

### Evaluation of Chromatin Damage Activity

**Figure 4:**
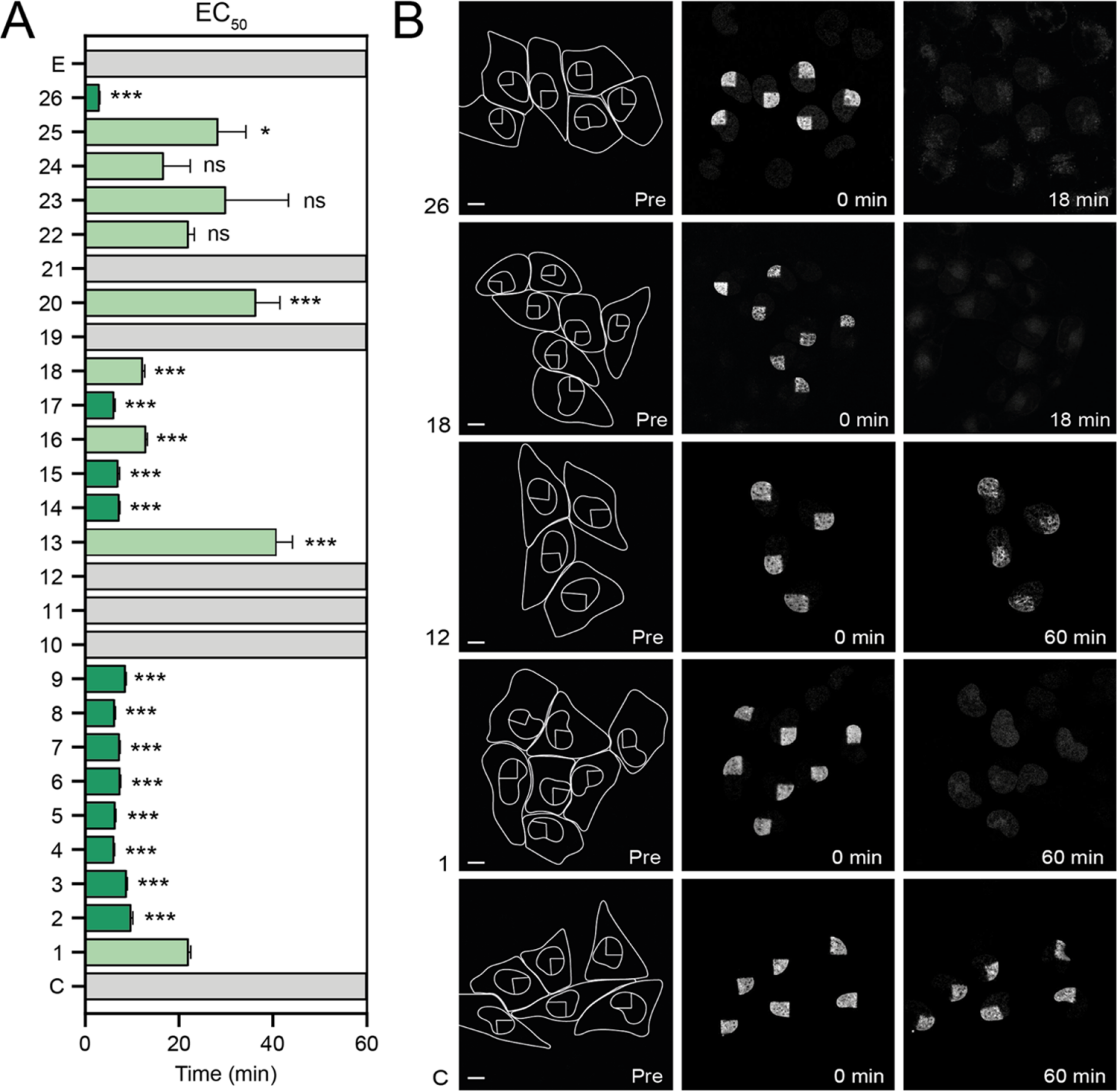
Efficacy of chromatin damage of the set of anthracycline derivatives **1**–**26**. (A) The rate of histone eviction for all derivatives is plotted as EC_50_ (time at which 50% of the initial signal in the photoactivated spot is reduced). Etoposide [E] was used as negative control. Nonlinear regression with sum-of-squares *F* test. *P < 0.05, ***P < 0.001, ns = not significant. (B) Illustration of the effects of indicated compounds (numbers on left side indicate the drug in Figure 1) on eviction of the photoactivated histones. Left panel: drawn cell out line and nucleus with the photoactivated part of the nucleus in living MelJuSo-PAGFP-H2A cells. Middle panel shows the photoactivated histones at the onset of the experiment after compound addition. Photo-activation was monitored by time-lapse confocal microscopy for 1 h in the presence of the indicated compounds at 10 μM. Stills made at 60 min are shown in the right panel. Scale bar, 10 μm.

For previously reported anthracycline variants we have shown that chromatin damage following histone eviction is strongly correlating with cytotoxicity.^14,15^ To visualize histone eviction, part of the nucleus of MelJuSo cells stably expressing PAGFP-H2A was photoactivated. Subsequently, the fluorescence intensity was measured directly after addition of the indicated compounds using timelapse confocal microscopy, as previously described.^12^ For all tested derivatives the rate of histone eviction (EC_50_, the time at which 50% of the initial signal is reduced) was plotted (Figure 4A). Whereas compounds **10**, **11** and **12** are proven effective DNA damage inducers, removal and/or replacing the amine abolished the capacity to evict histones (Figure 4A,B). Furthermore, analogues lacking the aglycone-carbonyl characteristic for both daunorubicin and doxorubicin (**19**, **21**) are unable to evict histones. Likewise, for their *N,N*-dimethylated counterparts (**20** and **22**), the rate of histone eviction was markedly reduced when comparing the deoxy variants to those bearing the original aglycones (**20***versus***16** and **22***versus***3**). In general, variants containing a tertiary amine at the 3′-and 4′-position in the carbohydrate attached to the doxorubicin tetracycle were effective histone evicting compounds (**3**–**9** and **14**), with strong eviction capacity and outperformed doxorubicin (**1**). The derivative with the fastest histone eviction activity was compound **26**. Combining the aclarubicin trisaccharide with the idarubicin aglycone, resulted in variant **26** that outperformed both aclarubicin (**2**) and idarubicin (**17**) in terms of the rate of histone eviction (Figure 4A,B). The aclarubicin/idarubicin hybrid structure (**26**) proved to be the most effective anthracycline variant with respect to histone eviction and this markedly improved chromatin damage activity may explain its superior cytotoxicity. This hypothesis is strengthened by the significant correlation of histone eviction rate and cytotoxicity when evaluating the compounds tested here (Supporting information, Figure S3).

While the alterations on the aglycones in this set of compounds failed to improve the chromatin damage activity of these compounds, shuffling the aglycone and saccharide moiety of proven effective anthracyclines (as for **5** and **26**) was effective in improving the histone eviction capacity of these compounds.

## Conclusions

Anthracyclines have been extensively used in the past decades to treat various types of cancer. Despite their effectivity, the use of anthracyclines in clinical practice is restricted by their severe side effects. Since the functional understanding of the mechanisms underlying the manifestation of off-target toxicities is still incomplete, studying the consequences of small systematic modifications can be valuable in understanding the biological activities of anthracyclines. To do so, we synthesized a diverse set of anthracycline variants with alterations in amine alkylation, replacement/removal of the basic amine and regio-isomers in the sugar moiety. Additionally, exploration of the chemical space in the aglycone yielded novel (dimethyl)amine monosaccharides and trisaccharides with strong cytotoxicity profiles. In total, 10 out of 19 new anthracycline derivatives were more cytotoxic than doxorubicin (**1**). Most notably, structures containing alkylated amines were particularly cytotoxic, while most of the variations on the tetracyclic aglycone did not typically yield more potent analogues. Exceptions are compound **19** and structures containing the idarubicin aglycone present in **17**, **18**, **25** and **26**. Especially the latter *N,N*-dimethylidarubicin-trisaccharide **26** has strong cytotoxicity, with IC_50_ values in the low nanomolar range in three cancer cell lines.

We have shown before that anthracycline variants that solely induce chromatin damage but not DNA double strand breaks still have excellent cytotoxic activity.^14,15^ In line with these results, we now report a significant correlation between the rate of histone eviction and cytotoxicity. The extent to which anthracyclines induce DNA double strand breaks, on the other hand, does not correlate with their cytotoxicity. This was also noted in the clinic, as etoposide (that only induces DNA breaks) is a considerably less potent anti-cancer drug. Interestingly, etoposide also displays milder side effects compared to doxorubicin. This finding is strengthened by the additional biological data presented for 13-deoxy-doxorubicin (**21**). While unable to evict histones, **21** is a very efficient DNA damaging agent. This variant entered phase I/II clinical trials but was never developed further.^29,30^ These observations show that it is imperative to understand the biological consequences of structural variations for rational design of novel anthracyclines. In the development of annamycin, another anthracycline variant that entered phase I/II clinical trials,^31^ several important structural modifications were incorporated.^32^ This analogue is characterized by the absence of the aglycone methoxy group, the introduction of an iodine at the 2′-position of the sugar and the replacement of the primary amine at the 3′-position with an OH group. The absence of the amino group results in reduced basicity, which appears to be at the cost of potency, as is also seen for the cytotoxicity profile of hydroxydoxorubicin (**11**). Therefore, removal of the methoxy on the aglycone seems to be important to increase cytotoxicity, which correlates with our findings on the structural variants with the idarubicin aglycone (**17**, **18**, **25** and **26**) that proved to be very potent.

From the set of anthracycline variants harboring cyclic (tertiary) amines, azetidine (**6**) proved equally effective to doxorubicin, whereas the other three cyclic amines (**7**–**9**) were more cytotoxic. These results are comparable to previous described cytotoxicity profiles in cell lines of different cancer origin.^33,34^ Of the three doxorubicin derivates in which the primary amine was either replaced (**10**, **11**) or removed (**12**), only azido-doxorubicin (**10**) was significantly more cytotoxic to K562 cells than the parent compound. However, the cytotoxicity of this variant in MelJuSo and U2OS cell lines was considerably lower. Another study in which the amino group of daunorubicin was substituted for an azide showed that this variant is also particularly toxic for K562 cells.^35^ This suggests that the improved toxicity seen for this modification might be cell-type specific.

Based on these and earlier data, we may deduce five guidelines related to the potency of anthracyclines:

(1) The main cytotoxic activity of these compounds is associated with histone eviction activity rather than DNA double strand break induction;
(2) Usually, *N,N*-dimethylation eliminates DNA double strand break formation at no cost to cytotoxicity;
(3) Small differences in the tetracycle aglycone structure further contribute to the cytotoxicity, as illustrated by the difference in cytotoxicity between doxorubicin (**1**), 13-deoxydoxorubicin (**21**) and idarubicin (**4**);
(4) The position of the amine in the sugar has minor effects, since placing the amine on either the 3′- or 4′-position does not significantly affect cytotoxicity;
(5) Replacing the amine by an OH or H group strongly reduces cytotoxicity.

These points are combined in *N,N*-dimethylidarubicin trisaccharide (**26**), which is 16-fold more cytotoxic than doxorubicin (**1**). It is also 1.5-fold more cytotoxic than the clinically used variants idarubicin (**17**), which causes various off-target toxicities,^36^ and aclarubicin (**2**) which is only used in China and Japan. Additionally, this compound is more efficient in terms of histone eviction, without inducing any DNA double strand breaks. Further *in vivo* studies are required on the cardiotoxic profile of **26**, to establish whether increased cytotoxicity to cancer cells could enlarge the therapeutic window for cancer patients. Such studies may ultimately yield more effective anthracycline variants with limited adverse toxicity.

## Experimental Section

### Chemistry

The anthracycline analogues **3**–**5** were synthesized as described.^14,15^ Syntheses of compounds **6**–**14**, **16**, **18**–**26** and intermediates are described in the Supporting Information. Compounds are >95% pure by high-performance liquid chromatography (HPLC) analysis.

### Reagents and Antibodies

Doxorubicin was obtained from Accord Healthcare Limited, U.K., aclarubicin (sc-200160) was purchased from Santa Cruz Biotechnology, daunorubicin was obtained from Sanofi, idarubicin was obtained from Pfizer, etoposide was obtained from Pharmachemie (the Netherlands). Primary antibodies used for Western blotting: γH2AX (1:1000, 05-036, Millipore), β-actin (1:10,000, A5441, Sigma). Secondary antibody used for blotting: IRDye 800CW goat anti-mouse IgG (H + L) (926-32210, Li-COR, 1:10,000).

### Cell Culture

K562 cells (B. Pang, Leiden University Medical Center, the Netherlands) were maintained in RPMI-1640 medium supplemented with 8% FCS. MelJuSo cells were maintained in IMDM supplemented with 8% FCS. MelJuSo cells stably expressing PAGFP-H2A were maintained in IMDM supplemented with 8% FCS and G-418, as described.^12^ U2Os cells (ATCC HTB-96) were maintained in DMEM medium supplemented with 8% FCS. Cell lines were maintained in a humidified atmosphere of 5% CO_2_ at 37 °C, regularly tested for the absence of mycoplasma and the origin of cell lines was validated using short tandem repeat (STR) analysis.

### Short-Term Cell Viability Assay

Cells were seeded into 96-well format (2000 cells/well). Twenty-four hours after seeding, cells were treated with indicated compounds for 2 h at various concentrations. Subsequently, compounds were removed, cells were washed and were left to grow for an additional 72 h. Cell viability was measured using the CellTiter-Blue viability assay (Promega). Relative survival was normalized to the untreated control and corrected for background signal.

### Western Blot and Constant-Field Gel Electrophoresis (CFGE)

Cells were seeded into 12-well format (250.000 cells/well), treated with indicated drugs at 10 μM for 2 h. Subsequently, drugs were removed by extensive washing and cells were collected and processed immediately for the assays. For Western blot, cells were lysed directly in SDS-sample buffer (2% SDS, 10% glycerol, 5% β-mercaptoethanol, 60mM Tris–HCl pH 6.8 and 0.01% bromophenol blue). Samples were separated by sodium dodecyl sulphate-polyacrylamide gel electrophoresis (SDS-PAGE) and transferred to a PVDF membrane (Immobilon-P, 0.45 μm, Millipore). Blocking of the filters and antibody incubations were done in PBS supplemented with 0.1 (v/v)% Tween and 5% (w/v) milk powder (Skim milk powder, LP0031, Oxiod). Blots were imaged by the Odyssey Classic imager (Li-Cor). Intensity of bands was quantified using ImageJ or Image Studio software. For CFGE: DNA double strand breaks were quantified by constant-field gel electrophoresis as described.^28^ Images were quantified using ImageJ software.

### Microscopy Analysis

For PAGFP-H2A photoactivation and time-lapse confocal imaging, cells were seeded in a 35 mm glass bottom petri dish (Poly-d-lysine-Coated, MatTek Corporation), and imaged 16 h later as described for 1 h following addition of 10μM of the indicated compounds.^12^ Time-lapse confocal imaging was performed on a Leica SP8 confocal microscope system 63x lens, equipped with a climate chamber. Movies were quantified using Image J software.

### Quantification and Statistical Analysis

Each experiment was assayed in biological triplicate, unless stated otherwise. Error bars denote ±SD. Statistical analyses were performed using Prism 8 software (GraphPad Inc.). ns = significant, *p = < 0.05, **p = < 0.01, ***p = < 0.001

## Supporting Information Available

γH2AX levels induced by compounds **1**–**26** tested at 1 and 5 μM concentrations respectively (Figures S1 and S2); correlation between chromatin- or DNA damage and cytotoxicity for compounds **1**–**26** (Figure S3); detailed synthesis schemes, synthetic procedures; and analytical spectra (H- and C-NMR, COSY and HSQC, HRMS) of compounds **6**–**14**, **16**, **18**–**26** and their synthetic intermediates (PDF)
Molecular formula strings and tabulated biological assay data (CSV)

## Abbreviations Used

PAGFP-H2A: photo-activatable histone H2A.
