# Supplementary material for: Re-Exploring the Anthracycline Chemical Space for Better Anti-Cancer Compounds
Source: J Med Chem. 2023 Aug 10;66(16):11390–8. doi: 10.1021/acs.jmedchem.3c00853 (PMC10461226; doi:10.1021/acs.jmedchem.3c00853)
Supplement: Supplementary file 1 — jm3c00853_si_001.pdf [file jm3c00853_si_001.pdf]

Supporting information for:

Re-Exploring the Anthracycline Chemical Space for Better Anti-Cancer Compounds

Merle A. van Gelder[#,1], Sabina Y. van der Zanden[#,1], Merijn B.L. Vriends[2], Roos A. Wagensveld[1], Gijsbert A. van der Marel[2], Jeroen D.C. Codée[2], Herman S. Overkleeft[2], Dennis P.A. Wander[*,1], Jacques J.C. Neefjes[*,1]

[1] Department of Cell and Chemical Biology, ONCODE Institute, Leiden University Medical Center, Einthovenweg 20, 2333 ZC Leiden, The Netherlands

[2] Leiden Institute of Chemistry, Leiden University, Einsteinweg 55, 2333 CC Leiden, The Netherlands

[#] These authors contributed equally

* Corresponding authors:

Dennis P.A. Wander: d.p.a.wander@lumc.nl

Jacques J.C. Neefjes: j.j.c.neefjes@lumc.nl

Table of Contents

A: Supplemental Figures S3

B: Synthesis of doxorubicinone derivatives **6-14** and accompanying analytical data...........S5

C: Synthesis of (*N,N*-dimethyl) doxorubicin derivatives **15-24**, differing in the aglycone part and accompanying analytical data S73

D: Synthesis of idarubicinone trisaccharides **25** and **26** and accompanying analytical data S113

E: HRMS traces of compounds **6-26**..S124

F: HPLC traces...S143

G: References S145

A: Supplemental Figures

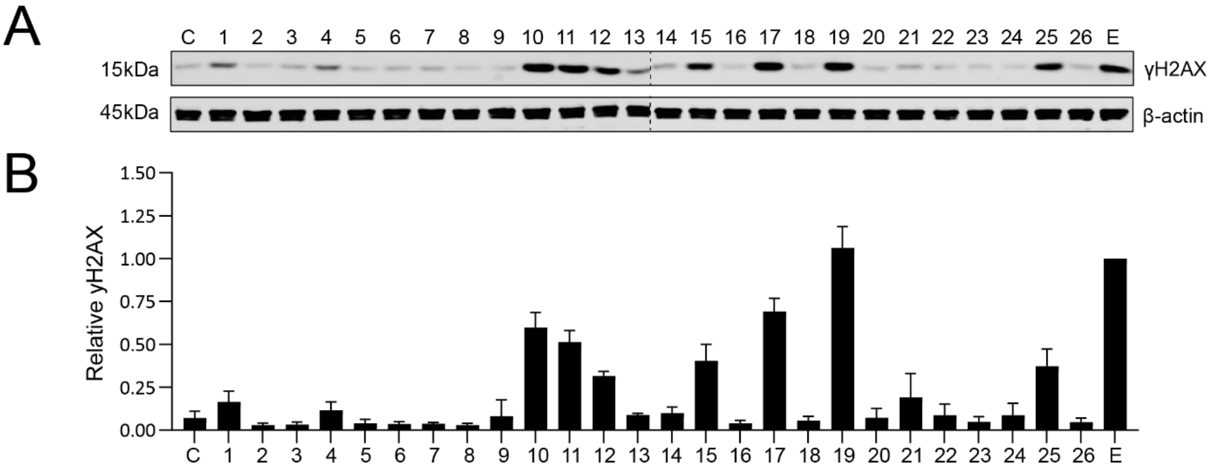

Figure S1 – DNA damage capacity of the full set of anthracycline derivatives **1-26**. Numbers correspond to the structures in Figure 1, C; unmanipulated control. (A) K562 cells were treated for 2 h with 1 μM of the indicated compounds, etoposide [E] was used as positive control. γH2AX levels were examined by Western blot. Actin was used as a loading control, and position of molecular weight markers is indicated. (B) Quantification of γH2AX signal normalized to the loading control. Results are presented as mean ± SD of three independent experiments.

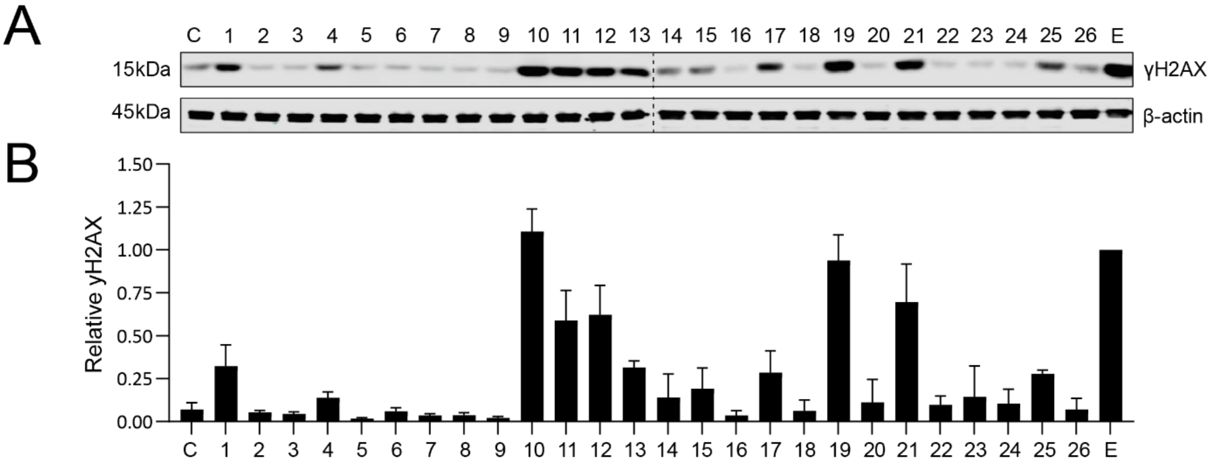

Figure S2 – DNA damage capacity of the full set of anthracycline derivatives **1-26**. Numbers correspond to the structures in Figure 1, C; unmanipulated control. (A) K562 cells were treated for 2 h with 5 μM of the indicated compounds, etoposide [E] was used as positive control. γH2AX levels were examined by Western blot. Actin was used as a loading control, and position of molecular weight markers is indicated. (B) Quantification of γH2AX signal normalized to the loading control. Results are presented as mean ± SD of three independent experiments.

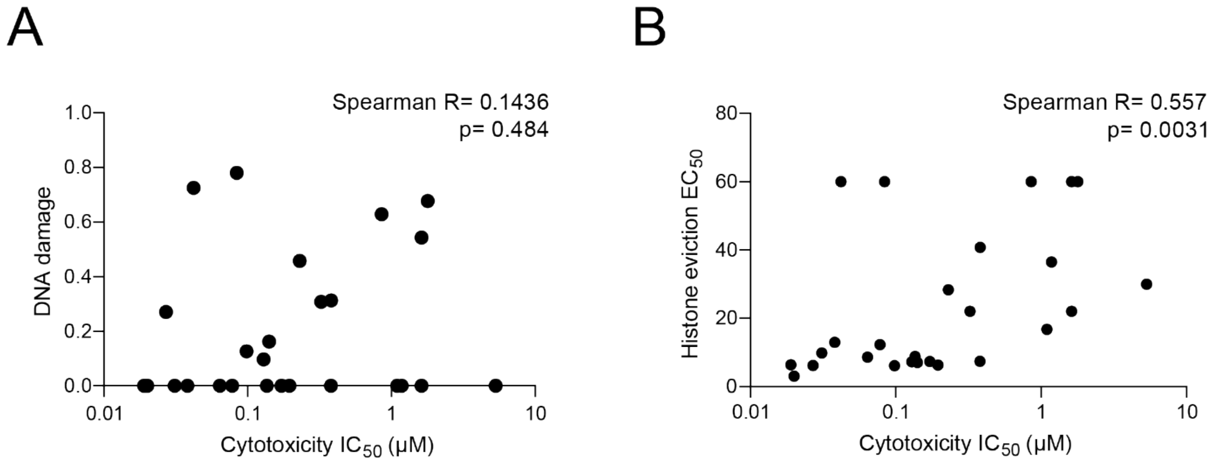

Figure S3 – (A) Correlation values between cytotoxicity (IC_{50}) and DNA damaging capacity (expressed as fraction of broken DNA). Graphical representation of the relationship between cytotoxicity and DNA damage capacity. (B) Correlation values between cytotoxicity (IC_{50}) and rate of histone eviction (EC_{50}). Graphical representation of the relationship between cytotoxicity and histone eviction rate.

B: Synthesis of doxorubicinone derivatives 6-14 and accompanying analytical data

All reagents were of commercial grade and used as received. Traces of water from reagents were removed by co-evaporation with toluene in reactions that required anhydrous conditions. All moisture/oxygen sensitive reactions were performed under an argon atmosphere. DCM used in the glycosylation reactions was dried with flamed 4Å molecular sieves before being used. Reactions were monitored by TLC analysis with detection by UV (254 nm) and where applicable by spraying with 20% sulfuric acid in EtOH or with a solution of $(NH_4)_6Mo_7O_{24}\cdot 4H_2O$ (25 g/L) and $(NH_4)_4Ce(SO_4)_4\cdot 2H_2O$ (10 g/L) in 10% sulfuric acid (aq.) followed by charring at ~150 °C. Flash column chromatography was performed on silica gel (40-63μm). ^1H and ^{13}C spectra were recorded on a Bruker AV 400 and Bruker AV 500 in $CDCl_3$, CD_3OD, pyridine-d5 or D_2O. Chemical shifts (δ) are given in ppm relative to tetramethylsilane (TMS) as internal standard (^1H NMR in $CDCl_3$) or the residual signal of the deuterated solvent. Coupling constants (J) are given in Hz. All ^{13}C spectra are proton decoupled. Column chromatography was carried out using silica gel (0.040-0.063 mm). Size-exclusion chromatography was carried out using Sephadex LH-20, using DCM:MeOH (1:1, v/v) as the eluent. Neutral silica was prepared by stirring regular silica gel in aqueous ammonia, followed by filtration, washing with water and heating at 150°C overnight. High-resolution mass spectrometry (HRMS) analysis was performed with a LTQ Orbitrap mass spectrometer (Thermo Finnigan), equipped with an electronspray ion source in positive mode (source voltage 3.5 kV, sheath gas flow 10 mL/min, capillary temperature 250 °C) with resolution R = 60000 at m/z 400 (mass range m/z = 150 – 2000) and dioctyl phthalate (m/z = 391.28428) as a "lock mass", or with a Synapt G2-Si (Waters) , equipped with an electronspray ion source in positive mode (ESI-TOF), injection via NanoEquity system (Waters), with LeuEnk (m/z = 556.2771) as "lock mass". Eluents used: $MeCN:H_2O$ (1:1 v/v) supplemented with 0.1% formic acid. The high-resolution mass spectrometers were calibrated prior to measurements with a calibration mixture (Thermo Finnigan).

General Procedure A: *N*-cyclic doxorubicins: To a solution of doxorubicin·HCl in DMF (0.033M) were added triethylamine (3 eq) and the corresponding diiodoalkane or diiodoether (18 eq). The mixture was allowed to stir for 5 days, or until LCMS showed disappearance of the starting material. It was then poured into H_2O, extracted with $CHCl_3$ repetitively, dried over Na_2SO_4 and concentrated *in vacuo*. Column chromatography on neutral silica (MeOH:DCM) gave the title cyclic amines.

General procedure B: Steglich esterification with *ortho*-cyclopropylbenzoic acid: To a solution of the hemiacetal in DCM (0.1 M) were added DIPEA (9 eq), DMAP (1 eq), EDCI·HCl (3.5 eq) and freshly saponified *o*-cyclopropylethynylbenzoic acid[4] (3 eq). After disappearance of the starting hemiacetal, the mixture was diluted with DCM and washed with sat. aq. $NaHCO_3$ and brine, dried over $MgSO_4$ and concentrated *in vacuo*. Column chromatography gave the corresponding anomeric benzoates.

General procedure C: Glycosylation of alkynylbenzoate donors: To a solution of the alkynylbenzoate donor and anthracyclinone acceptor (1.5 eq) in DCM (0.05 M), were added activated molecular sieves (4Å) and the mixture was stirred for 30 minutes. Subsequently, a freshly prepared 0.1 M DCM solution of PPh_3AuNTf_2 (prepared by stirring 1:1 PPh_3AuCl and $AgNTf_2$ in DCM for 30 minutes) (0.1 eq) in DCM was added dropwise. After stirring 30 minutes, the mixture was filtered and concentrated *in vacuo*. Column chromatography gave the title anthracyclinone saccharides.

General procedure D: *N*-Alloc removal: A solution of the allyloxycarbamate and *N,N*-dimethylbarbituric acid (4.5 eq) in DCM (0.01 M) was degassed for 5 minutes. Then, $Pd(PPh_3)_4$ (0.05

eq) was added and the mixture was allowed to stir for 30 minutes. The reaction was then directly subjected to column chromatography to give the title amines.

General procedure E: Desilylation: A solution of the amine in pyridine (0.01-0.05 M) was cooled to 0 ^oC. HF·pyridine (70 wt% HF, ± 3.8 mL/mmol starting material, 146 eq) was added and the reaction mixture was stirred for 1-5h at this temperature. Solid $NaHCO_3$ was added to quench and the mixture was stirred until cessation of effervescence. Salts were then filtered off and the filtrate was diluted with DCM (10 volumes), washed with H_2O, dried over Na_2SO_4 and concentrated *in vacuo*. Column chromatography on neutralized silica gel gave the title anthracyclines.

General procedure F: IDCP-mediated glycosylation: To a solution of the glycosyl acceptor (1 eq) and the glycosyl donor (1.3 eq) in Et_2O:DCE (0.033M, 4:1 v/v), activated molecular sieves (4Å) were added. The mixture was stirred for 30 minutes and then, at 10^oC, iodonium dicollidine perchlorate (4 eq) was added. After 60 minutes, it was diluted with EtOAc and filtered, washed with 10% aq. $Na_2S_2O_3$, 1M $CuSO_4$ solution twice, H_2O and then dried over $MgSO_4$. Concentration *in vacuo* and column chromatography (5:95 – 10:90 EtOAc:pentane) of the residue gave the di- or trisaccharide.

General procedure G: *p*-Methoxyphenolate oxidative deprotection: To a solution of the *p*-methoxyphenyl glycoside in 1:1 MeCN:H_2O (0.02M, v/v) were added NaOAc (10 eq) and then $Ag(DPAH)_2 \cdot H_2O$[5] (2.1 eq for trisaccharides, 4 eq for monosaccharides) portionwise over 30 minutes at 0^oC. The mixture was stirred until disappearance of the starting material; after which it was poured into sat. aq. $NaHCO_3$. This was then extracted with DCM thrice, dried over $MgSO_4$ and concentrated *in vacuo* to give the crude lactols.

3'-Desamino-3'-azetidino-doxorubicin (6)

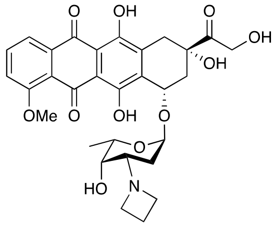

Prepared according to General Procedure A from doxorubicin·HCl (50 mg) and diiodopropane to give the title compound as a red solid (17 mg, 29 µmol, 34%). ^1H NMR (400 MHz, Methanol-d_4) δ 7.81 – 7.57 (m, 2H), 7.41 (d, *J* = 8.0 Hz, 1H), 5.39 (d, *J* = 3.6 Hz, 1H), 4.78 – 4.62 (m, 2H), 4.15 (q, *J* = 6.6 Hz, 1H), 3.94 (d, *J* = 17.7 Hz, 7H), 3.69 (d, *J* = 2.7 Hz, 1H), 3.41 (s, 1H), 2.97 – 2.87 (m, 1H), 2.68 (d, *J* = 18.4 Hz, 1H), 2.34 (p, *J* = 7.8 Hz, 2H), 2.26 (d, *J* = 14.6 Hz, 1H), 2.05 (dd, *J* = 14.6, 4.9 Hz, 1H), 1.87 (dt, *J* = 9.5, 4.6 Hz, 1H), 1.77 (td, *J* = 12.7, 3.9 Hz, 1H), 1.26 (d, *J* = 6.6 Hz, 3H). ^{13}C NMR (101 MHz, MeOD) δ 214.7, 187.6, 187.4, 162.3, 157.2, 155.9, 137.2, 135.9, 135.4, 135.1, 121.1, 120.3, 112.2, 111.9, 100.9, 77.0, 71.1, 67.9, 65.7, 64.7, 62.0, 57.1, 53.5, 37.1, 33.6, 26.5, 17.2, 16.9. HRMS: $[M + H]^+$ calculated for $C_{30}H_{34}NO_{11}$ 584.2132; found 584.2129.

3'-Desamino-3'-pyrrolidino-doxorubicin (7)

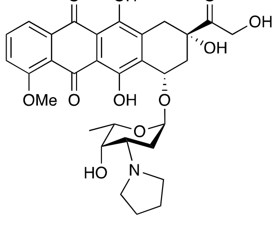

Prepared according to General Procedure A from doxorubicin·HCl (50 mg) and diiodopropane to give the title compound as a red solid (27 mg, 45 µmol, 53%). HRMS: $[M + H]^+$ calculated for $C_{31}H_{36}NO_{11}$ 598.2288; found 598.2291.

3'-Desamino-3'-pyrrolidino-doxorubicin (8)

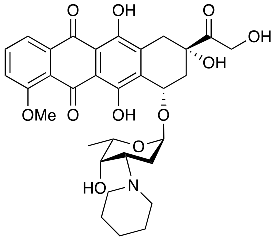

Prepared according to General Procedure A from doxorubicin·HCl (100 mg) and diiodopentane to give the title compound as a red solid (81 mg, 0.13 mmol, 77%). ^{1}H NMR (500 MHz, Chloroform-d_3) δ 14.63 (s, 1H), 13.57 (s, 1H), 8.17 – 8.01 (m, 1H), 7.77 (t, *J* = 8.1 Hz, 1H), 7.47 (d, *J* = 8.5 Hz, 1H), 5.89 (d, *J* = 3.5 Hz, 1H), 5.61 (d, *J* = 20.0 Hz, 1H), 5.53 – 5.39 (m, 2H), 4.79 (q, *J* = 6.6 Hz, 1H), 4.46 (s, 1H), 3.98 (s, 3H), 3.66 – 3.36 (m, 6H), 3.23 (t, *J* = 6.9 Hz, 1H), 2.88 (dt, *J* = 14.5, 2.3 Hz, 1H), 2.59 (dd, *J* = 14.5, 5.3 Hz, 1H), 2.46 (dh, *J* = 17.0, 3.8 Hz, 2H), 1.81 (dtt, *J* = 11.1, 8.1, 4.6 Hz, 1H), 1.74 – 1.57 (m, 4H), 1.53 (d, *J* = 6.5 Hz, 3H), 1.44 (p, *J* = 7.5 Hz, 1H), 1.40 – 1.22 (m, 6H). ^{13}C NMR (126 MHz, $CDCl_3$) δ 215.8, 187.6, 161.9, 157.6, 156.1, 121.5, 120.0, 112.2, 111.9, 101.1, 76.9, 70.9, 68.3, 66.5, 66.4, 62.0, 57.2, 53.9, 51.1, 37.9, 34.2, 28.0, 24.4, 17.9, 8.3, 7.8. HRMS: $[M + H]^+$ calculated for $C_{32}H_{38}NO_{11}$ 612.2445; found 612.2242.

3'-Desamino-3'-morpholino-doxorubicin (9)

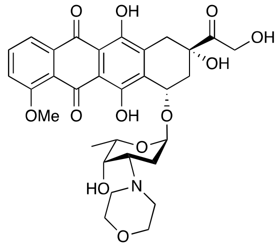

Prepared according to General Procedure A from doxorubicin·HCl (50 mg) and bis(2-iodo)ethyl ether to give the title compound as a red solid (32 mg, 52 μmol, 60%). ^{1}H NMR (400 MHz, $CDCl_3$) δ 13.95 (s, 1H), 13.20 (s, 1H), 8.01 (dd, *J* = 7.7, 1.0 Hz, 1H), 7.79 (t, *J* = 8.1 Hz, 1H), 7.46 – 7.36 (m, 1H), 5.56 (d, *J* = 3.1 Hz, 1H), 5.28 (dd, *J* = 4.1, 2.1 Hz, 1H), 4.74 (d, *J* = 15.8 Hz, 3H), 4.09 (s, 3H), 3.96 (q, *J* = 6.7 Hz, 1H), 3.71 (dt, *J* = 12.3, 4.1 Hz, 6H), 3.23 (dd, *J* = 18.8, 1.9 Hz, 1H), 2.97 (d, *J* = 18.8 Hz, 1H), 2.49 – 2.32 (m, 4H), 2.17 (dd, *J* = 14.7, 4.1 Hz, 1H), 1.78 (dd, *J* = 11.2, 4.1 Hz, 2H), 1.39 (d, *J* = 6.6 Hz, 3H). ^{13}C NMR (101 MHz, MeOD) δ 213.8, 187.2, 186.7, 161.1, 156.4, 155.7, 136.0, 135.5, 133.9, 133.6, 125.6, 120.9, 120.0, 118.6, 111.6, 111.5, 101.1, 70.0, 67.1, 66.9, 65.6, 65.1, 58.8, 56.8, 49.8, 35.6, 34.0, 30.4, 29.8, 27.5, 17.3. HRMS: $[M + H]^+$ calculated for $C_{31}H_{36}NO_{12}$ 614.2238; found 614.2241.

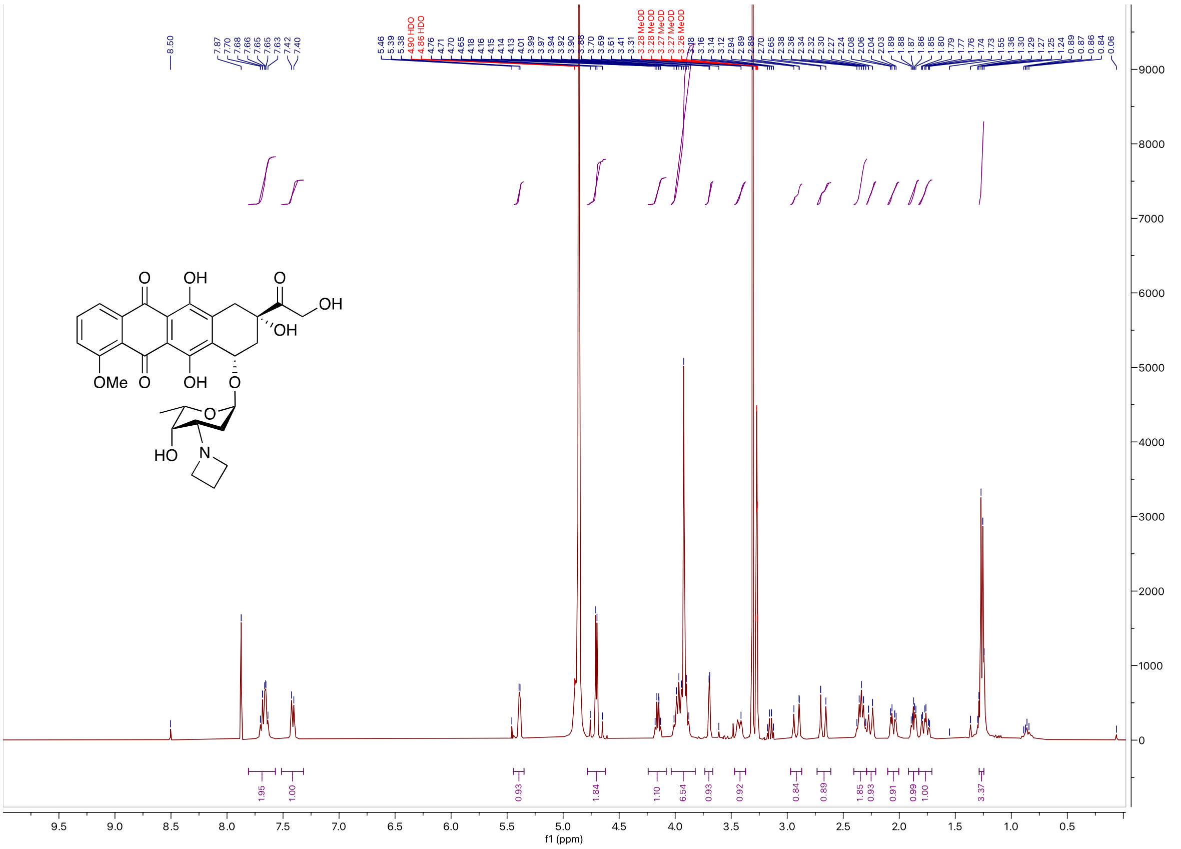
8.50
7.87 7.70 7.68 7.66 7.65 7.65 7.63 7.42 7.40
5.46 5.39 5.38
4.90 HDO
4.86 HDO
4.76 4.71 4.70 4.65 4.18 4.16 4.15 4.14 4.13 4.01 3.99 3.97 3.94 3.92 3.90 3.88 3.70 3.69 3.61 3.41 3.31
3.28 MeOD
3.28 MeOD
3.27 MeOD
3.27 MeOD
3.26 MeOD
3.18 3.16 3.14 3.12 2.94 2.89 2.89 2.70 2.65 2.38 2.36 2.34 2.32 2.30 2.27 2.24 2.08 2.06 2.04 2.03 1.89 1.88 1.87 1.86 1.85 1.80 1.79 1.77 1.76 1.74 1.73 1.55 1.36 1.30 1.29 1.27 1.25 1.24 0.89 0.87 0.86 0.84 0.06
OH
O
OH
O
OH
OMe
O
OH
O
O
HO
N
1.95 1.00 0.93 1.84 1.10 6.54 0.93 0.92 0.84 0.89 1.85 0.93 0.91 0.99 1.00 3.37
f1 (ppm)

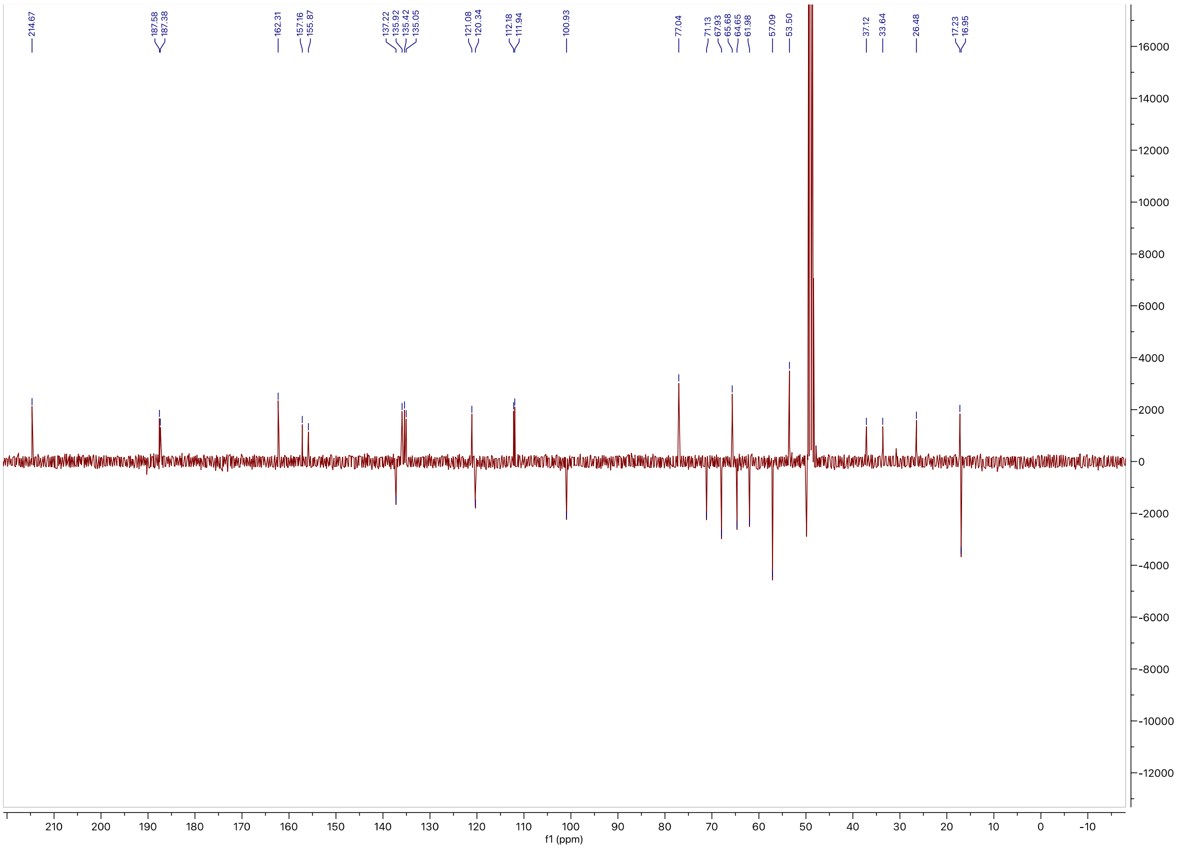
214.67
187.58 187.38
162.31
157.16 155.87
137.22 135.92 135.42 135.05
121.08 120.34
112.18 111.94
100.93
77.04
71.13 67.93 65.68 64.65 61.98
57.09
53.50
37.12
33.64
26.48
17.23 16.95
f1 (ppm)

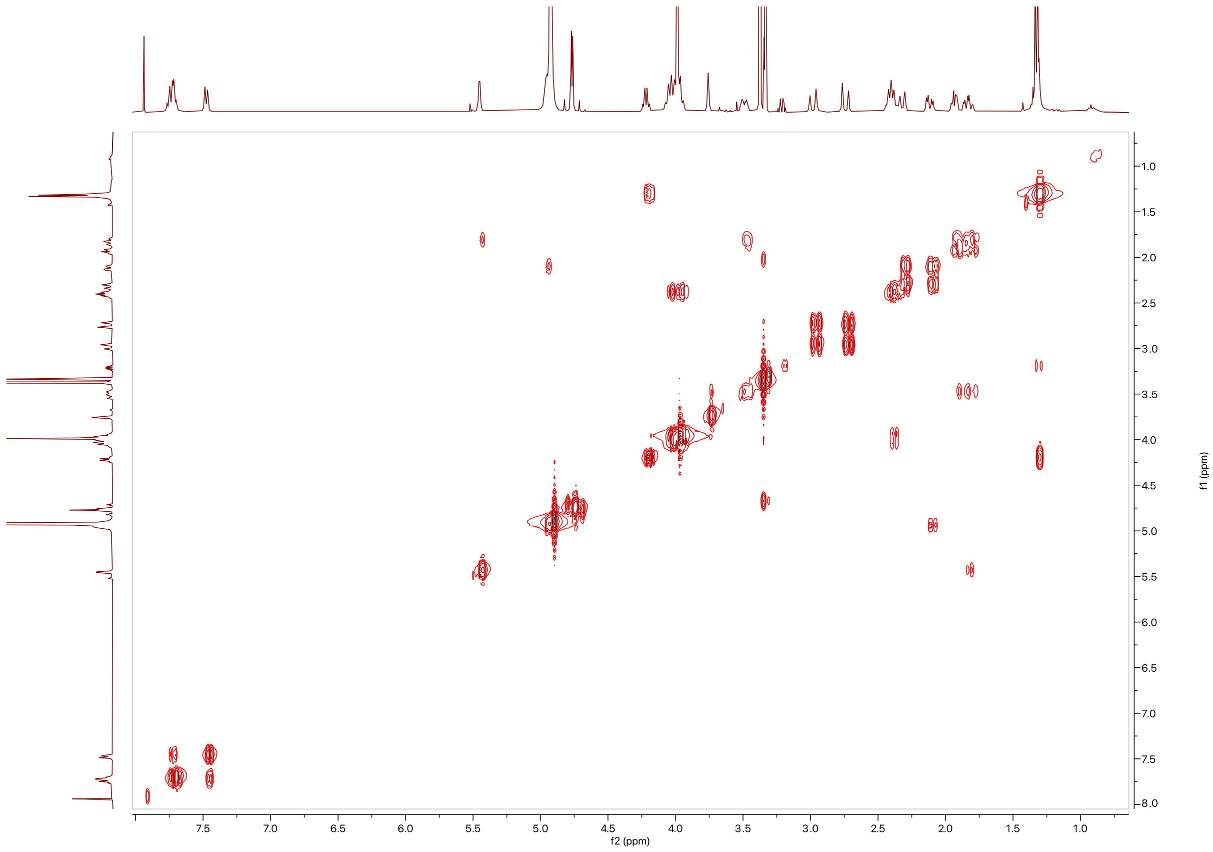
f2 (ppm)
f1 (ppm)

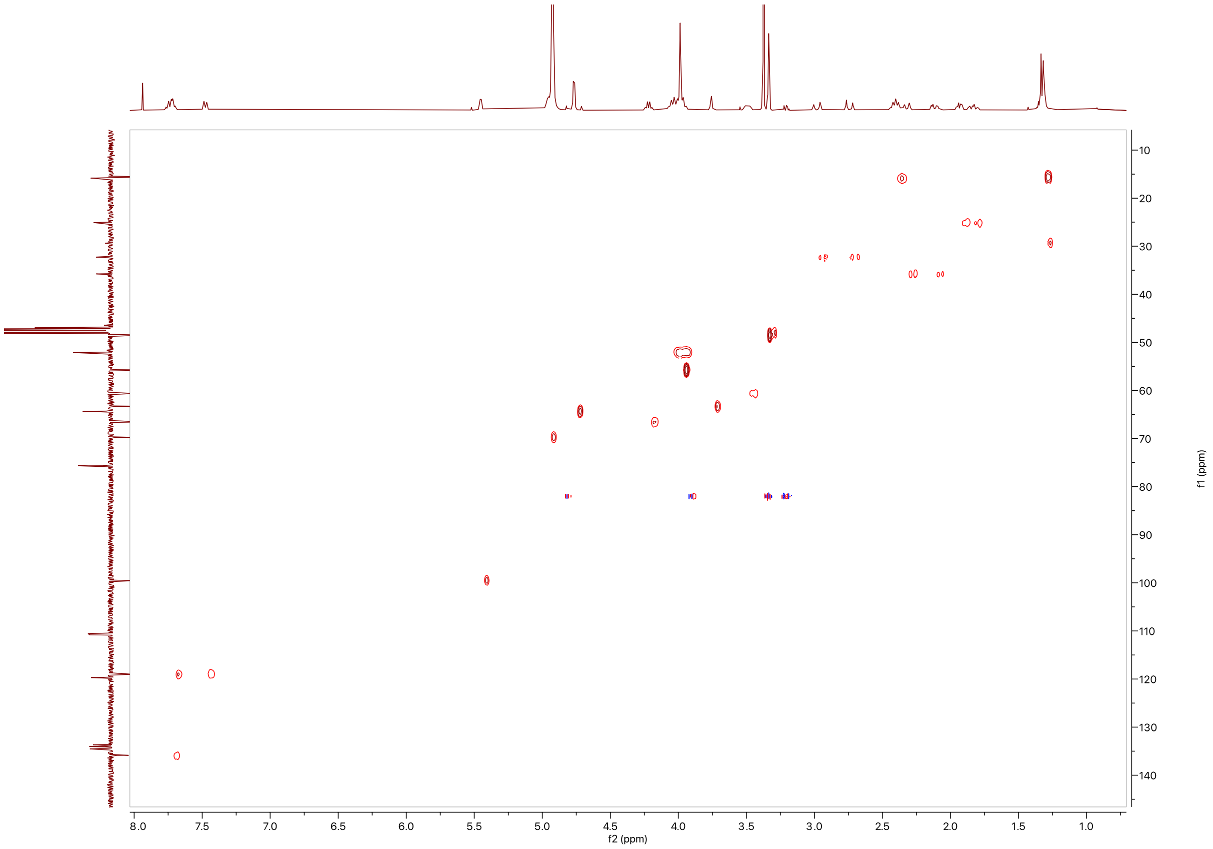
f2 (ppm)
f1 (ppm)

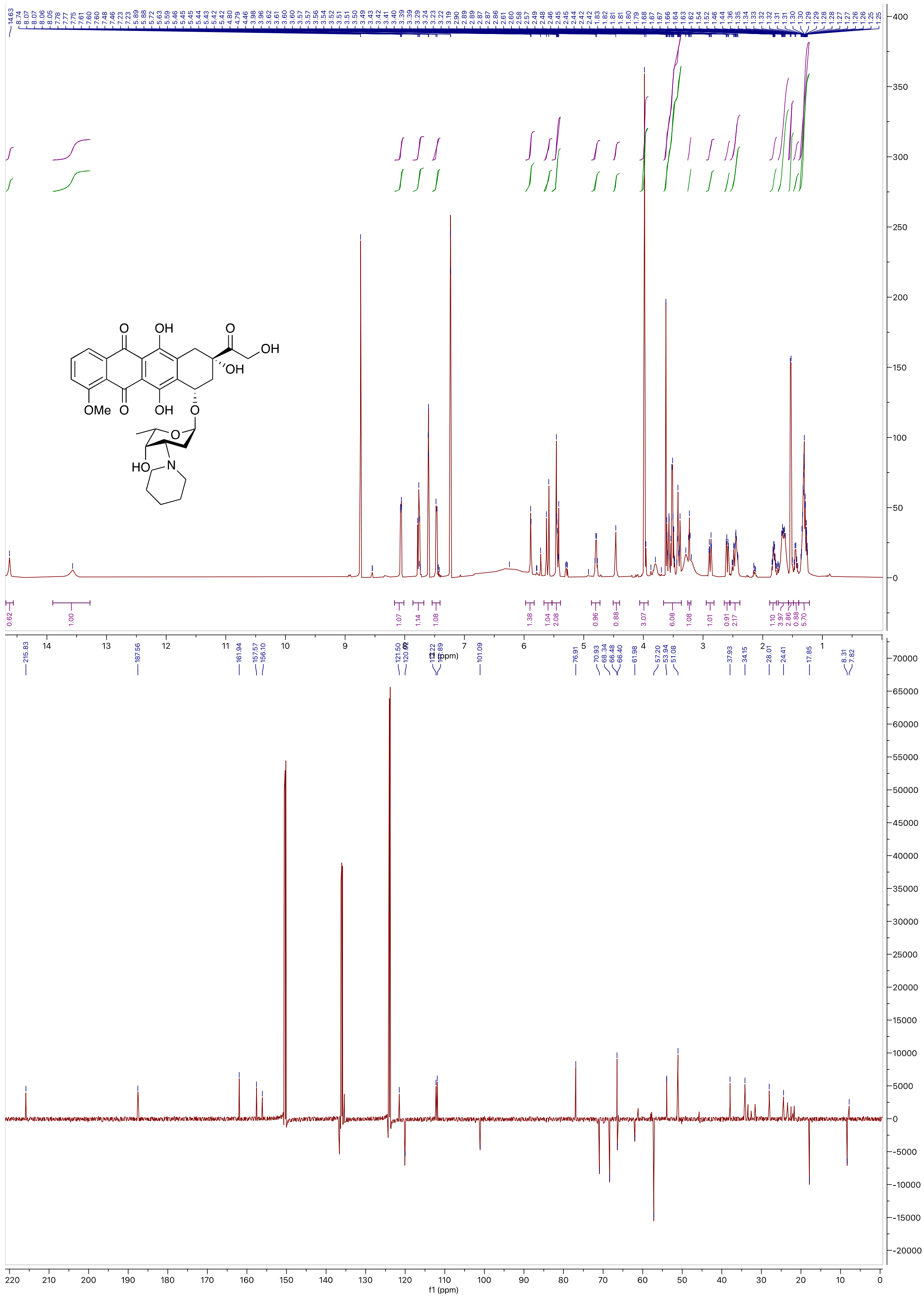
OH
O
OH
OH
OMe
O
OH
O
O
HO
N
215.83
187.56
161.94
157.57
156.10
121.50
112.22
111.89
101.09
76.91
70.93
68.34
66.48
66.40
61.98
57.20
53.94
51.08
37.93
34.15
28.01
24.41
17.85
8.31
7.82
f1 (ppm)

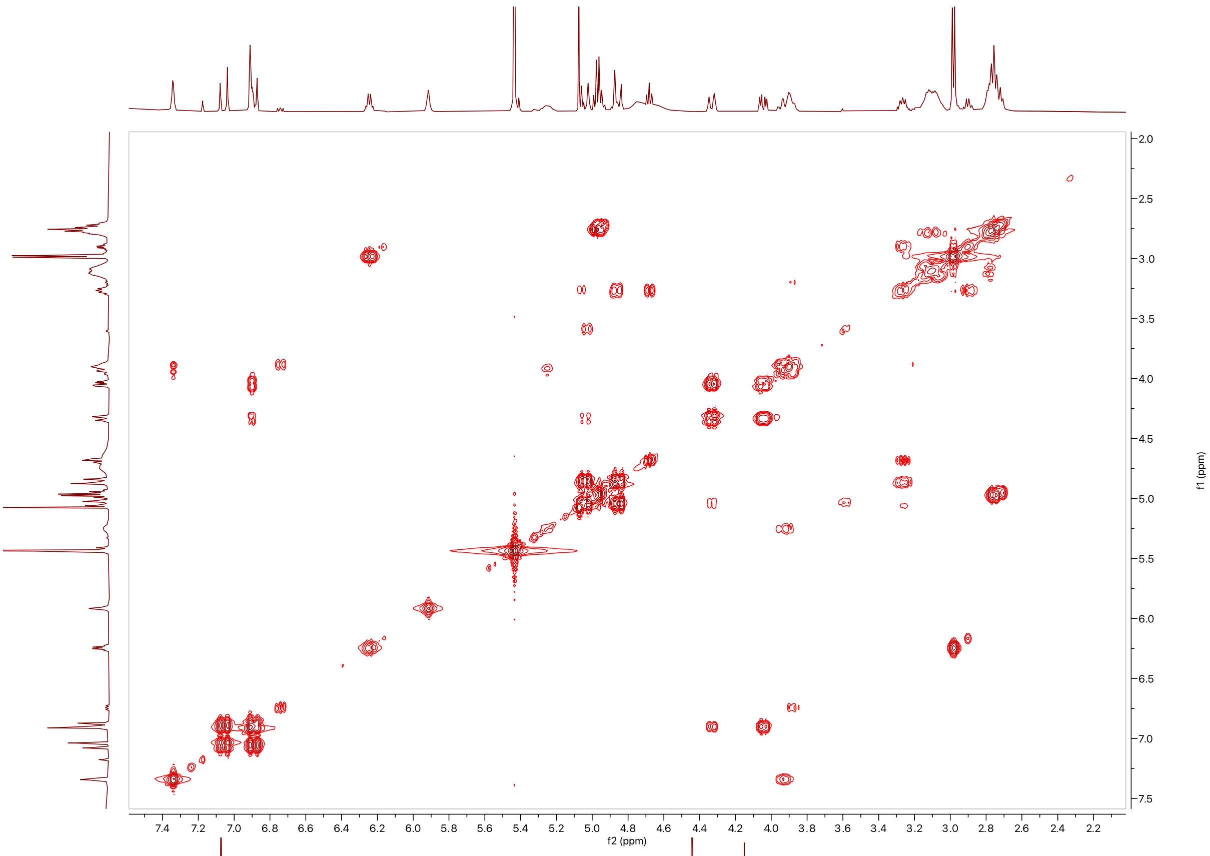
7.4
7.2
7.0
6.8
6.6
6.4
6.2
6.0
5.8
5.6
5.4
5.2
5.0
4.8
4.6
4.4
4.2
4.0
3.8
3.6
3.4
3.2
3.0
2.8
2.6
2.4
2.2
f2 (ppm)
2.0
2.5
3.0
3.5
4.0
4.5
5.0
5.5
6.0
6.5
7.0
7.5
f1 (ppm)

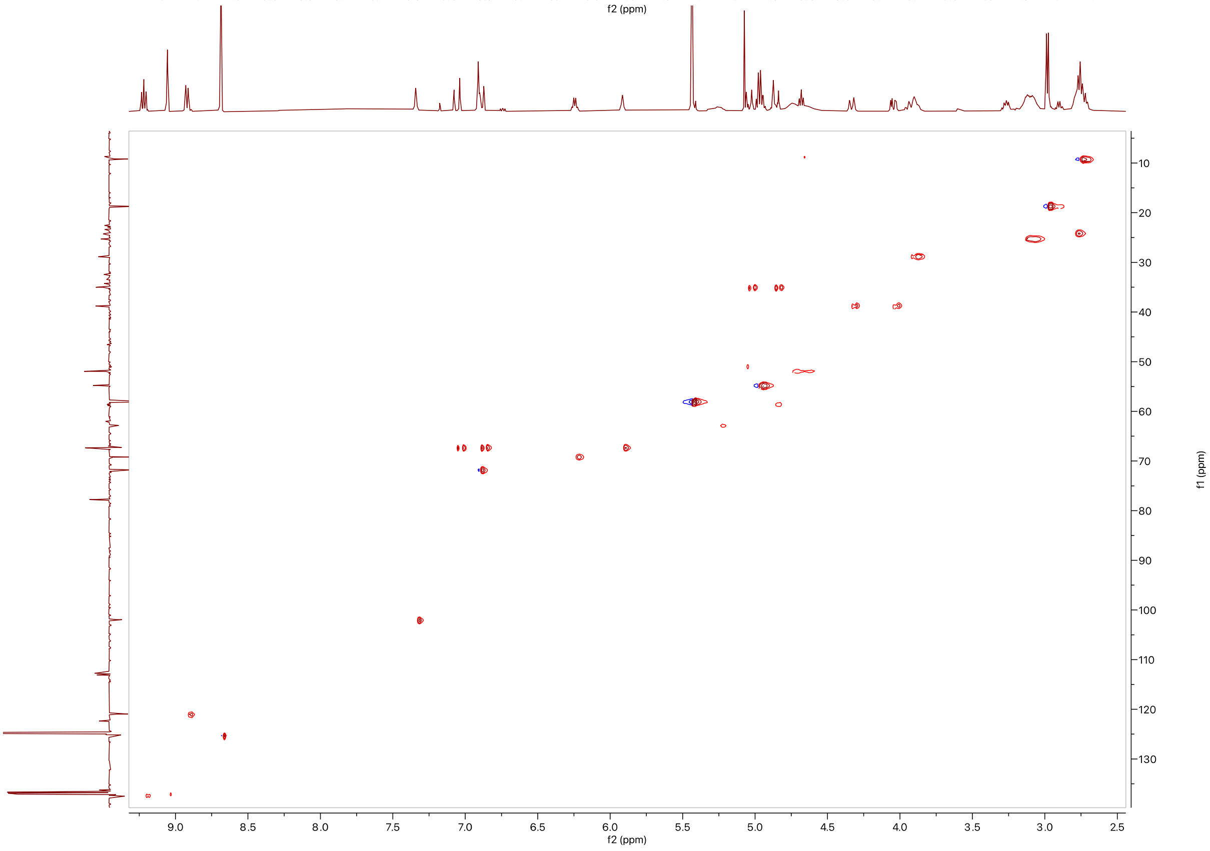
9.0
8.5
8.0
7.5
7.0
6.5
6.0
5.5
5.0
4.5
4.0
3.5
3.0
2.5
f2 (ppm)
10
20
30
40
50
60
70
80
90
100
110
120
130
f1 (ppm)

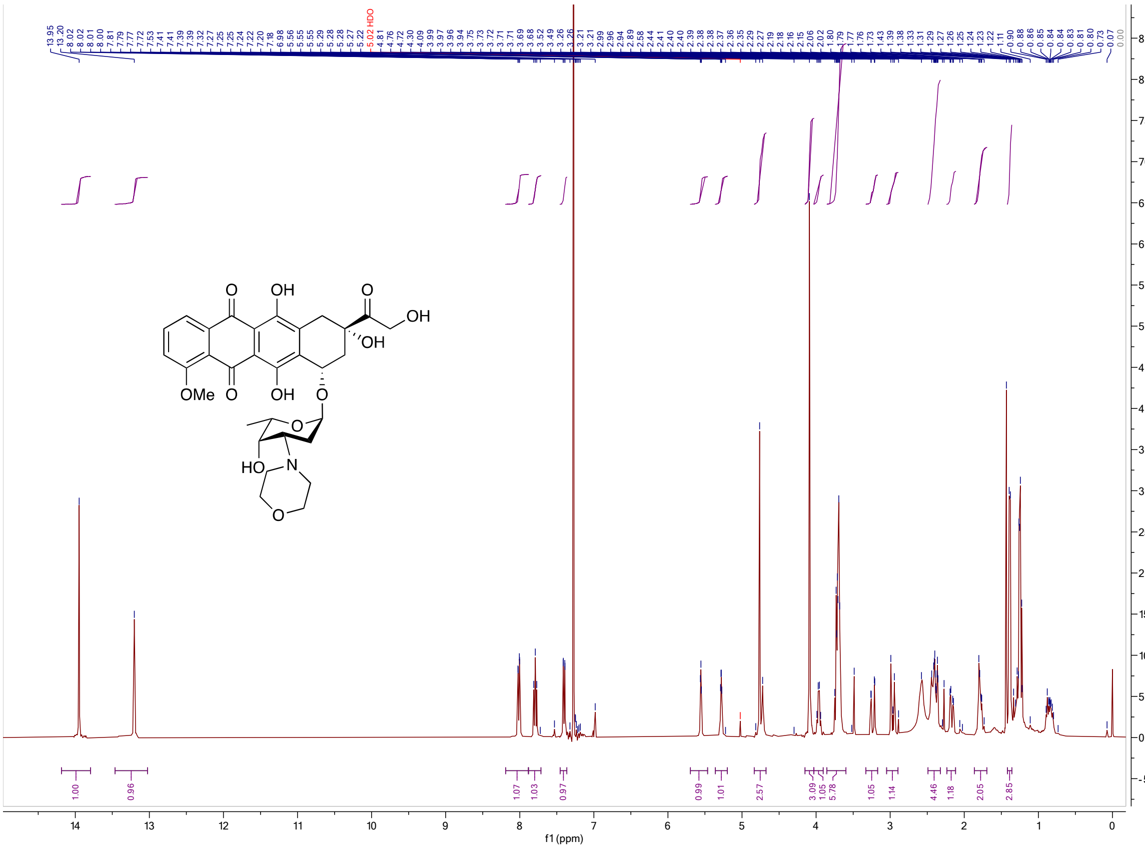
13.95 13.20 8.02 8.02 8.01 8.00 7.81 7.79 7.77 7.72 7.53 7.41 7.39 7.39 7.32 7.27 7.25 7.25 7.24 7.22 7.20 7.18 6.98 5.56 5.55 5.55 5.29 5.28 5.28 5.27 5.22 5.02 HDO 4.81 4.76 4.72 4.30 4.09 3.99 3.97 3.96 3.94 3.75 3.73 3.72 3.71 3.71 3.69 3.68 3.52 3.49 3.26 3.26 3.21 3.21 2.99 2.96 2.94 2.89 2.58 2.44 2.41 2.40 2.40 2.39 2.38 2.38 2.37 2.36 2.35 2.29 2.27 2.19 2.18 2.16 2.15 2.06 2.02 1.80 1.79 1.77 1.76 1.73 1.43 1.39 1.38 1.33 1.31 1.29 1.27 1.26 1.25 1.24 1.23 1.22 1.11 0.90 0.88 0.86 0.85 0.84 0.84 0.83 0.81 0.80 0.73 0.07 0.00
O OH O OH OH OMe O OH O O HO N O
1.00 0.96 1.07 1.03 0.97 0.99 1.01 2.57 3.09 1.05 5.78 1.05 1.14 4.46 1.18 2.05 2.85
f1 (ppm)

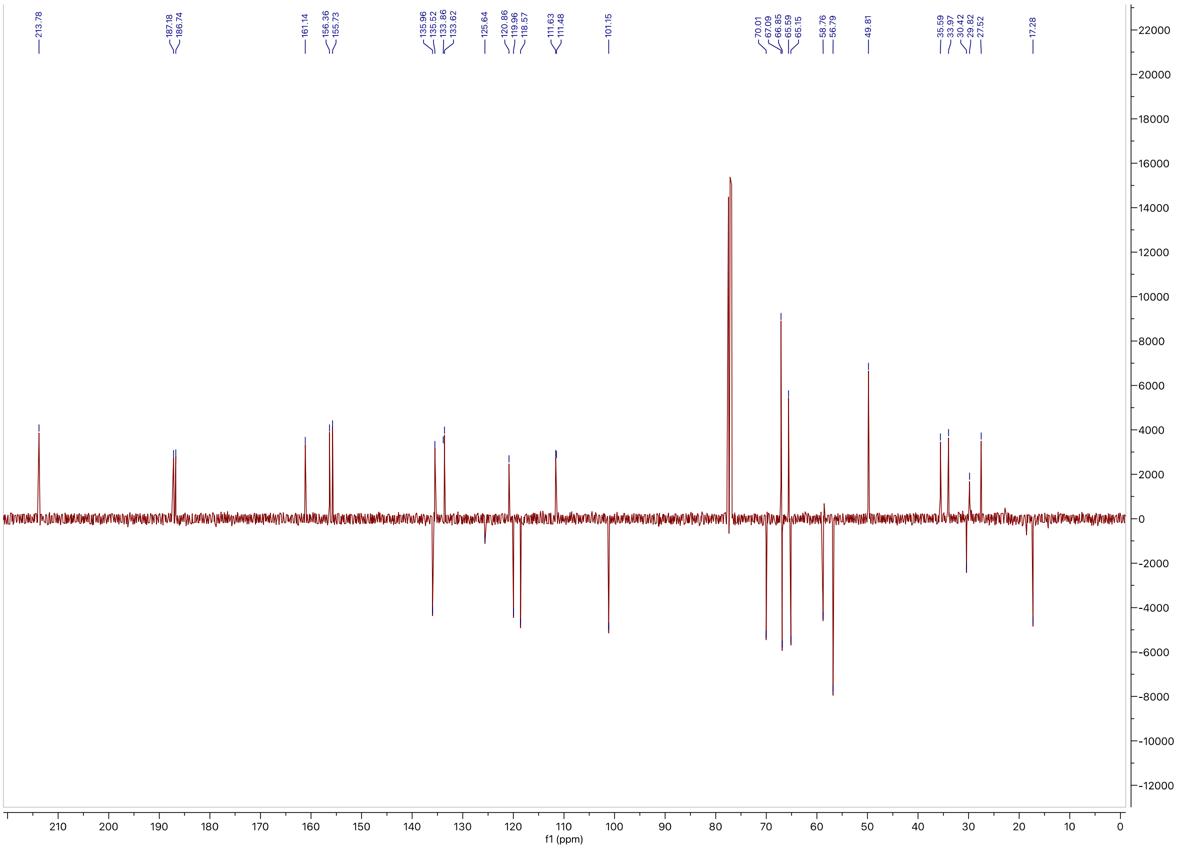
213.78 187.18 186.74 161.14 156.36 155.73 135.96 135.52 133.86 133.62 125.64 120.86 119.96 118.57 111.63 111.48 101.15 70.01 67.09 66.85 65.59 65.15 58.76 56.79 49.81 35.59 33.97 30.42 29.82 27.52 17.28
f1 (ppm)

7-[3-Azido-2,3-dideoxy-L-fucopyranoside]-14-O-tert-butyldimethylsilyl-doxorubicinone (10)

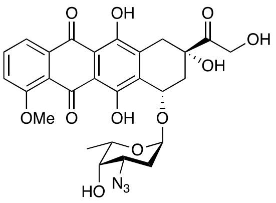

To a mixture of doxorubicin hydrochloride (200 mg, 0.340 mmol), potassium carbonate (72 mg, 0.51 mmol, 1.5 eq) and copper sulfate (cat. amount) in methanol (4 mL) was added imidazole-1-sulfonyl azide hydrochloride[6] (22 mg, 0.1035 mmol, 3.3 eq) and the reaction mixture was stirred overnight. The mixture diluted with water and extracted with DCM thrice. The combined organic layers dried over Na_2SO_4 and concentrated *in vacuo*. Column chromatography (10:90 MeOH:DCM) gave 3'-azidodoxorubicin as a red solid (140 mg, 0.248 mmol, 72%). Spectral data was in accordance with that of literary precedence.[7]

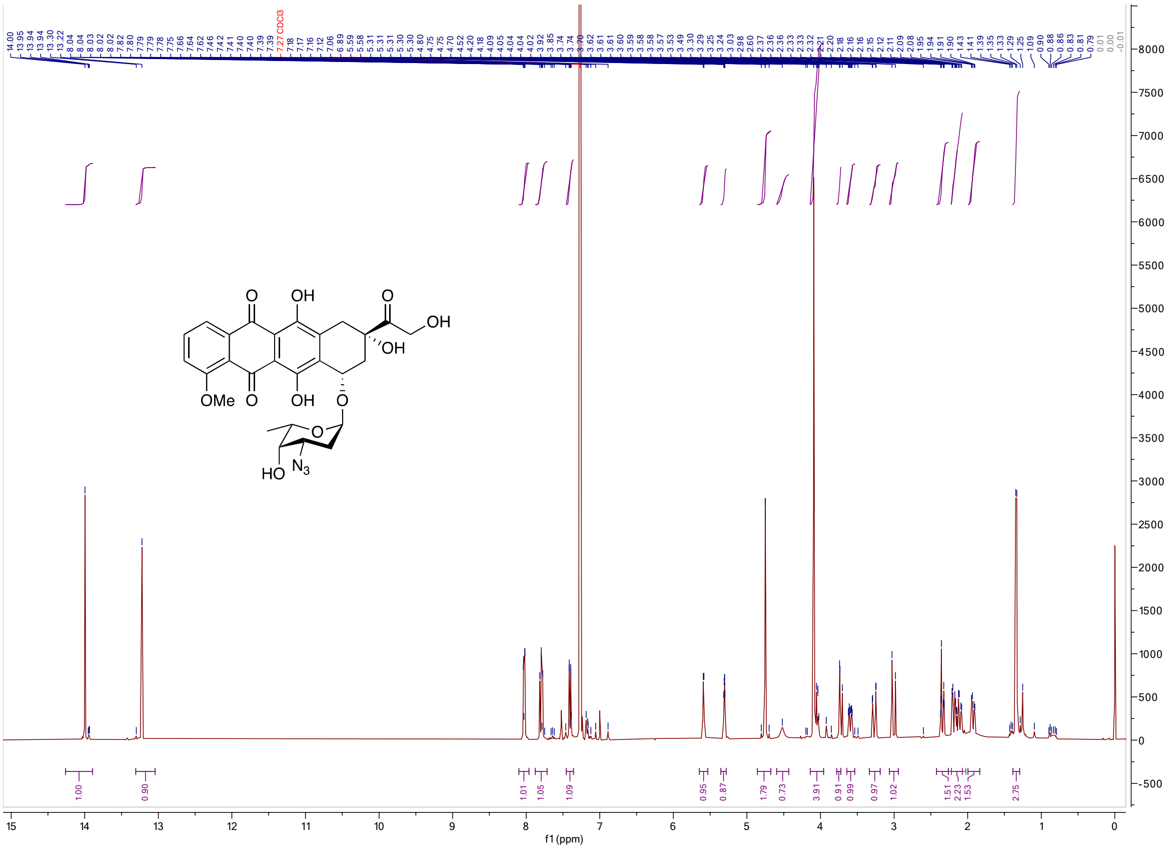
7.27 CDCl3
OH
O
OH
OMe
O
OH
O
OH
OH
O
O
N3
HO
1.00
0.90
1.01
1.05
1.09
0.95
0.87
1.79
0.73
3.91
0.91
0.99
0.97
1.02
1.51
2.23
1.53
2.75
f1 (ppm)

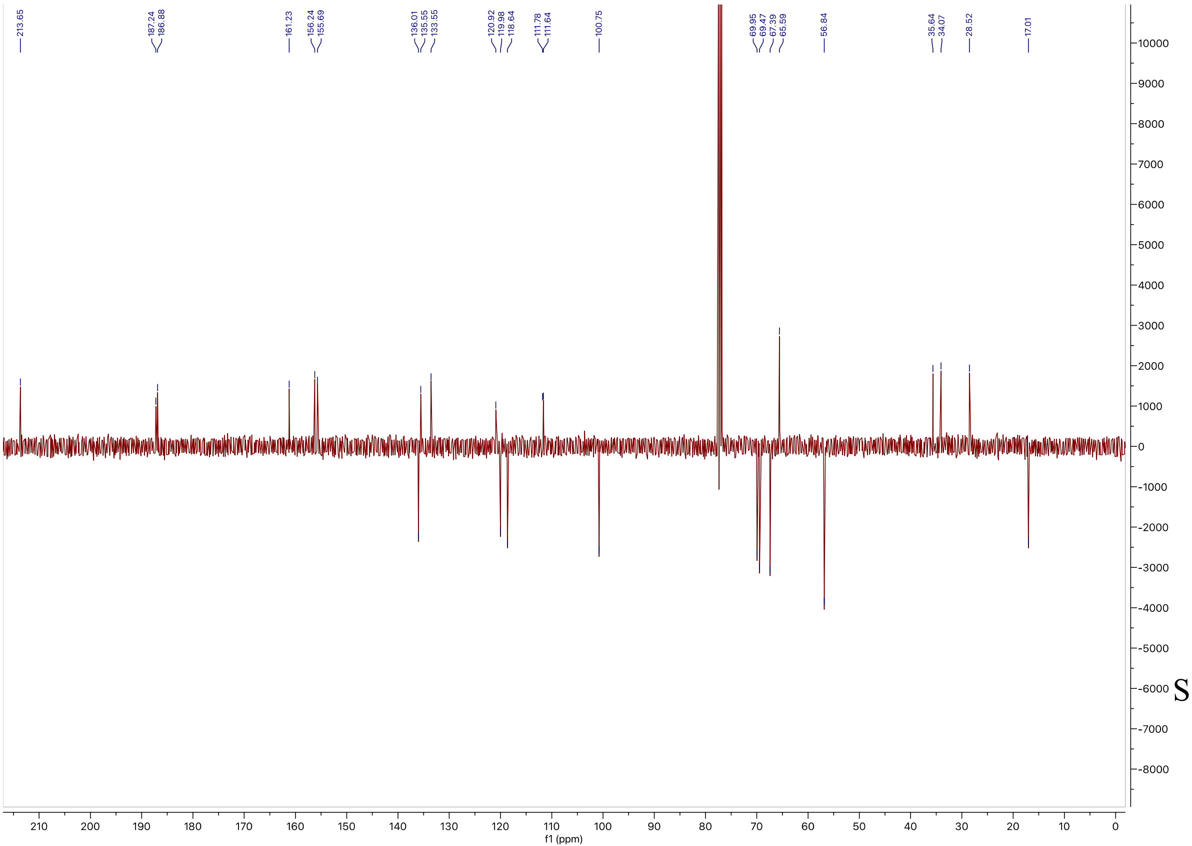
213.65
187.24
186.88
161.23
156.24
155.69
136.01
135.55
133.55
120.92
119.98
118.64
111.78
111.64
100.75
69.95
69.47
67.39
65.59
56.84
35.64
34.07
28.52
17.01
f1 (ppm)

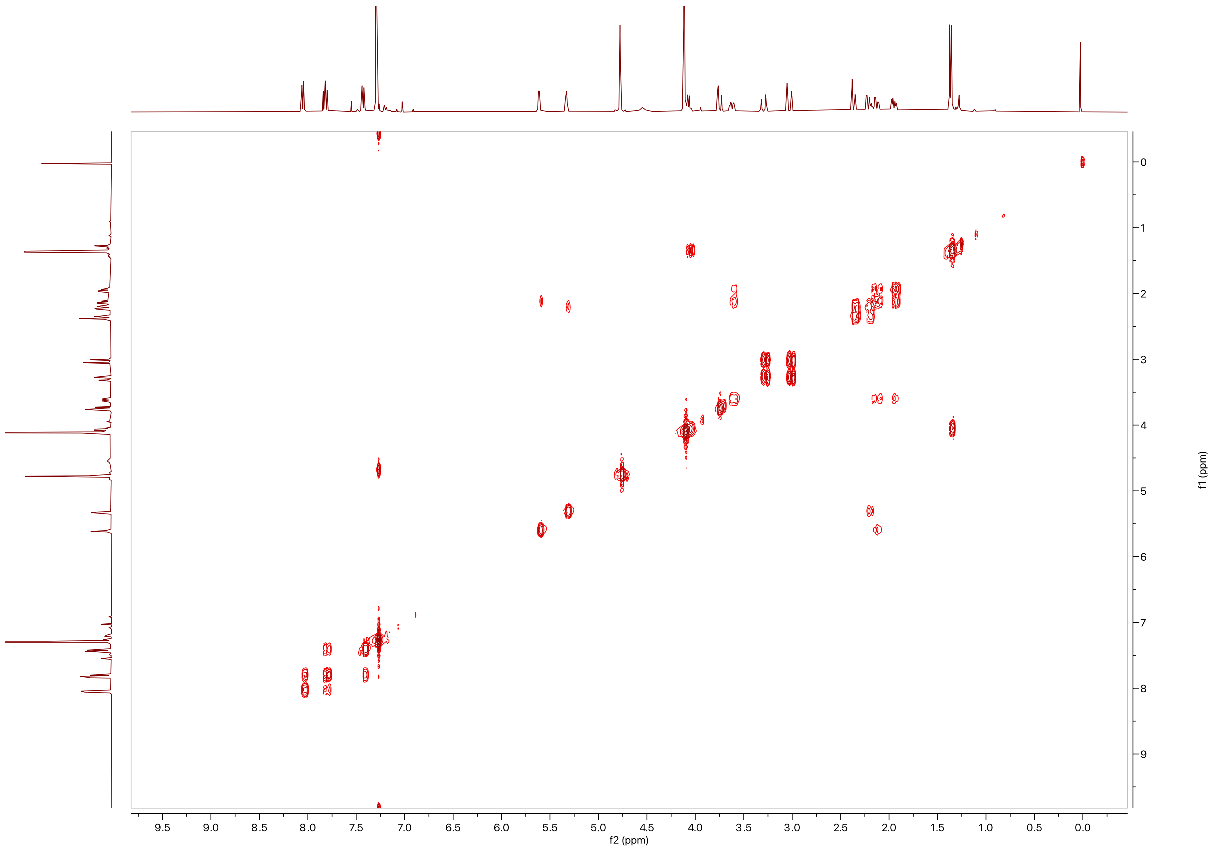
9.5 9.0 8.5 8.0 7.5 7.0 6.5 6.0 5.5 5.0 4.5 4.0 3.5 3.0 2.5 2.0 1.5 1.0 0.5 0.0
f2 (ppm)
0 1 2 3 4 5 6 7 8 9
f1 (ppm)

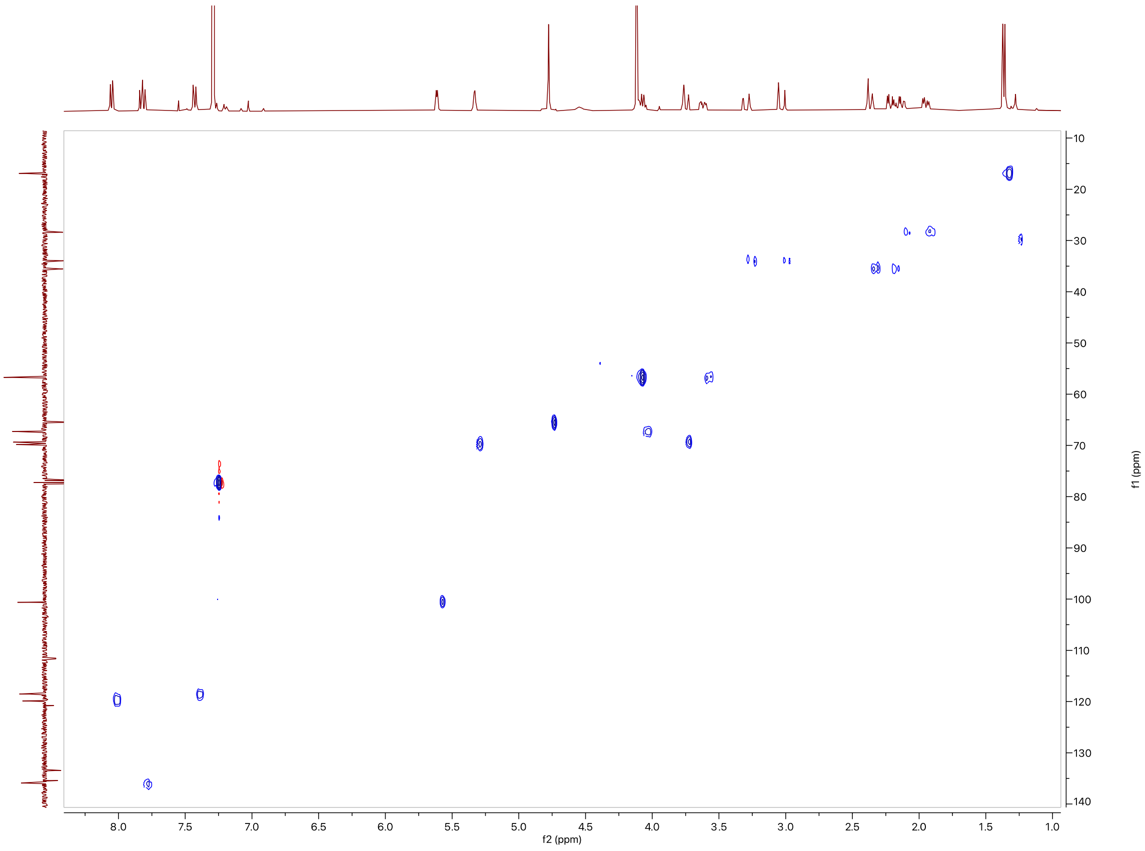
8.0 7.5 7.0 6.5 6.0 5.5 5.0 4.5 4.0 3.5 3.0 2.5 2.0 1.5 1.0
f2 (ppm)
10 20 30 40 50 60 70 80 90 100 110 120 130 140
f1 (ppm)

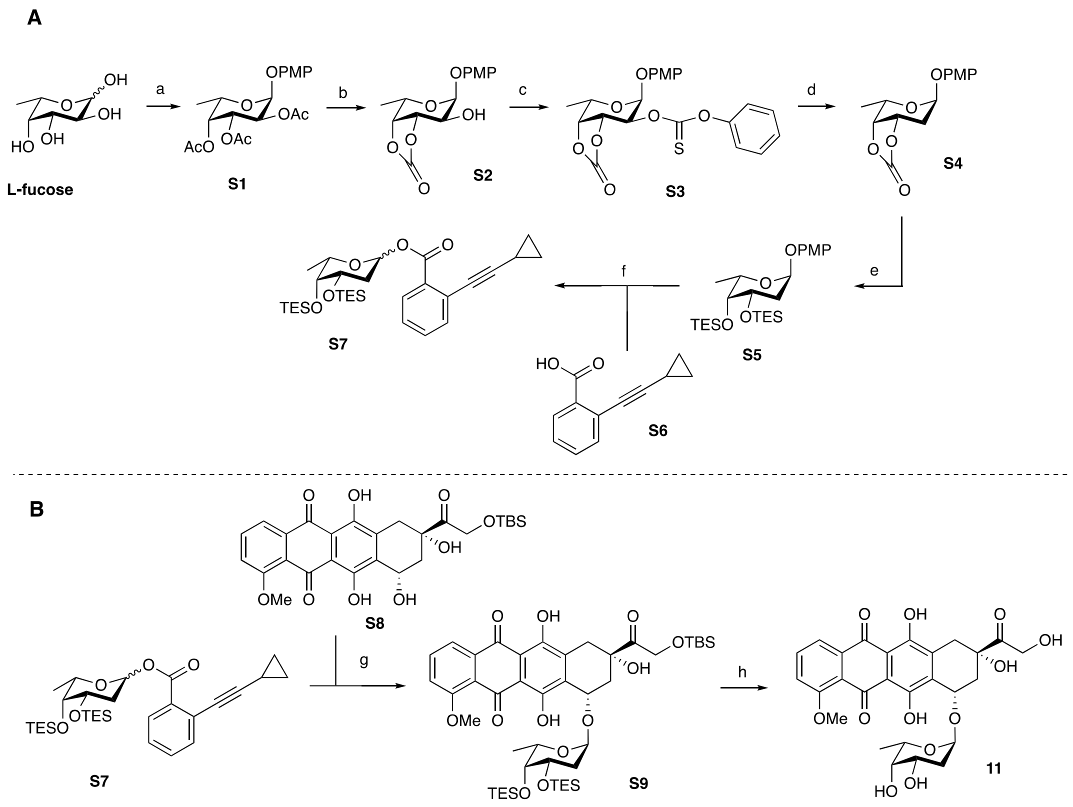

Scheme S1. Synthesis of 3'-desamino-3'-hydroxydoxorubicin (**11**). (A) Synthesis of 2-deoxyfucosyl alkynylbenzoate donor **S7**; (B) Glycosylation and deprotection. *Reagents and conditions:* (a) *i.* Ac_2O, pyr.; *ii.* *p*-methoxyphenol, $BF_3{\cdot}OEt_2$, DCM, 0 °C to RT, 76% over 2 steps; (b) *i.* NaOMe, MeOH; *ii.* carbonyldiimidazole, DMF, 85 °C, quant. over 2 steps; (c) *O*-phenyl thionochloroformate, pyr., DCM, 92%; (d) Bu_3SnH, AIBN, toluene, 80 °C, 85%; (e) *i.* NaOMe, MeOH; *ii.* triethylsilyl triflate, pyr., DMF, 64% over 2 steps; (f) *i.* Ag(II)(hydrogen dipicolinate)$_2$, NaOAc, ACN, H_2O, 0 °C; *ii.* EDCI·HCl, DIPEA, DMAP, DCM, 61% over 2 steps (1:9 α:β); (g) PPh_3AuNTf_2, DCM, 63% (α-only); (h) HF·pyridine, THF/pyr., 78%.

p-Methoxyphenyl-2,3,4-O-acetyl-α-L-fucopyranoside (S1)[8]

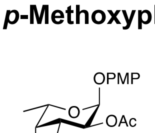

Commercially available L-fucose (6.53 g, 39.8 mmol) was suspended in pyridine (155 mL) and acetic anhydride (77 mL), to which DMAP (690 mg, 5.65 mmol, 0.14 eq) was added. After stirring overnight, the mixture was concentrated *in vacuo*. It was then partitioned between EtOAc and 1M HCl, and the organic layer was successively washed with sat. aq. $NaHCO_3$ and brine, dried over $MgSO_4$ and concentrated *in vacuo* to give the crude peracetylated fucose. This was then together with *p*-methoxyphenol (7.41 g, 59.7 mmol, 1.5 eq) coevaporated from toluene and dissolved in DCM (320 mL). Then at 0°C, $BF_3{\cdot}OEt_2$ (8.42 mL, 79.6 mmol, 2 eq) was added and the mixture was allowed to warm up to RT overnight. It was then poured into sat. aq. $NaHCO_3$, and the organic layer was washed with 1M NaOH, dried over $MgSO_4$ and concentrated *in vacuo*. Column chromatography (9:1:1 pentane:EtOAc:DCM) gave the title compound as a colourless syrup (12.5g, 31.6 mmol, 79% over 3 steps). Spectral data was in accordance with that of literary precedence.[8]

***p*-Methoxyphenyl-3,4-O-carbonate-α-L-fucopyranoside (S2)**

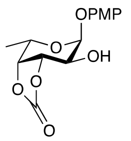

To a solution of **S1** (10.38 g, 26.2 mmol) in MeOH (180 mL) was added NaOMe until pH>10 and the mixture was stirred for 3.5 hours. It was then neutralized by addition of AcOH and concentrated *in vacuo* to yield the corresponding triol. This crude triol was then dissolved in DMF (100 mL) and added dropwise to a solution of carbonyl diimidazole (4.25 g, 26.2 mmol, 1 eq) in DMF (130 mL) by syringe pump over 1 hour at 85 ^{o}C. Thereafter, 1M HCl (200 mL) was added and the mixture was stirred for a further 15 minutes at the same temperature. It was then diluted with EtOAc and washed with H_2O thrice and sat. aq. $NaHCO_3$. Drying over $MgSO_4$ and concentration *in vacuo* gave the title compound as a white solid (7.76 g, 26.2 mmol, quant. over 2 steps). ^{1}H NMR (400 MHz, Chloroform-*d*) δ 7.09 – 6.91 (m, 2H), 6.91 – 6.79 (m, 2H), 5.40 (d, *J* = 4.0 Hz, 1H), 4.97 (dd, *J* = 7.5, 5.7 Hz, 1H), 4.68 (dd, *J* = 7.6, 2.1 Hz, 1H), 4.37 (qd, *J* = 6.6, 2.1 Hz, 1H), 4.18 (dt, *J* = 6.1, 3.0 Hz, 1H), 3.77 (s, 3H), 3.49 (d, *J* = 4.0 Hz, 1H), 1.32 (d, *J* = 6.6 Hz, 3H). ^{13}C NMR (101 MHz, $CDCl_3$) δ 155.6, 154.3, 150.1, 118.2, 114.8, 95.9, 77.0, 76.0, 66.6, 63.8, 55.7, 15.6. HRMS: $(M + Na)^+$ calculated for $C_{14}H_{16}O_7Na$ 319.0794; found 319.0788.

***p*-Methoxyphenyl-3,4-O-carbonate-2-*O*-(phenoxy)thiocarbonyl-α-L-fucopyranoside (S3)**

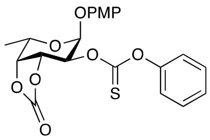

To a solution of **S2** (6.70 g, 22.6 mmol) in DCM/pyr (220 mL, 1:1 v/v), after which *O*-phenyl chlorothionoformate (4.84 mL, 1.55 eq) was added at 0°C. After stirring overnight, MeOH (6 mL) was added to quench and the mixture was concentrated *in vacuo*. The residue was partitioned between EtOAc and H_2O, then the organic layer was dried over $MgSO_4$ and concentrated *in vacuo*. Column chromatography (5-20% EtOAc in pentane) gave the title compound as an orange foam (9.00g, 20.8 mmol, 92%). ^{1}H NMR (400 MHz, Chloroform-*d*) δ 7.47 – 7.36 (m, 2H), 7.36 – 7.29 (m, 1H), 7.16 – 7.08 (m, 2H), 7.09 – 6.95 (m, 2H), 6.91 – 6.80 (m, 2H), 5.85 (d, *J* = 3.7 Hz, 1H), 5.63 (dd, *J* = 7.6, 3.7 Hz, 1H), 5.24 (dd, *J* = 7.6, 6.9 Hz, 1H), 4.79 (dd, *J* = 6.9, 2.6 Hz, 1H), 4.33 (qd, *J* = 6.7, 2.6 Hz, 1H), 3.79 (s, 3H), 1.44 (d, *J* = 6.7 Hz, 3H). ^{13}C NMR (101 MHz, $CDCl_3$) δ 194.1, 155.8, 153.5, 153.4, 150.2, 129.8, 127.1, 121.8, 117.7, 114.9, 93.8, 78.0, 77.7, 74.5, 63.2, 55.8, 15.8. HRMS: $(M + Na)^+$ calculated for $C_{21}H_{20}O_8SNa$ 455.0777; found 455.0778.

***p*-Methoxyphenyl-2-deoxy-3,4-*O*-carbonate-α-L-fucopyranoside (S4)**

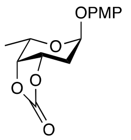

A solution of **S3** (2.12 g, 4.90 mmol), tributyltin hydride (3.95 mL, 14.7 mmol, 3 eq) and azobisisobutyronitrile (0.2M in toluene, 0.98 mmol, 0.2 eq) in toluene (160 mL) was heated at 100°C for 10 minutes. It was then allowed to cool to room temperature, washed with 1M NaOH, dried over $MgSO_4$ and concentrated *in vacuo*. Column chromatography (20:80 – 40:60 Et_2O:pentane) gave the title compound as a colourless oil (1.16 g, 4.14 mmol, 85%). ^{1}H NMR (400 MHz, Chloroform-*d*) δ 7.06 – 6.89 (m, 2H), 6.89 – 6.74 (m, 2H), 5.53 (t, *J* = 6.3 Hz, 1H), 5.08 (dt, *J* = 8.3, 3.6 Hz, 1H), 4.60 (dd, *J* = 8.4, 1.8 Hz, 1H), 4.18 (qd, *J* = 6.6, 1.8 Hz, 1H), 3.77 (s, 3H), 2.62 (ddd, *J* = 15.7, 5.9, 4.0 Hz, 1H), 2.11 (ddd, *J* = 15.8, 6.8, 3.4 Hz, 1H), 1.32 (d, *J* = 6.5 Hz, 3H). ^{13}C NMR (101 MHz, $CDCl_3$) δ 155.3, 154.4, 150.7, 118.3, 114.7, 95.0, 76.0, 72.5, 64.1, 55.8, 29.1, 15.5. HRMS: $(M + Na)^+$ calculated for $C_{14}H_{16}O_6Na$ 303.0845; found 303.0847.

***p*-Methoxyphenyl-2-deoxy-3,4-*O*-triethylsilyl -α-L-fucopyranoside (S5)**

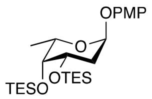

To a solution of **S4** (420 mg, 1.5 mmol) in MeOH (3.8 mL) was added NaOMe (16 mg, 0.30 mmol, 0.2 eq) and the mixture was allowed to stir overnight. It was quenched by addition of dry ice and concentrated *in vacuo* to yield the corresponding diol. This was then dissolved in DMF (7.6 mL), to which pyridine (0.70 mL, 8.7 mmol, 5.8 eq) and triethylsilyl triflate (1.2 mL, 5.1 mmol, 3.4 eq) were added at 0°C. The resulting mixture was allowed to stir overnight, after which another portion of both reagents was added at 0°C, and the mixture was allowed to stir overnight once again. It was then poured into EtOAc, washed with H_2O 5x, dried over $MgSO_4$ and concentrated *in vacuo*. Column chromatography (100:1 pentane:Et_3N – 90:10:1 pentane:Et_2O:Et_3N) gave the title compound as a clear oil (460 mg, 0.95 mmol, 64% over 2 steps). ^{1}H NMR (400 MHz, Chloroform-*d*) δ 7.06 – 6.90 (m, 2H), 6.90 – 6.72 (m, 2H), 5.50 (d, *J* = 3.2 Hz, 1H), 4.18 (ddd, *J* = 11.7, 4.5, 2.5 Hz, 1H), 3.92 (q, *J* = 6.5 Hz, 1H), 3.77 (s, 3H), 3.64 (d, *J* = 2.5 Hz, 1H), 2.20 (td, *J* = 12.3, 3.6 Hz, 1H), 1.78 (ddt, *J* = 12.7, 4.6, 1.3 Hz, 1H), 1.15 (d, *J* = 6.5 Hz, 3H), 0.99 (dt, *J* = 8.9, 8.0 Hz, 18H), 0.78 – 0.44 (m, 12H). ^{13}C NMR (101 MHz, $CDCl_3$) δ 154.8, 151.7, 118.0, 114.9, 97.8, 73.9, 68.7, 67.9, 56.1, 33.6, 17.8, 7.5, 7.3, 5.7, 5.3. HRMS: $(M + Na)^+$ calculated for $C_{25}H_{46}O_5Si_2Na$ 505.2782; found 505.2777.

***o*-Cyclopropylethynylbenzoyl-2-deoxy-3,4-*O*-triethylsilyl-L-fucopyranoside (S7)**

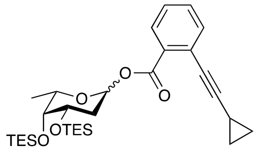

To a solution of **S5** (450 mg, 0.93 mmol) in MeCN:H_2O (50 mL, 1:1 v/v) were added NaOAc (808 mg, 9.3 mmol, 10 eq) and then $Ag(DPAH)_2 \cdot H_2O$ (1.76 g, 3.72 mmol, 4 eq) portionwise over 30 minutes at 0°C. The mixture was stirred for 3.5 hours; after which it was poured into sat. aq. $NaHCO_3$. This was then extracted with DCM thrice, dried over $MgSO_4$ and concentrated *in vacuo* to give the crude lactol. To a solution of this in DCM were added DIPEA (0.75 mL, 4.2 mmol, 4.5 eq), DMAP (119 mg, 0.93 mmol, 1 eq), EDCI·HCl (581 mg, 2.93 mmol, 3.2 eq) and freshly saponified *o*-cyclopropylethynylbenzoic acid **S6** (559 mg, 2.79 mmol, 3 eq). After stirring overnight, the mixture was diluted with DCM and washed with sat. aq. $NaHCO_3$ and brine. Drying over $MgSO_4$, concentration *in vacuo* and column chromatography of the residue (2:98 – 4:96 EtOAc:pentane) followed by size-exclusion chromatography (Sephadex LH-20, 1:1 DCM:MeOH v/v) gave the title compound as a white solid (312 mg, 0.57 mmol, 1:9 α:β, 62% over 2 steps). Spectral data for the β-anomer: ^{1}H NMR (400 MHz, Chloroform-*d*) δ 7.99 (dd, *J* = 7.9, 1.4 Hz, 1H), 7.54 – 7.34 (m, 2H), 7.29 (qd, *J* = 7.3, 1.4 Hz, 1H), 5.90 (dd, *J* = 10.2, 2.3 Hz, 1H), 3.77 (ddd, *J* = 11.9, 4.3, 2.6 Hz, 1H), 3.65 – 3.55 (m, 2H), 2.20 (td, *J* = 11.8, 10.1 Hz, 1H), 1.82 (dddd, *J* = 11.6, 4.3, 2.3, 1.0 Hz, 1H), 1.55 – 1.47 (m, 1H), 1.28 (d, *J* = 6.3 Hz, 3H), 0.98 (tt, *J* = 7.5, 3.8 Hz, 18H), 0.92 – 0.85 (m, 4H), 0.76 – 0.54 (m, 12H). ^{13}C NMR (101 MHz, $CDCl_3$) δ 164.7, 134.2, 132.0, 131.0, 131.0, 127.0, 125.1, 99.8, 93.2, 74.7, 72.7, 72.5, 70.8, 33.8, 17.3, 9.0, 7.2, 6.9, 5.3, 4.9, 0.8. HRMS: $(M + Na)^+$ calculated for $C_{30}H_{48}O_5Si_2Na$ 567.2938; found 567.2946.

7-[2-Deoxy-3,4-*O*-triethylsilyl-α-L-fucopyranoside]-14-*O*-*tert*-butyldimethylsilyl-doxorubicinone (S9)

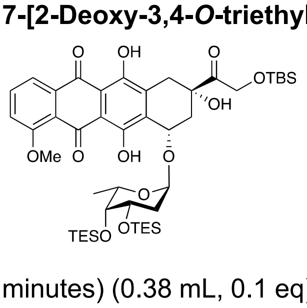

To a solution of glycosyl donor **S7** (207 mg, 0.38 mmol) and the glycosyl acceptor **S8** (301 mg, 0.57 mmol, 1.5 eq) in DCM (7.6 mL), activated molecular sieves (4Å) were added. The mixture was stirred for 30 minutes at room temperature andthen a freshly prepared 0.1M DCM solution of PPh_3AuNTf_2 (prepared by stirring 1:1 PPh_3AuCl and $AgNTf_2$ in DCM for 30 minutes) (0.38 mL, 0.1 eq) in DCM was added dropwise. After 15 minutes, the mixture was filtered and concentrated *in vacuo*. Column chromatography (20:80 Et_2O:pentane and then 1:99 – 2:98 acetone:toluene) of the residue gave the title compound as a red solid (211 mg, 0.24 mmol, 63%). ^{1}H

NMR (400 MHz, Chloroform-*d*) δ 13.83 (s, 1H), 13.12 (s, 1H), 7.93 (dd, *J* = 7.7, 1.1 Hz, 1H), 7.73 (t, *J* = 8.1 Hz, 1H), 7.37 (dd, *J* = 8.6, 1.1 Hz, 1H), 5.50 (d, *J* = 3.8 Hz, 1H), 5.23 (dd, *J* = 4.0, 2.2 Hz, 1H), 5.01 – 4.83 (m, 2H), 4.79 (s, 1H), 4.08 (s, 3H), 3.91 (q, *J* = 6.4 Hz, 1H), 3.77 (ddd, *J* = 12.0, 4.6, 2.4 Hz, 1H), 3.69 – 3.56 (m, 1H), 3.11 (dd, *J* = 19.0, 1.9 Hz, 1H), 2.86 (d, *J* = 18.8 Hz, 1H), 2.32 (dt, *J* = 14.8, 2.1 Hz, 1H), 2.15 – 2.05 (m, 2H), 1.58 (dd, *J* = 12.9, 4.5 Hz, 1H), 1.26 (d, *J* = 6.3 Hz, 4H), 1.04 – 0.92 (m, 18H), 0.87 (t, *J* = 7.9 Hz, 9H), 0.67 (qd, *J* = 8.3, 7.9, 3.7 Hz, 6H), 0.53 (qd, *J* = 8.3, 7.9, 1.9 Hz, 6H), 0.15 (d, *J* = 2.7 Hz, 6H). ^{13}C NMR (101 MHz, $CDCl_3$) δ 211.5, 186.8, 186.5, 161.0, 156.4, 155.7, 135.6, 135.4, 134.1, 120.8, 119.7, 118.4, 111.2, 101.6, 73.4, 69.0, 68.9, 67.5, 66.7, 56.7, 35.4, 34.0, 32.9, 26.0, 18.7, 17.5, 7.1, 6.8, 5.3, 4.8, -5.2. HRMS: $(M + Na)^+$ calculated for $C_{45}H_{70}O_{12}Si_3Na$ 909.4073; found 909.4107.

7-[2-Deoxy-α-L-fucopyranoside]-doxorubicinone (11)

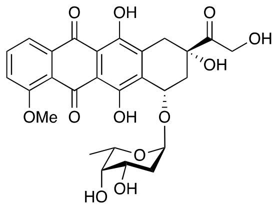

S9 (105 mg, 0.118 mmol) was dissolved in THF:pyr (12.3 mL, 2:1 v/v), to which HF·pyr complex (743 μL) was added at 0°C. After stirring for 3 hours, the same amount of HF·pyr complex was added and the mixture was stirred a further 1.5 hours. It was then poured into sat. aq. $NaHCO_3$, extracted with DCM twice, dried over Na_2SO_4 and concentrated *in vacuo*. Column chromatography on neutral silica (33:66 – 50:50 acetone:toluene) gave a solid, which was triturated with $CHCl_3$ and filtered. Evaporation of the filtrate gave the title compound as a red solid (50 mg, 92 μmol, 78%). Analytical data were in agreement with literature precedence.[9] ^{1}H NMR (400 MHz, Pyridine-d_5) δ 8.08 (d, *J* = 7.6 Hz, 1H), 7.82 (t, *J* = 8.1 Hz, 1H), 7.51 (d, *J* = 8.5 Hz, 1H), 5.85 (d, *J* = 3.8 Hz, 1H), 5.50 – 5.37 (m, 3H), 4.68 (q, *J* = 6.4 Hz, 1H), 4.52 (ddd, *J* = 12.1, 4.9, 2.9 Hz, 1H), 4.06 (s, 4H), 3.53 (q, *J* = 18.4 Hz, 2H), 2.92 – 2.83 (m, 1H), 2.65 (td, *J* = 12.5, 4.0 Hz, 1H), 2.54 (dd, *J* = 14.4, 5.1 Hz, 1H), 2.38 (dd, *J* = 12.7, 4.9 Hz, 1H), 1.58 (d, *J* = 6.5 Hz, 3H). ^{13}C NMR (101 MHz, Pyr) δ 215.5, 187.8, 162.1, 136.9, 135.2, 124.7, 121.7, 120.2, 112.5, 112.1, 103.2, 77.3, 72.5, 71.4, 68.8, 67.0, 66.1, 57.4, 38.0, 34.5, 34.2, 18.2. HRMS: $(M + H)^+$ calculated for $C_{27}H_{29}O_{12}$ 545.1659; found 545.2017.

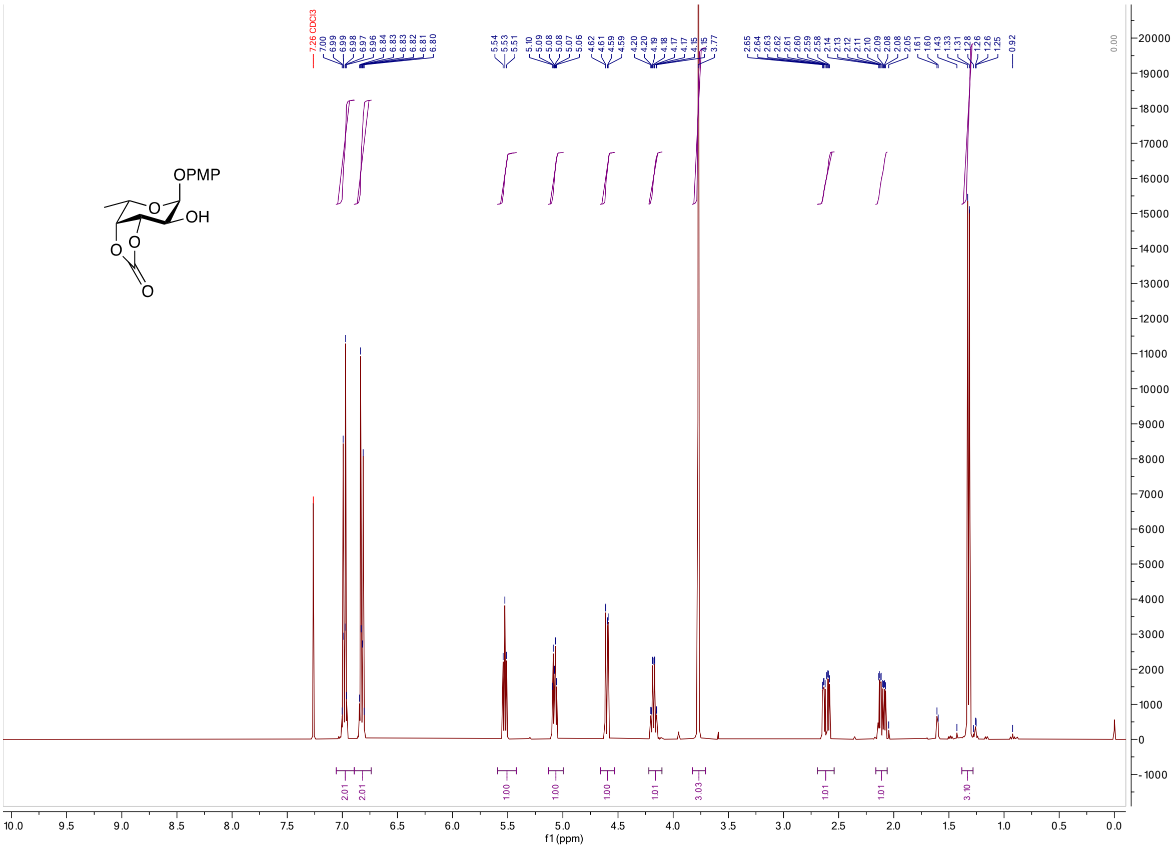
7.26 CDCl3
7.00 6.99 6.99 6.98 6.97 6.86 6.84 6.83 6.83 6.82 6.81 6.80
5.54 5.53 5.51
5.10 5.09 5.08 5.08 5.07 5.06
4.62 4.61 4.59 4.59
4.20 4.20 4.19 4.18 4.17 4.17 4.15 4.15
3.77
2.65 2.64 2.63 2.62 2.61 2.60 2.59 2.58
2.14 2.13 2.12 2.11 2.10 2.09 2.08 2.08 2.05
1.61 1.60 1.43 1.33 1.31 1.28 1.26 1.25
0.92
0.00
OPMP
O
OH
O
O
O
2.01 2.01 1.00 1.00 1.00 1.01 3.03 1.01 1.01 3.10
f1 (ppm)

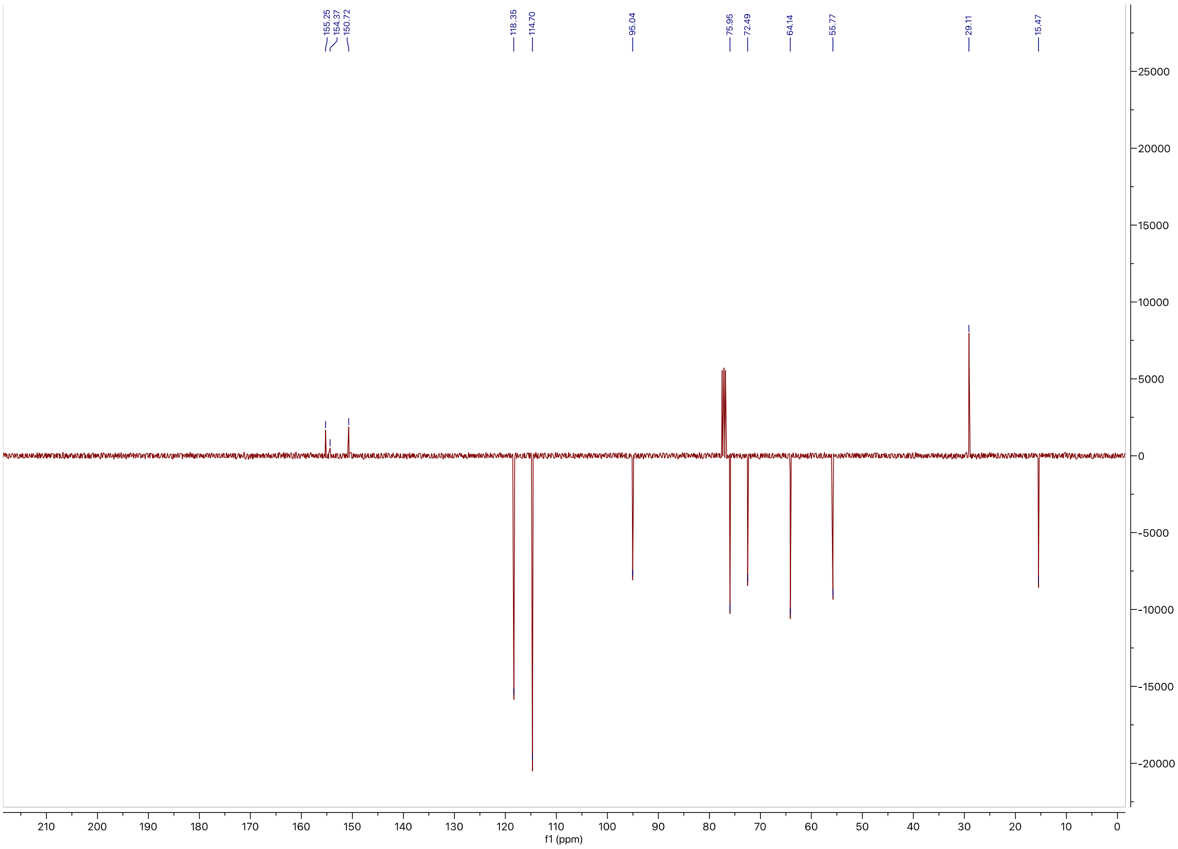
155.25
154.37
150.72
118.35
114.70
95.04
75.95
72.49
64.14
55.77
29.11
15.47
f1 (ppm)

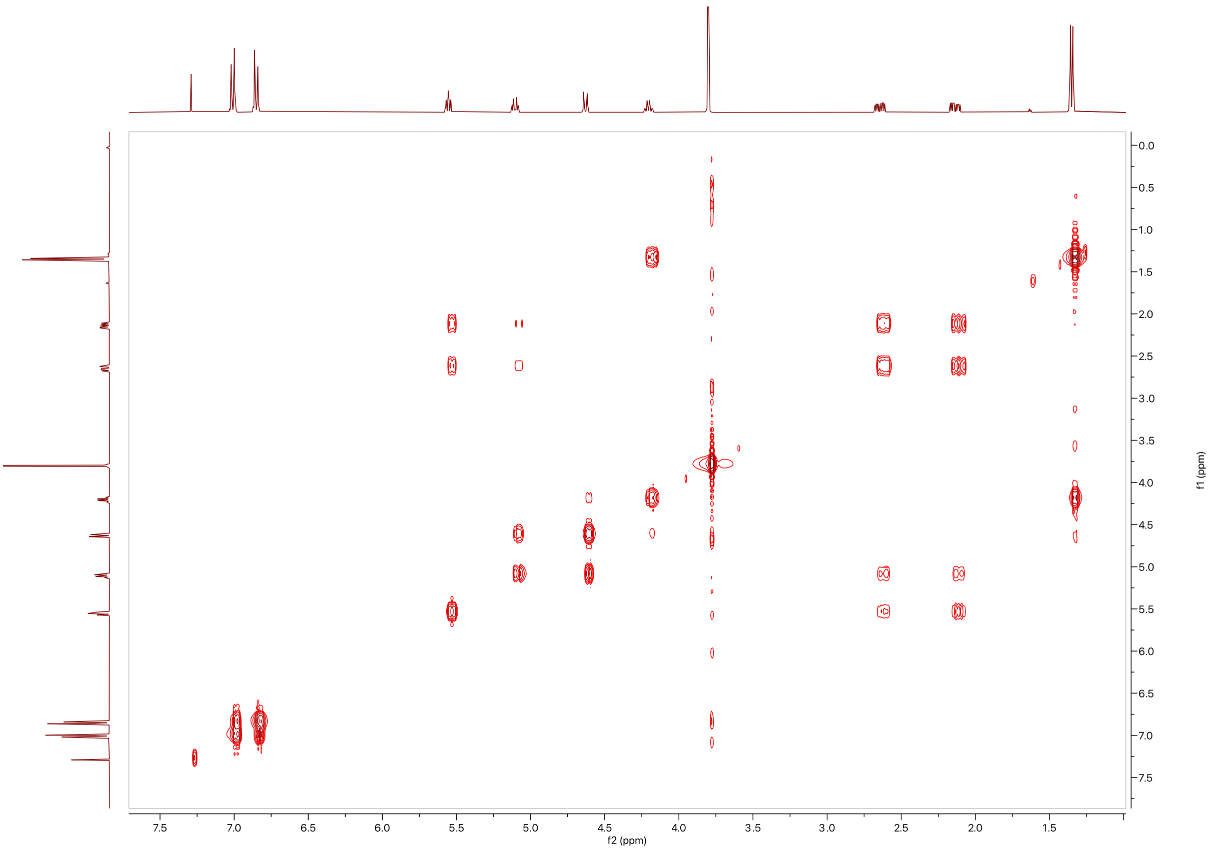

7.5 7.0 6.5 6.0 5.5 5.0 4.5 4.0 3.5 3.0 2.5 2.0 1.5
f2 (ppm)
0.0 0.5 1.0 1.5 2.0 2.5 3.0 3.5 4.0 4.5 5.0 5.5 6.0 6.5 7.0 7.5
f1 (ppm)

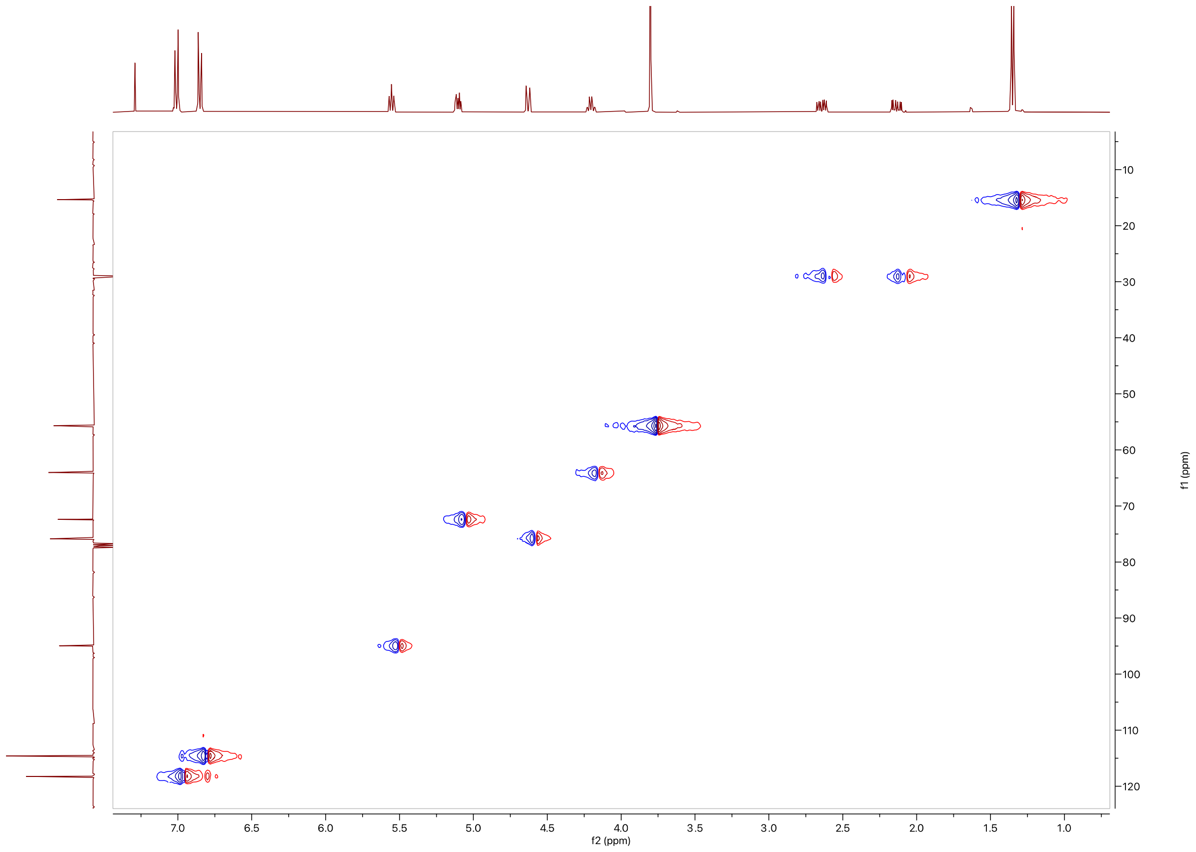

7.0 6.5 6.0 5.5 5.0 4.5 4.0 3.5 3.0 2.5 2.0 1.5 1.0
f2 (ppm)
10 20 30 40 50 60 70 80 90 100 110 120
f1 (ppm)

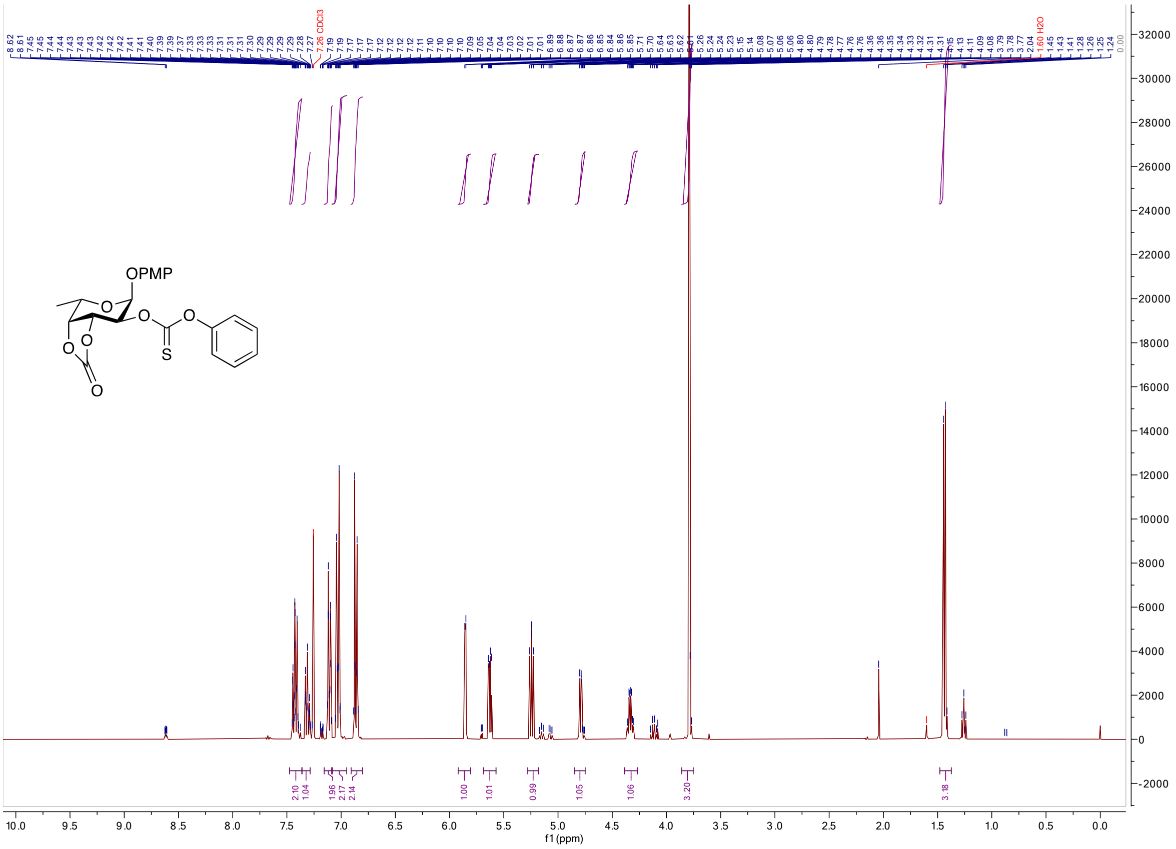

OPMP
O
O
O
O
O
S
O
8.62 8.61 7.45 7.44 7.44 7.43 7.43 7.42 7.42 7.41 7.41 7.40 7.39 7.39 7.37 7.33 7.33 7.31 7.31 7.30 7.29 7.29 7.29 7.28 7.27
7.26 CDCl3
7.19 7.19 7.17 7.17 7.17 7.12 7.12 7.12 7.11 7.10 7.10 7.10 7.10 7.09 7.05 7.04 7.04 7.03 7.02 7.01 7.01 6.89 6.88 6.87 6.87 6.86 6.85 6.84 5.86 5.85 5.71 5.70 5.64 5.63 5.62 5.61 5.26 5.24 5.24 5.23 5.15 5.14 5.08 5.07 5.06 5.06 4.80 4.80 4.79 4.78 4.77 4.76 4.76 4.36 4.36 4.35 4.34 4.33 4.32 4.31 4.15 4.13 4.11 4.09 4.08 3.79 3.78 3.77 2.04
1.60 H2O
1.45 1.43 1.41 1.28 1.26 1.25 1.24 0.00
2.10 1.04 1.96 2.17 2.14 1.00 1.01 0.99 1.05 1.06 3.20 3.18
f1 (ppm)

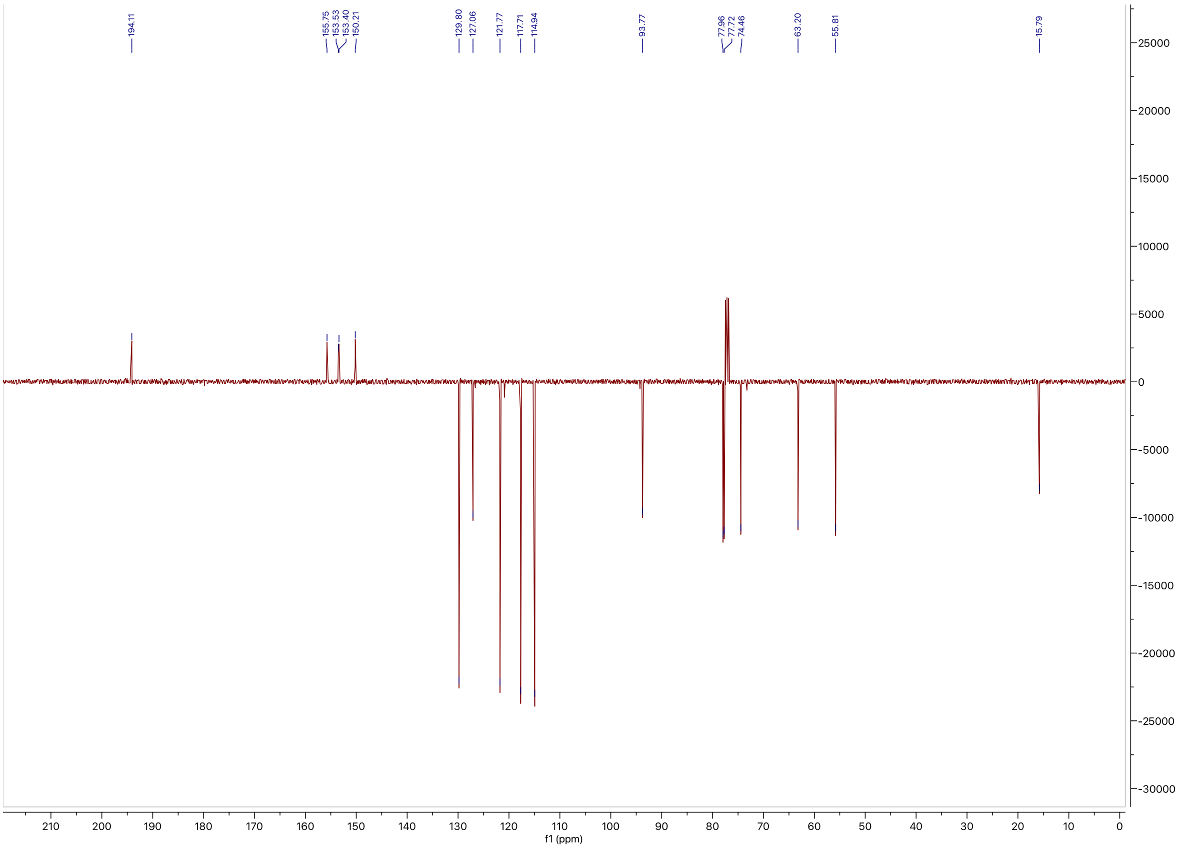

194.11
155.75 153.53 153.40 150.21
129.80 127.06 121.77 117.71 114.94
93.77
77.96 77.72 74.46
63.20 55.81
15.79
f1 (ppm)

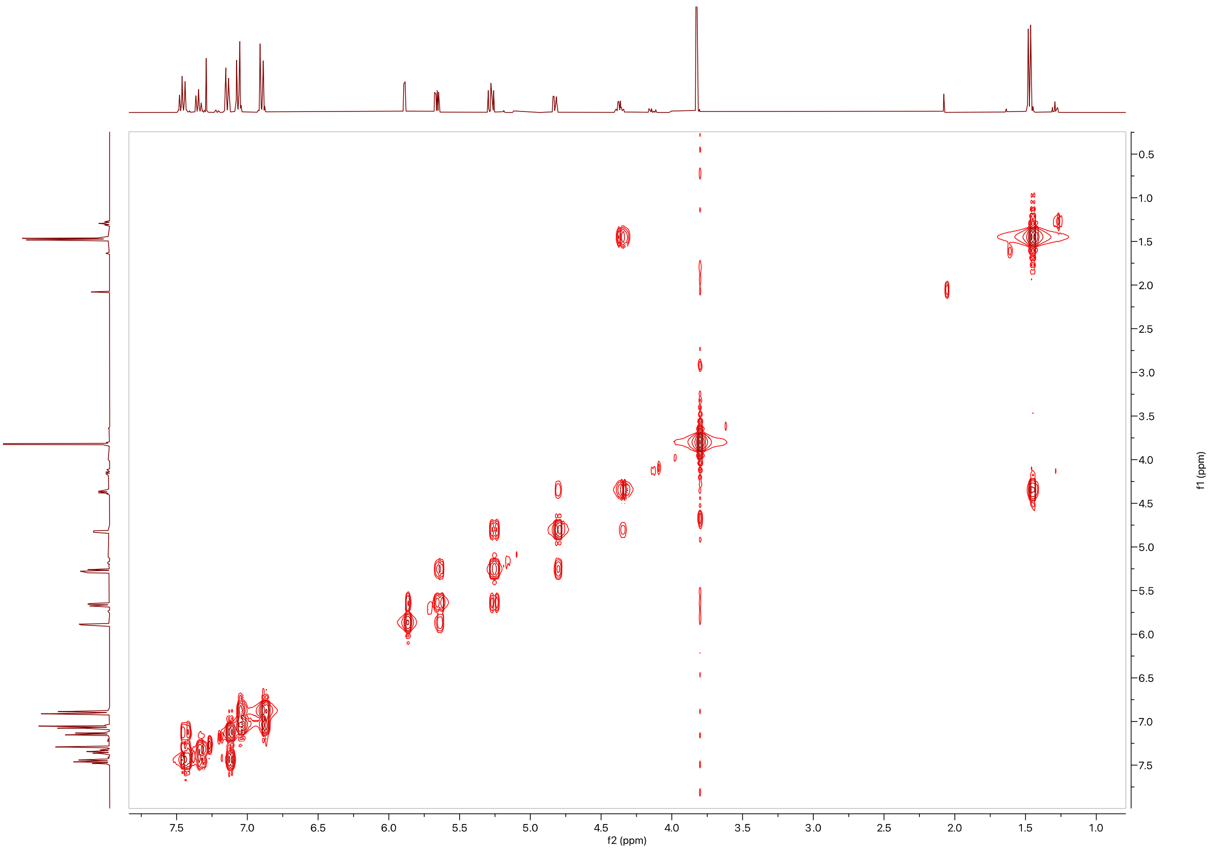

7.5
7.0
6.5
6.0
5.5
5.0
4.5
4.0
3.5
3.0
2.5
2.0
1.5
1.0
f2 (ppm)
f1 (ppm)

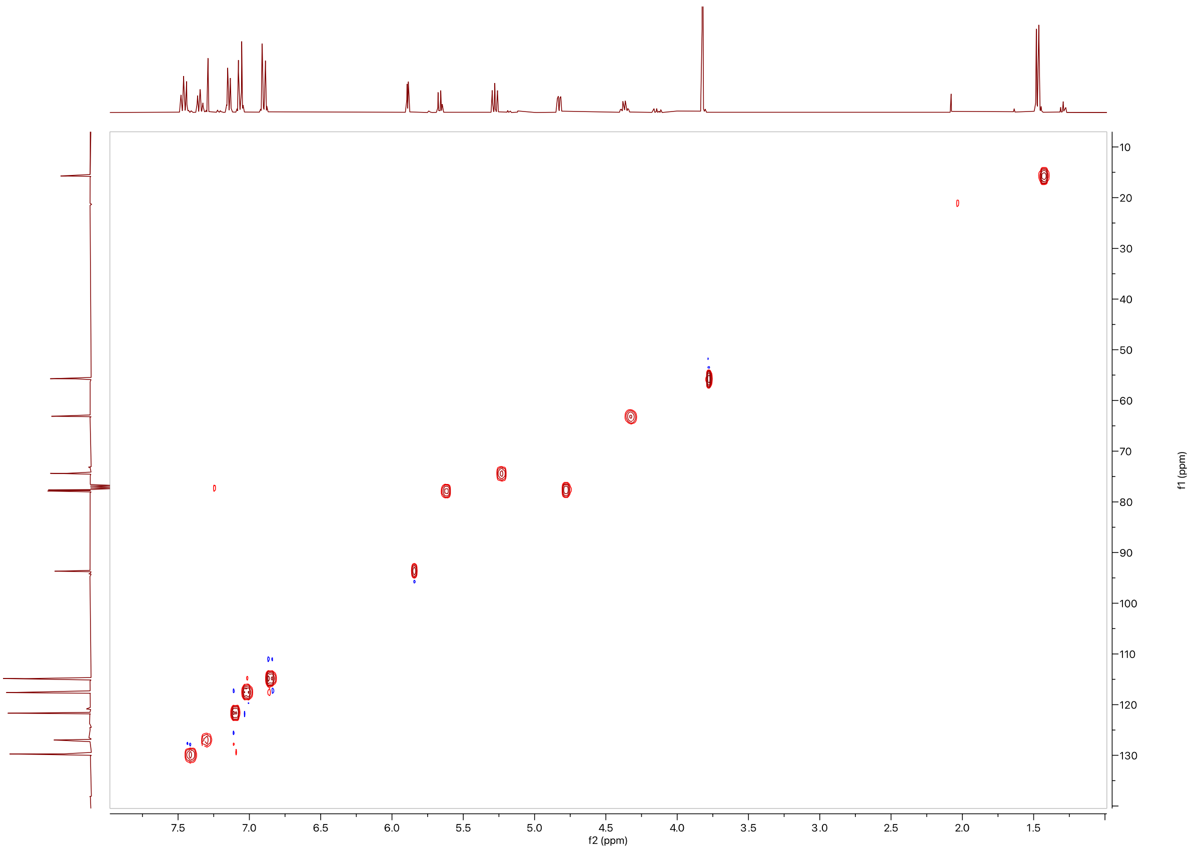

7.5
7.0
6.5
6.0
5.5
5.0
4.5
4.0
3.5
3.0
2.5
2.0
1.5
f2 (ppm)
f1 (ppm)

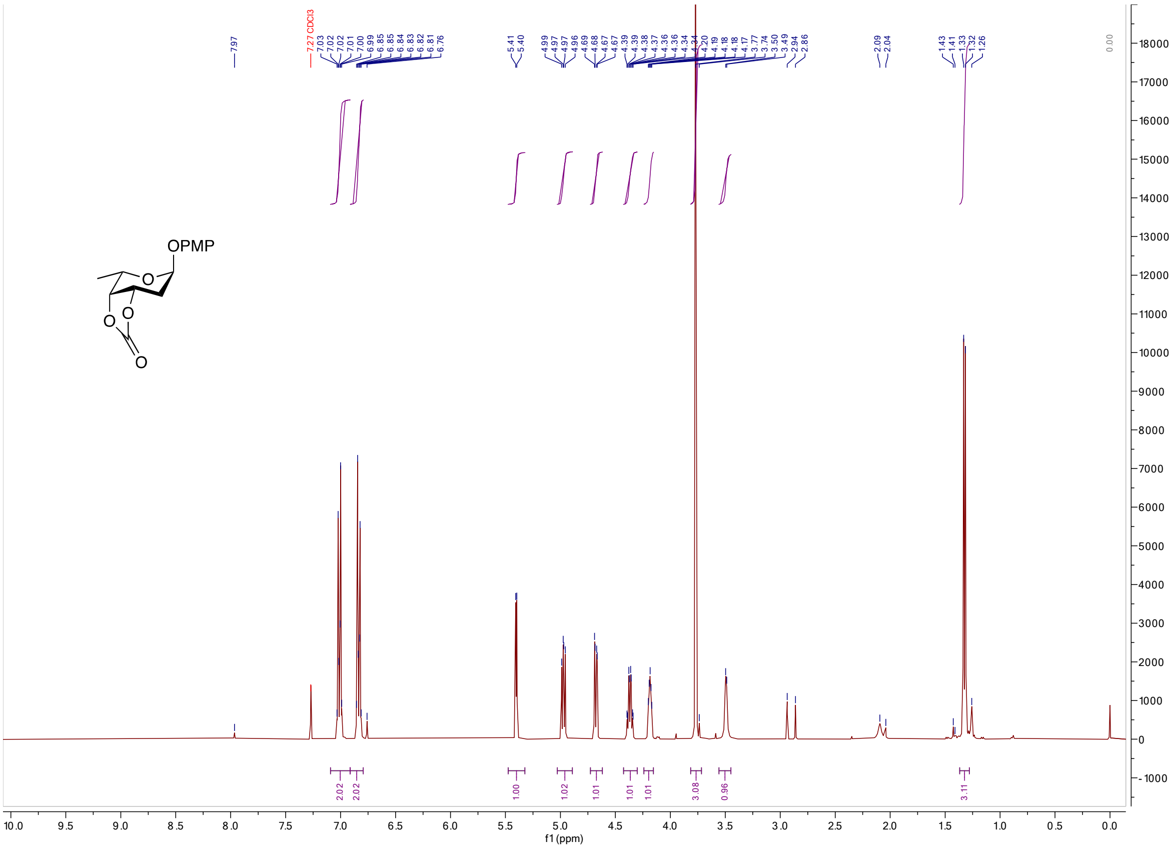
OPMP
O
O
O
O
7.97
7.27 CDCl3
7.03 7.02 7.02 7.01 7.00 6.99 6.85 6.85 6.84 6.83 6.82 6.81 6.76
5.41 5.40
4.99 4.97 4.97 4.96 4.69 4.68 4.67 4.67 4.39 4.39 4.38 4.37 4.36 4.36 4.34 4.34 4.20 4.19 4.18 4.18 4.17 3.77 3.74 3.50 3.49 2.94 2.86
2.09 2.04
1.43 1.41 1.33 1.32 1.26
0.00
2.02 2.02 1.00 1.02 1.01 1.01 1.01 3.08 0.96 3.11
f1 (ppm)

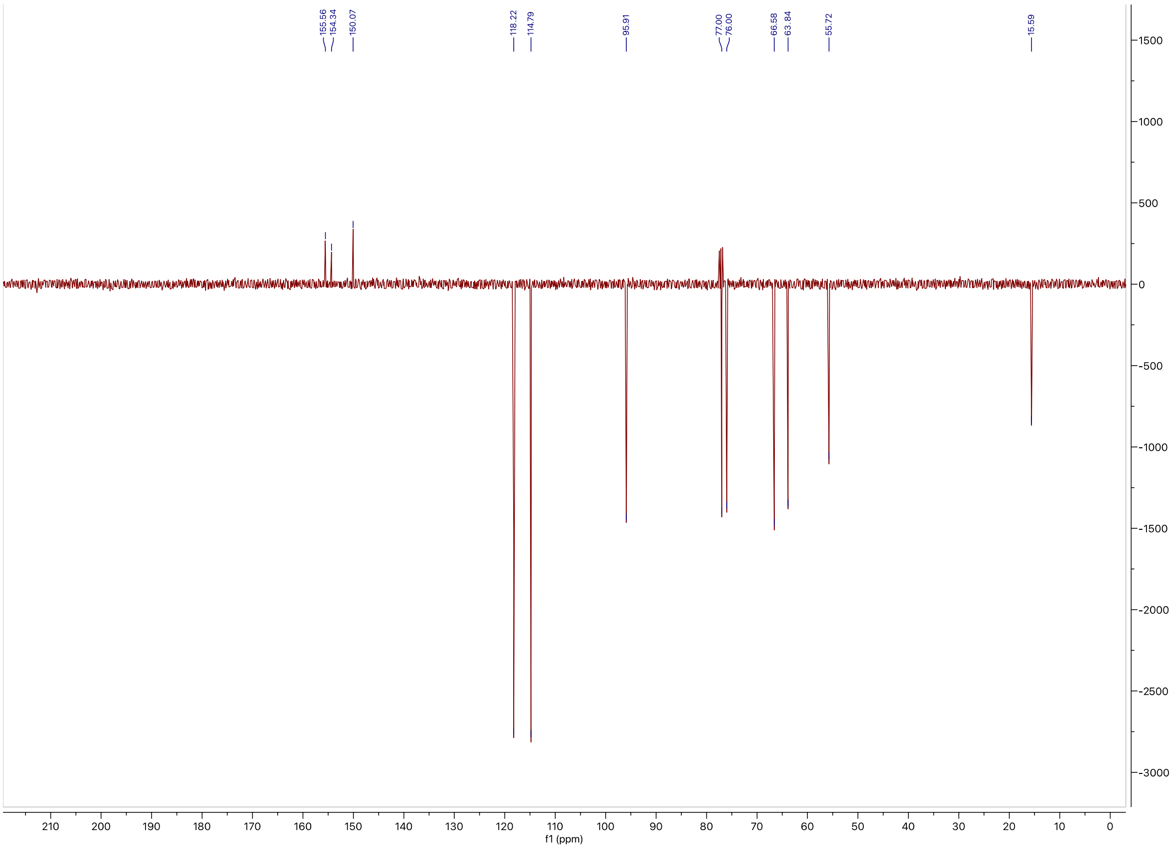
155.56 154.34 150.07
118.22 114.79
95.91
77.00 76.00
66.58 63.84
55.72
15.59
f1 (ppm)

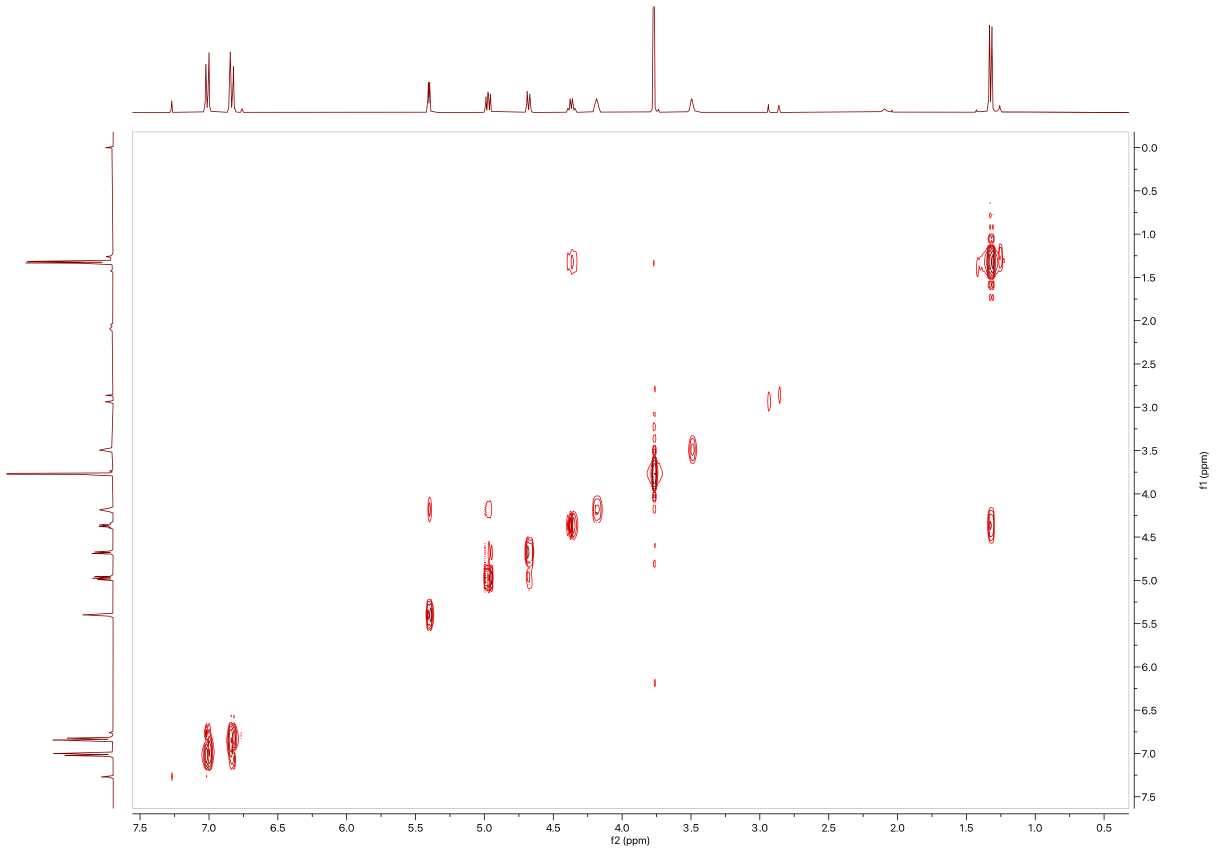
7.5 7.0 6.5 6.0 5.5 5.0 4.5 4.0 3.5 3.0 2.5 2.0 1.5 1.0 0.5
f2 (ppm)
0.0 0.5 1.0 1.5 2.0 2.5 3.0 3.5 4.0 4.5 5.0 5.5 6.0 6.5 7.0 7.5
f1 (ppm)

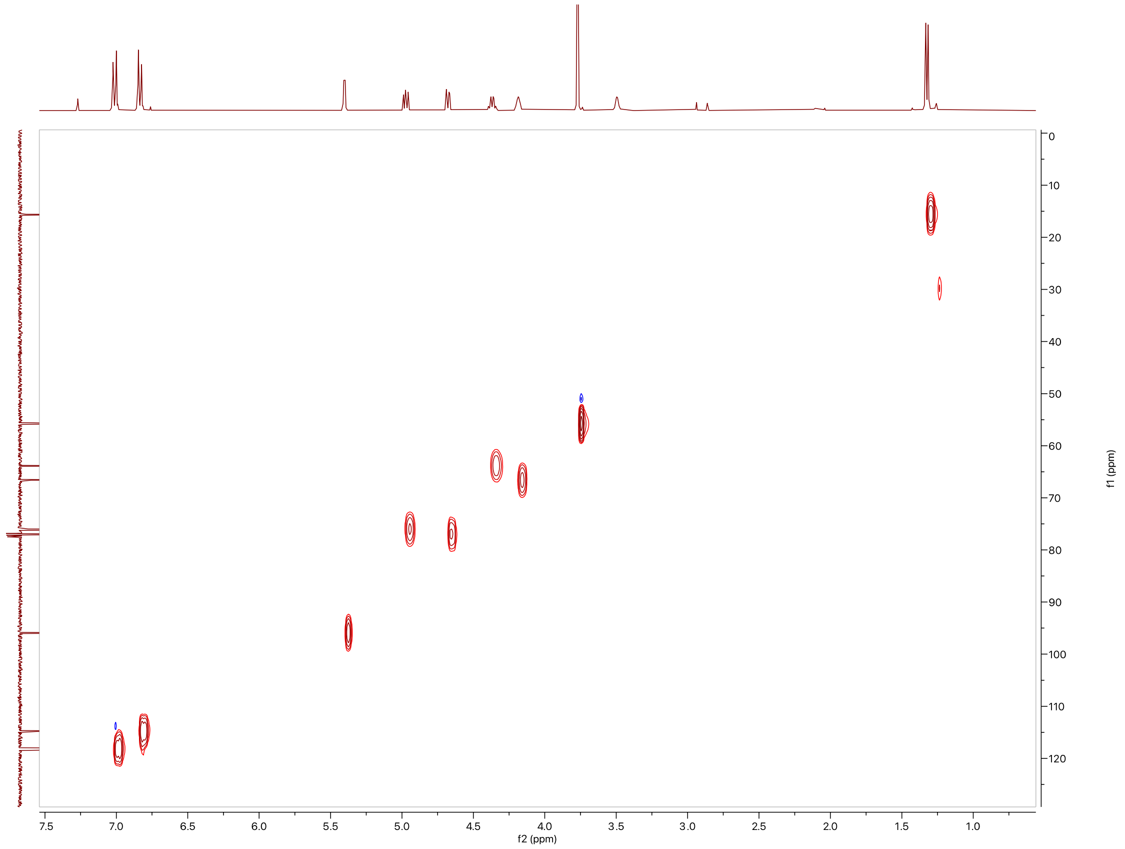
7.5 7.0 6.5 6.0 5.5 5.0 4.5 4.0 3.5 3.0 2.5 2.0 1.5 1.0
f2 (ppm)
0 10 20 30 40 50 60 70 80 90 100 110 120
f1 (ppm)

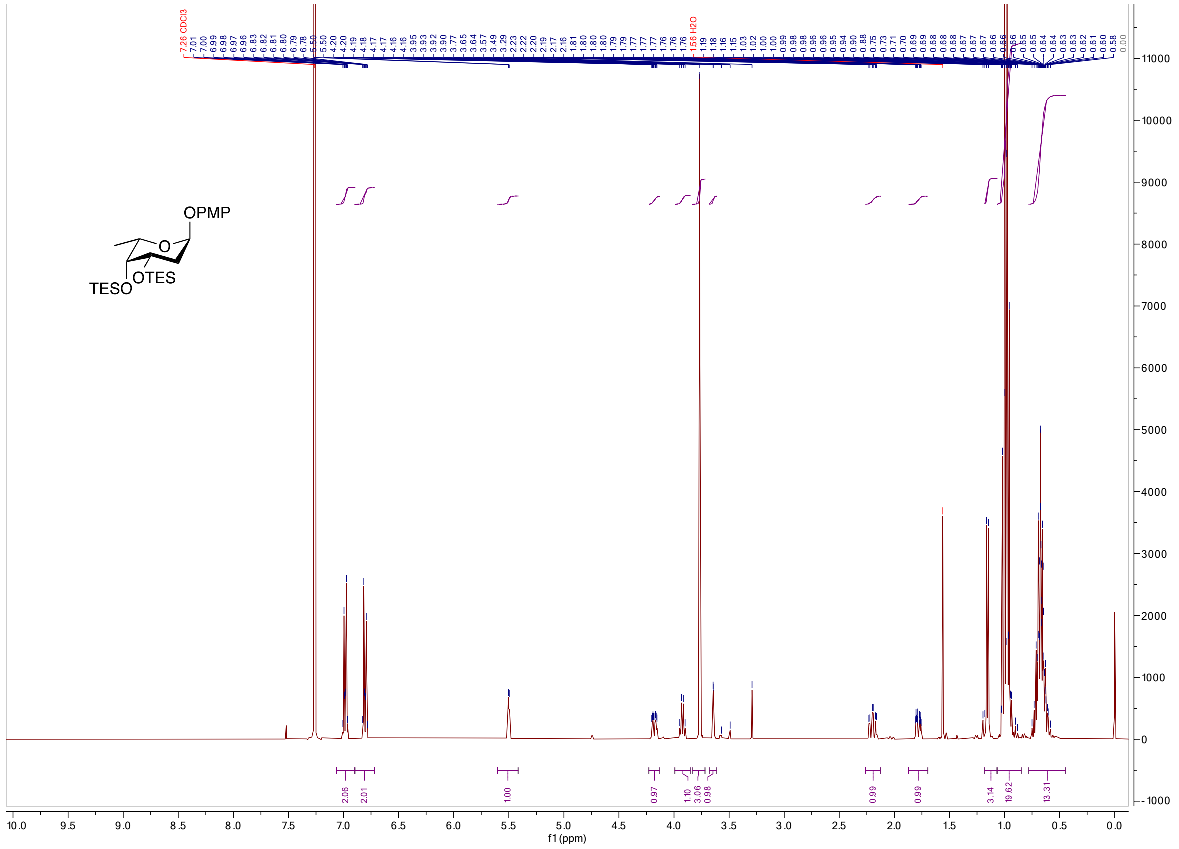
OPMP
O
TESO
OTES
2.06
2.01
1.00
0.97
1.10
3.06
0.98
0.99
0.99
3.14
19.62
13.31
f1 (ppm)

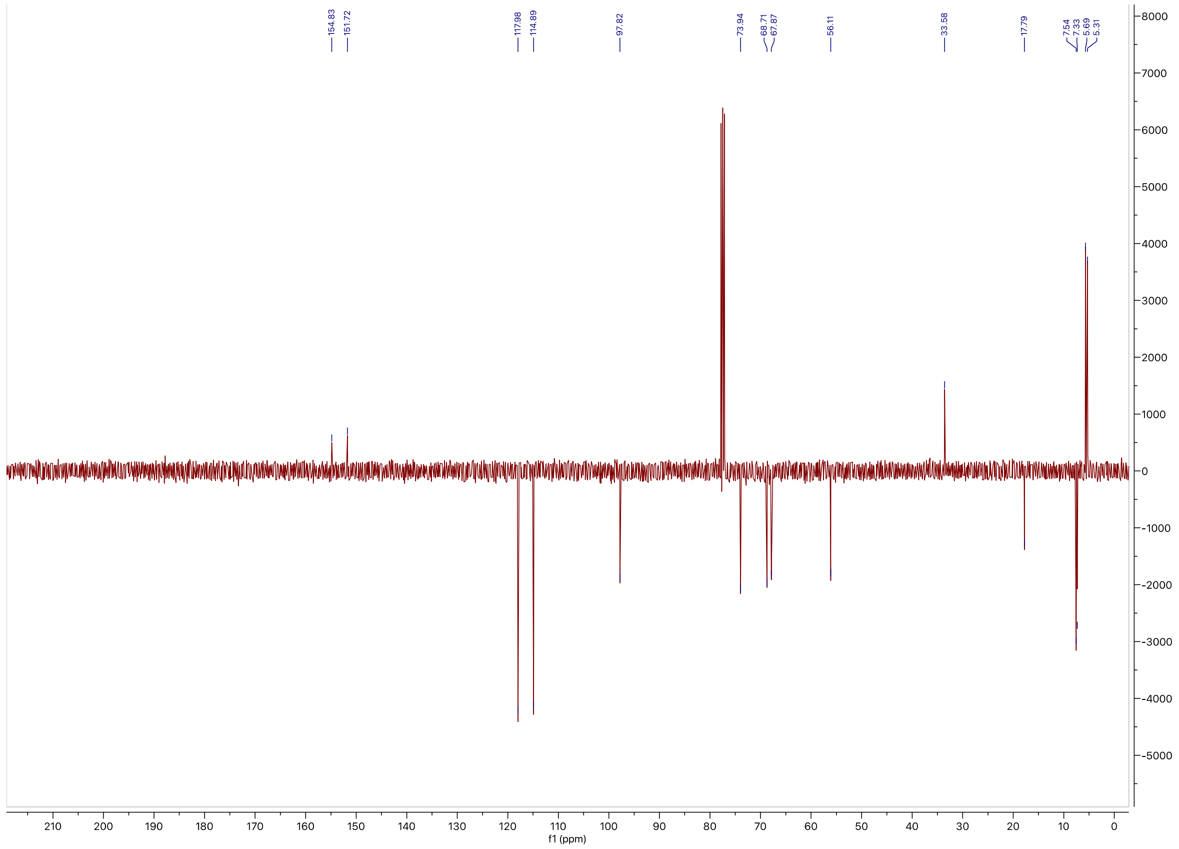
154.83
151.72
117.98
114.89
97.82
73.94
68.71
67.87
56.11
33.58
17.79
7.54
7.33
5.69
5.31
f1 (ppm)

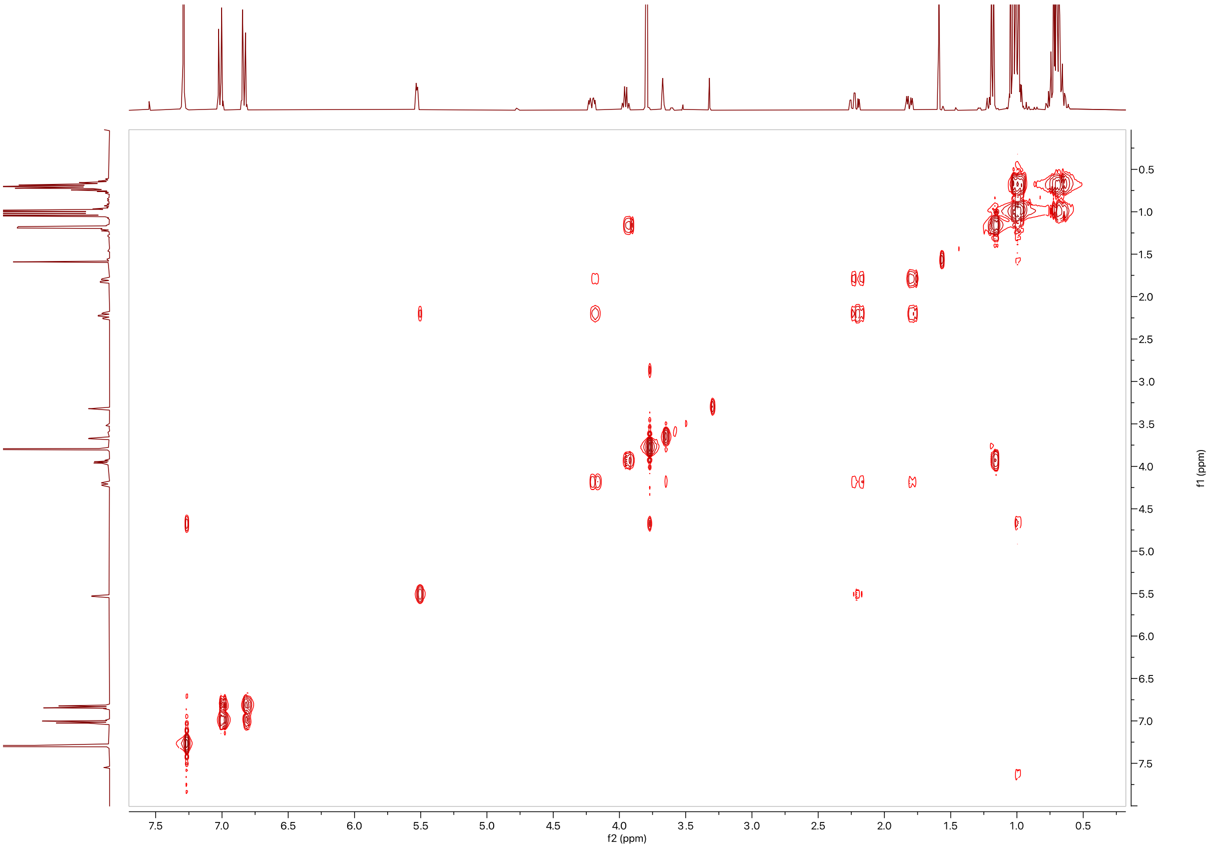
f2 (ppm)
f1 (ppm)

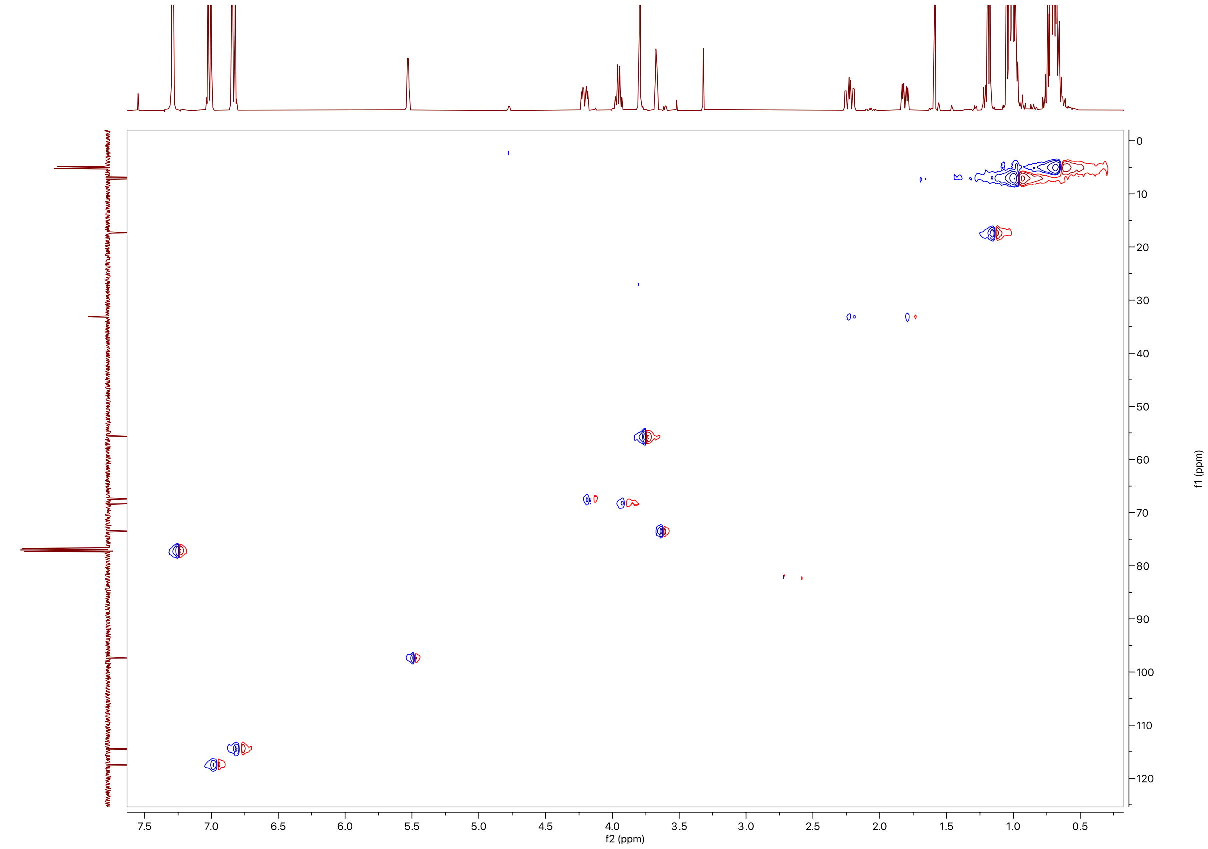
f2 (ppm)
f1 (ppm)

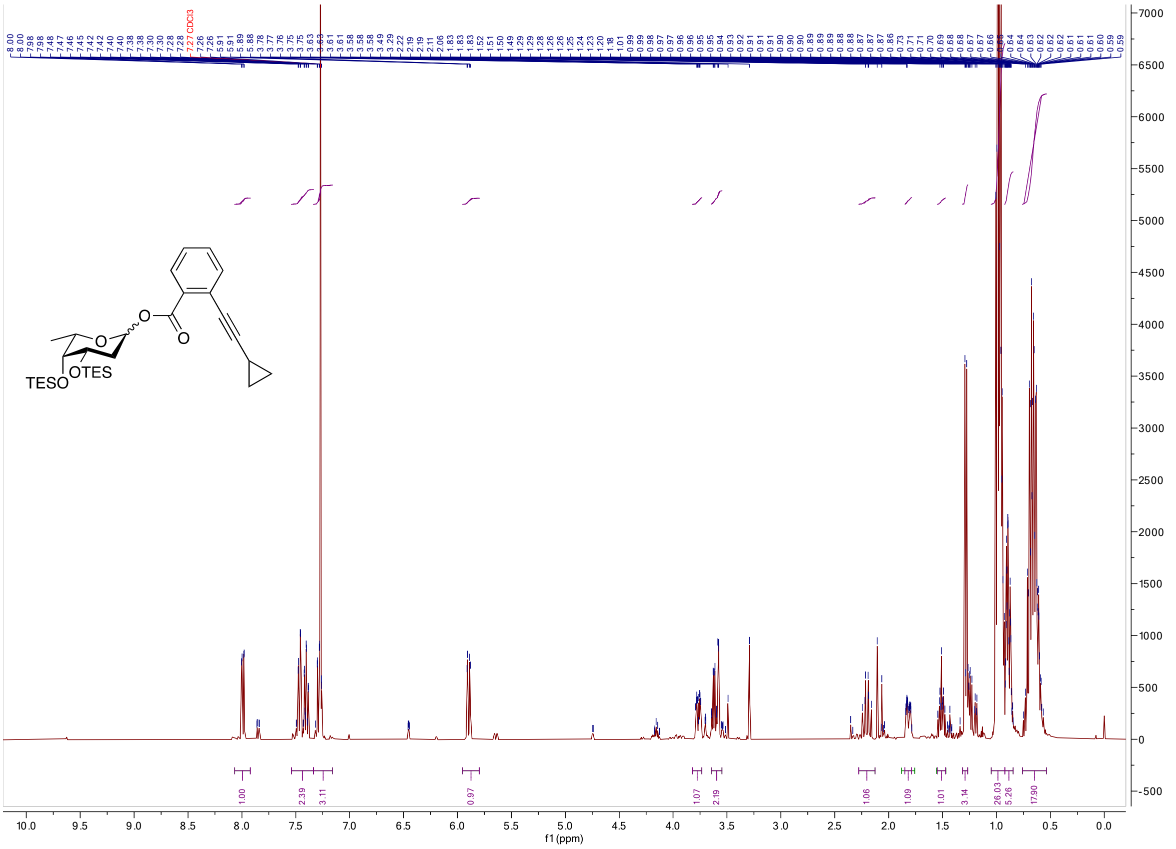

TESO
OTES
O
O
O
7.27 CDCl3
f1 (ppm)

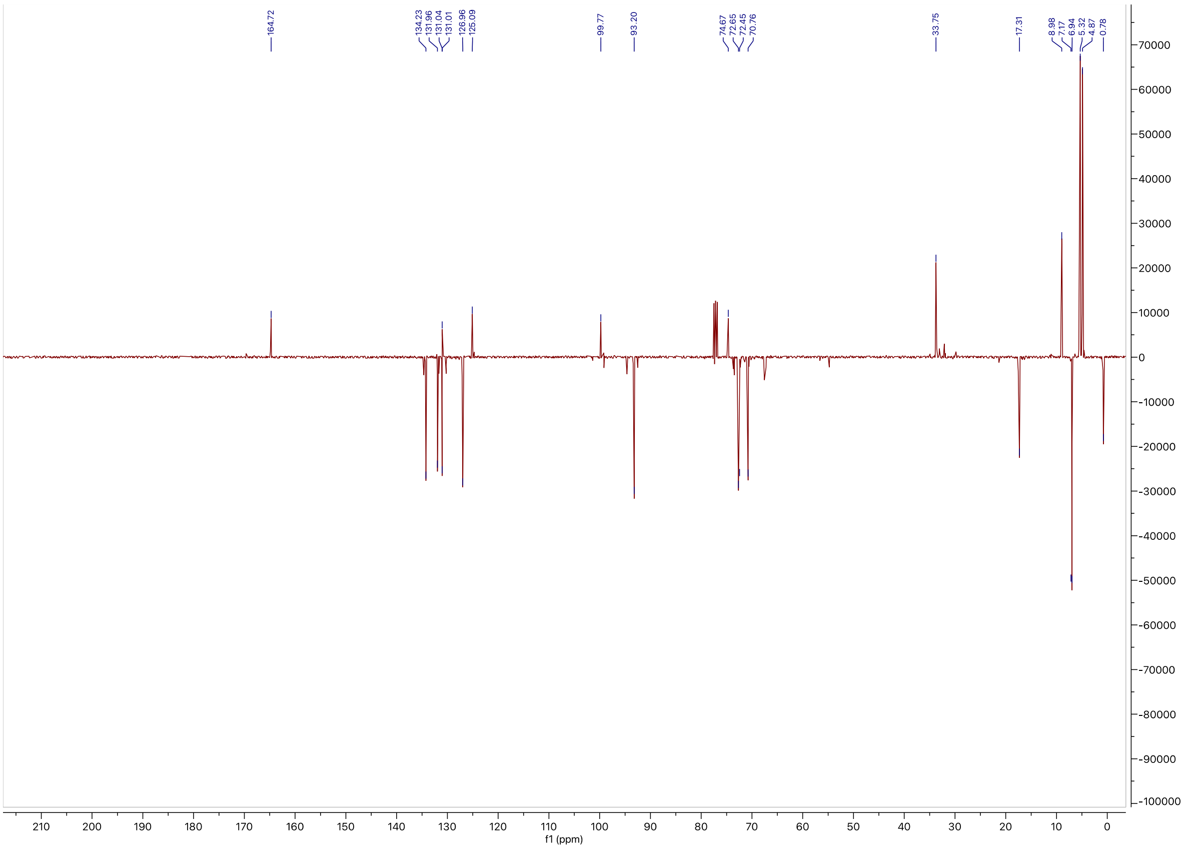

164.72
134.23
131.96
131.04
131.01
126.96
125.09
99.77
93.20
74.67
72.65
72.45
70.76
33.75
17.31
8.98
7.17
6.94
5.32
4.87
0.78
f1 (ppm)

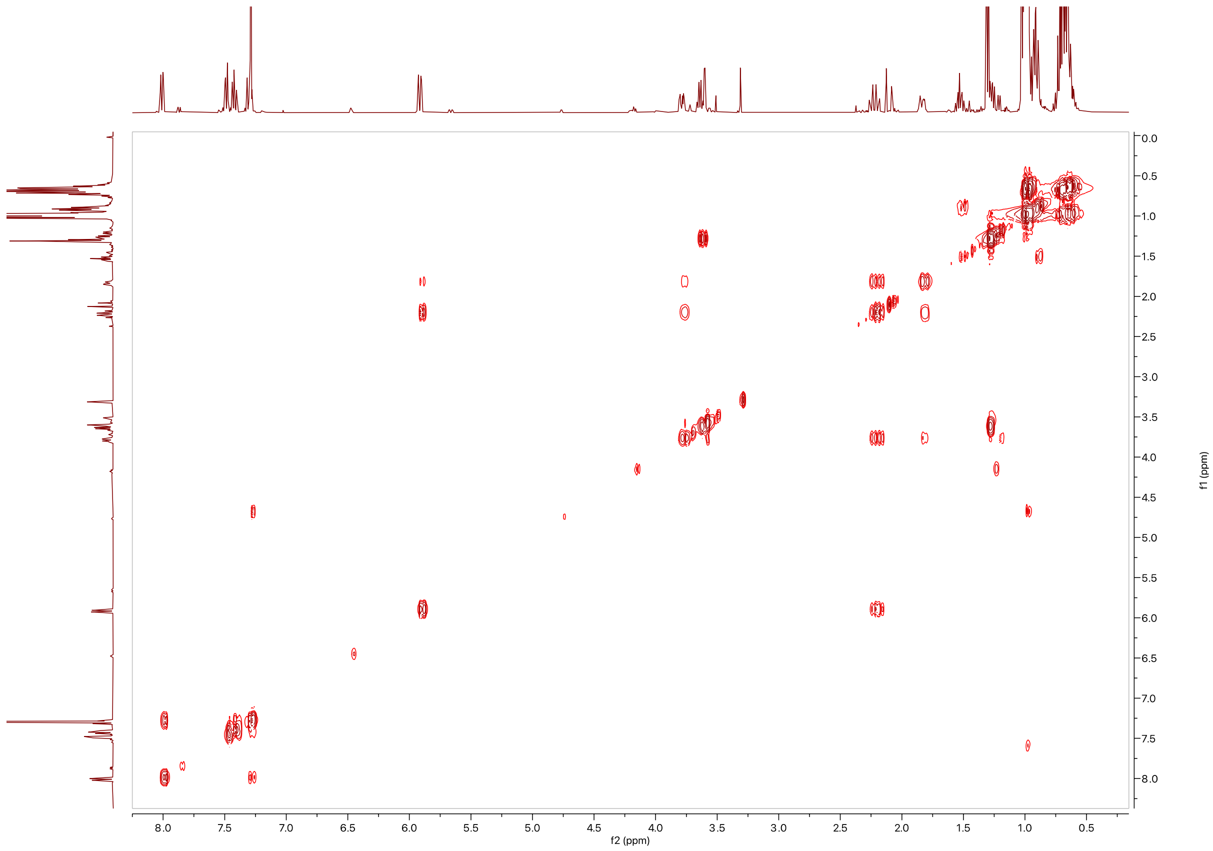
f2 (ppm)
f1 (ppm)

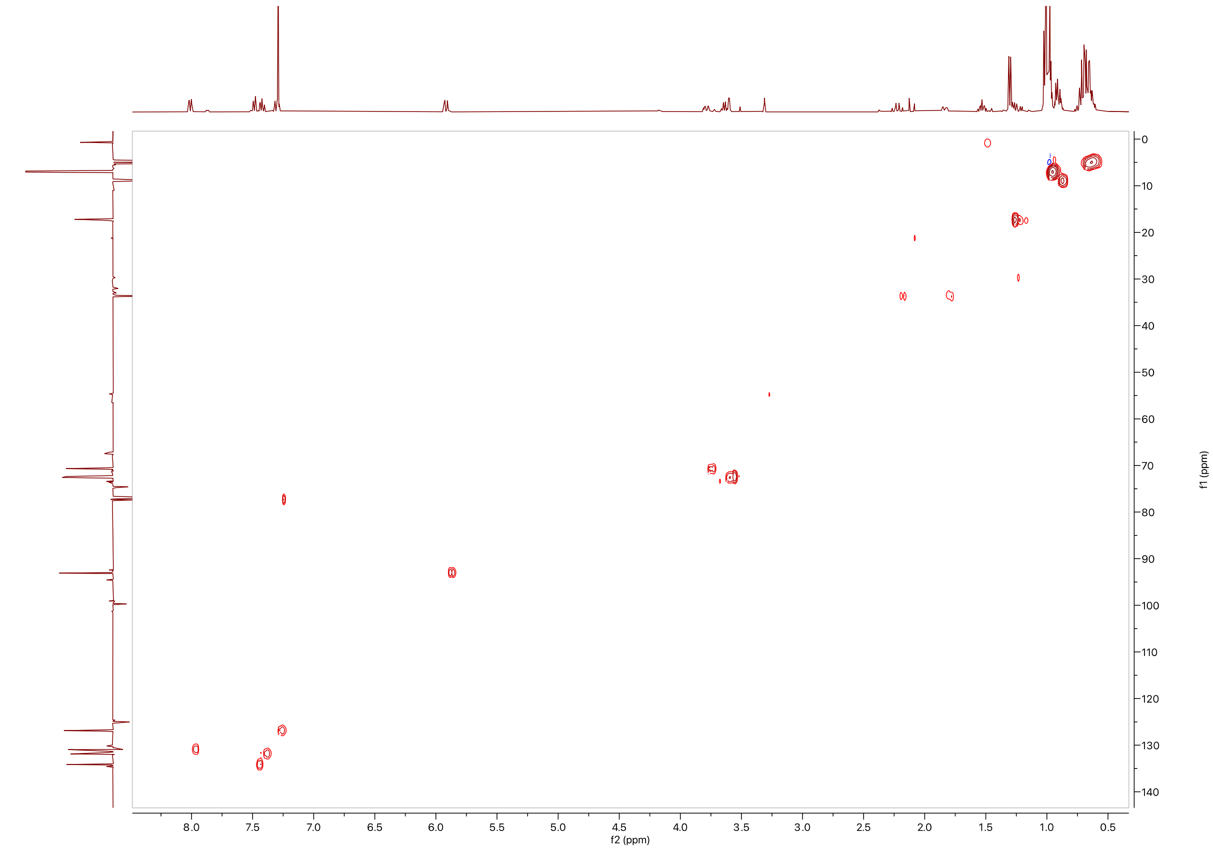
f2 (ppm)
f1 (ppm)

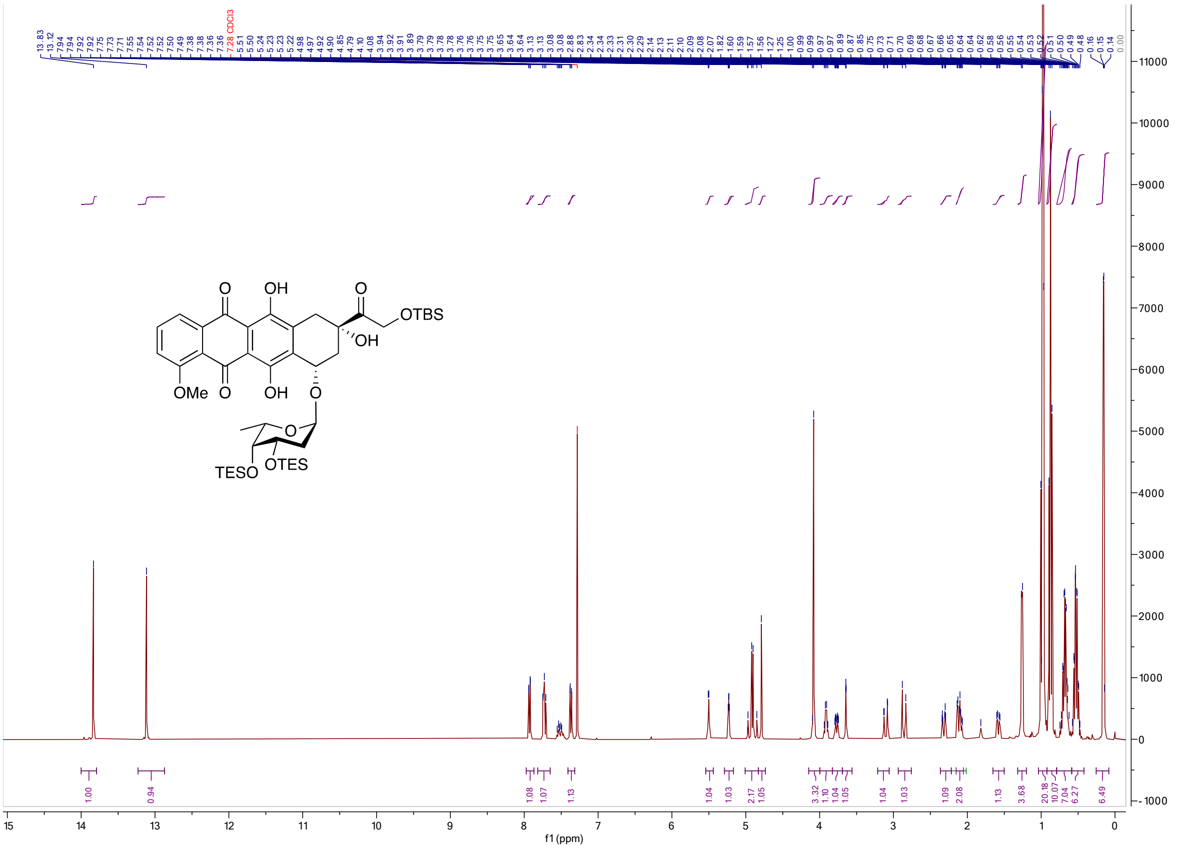
OH
O
OTBS
OH
OMe
OH
O
TESO
OTES
7.28 CDCl3
f1 (ppm)

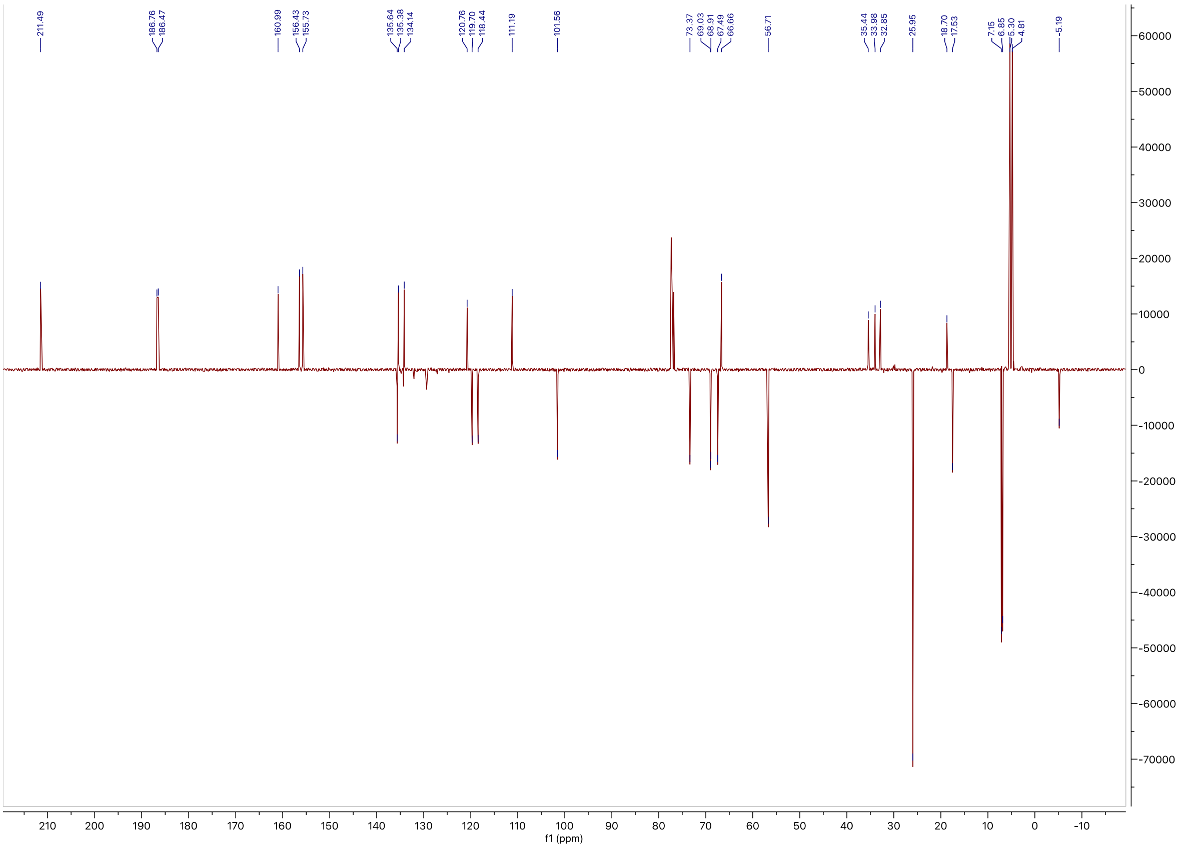
f1 (ppm)

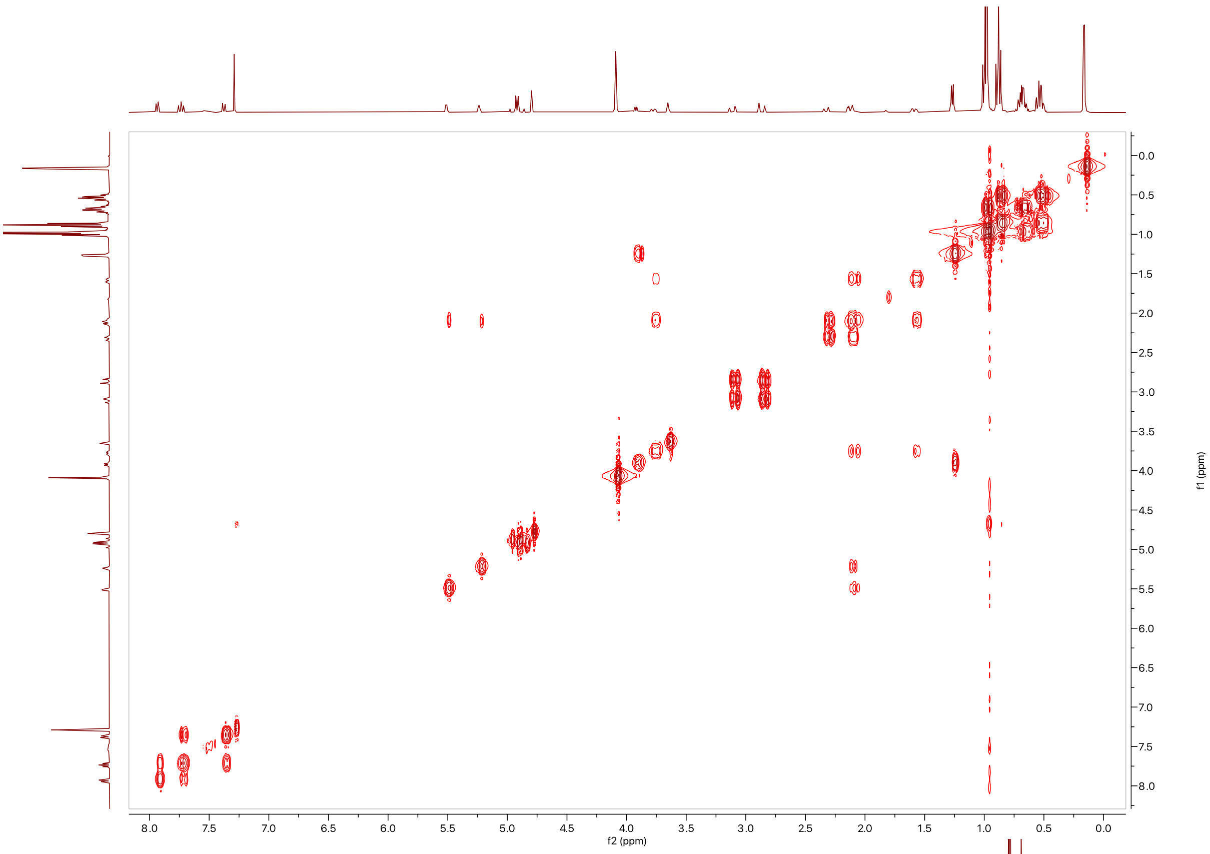
f2 (ppm)
f1 (ppm)

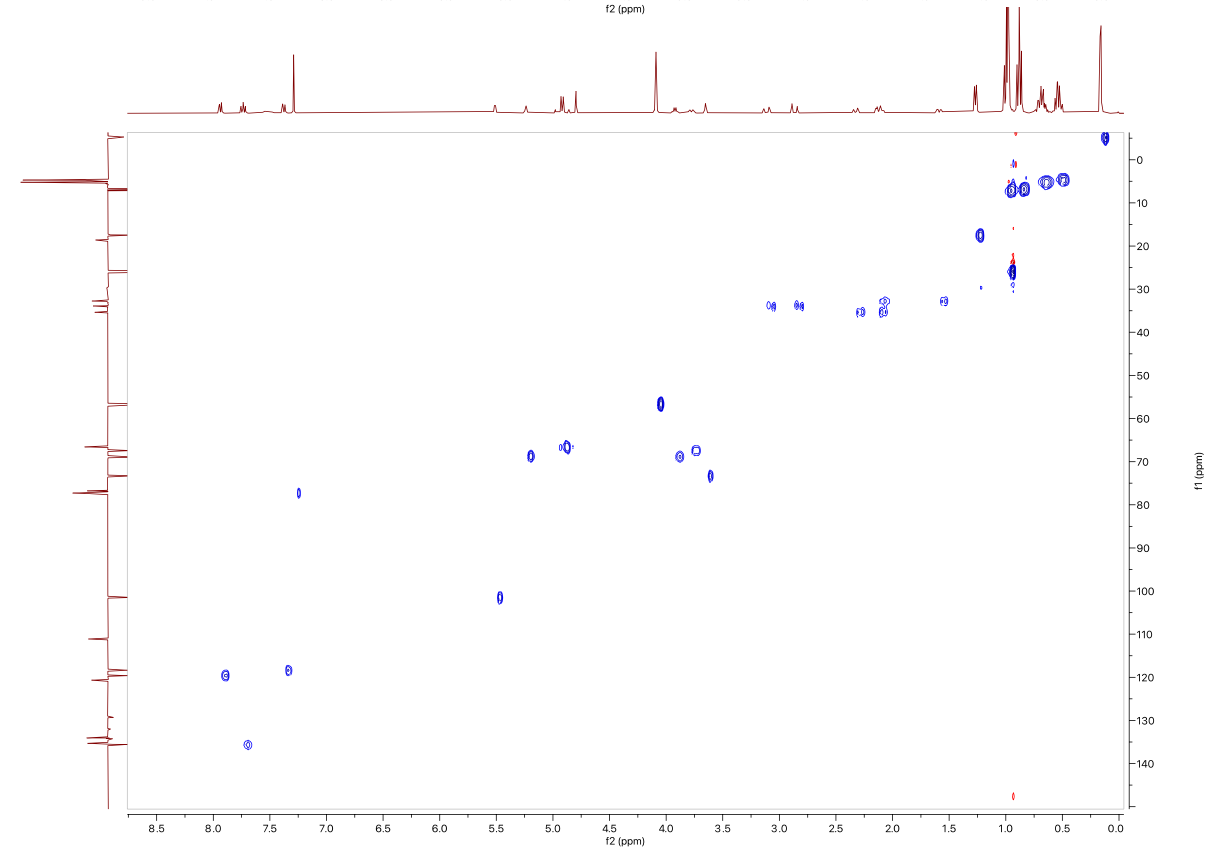
f2 (ppm)
f1 (ppm)

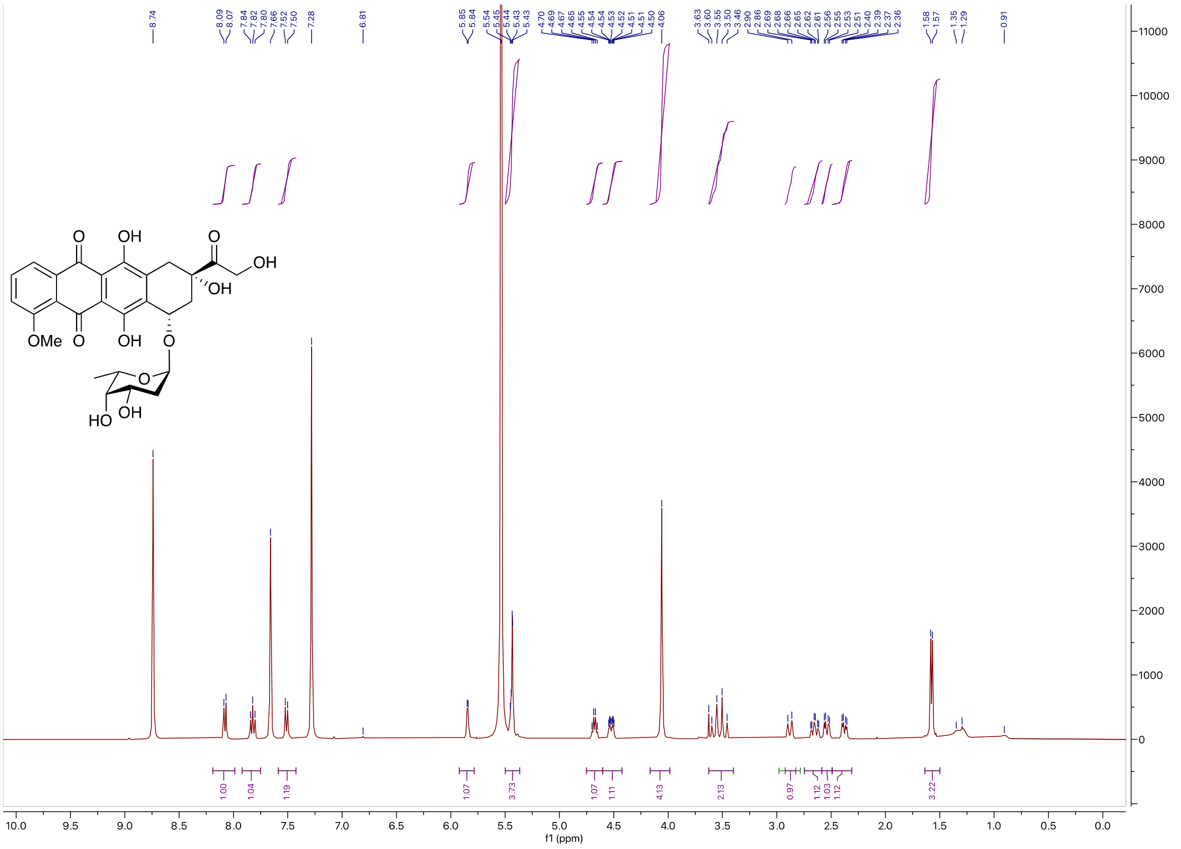
8.74
8.09
8.07
7.84
7.82
7.80
7.66
7.52
7.50
7.28
6.81
5.85
5.84
5.54
5.45
5.44
5.43
5.43
4.70
4.69
4.67
4.65
4.55
4.54
4.54
4.53
4.52
4.51
4.51
4.50
4.06
3.63
3.60
3.55
3.50
3.46
2.90
2.86
2.69
2.68
2.66
2.65
2.62
2.61
2.56
2.55
2.53
2.51
2.40
2.39
2.37
2.36
1.58
1.57
1.35
1.29
0.91
OH
O
OH
OMe
HO
1.00
1.04
1.19
1.07
3.73
1.07
1.11
4.13
2.13
0.97
1.12
1.03
1.12
3.22
f1 (ppm)
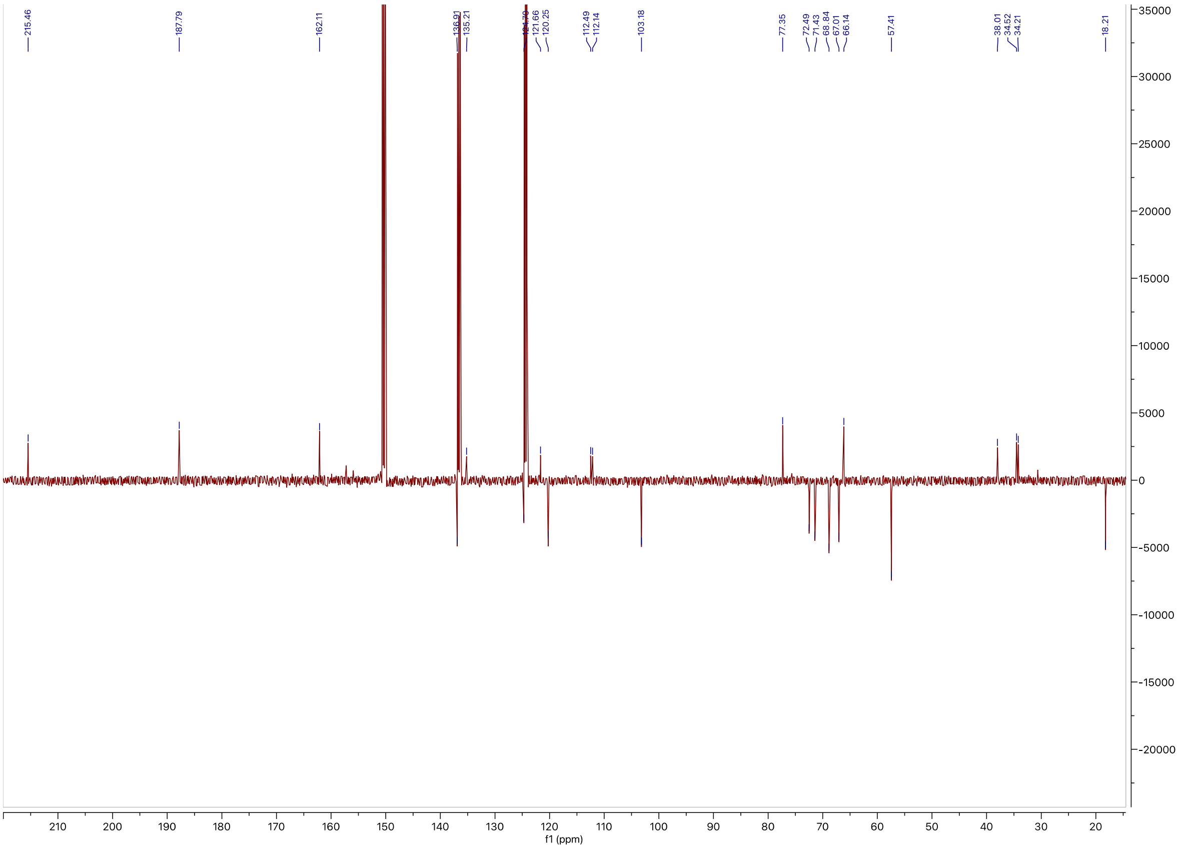
215.46
187.79
162.11
136.91
135.21
121.66
120.25
112.49
112.14
103.18
77.35
72.49
71.43
68.84
67.01
66.14
57.41
38.01
34.52
34.21
18.21
f1 (ppm)

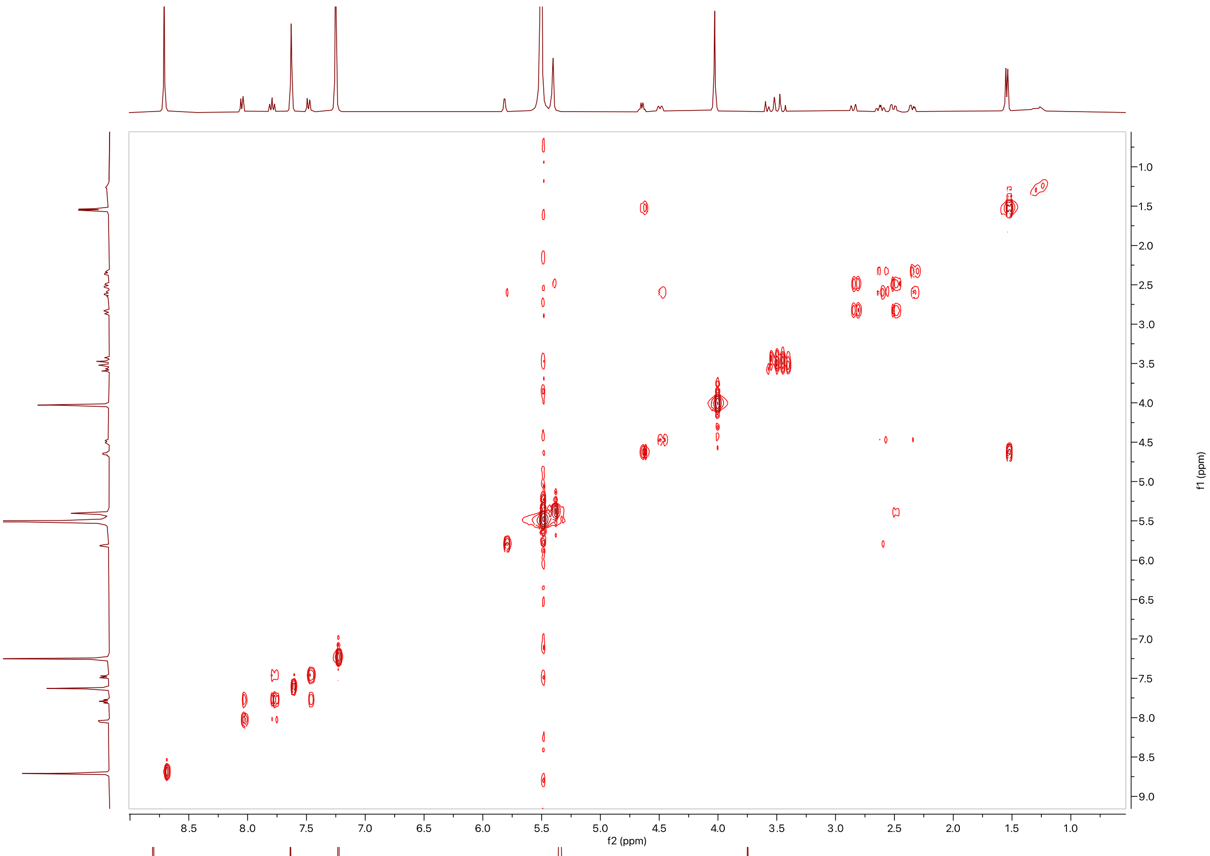
f2 (ppm)
f1 (ppm)

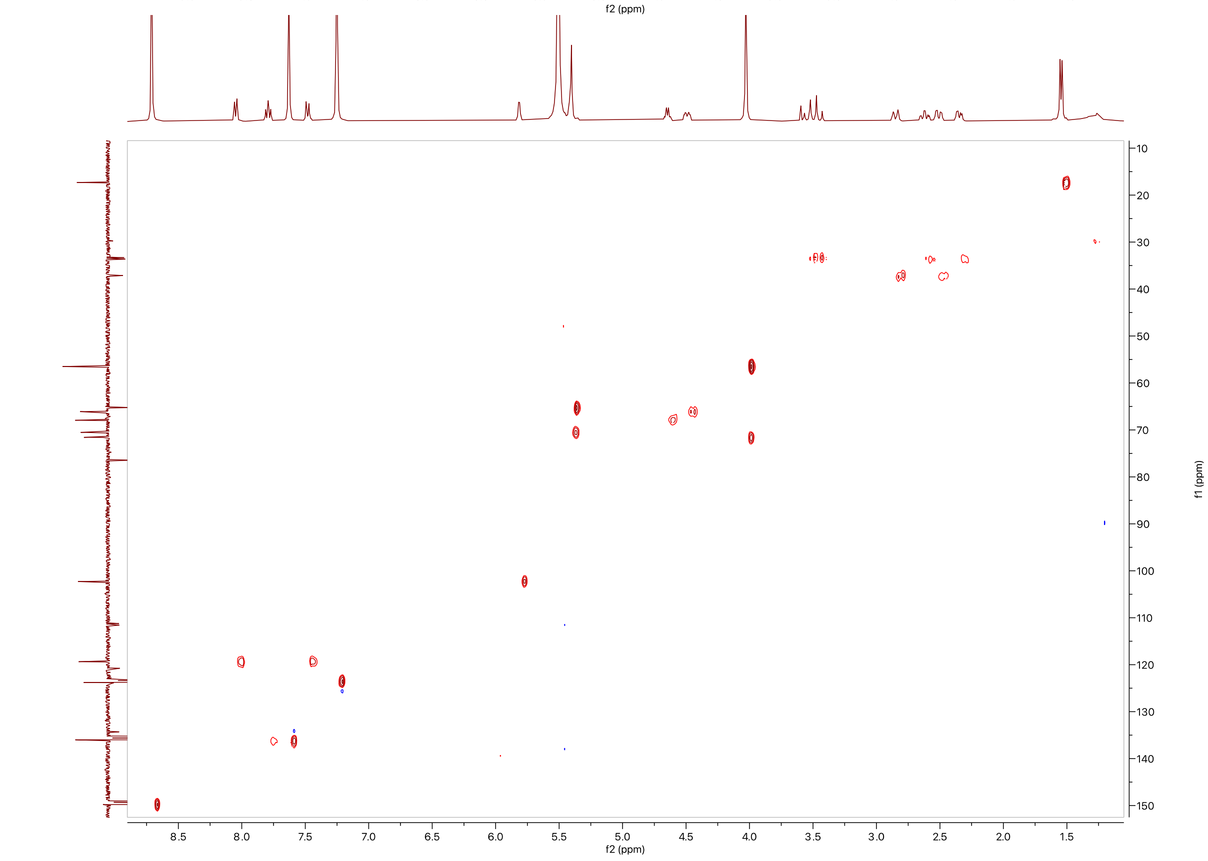
f2 (ppm)
f1 (ppm)

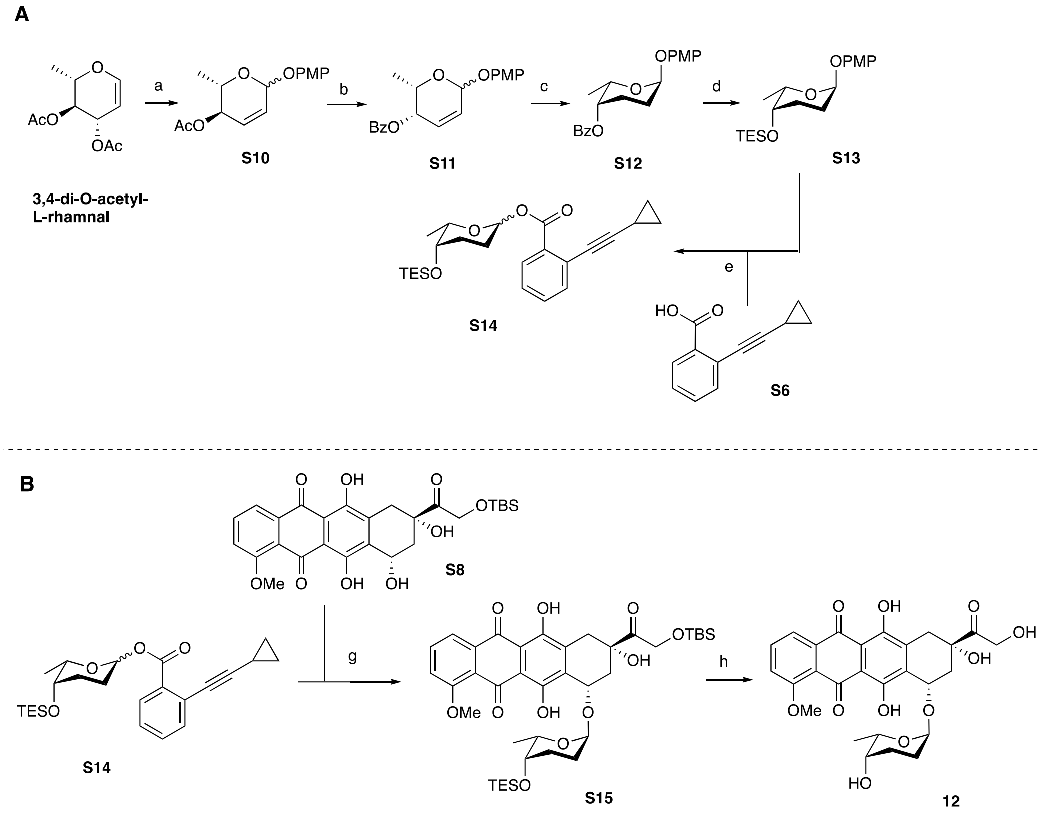

Scheme S2. Synthesis of 3’-desaminodoxorubicin (**12**). (A) Synthesis of 2-rhodinosyl alkynylbenzoate donor **S14**; (B) Glycosylation and deprotection. *Reagents and conditions:* (a) *p*-methoxyphenol, $BF_3 \cdot OEt_2$, toluene, -10 °C, 41%, (12.5:1 α:β); (b) *i.* NaOMe, MeOH; *ii.* benzoic acid, PPh_3, diethylazodicarboxylate, THF, 0 °C to RT, 80% over 2 steps; (c) Rh/Al_2O_3, H_2, toluene, EtOAc, 0 °C, quant.; (d) *i.* NaOMe, MeOH; *ii.* triethylsilyl triflate, pyr., DMF, 73% over 2 steps; (e) *i.* Ag(II)(hydrogen dipicolinate)$_2$, NaOAc, ACN, H_2O, 0 °C; *ii.* EDCI·HCl, DIPEA, DMAP, DCM, 61% over 2 steps (1:9 α:β); (g) PPh_3AuNTf_2, DCM, 39% (α-only); (h) HF·pyridine, THF/pyr., 93%.

p-Methoxyphenyl-4-*O*-acetyl-2,3,6-trideoxy-L-*erythro*-hexopyranoside (S10)[10,11]

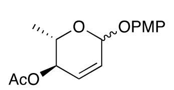

3,4-di-*O*-acetyl-L-rhamnal (3.96 g, 18.5 mmol) and *p*-methoxyphenol (2.48 g, 20.0 mmol, 1.08 eq) were jointly coevaporated from toluene, after which they were dissolved in toluene (150 mL). To this solution at -10 °C was added $BF_3 \cdot OEt_2$ (0.11 mL, 0.93 mmol, 0.05 eq) and the mixture was stirred at this temperature for 2 h. It was then poured into sat. aq. $NaHCO_3$ and extracted with DCM. The resulting organic layer was washed with 1M NaOH and brine, dried over $MgSO_4$ and concentrated *in vacuo*. Column chromatography (5:95 Et_2O:pentane) gave the title compound as a white solid (2.13 g, 7.65 mmol, 41%, 12.5:1 α:β). Spectral data was in accordance with that of literary precedence.[10,11]

p-Methoxyphenyl-4-O-benzoyl-2,3,6-trideoxy-L-threo-hexopyranoside (S11)

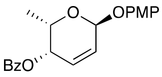

To a solution of **S10** (2.13 g, 7.65 mmol, 12.5:1 α:β) in MeOH (77 mL) was added NaOMe (83 mg, 1.54 mmol, 0.2 eq) and the mixture was stirred for 1.5 hours. It was then quenched by addition of dry ice and concentrated *in vacuo*. The residue was partitioned between EtOAc and H_2O, after which the organic layer was dried over $MgSO_4$ and concentrated *in vacuo*. The resulting allylic alcohol was then dissolved in THF (17 mL), together with benzoic acid (1.96 g, 16.1 mmol, 2.1 eq) and triphenylphosphine (4.21 g, 16.1 mmol, 2.1 eq). To this, diethyl azodicarboxylate (4.6 mL, 14.9 mmol, 1.95 eq) was added dropwise at 0°C. After stirring overnight, the reaction mixture was concentrated *in vacuo*. Then, Et_2O was added to the residue and this was filtered off. The filtrate was washed with sat. aq. $NaHCO_3$ twice, dried over $MgSO_4$ and concentrated *in vacuo*. Column chromatography (5:95 Et_2O:pentane) gave the title compound as an orange oil (2.07 g, 6.08 mmol, 80% over 2 steps). ^{1}H NMR (400 MHz, Chloroform-*d*) δ 8.11 – 8.08 (m, 2H), 7.58 (ddt, *J* = 7.8, 6.9, 1.3 Hz, 1H), 7.50 – 7.41 (m, 2H), 7.10 – 7.06 (m, 2H), 6.93 – 6.83 (m, 2H), 6.33 (ddd, *J* = 9.9, 5.5, 1.1 Hz, 1H), 6.21 (ddd, *J* = 9.9, 3.2, 0.6 Hz, 1H), 5.71 – 5.64 (m, 1H), 5.23 (ddd, *J* = 5.5, 2.5, 0.6 Hz, 1H), 4.51 (qd, *J* = 6.6, 2.5 Hz, 1H), 3.79 (s, 3H), 1.31 (d, *J* = 6.6 Hz, 3H). ^{13}C NMR (101 MHz, $CDCl_3$) δ 166.3, 155.1, 151.5, 133.4, 129.9, 128.6, 126.8, 118.4, 114.7, 94.1, 65.9, 65.5, 55.8, 16.3. HRMS: $(M + Na)^+$ calculated for $C_{20}H_{20}O_5Na$ 363.1208; found 363.1214.

p-Methoxyphenol-4-O-benzoyl-2,3-dideoxy-α-L-fucopyranoside (S12)

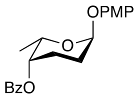

To a solution of **S11** (2.07 g, 6.08 mmol) in toluene:EtOAc (9:1 v/v, 125 mL) was added rhodium on alumina (5% rhodium, 250 mg) at 0°C. The reaction was then placed under hydrogen atmosphere and stirred overnight. It was then filtered off over Celite and concentrated *in vacuo* to give the title compound as a light-yellow solid (2.08 g, 6.08 mmol, quant.). ^{1}H NMR (400 MHz, Chloroform-*d*) δ 8.15 – 8.12 (m, 2H), 7.59 (ddt, *J* = 8.7, 7.0, 1.3 Hz, 1H), 7.54 – 7.40 (m, 2H), 7.10 – 6.99 (m, 2H), 6.88 – 6.79 (m, 2H), 5.57 (d, *J* = 2.6 Hz, 1H), 5.12 (s, 1H), 4.25 (qd, *J* = 6.6, 1.5 Hz, 1H), 3.78 (s, 3H), 2.37 (tdd, *J* = 14.0, 4.6, 2.8 Hz, 1H), 2.21 – 2.10 (m, 1H), 2.10 – 1.96 (m, 1H), 1.83 (ddt, *J* = 13.5, 4.0, 1.8 Hz, 1H), 1.16 (d, *J* = 6.5 Hz, 3H). ^{13}C NMR (101 MHz, $CDCl_3$) δ 166.3, 154.7, 151.2, 133.2, 130.4, 129.8, 128.6, 117.7, 114.7, 96.4, 70.0, 66.2, 55.8, 24.6, 23.1, 17.4. HRMS: $(M + Na)^+$ calculated for $C_{20}H_{22}O_5Na$ 365.1365; found 365.1362.

p-Methoxyphenol-2,3-dideoxy-1-thio-α-L-fucopyranoside (S13)

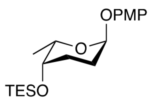

A solution of **S12** (2.08 g, 6.08 mmol) in dioxane (40 mL), MeOH (40 mL) and 1M NaOH (20 mL) was stirred at 60 °C for 2.5 hours, after which it was concentrated *in vacuo*. The residue was partitioned between EtOAc and sat. aq. NH_4Cl, after which the organic layer was dried over $MgSO_4$ and concentrated *in vacuo*. The crude alcohol was then redissolved in DMF (10 mL), after which pyridine (1.47 mL, 18.2 mmol, 3 eq) and triethylsilyl triflate (2.47 mL, 10.9 mmol, 1.8 eq) were added at 0°C and allowed to stir overnight. The reaction mixture was then partitioned between EtOAc and sat. aq. $NaHCO_3$, after which the organic layer was dried over $MgSO_4$ and concentrated *in vacuo*. Column chromatography (2:98:1 – 5:95:1 Et_2O:pentane:Et_3N) gave the title compound as a light yellow oil (1.57 g, 4.45 mmol, 73% over 2 steps). ^{1}H NMR (400 MHz, Chloroform-*d*) δ 7.08 – 6.91 (m, 2H), 6.89 – 6.70 (m, 2H), 5.46 (t, *J* = 2.0 Hz, 1H), 4.05 – 3.89 (m, 1H), 3.77 (s, 3H), 3.67 – 3.62 (m, 1H), 2.23 – 2.15 (m, 2H), 1.75 – 1.63 (m, 2H), 1.10 (d, *J* = 6.5 Hz, 3H), 0.99 (t, *J* = 7.9 Hz, 9H), 0.73 – 0.55 (m, 6H). ^{13}C NMR (101 MHz, $CDCl_3$) δ 154.4, 151.5, 117.6, 114.6, 96.4, 67.8, 55.8, 26.5, 23.9, 17.6, 7.1, 5.0. HRMS: $(M + Na)^+$ calculated for $C_{19}H_{32}O_4SiNa$ 375.1968; found 375.197.

o-Cyclopropylethynylbenzoyl-2,3-dideoxy-4-O-triethylsilyl-L-fucopyranoside (S14)

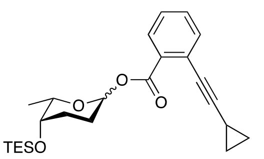

To a solution of **S13** (386 mg, 0.93 mmol) in MeCN:H_2O (50 mL, 1:1 v/v) were added NaOAc (808 mg, 9.3 mmol, 10 eq) and then Ag(DPAH)$_2$·H_2O[5] (1.76 g, 3.72 mmol, 4 eq) at 0°C. The mixture was stirred for 30 minutes; after which it was poured into sat. aq. $NaHCO_3$. This was then extracted with DCM thrice, dried over $MgSO_4$ and concentrated *in vacuo*. Column chromatography (10:90 – 50:50 Et_2O:pentane) gave the lactol. To a solution of this in DCM were added DIPEA (0.75 mL, 4.2 mmol, 4.5 eq), DMAP (119 mg, 0.93 mmol, 1 eq), EDCI·HCl (581 mg, 2.93 mmol, 3.2 eq) and freshly saponified cyclopropylethynylbenzoic acid **S6** (559 mg, 2.79 mmol, 3 eq). After stirring overnight, the mixture was diluted with DCM and washed with sat. aq. $NaHCO_3$ and brine. Drying over $MgSO_4$, concentration *in vacuo* and column chromatography of the residue (5:95 EtOAc:pentane) followed by size-exclusion chromatography (Sephadex LH-20, 1:1 DCM:MeOH v/v) gave the title compound as a white solid (236 mg, 0.803 mmol, 82% over 2 steps, 1:4 α:β). Spectral data for the β-anomer: ^{1}H NMR (500 MHz, Chloroform-*d*) δ 8.02 – 7.97 (m, 1H), 7.47 (td, *J* = 7.6, 1.3 Hz, 1H), 7.44 – 7.35 (m, 1H), 7.35 – 7.27 (m, 1H), 5.96 (dd, *J* = 9.0, 2.4 Hz, 1H), 3.79 (qd, *J* = 6.5, 1.9 Hz, 1H), 3.63 (p, *J* = 2.2 Hz, 1H), 2.14 – 2.05 (m, 1H), 1.98 (dq, *J* = 13.4, 5.3, 4.6 Hz, 1H), 1.82 – 1.72 (m, 2H), 1.51 (ddd, *J* = 8.2, 5.2, 2.8 Hz, 1H), 1.26 (d, *J* = 6.5 Hz, 3H), 0.99 (td, *J* = 7.9, 3.7 Hz, 9H), 0.93 – 0.82 (m, 4H), 0.64 (q, *J* = 7.9 Hz, 6H). ^{13}C NMR (126 MHz, $CDCl_3$) δ 164.8, 134.3, 131.8, 131.4, 130.9, 127.0, 125.1, 99.7, 95.2, 75.5, 67.0, 29.8, 25.1, 17.4, 9.0, 7.0, 5.0, 0.8. HRMS: (M + Na)$^+$ calculated for $C_{24}H_{34}O_4SiNa$ 437.2124; found 437.2126.

7-[2,3-Dideoxy-4-O-triethylsilyl-α-L-fucopyranoside]-14-O-tert-butyldimethylsilyl-doxorubicinone (S15)

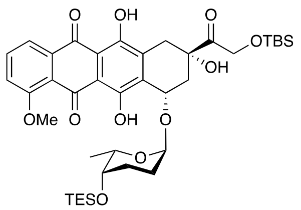

To a solution of glycosyl donor **S14** (61 mg, 0.183 mmol) and the glycosyl acceptor **S8** (109 mg, 0.27 mmol, 1.5 eq) in DCM (3.7 mL), activated molecular sieves (4Å) were added. The mixture was stirred for 30 minutes at room temperature and then a freshly prepared 0.1M DCM solution of PPh_3AuNTf_2 (prepared by stirring 1:1 PPh_3AuCl and $AgNTf_2$ in DCM for 30 minutes) (0.19 mL, 0.1 eq) in DCM was added dropwise. After 15 minutes, the mixture was filtered and concentrated *in vacuo*. Column chromatography (20:80 Et_2O:pentane and then 1:99 acetone:toluene) of the residue gave the title compound as a red solid (43 mg, 0.057 mmol, 39%). ^{1}H NMR (400 MHz, Chloroform-*d*) δ 13.89 (s, 1H), 13.24 (s, 1H), 8.00 (dd, *J* = 7.8, 1.1 Hz, 1H), 7.76 (t, *J* = 8.1 Hz, 1H), 7.38 (dd, *J* = 8.6, 1.1 Hz, 1H), 5.43 (d, *J* = 3.5 Hz, 1H), 5.29 (dd, *J* = 4.1, 2.2 Hz, 1H), 5.03 – 4.81 (m, 3H), 4.08 (s, 3H), 3.98 (tt, *J* = 6.5, 3.6 Hz, 1H), 3.67 (s, 1H), 3.19 (dd, *J* = 18.9, 1.9 Hz, 1H), 2.99 (d, *J* = 18.9 Hz, 1H), 2.38 (dt, *J* = 14.8, 2.2 Hz, 1H), 2.22 – 1.96 (m, 2H), 1.75 (tt, *J* = 13.4, 3.6 Hz, 1H), 1.61 (dd, *J* = 13.7, 3.9 Hz, 1H), 1.56 – 1.40 (m, 1H), 1.20 (d, *J* = 6.4 Hz, 3H), 0.97 (d, *J* = 7.2 Hz, 18H), 0.63 (q, *J* = 7.9 Hz, 6H), 0.14 (d, *J* = 2.0 Hz, 6H). ^{13}C NMR (101 MHz, $CDCl_3$) δ 211.6, 187.1, 186.7, 161.1, 156.5, 156.0, 135.7, 135.6, 134.5, 134.2, 121.0, 119.9, 118.5, 111.5, 111.3, 101.0, 69.3, 68.4, 67.6, 66.8, 56.8, 35.6, 34.1, 26.4, 26.0, 23.5, 18.7, 17.6, 7.1, 5.0, -5.3. HRMS: (M + Na)$^+$ calculated for $C_{39}H_{56}O_{11}Si_2Na$ 779.3259; found 779.3276.

3'-Desaminodoxorubicin (12)

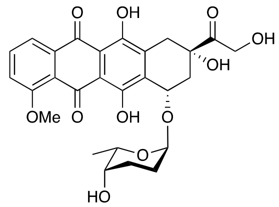

S15 (21 mg, 28 µmol) was dissolved in THF/pyr (3 mL, 2:1 v/v), to which HF·pyr complex (356 µL) was added at 0°C. After stirring for 1 hour, it was poured into sat. aq. $NaHCO_3$, extracted with DCM twice, dried over Na_2SO_4 and concentrated *in vacuo*. Column chromatography on neutral silica (20:80 acetone:toluene) gave the title compound as a red solid (14 mg, 26 µmol, 93%). ^{1}H NMR (400 MHz, Chloroform-*d*) δ 13.95 (s, 1H), 13.23 (s, 1H), 8.02 (dd, *J* = 7.8, 1.1 Hz, 1H), 7.88 – 7.71 (m, 1H), 7.40 (dd, *J* = 8.5, 1.2 Hz, 1H), 5.45 (d, *J* = 3.8 Hz, 1H), 5.34 (dd, *J* = 3.9, 2.2 Hz, 1H), 4.97 (s, 1H), 4.77 (s, 2H), 4.19 – 4.02 (m, 4H), 3.67 (s, 1H), 3.32 – 3.17 (m, 1H), 3.10 – 2.93 (m, 2H), 2.38 (dt, *J* = 14.6, 2.2 Hz, 1H), 2.16 (dd, *J* = 14.6, 4.0 Hz, 1H), 2.01 (tdd, *J* = 11.4, 8.5, 5.0 Hz, 1H), 1.78 (dq, *J* = 10.3, 3.4 Hz, 2H), 1.55 (dd, *J* = 14.3, 3.7 Hz, 1H), 1.26 (d, *J* = 6.7 Hz, 3H). ^{13}C NMR (101 MHz, $CDCl_3$) δ 214.0, 187.2, 186.8, 161.2, 156.4, 155.8, 135.9, 135.6, 134.1, 133.8, 121.0, 119.9, 118.6, 111.6, 111.5, 100.8, 69.2, 67.7, 67.1, 65.6, 56.8, 35.6, 34.2, 25.7, 23.2, 17.3. HRMS: $(M + Na)^+$ calculated for $C_{27}H_{28}O_{11}Na$ 551.1529; found 551.1533.

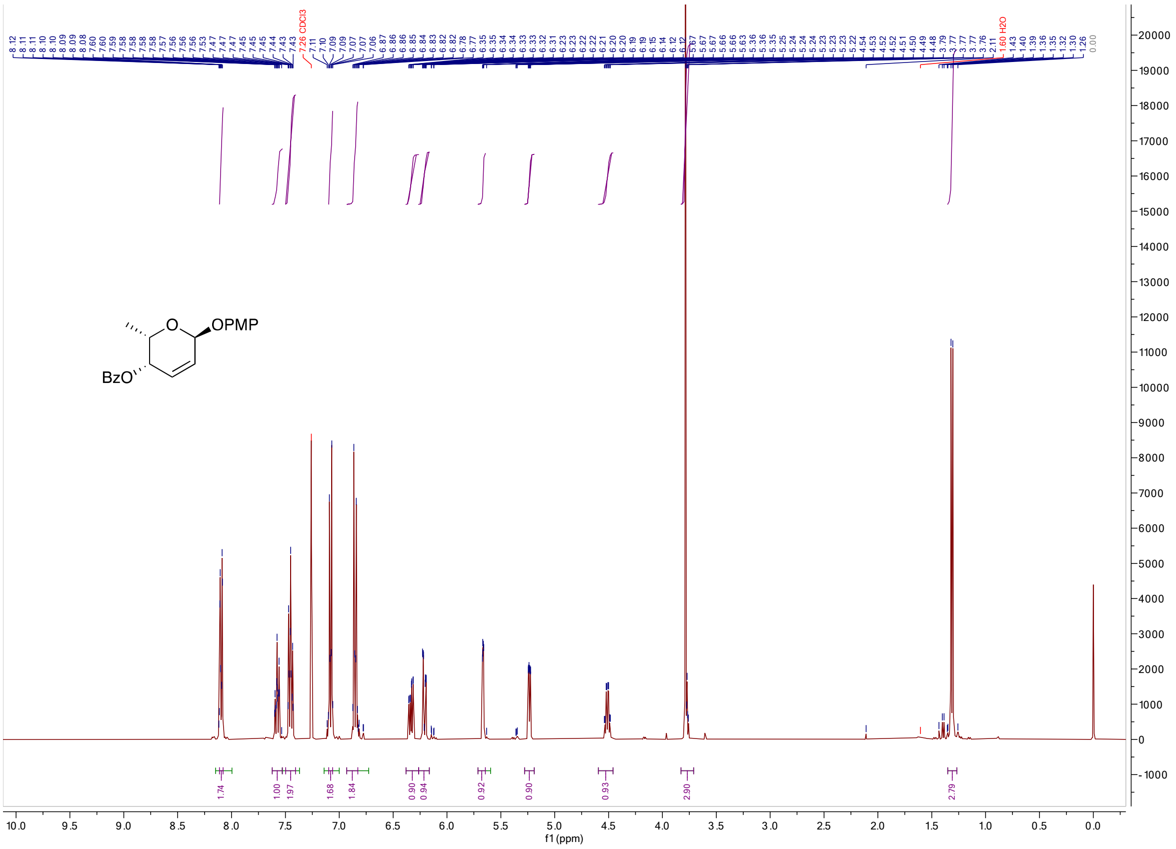
O
OPMP
BzO
1.74
1.00
1.97
1.68
1.84
0.90
0.94
0.92
0.90
0.93
2.90
2.79
f1 (ppm)

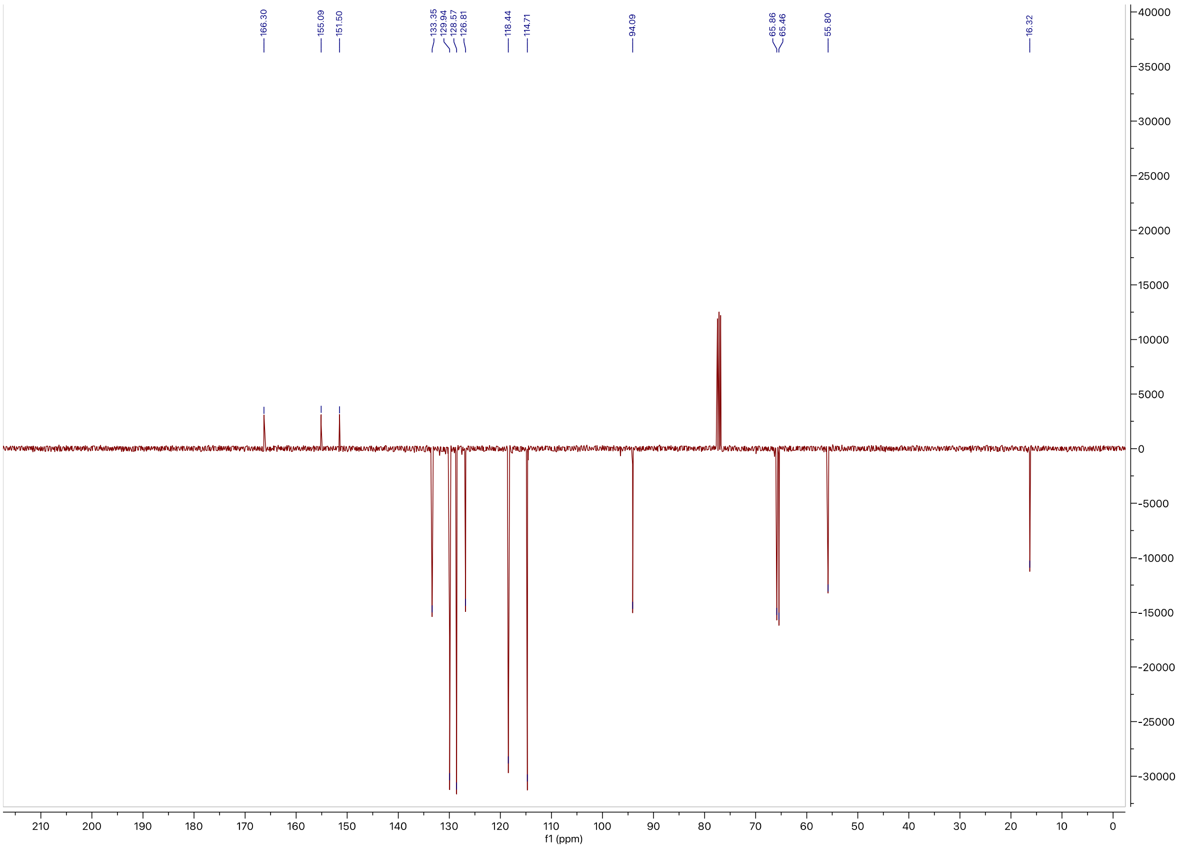
166.30
155.09
151.50
133.35
129.94
128.57
126.81
118.44
114.71
94.09
65.86
65.46
55.80
16.32
f1 (ppm)

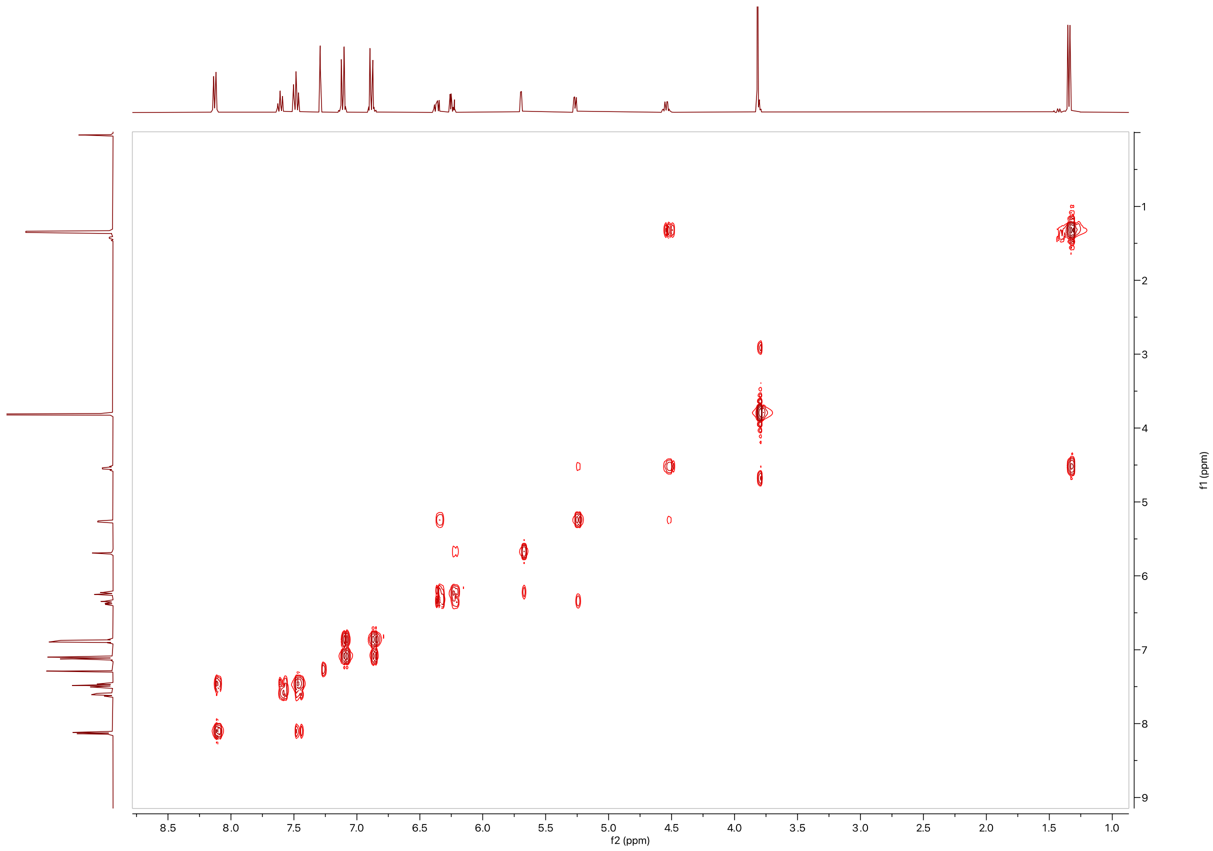
f2 (ppm)
f1 (ppm)

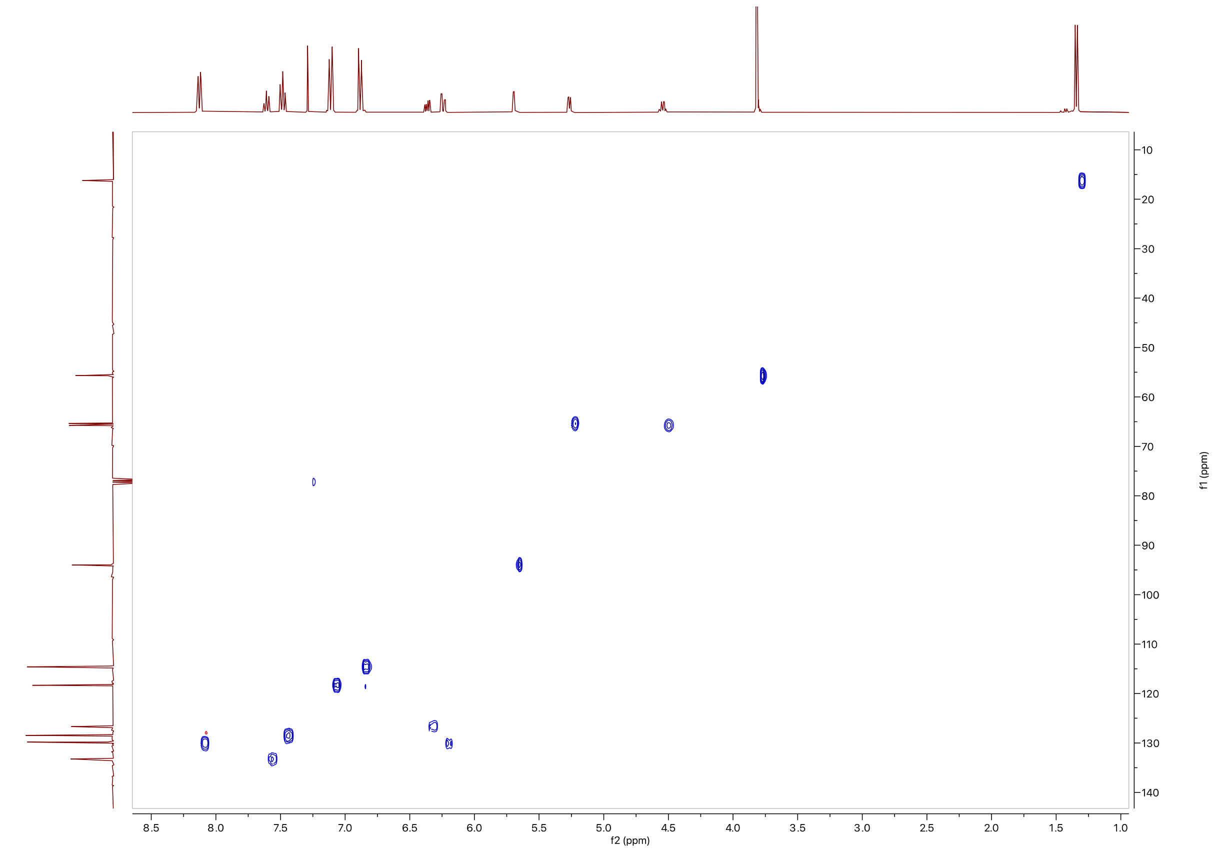
f2 (ppm)
f1 (ppm)

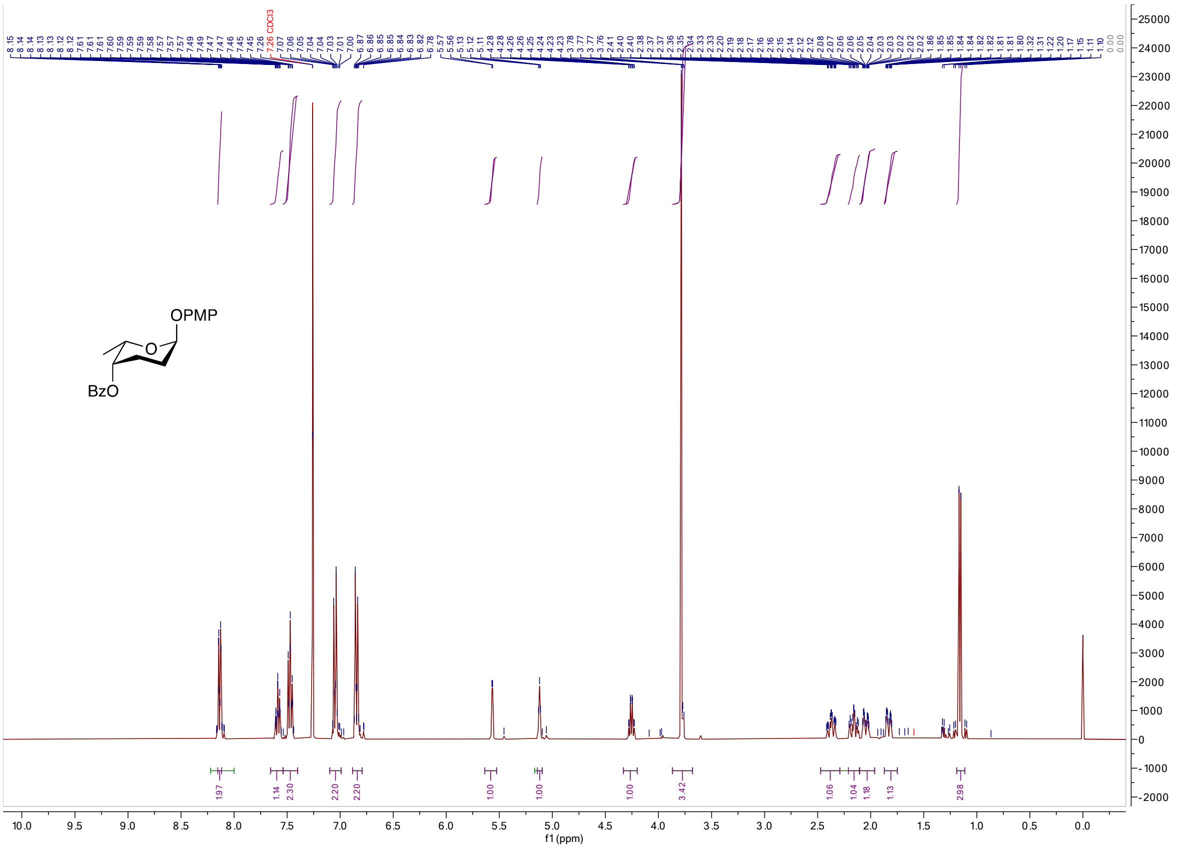
OPMP
O
BzO
7.26 CDCl3
1.97
1.14
2.30
2.20
2.20
1.00
1.00
1.00
3.42
1.06
1.04
1.18
1.13
2.98
f1 (ppm)

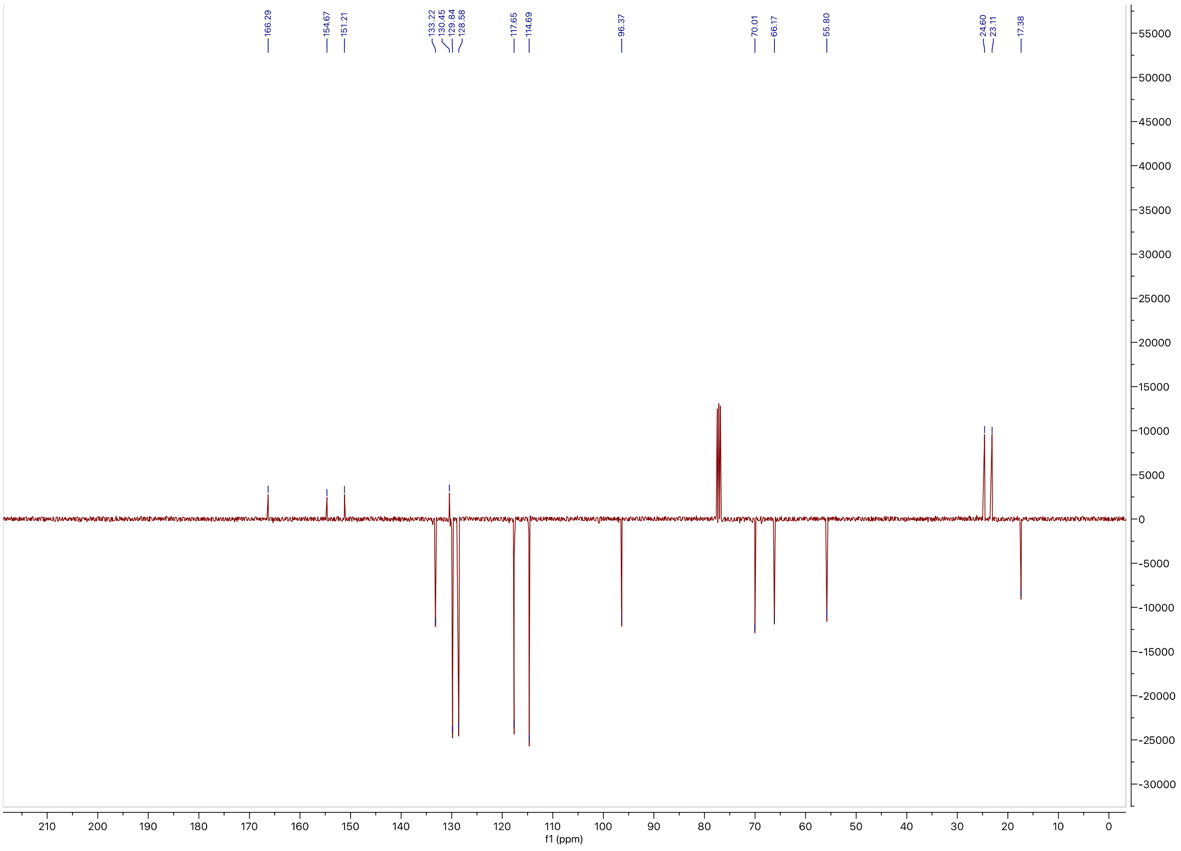
166.29
154.67
151.21
133.22
130.46
129.84
128.58
117.65
114.69
96.37
70.01
66.17
55.80
24.60
23.11
17.38
f1 (ppm)

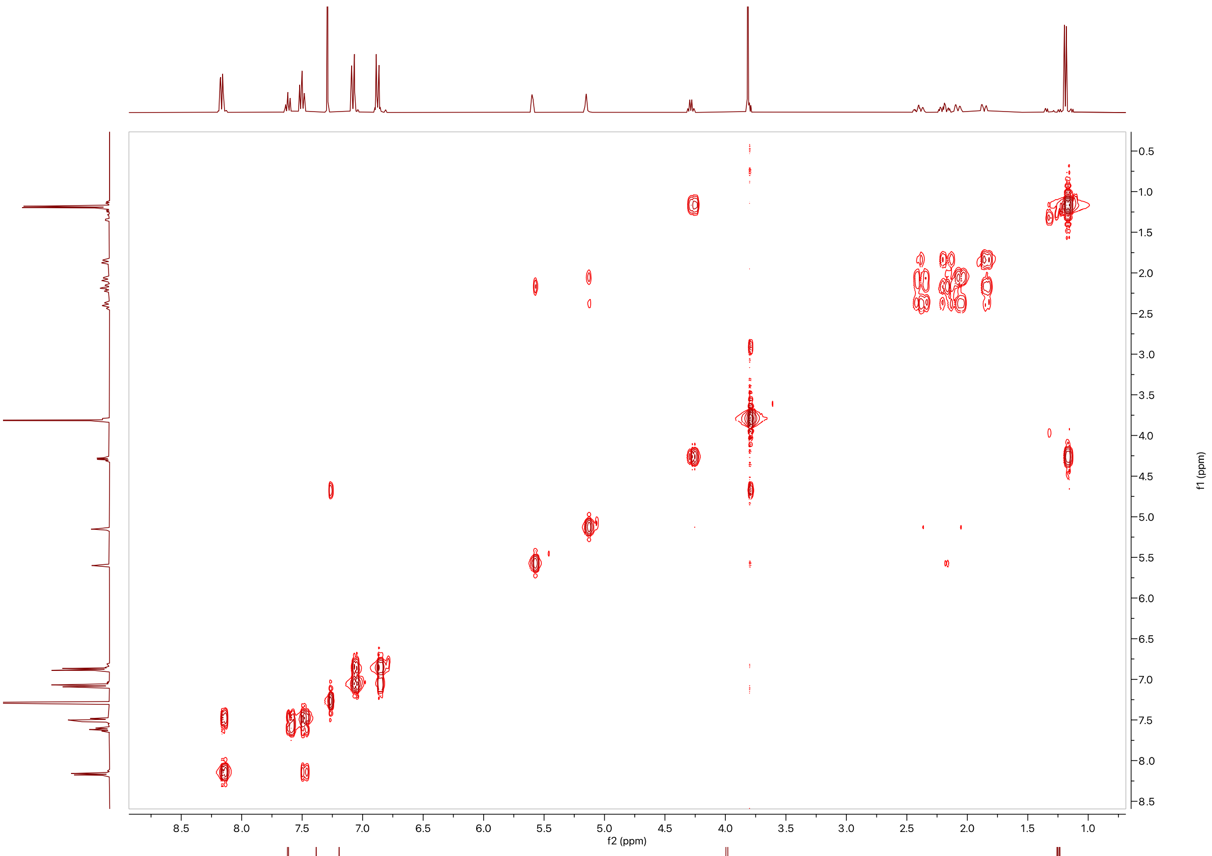
f2 (ppm)
f1 (ppm)

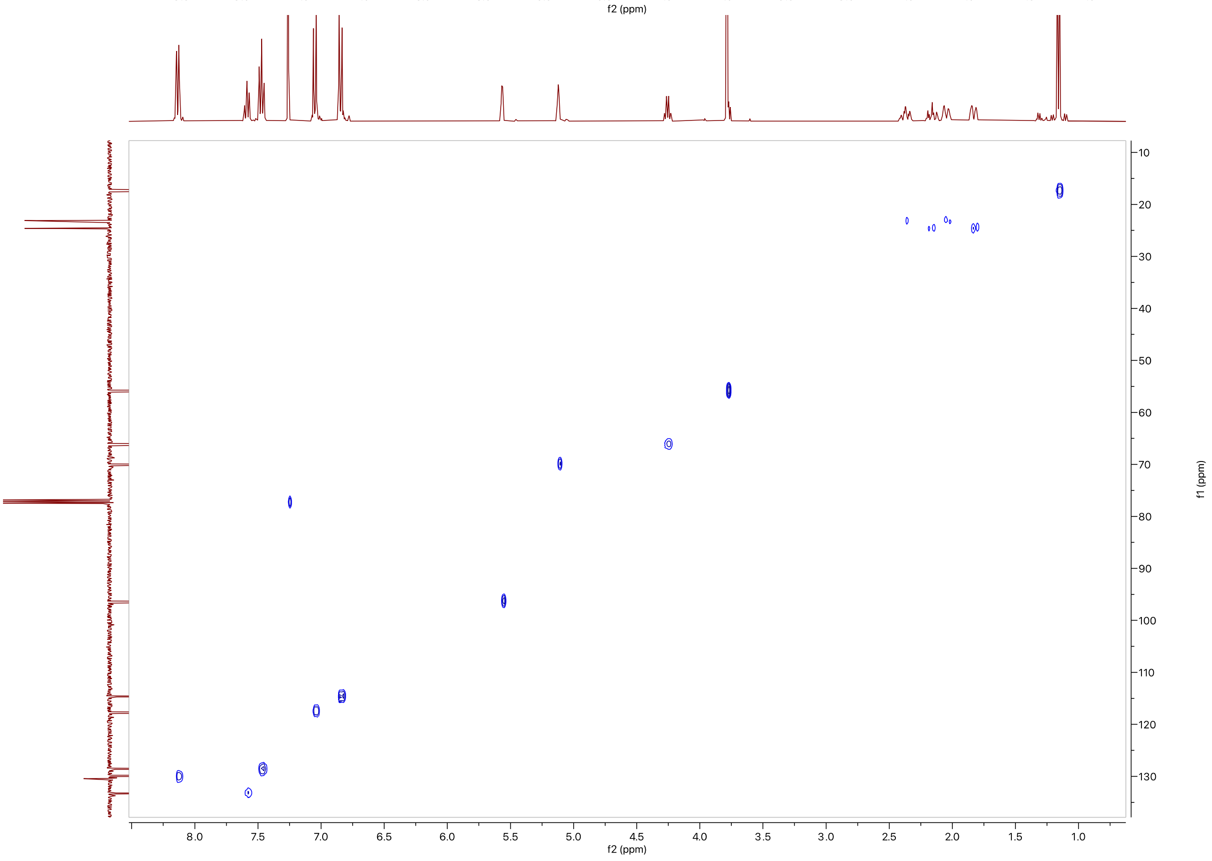
f2 (ppm)
f1 (ppm)

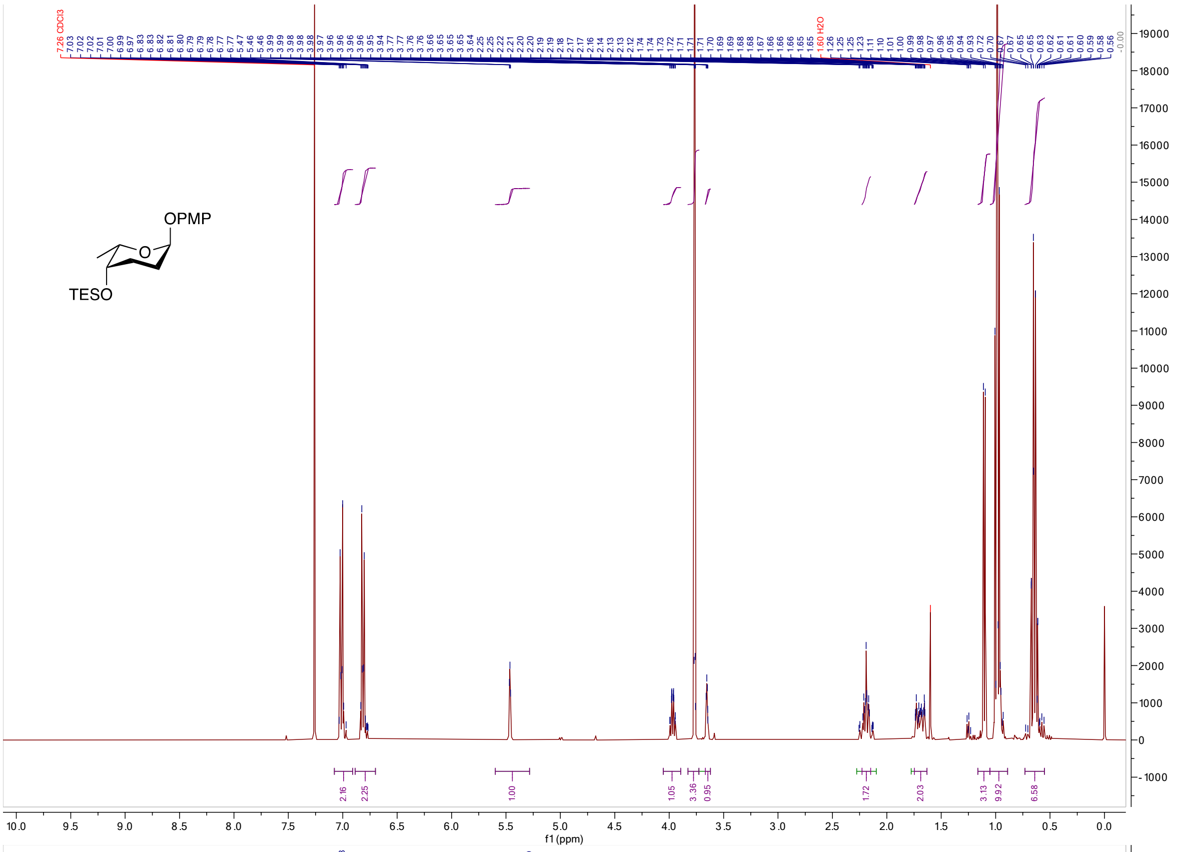
OPMP
O
TESO
7.26 CDCl3
1.60 H2O
2.16
2.25
1.00
1.05
3.36
0.95
1.72
2.03
3.13
9.92
6.58
f1 (ppm)

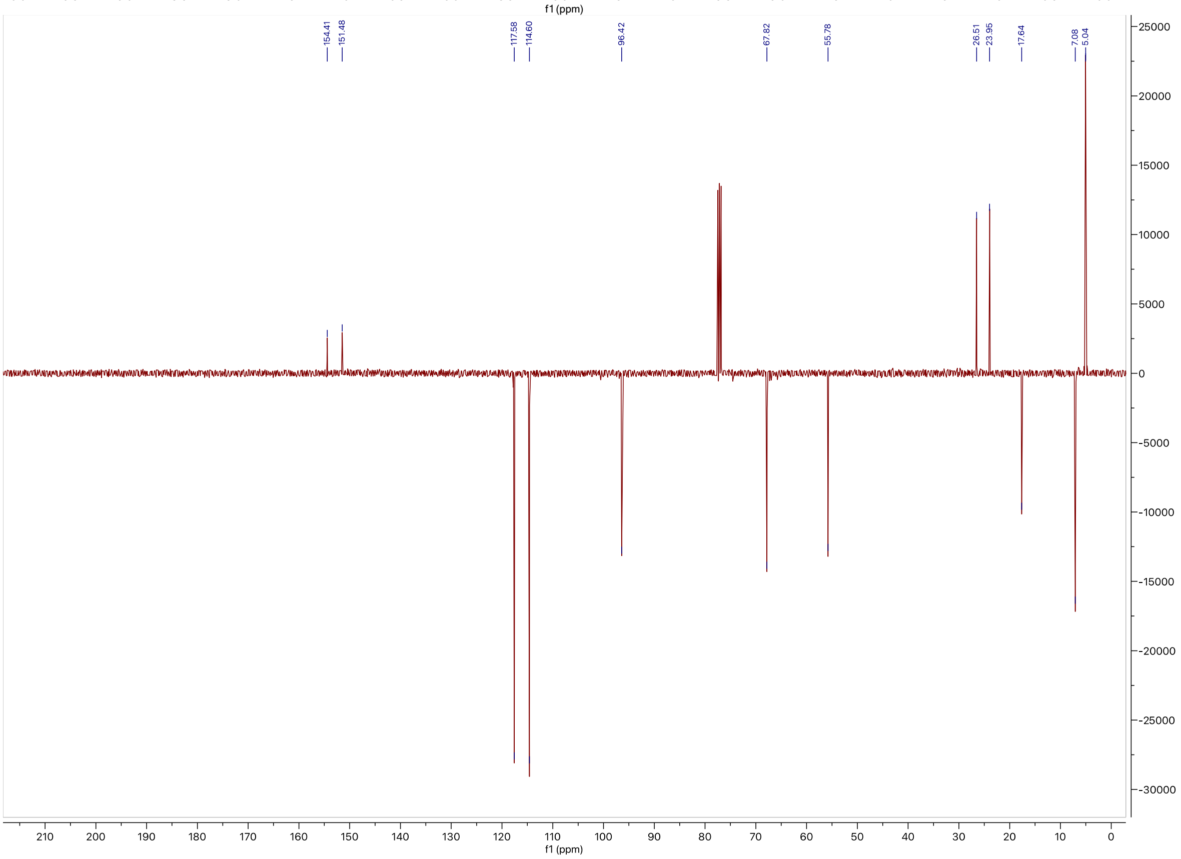
154.41
151.48
117.58
114.60
96.42
67.82
55.78
26.61
23.95
17.64
7.08
5.04
f1 (ppm)

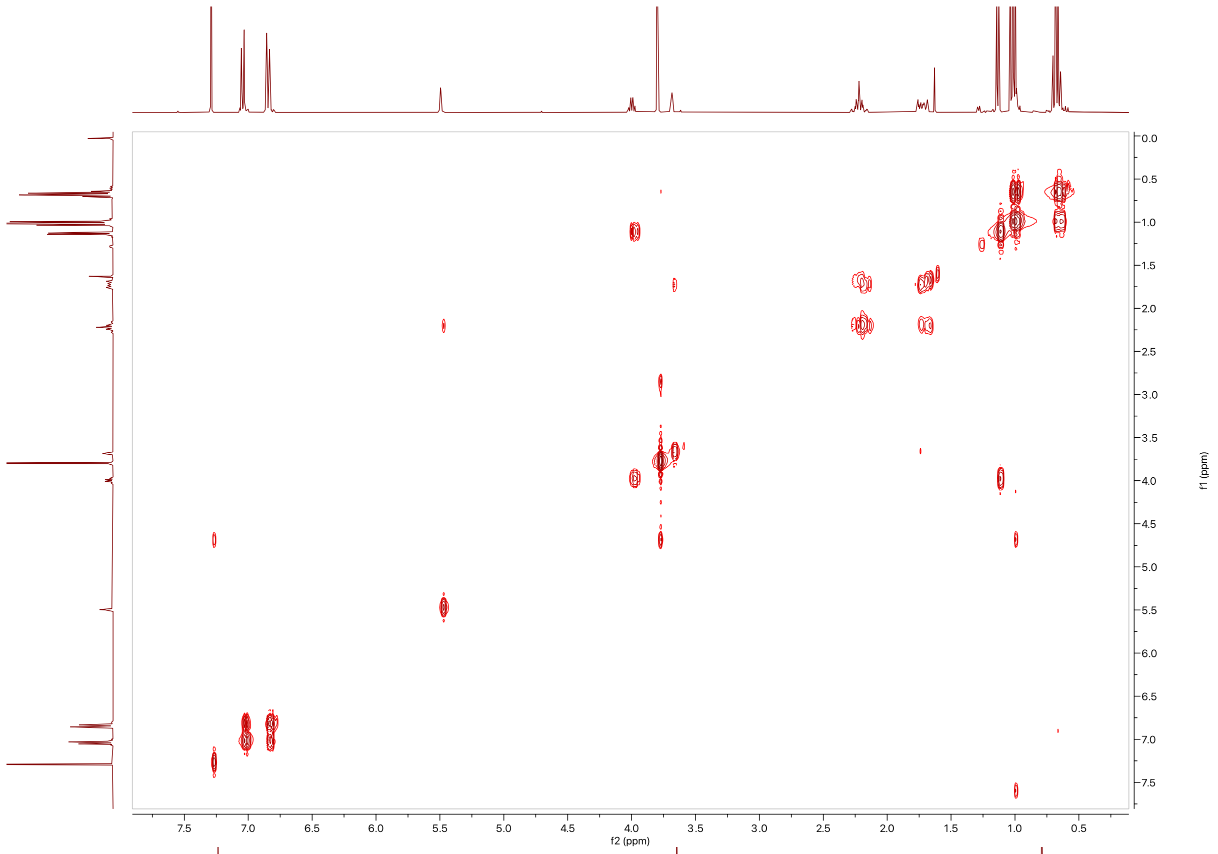
f2 (ppm)
f1 (ppm)

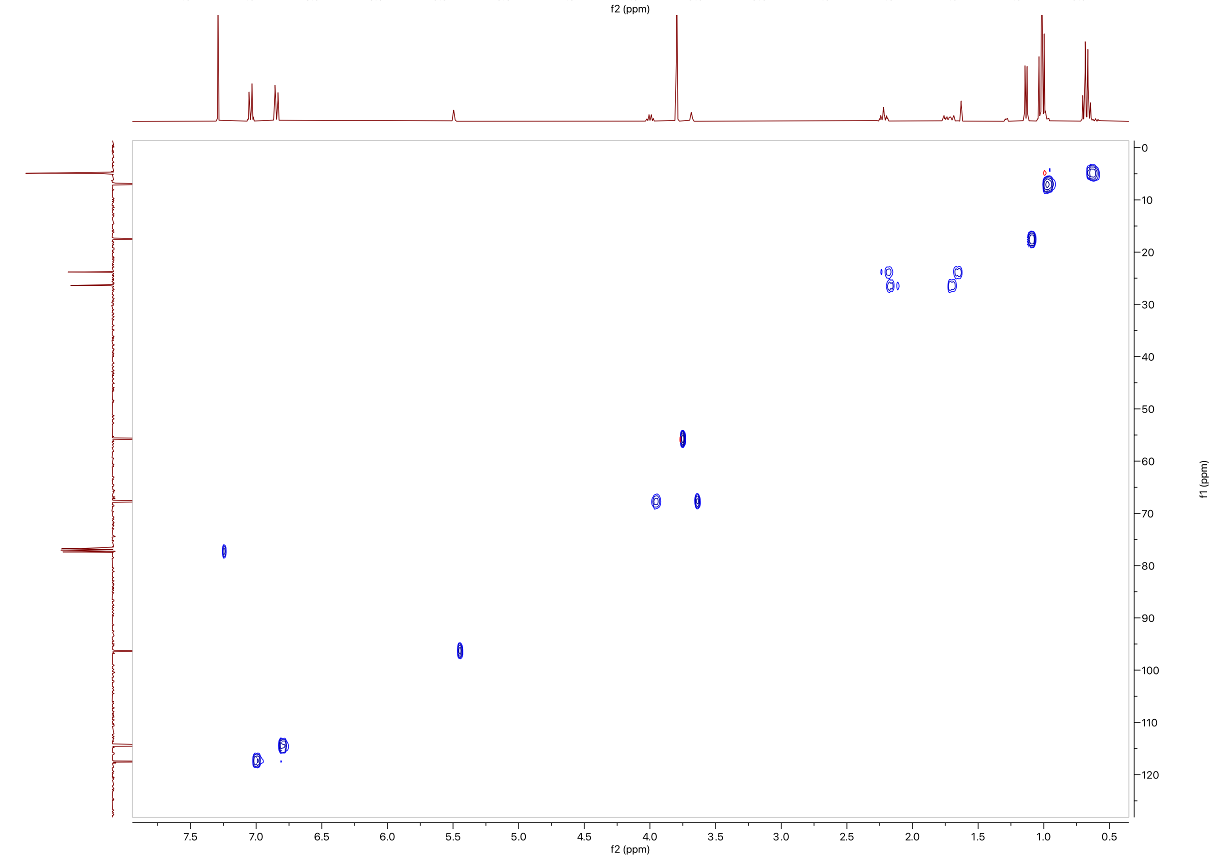
f2 (ppm)
f1 (ppm)

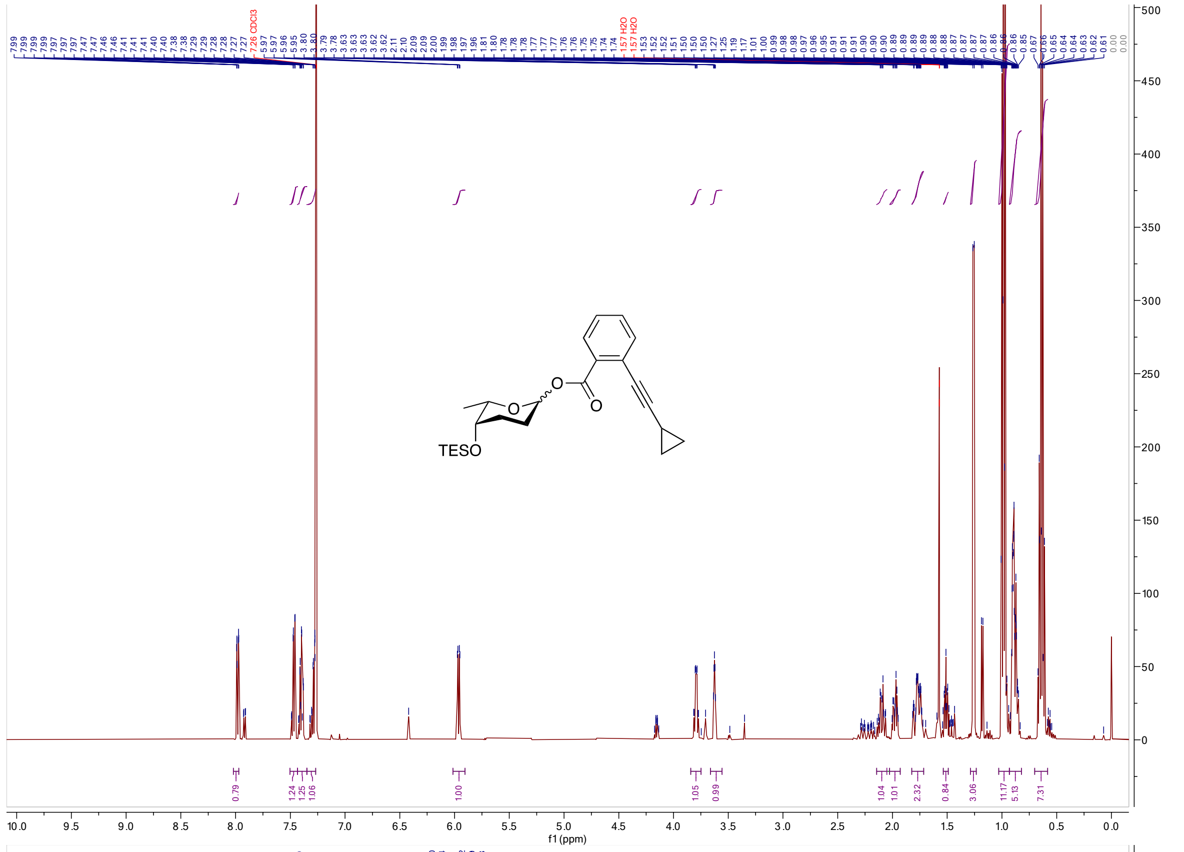

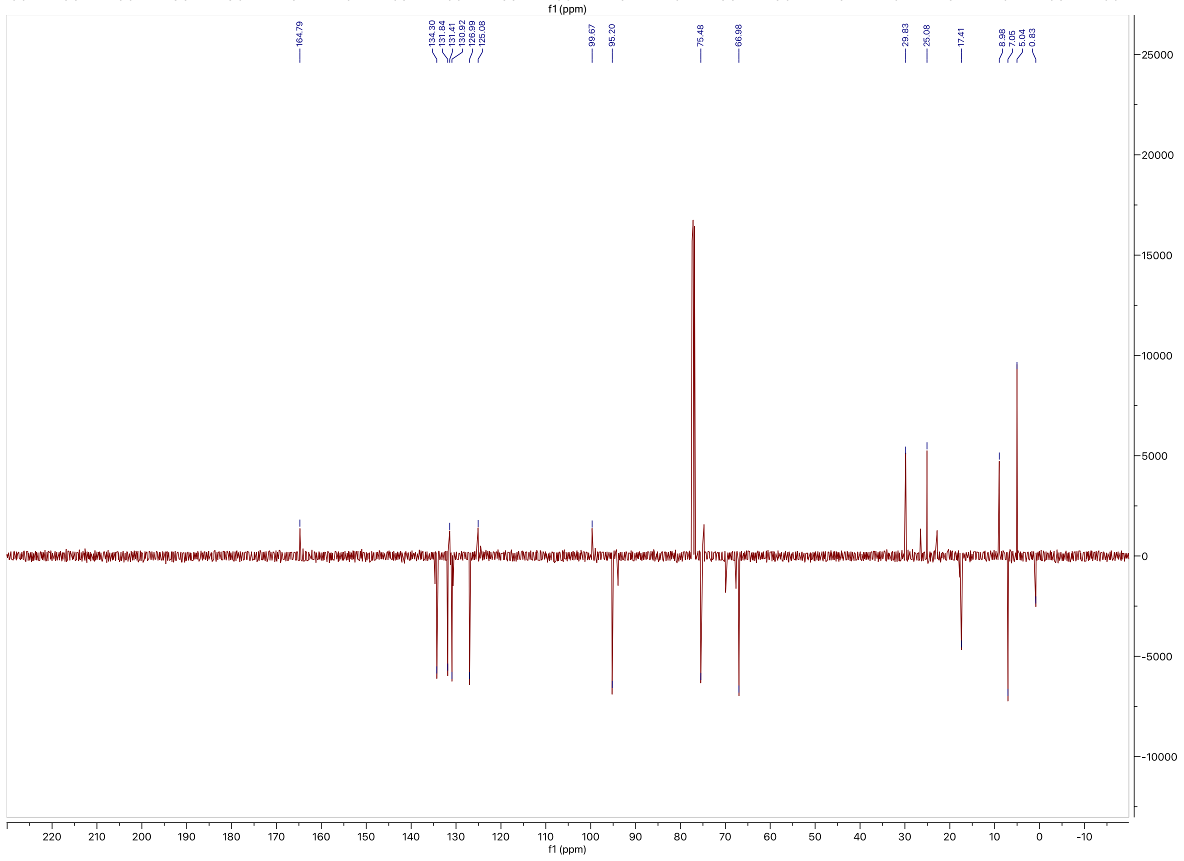

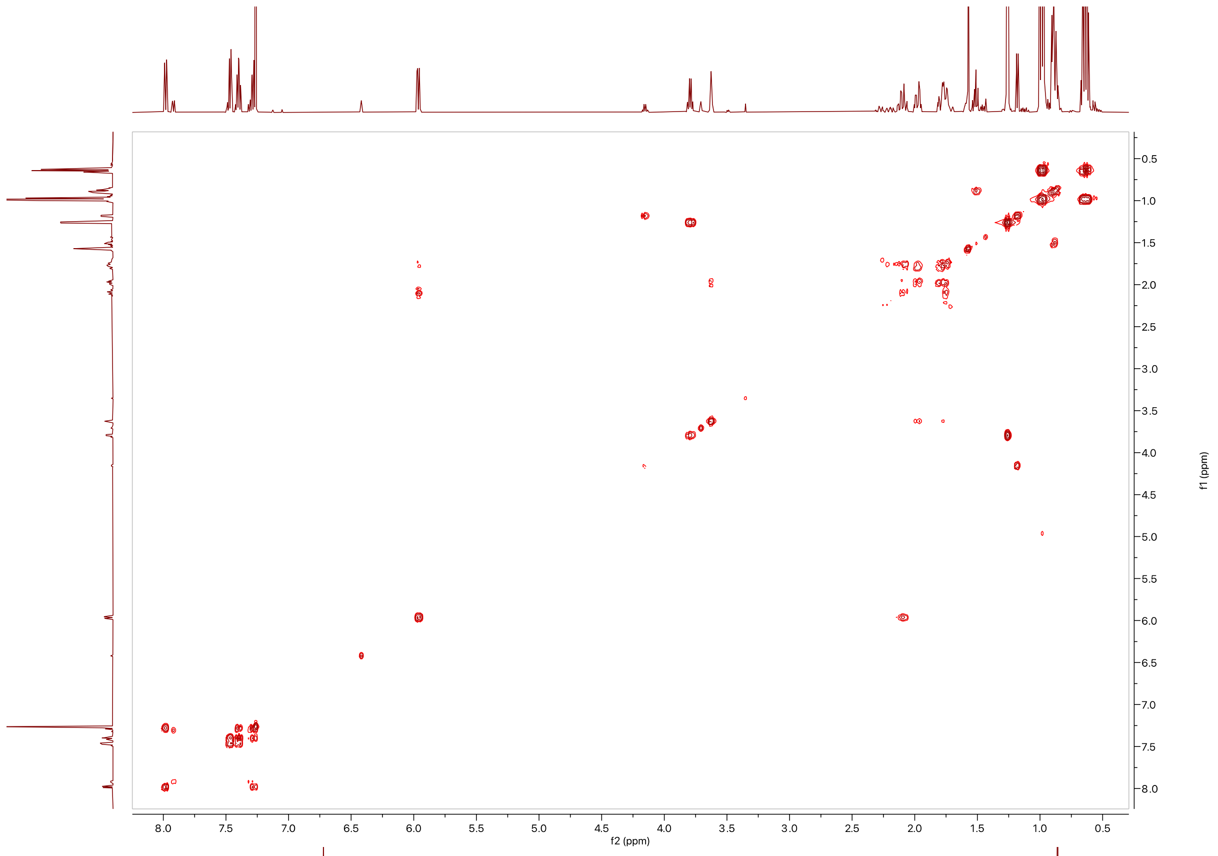
f2 (ppm)
f1 (ppm)

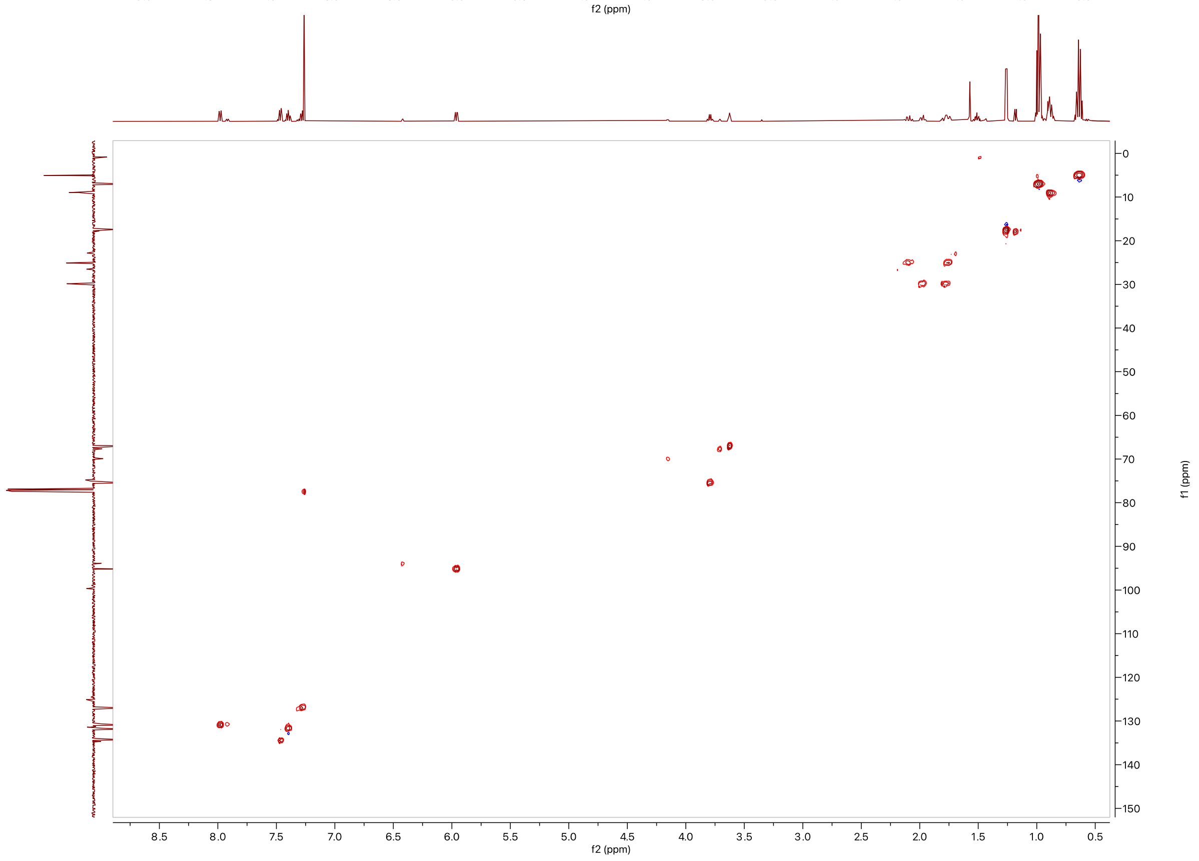
f2 (ppm)
f1 (ppm)

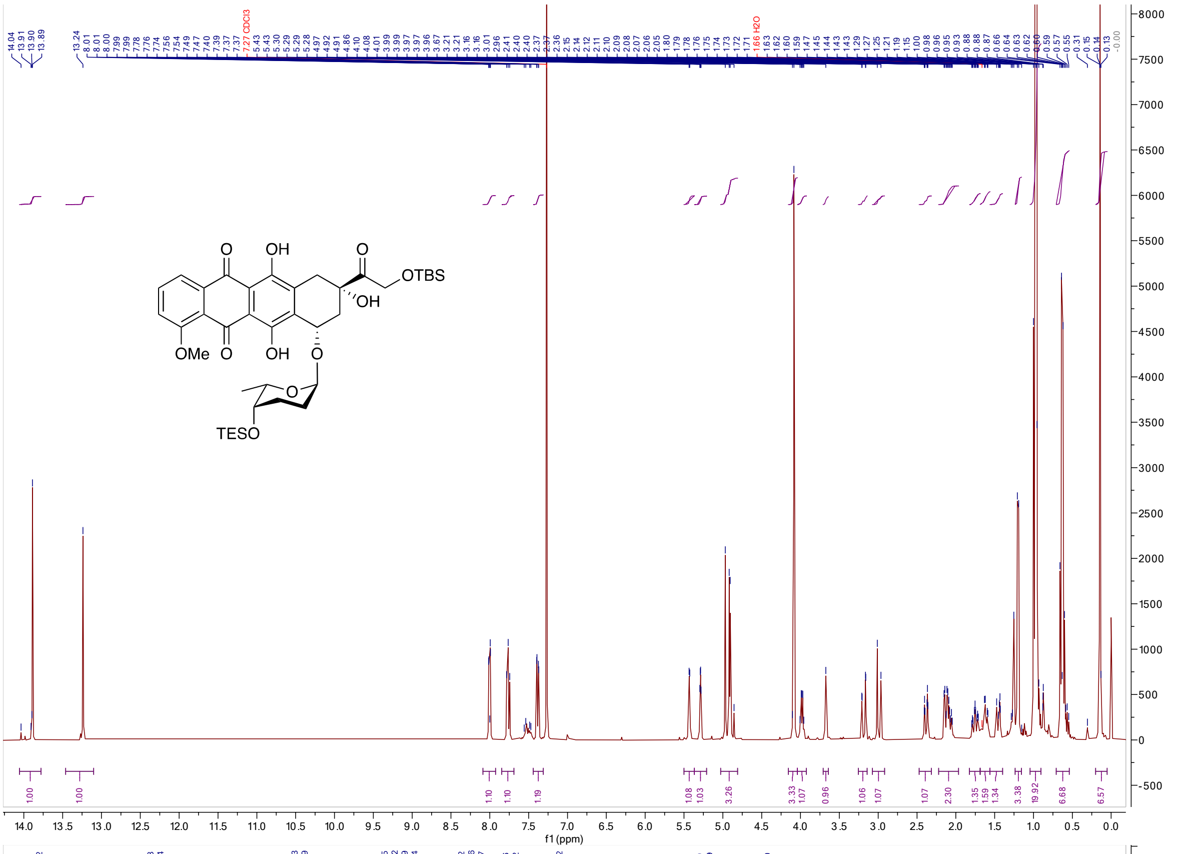
OTBS
OH
OMe
TESO
f1 (ppm)

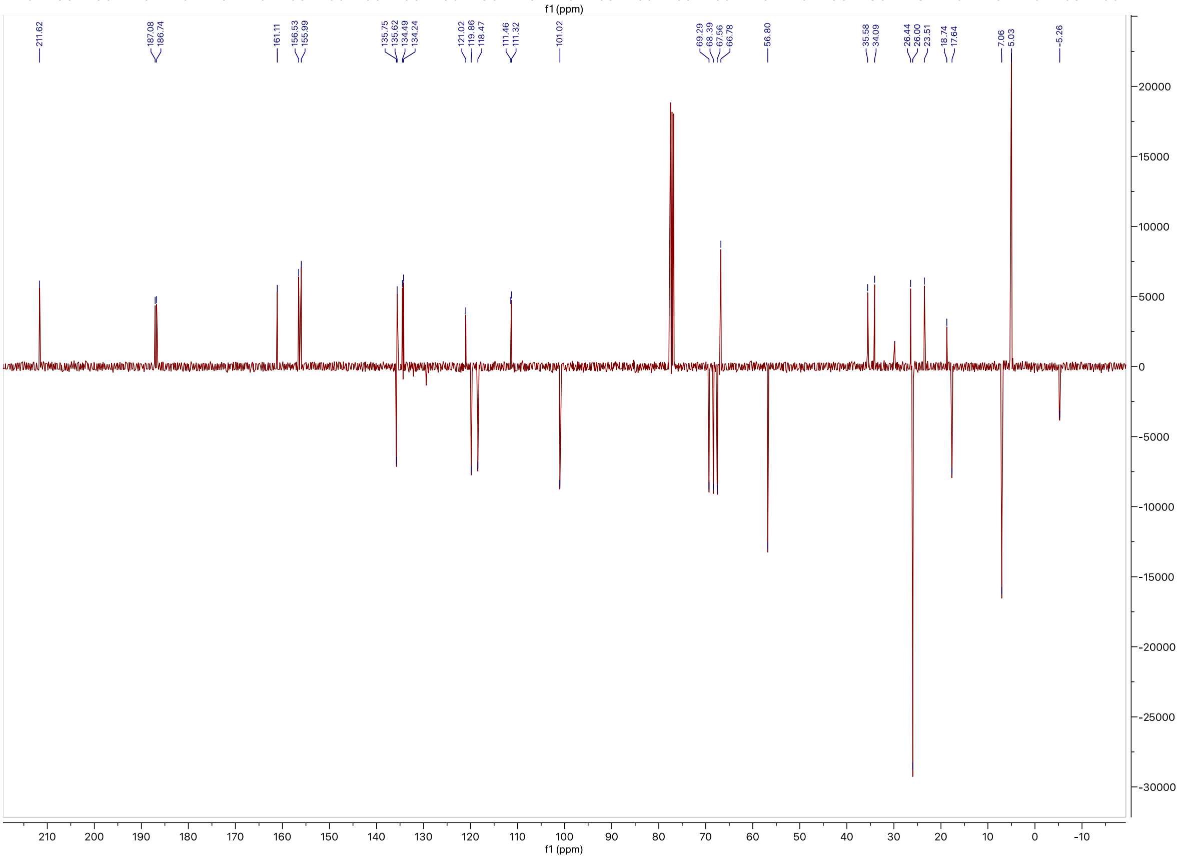
f1 (ppm)

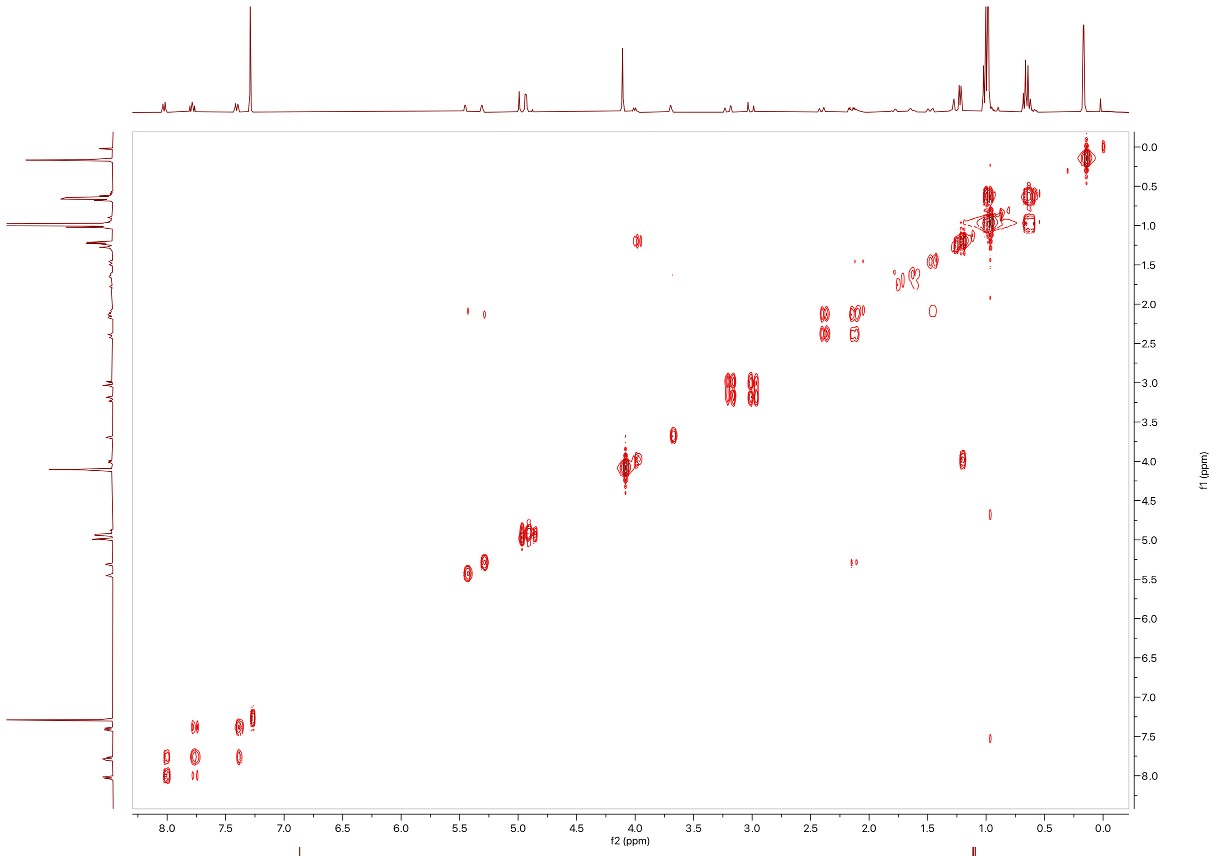
f2 (ppm)
f1 (ppm)

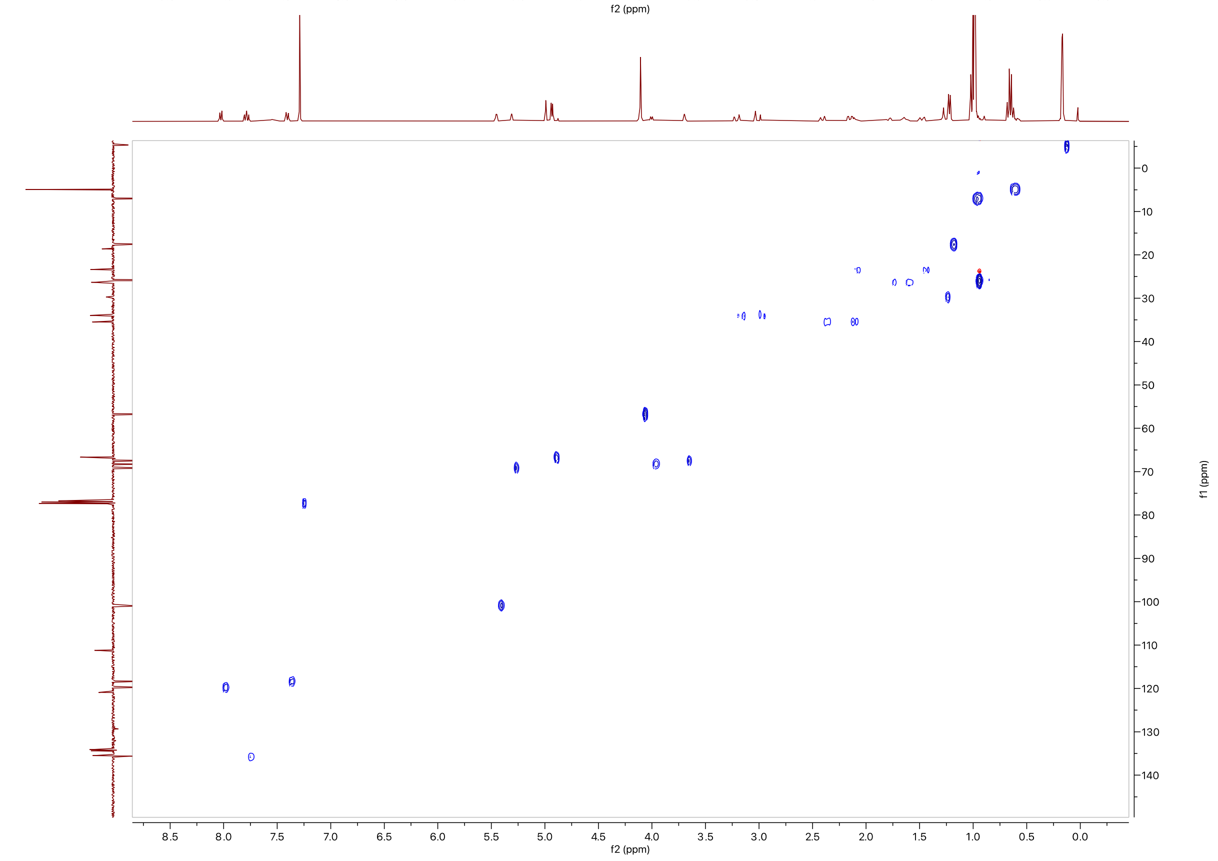
f2 (ppm)
f1 (ppm)

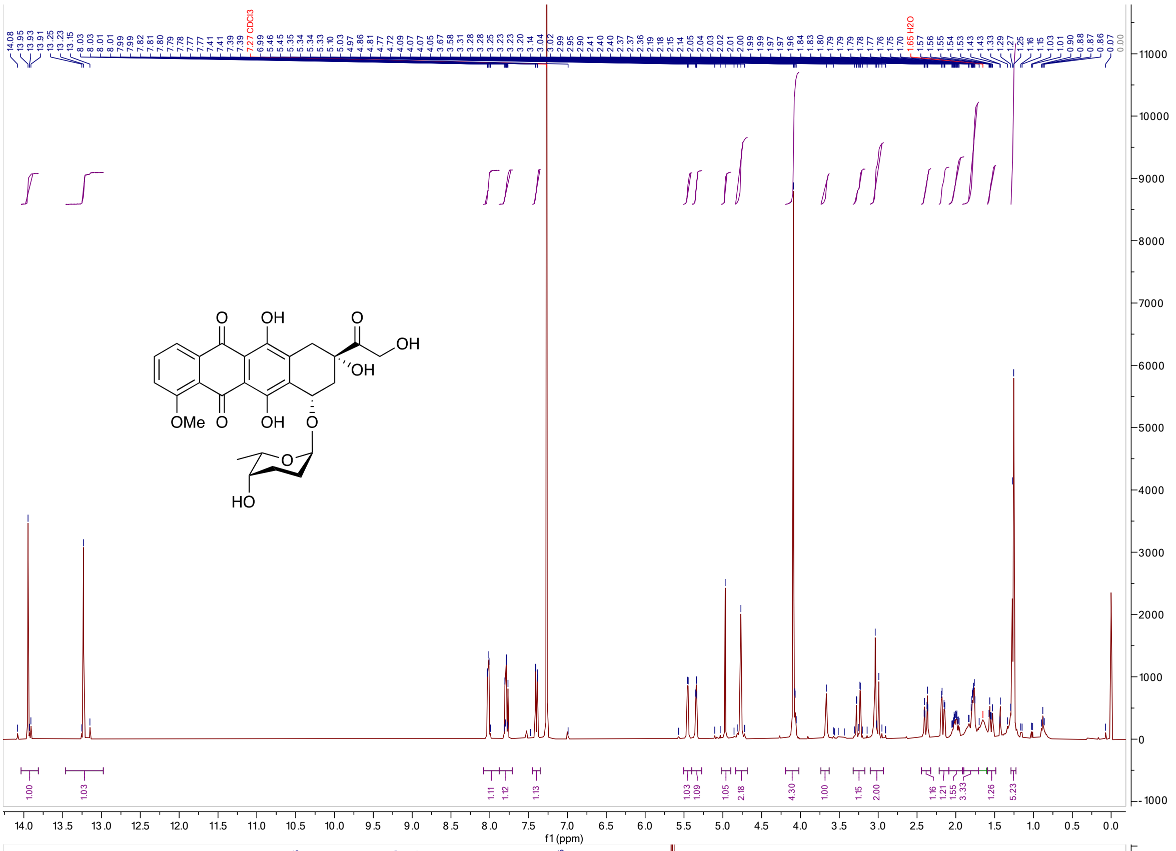
f1 (ppm)

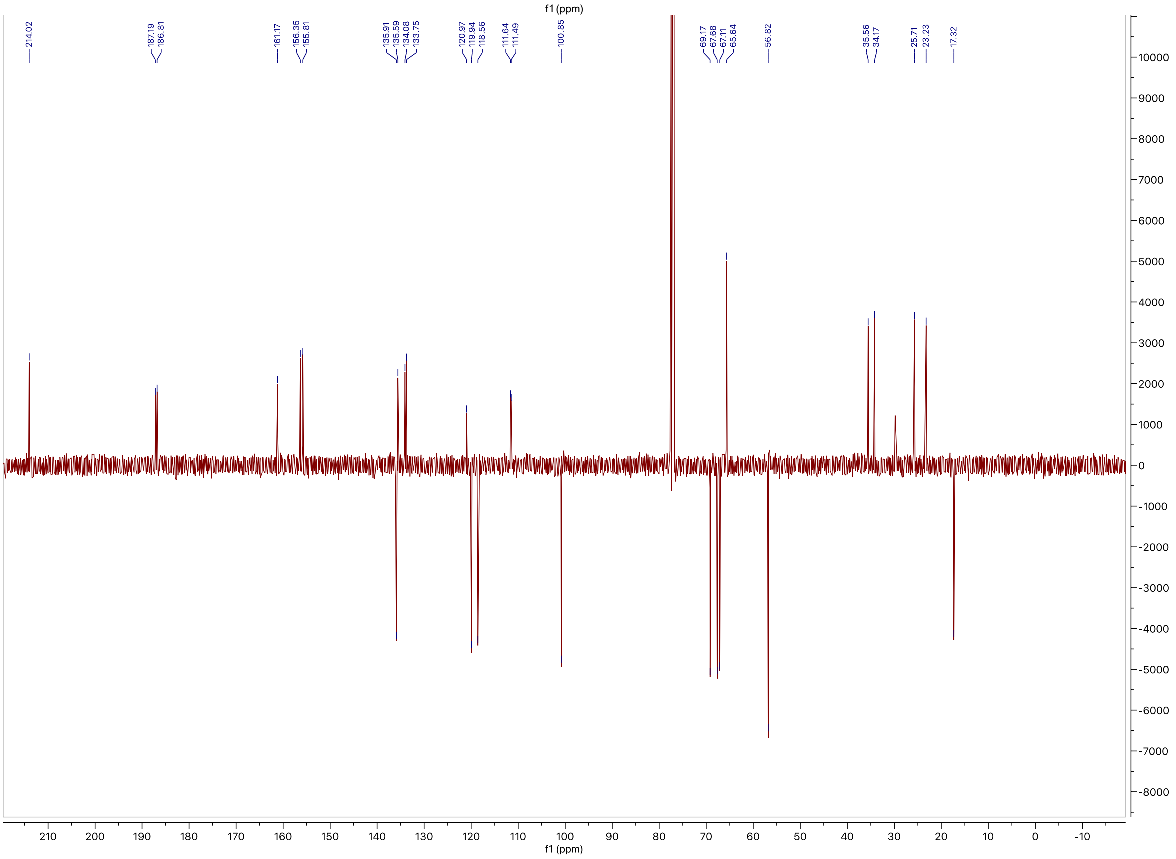
f1 (ppm)

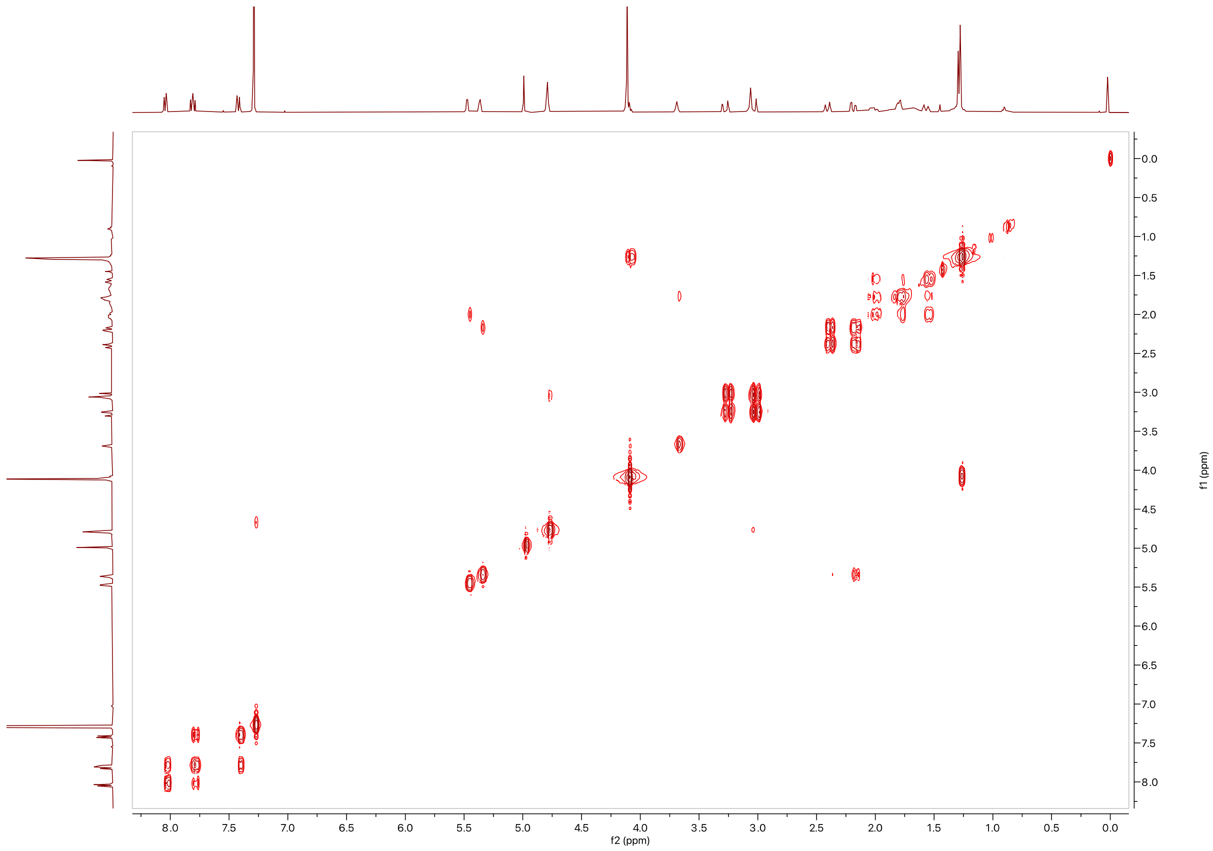
f2 (ppm)
f1 (ppm)

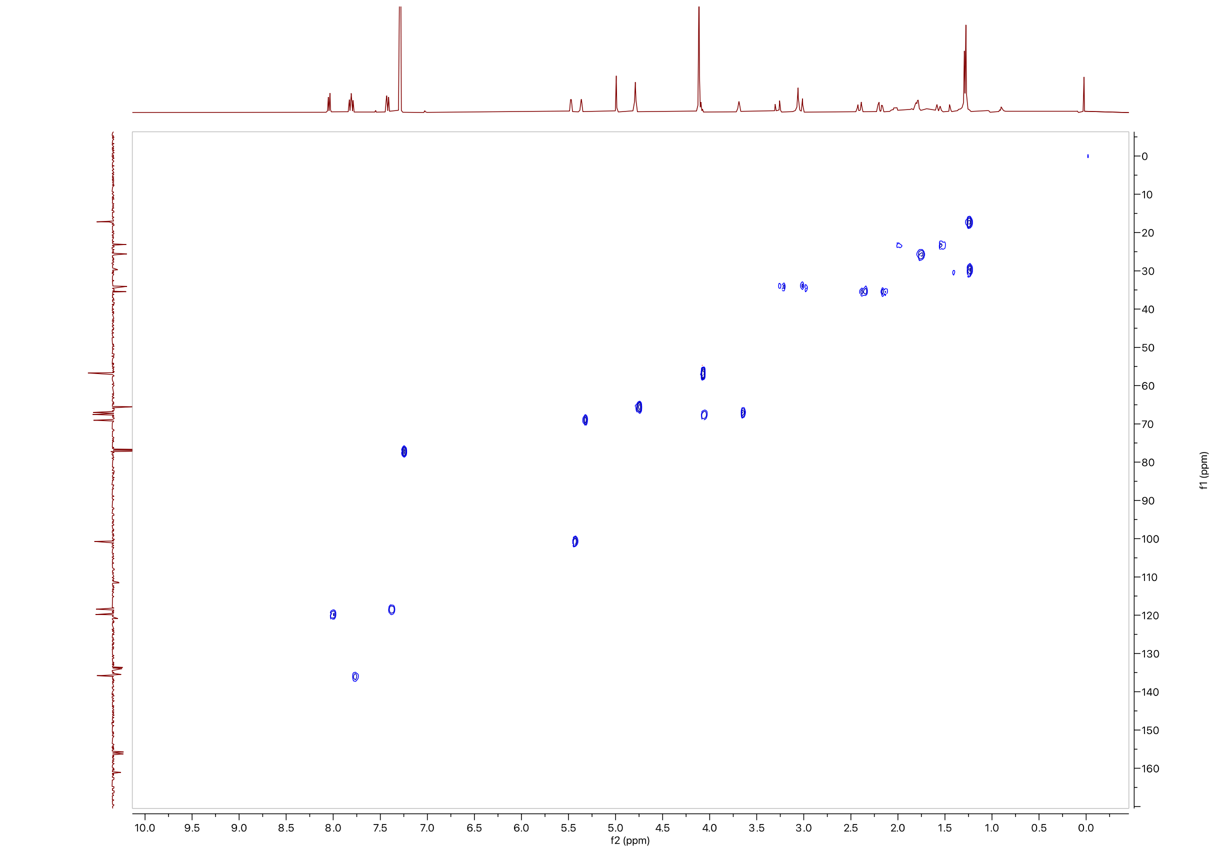
f2 (ppm)
f1 (ppm)

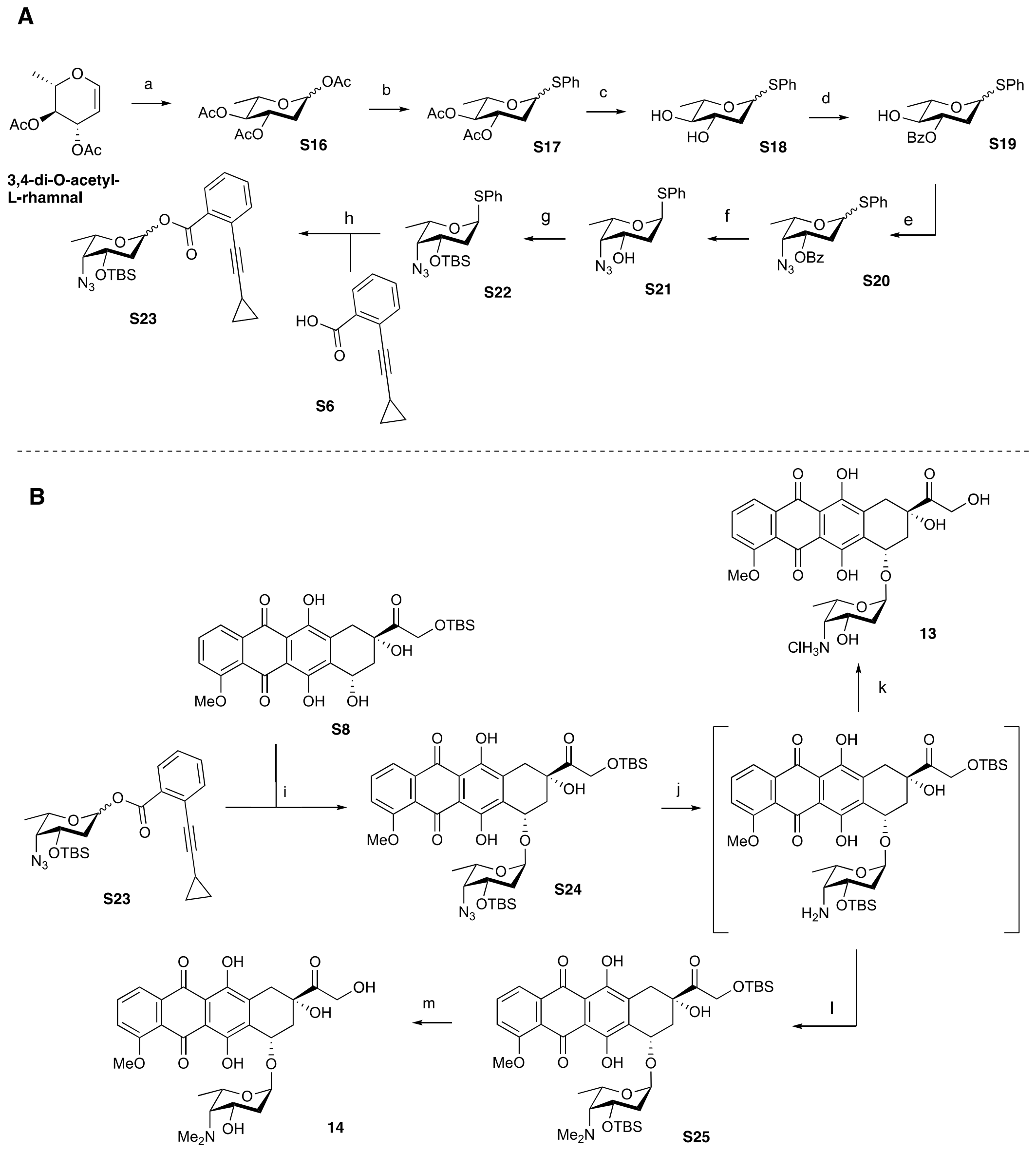

Scheme S3. Synthesis of 3,4-*iso*-doxorubicins **13** and **14**. (A) Synthesis of alkynylbenzoate donor **S23**; (B) Glycosylation, *N,N*-dimethylation and deprotections. *Reagents and conditions:* (a) AcOH, PPh_3·HBr, DCM, 51% (5:1 α:β); (b) PhSH, BF_3·Et_2O, DCM, quant.; (c) NaOMe, MeOH, 88%; (d) benzoyl chloride, Bu_2SnCl_2, DIPEA, THF, 84%; (e) *i.* triflic anhydride, pyr., 0 °C, *ii.* NaN_3, DMF, 68% over 2 steps; (f) NaOMe, MeOH, 86%; (g) TBS-Cl, imidazole, DMF, 94%; (h) *i.* $AgNO_3$, 2,6-lutidine, THF/H_2O; *ii.* EDCI·HCl, DMAP, DIPEA, DCM, 65% over 2 steps (1:3 α:β); (i) PPh_3AuNTf_2, DCM, 91% (>20:1 α:β); (j) PPh_3, THF, H_2O, 50 °C; (k) *i.* HF·pyr., pyr., 0 °C; *ii.* lyophilization from aq. HCl, 36% over 3 steps for **9**; (l) NaBH(OAc_3), aq. CH_2O, EtOH, 43% over 2 steps; (m) HF·pyr., pyr., 0 °C, 72%.

1,3,4-Tri-*O*-acetyl-2-deoxy-L-rhamnopyranoside (S16)[12]

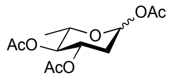

To a solution of 3,4-di-*O*-acetyl-L-rhamnal (9.37 g, 54.0 mmol) in DCM (300 mL) were added acetic acid (5.10 mL, 5.39 g, 86.0 mmol, 1.6 eq) and PPh_3·HBr (2.20 g, 3.26 mmol, 0.1 eq). The reaction mixture was stirred for two days and concentrated *in vacuo.* Column chromatography (25:75 – 30:70 Et_2O:pentane) afforded the title compound as a white

solid (7.63 g, 26.6 mmol, 51%, 5:1 α:β). Spectral data of the title compound was in accordance with that of literary precedence.[12]

Phenyl-3,4-di-*O*-acetyl-2-deoxy-1-thio-L-rhamnopyranoside (S17)

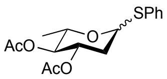

To a solution of **S16** (7.6 g, 28 mmol) in DCM (200 mL) were added thiophenol (3.7 mL, 33 mmol, 1.2 eq) and $BF_3 \cdot OEt_2$ (4.9 mL, 33 mmol, 1.2 eq). The reaction mixture was stirred for 1 h, diluted with DCM and washed with sat. aq. $NaHCO_3$, 1M NaOH and brine. The organic layer was dried over Na_2SO_4 and concentrated *in vacuo* to obtain the title compound as a colourless oil (8.9 g, 28 mmol, quant., 1:1 α:β). Spectral data of the title compound was in accordance with that of literary precedence. [12]

Phenyl-2-deoxy-1-thio-L-rhamnopyranoside (S18)

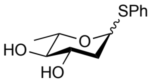

To a solution of **S17** (9.9 g, 30 mmol) in MeOH (250 mL) was added sodium methoxide (330 mg, 6.11 mmol, 0.2 eq). After stirring for 2h, the reaction mixture was quenched by addition of dry ice. Solvent was removed *in vacuo* and the residue was coevaporated thrice with toluene. Column chromatography (50:50 – 100:0 Et_2O:pentane) afforded the title compound as a sticky pale yellow syrup (6.30 g, 26.3 mmol, 88%, 1:1 α:β). Spectral data of the title compound was in accordance with that of literary precedence.[12]

Phenyl-3-*O*-benzoyl-2-deoxy-1-thio-L-rhamnopyranoside (S19)[12]

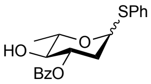

Diol **S18** (5.89 g, 24.5 mmol) and dibutyltin dichloride (365 mg, 1.20 mmol, 0.05 eq) were dissolved in THF (125 mL). Benzoyl chloride (3.4 mL, 29 mmol, 1.2 eq) was added and the reaction mixture was stirred overnight. The reaction mixture was quenched by addition of 1M HCl and was subsequently partitioned between EtOAc and 1M HCl. The organic layer was washed with sat. aq. $NaHCO_3$ solution and brine, dried over Na_2SO_4 and solvent was removed *in vacuo*. Purification by column chromatography (30:70 Et_2O:pentane) afforded the title compound as a sticky syrup (7.10 g, 20.5 mmol, 84%, 3:2 α:β). Spectral data of the title compound was in accordance with that of literary precedence.[12]

Phenyl-3-*O*-benzoyl-4-azido-2,4-deoxy-1-thio-L-fucopyranoside (S20)

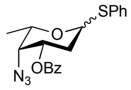

To a solution of **S19** (5.20 g, 15.3 mmol) in DCM (150 mL) were added pyridine (15 mL) and triflic anhydride (2.3 mL, 18 mmol, 1.2 eq) at 0˚C. After stirring at that temperature for 15 minutes, the reaction was diluted with DCM and washed successively with 1M aq. $CuSO_4$ and brine, dried over $MgSO_4$, filtered, and concentrated *in vacuo* at 0°C. The colorless oil thus obtained was dissolved in DMF (5 mL), to which NaN_3 (15.3 g, 37.5 mmol, 5 eq) was added, and the mixture was stirred overnight. The reaction mixture was then partitioned between H_2O and Et_2O, and the organic layer was washed with H_2O and brine and dried over $MgSO_4$. Solvent was removed *in vacuo* and purification by column chromatography (5:95 – 30:70 Et_2O:pentane) gave the title compound as a white solid (4.09 g, 11.1 mmol, 68%, 1:1 α:β). Spectral data for the α-anomer: ^{1}H NMR (400 MHz, Chloroform-*d*) δ 8.16 – 8.05 (m, 2H), 7.64 – 7.54 (m, 1H), 7.51 – 7.41 (m, 4H), 7.35 – 7.20 (m, 3H), 5.72 (d, J = 5.8 Hz, 1H), 5.60 (ddd, J = 12.3, 4.7, 3.2 Hz, 1H), 4.58 (qd, J = 6.4, 1.5 Hz, 1H), 3.94 (dt, J = 3.0, 1.3 Hz, 1H), 2.63 (td, J = 12.8, 5.9 Hz, 1H), 2.25 (ddt, J = 13.2, 4.8, 1.2 Hz, 1H), 1.31 (d, J = 6.4 Hz, 3H). ^{13}C NMR (101 MHz, $CDCl_3$) δ 165.7, 134.7, 133.7, 133.6, 131.1, 130.0, 129.3, 129.1, 128.7, 127.3, 83.7, 70.3, 66.1, 63.7, 30.9, 17.8. Spectral data for the β-anomer: ^{1}H NMR (400 MHz, Chloroform-*d*) δ 8.12 – 8.04 (m, 2H), 7.65 – 7.49 (m, 3H), 7.49 – 7.38 (m, 2H), 7.38 – 7.19 (m, 3H), 5.33 (ddd, J = 11.3, 5.4, 3.4 Hz, 1H), 4.81 (dd, J = 11.2, 2.9 Hz, 1H), 3.80 (dt, J = 3.5, 1.1 Hz, 1H), 3.71 (qd, J = 6.3, 1.3 Hz, 1H), 2.49 – 2.09 (m, 2H), 1.39 (d, J = 6.3 Hz, 3H). ^{13}C NMR (101 MHz, $CDCl_3$) δ

165.7, 133.7, 133.5, 132.0, 130.0, 129.2, 129.0, 128.6, 127.7, 82.4, 73.6, 72.8, 62.5, 31.5, 18.3. HRMS: $[M+Na]^+$ calculated for $C_{19}H_{20}O_4SNa$ 392.1039; found 392.1044.

Phenyl-4-azido-2,4-deoxy-1-thio-α-L-fucopyranoside (S21)

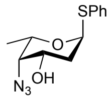

To a solution of **S20** (1.94 g, 5.25 mmol) in MeOH (200 mL) was added NaOMe until pH >10. After stirring overnight, the reaction mixture was quenched by addition of dry ice and solvent was removed *in vacuo*. Purification by column chromatography (10:90 - 50:50 Et_2O:pentane) afforded the title compound as a white solid (1.20 g, 4.52 mmol, 86%). ^1H NMR (400 MHz, Chloroform-*d*) δ 7.48 – 7.36 (m, 2H), 7.36 – 7.16 (m, 5H), 5.65 (d, *J* = 5.4 Hz, 1H), 4.48 (qdd, *J* = 6.5, 1.6, 0.6 Hz, 1H), 4.32 – 4.13 (m, 1H), 3.67 (dt, *J* = 3.1, 1.3 Hz, 1H), 2.27 (ddd, *J* = 13.4, 12.0, 5.8 Hz, 1H), 2.11 (ddt, *J* = 13.3, 4.9, 1.1 Hz, 1H), 1.92 (d, *J* = 7.8 Hz, 1H), 1.33 (d, *J* = 6.5 Hz, 3H). ^{13}C NMR (101 MHz, $CDCl_3$) δ 134.9, 131.1, 129.1, 127.3, 84.0, 67.2, 66.6, 66.6, 34.4, 17.9. HRMS: $[M-N_2 +H]^+$ calculated for $C_{19}H_{20}NO_3S$ 238.0896; found 238.0903.

Phenyl-3-*O*-*tert*-butyldimethylsilyl-4-azido-2,4-deoxy-1-thio-L-fucopyranoside (S22)

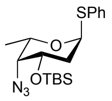

To a solution of **S21** (318 mg, 1.20 mmol) in DMF (7.5mL), imidazole (204 mg, 1.80 mmol, 1.5 eq) and TBS-Cl (50 wt% in toluene, 0.20 mL, 2.4 mmol, 2 eq) were added and the reaction mixture was stirred for 3h. The reaction mixture was diluted with Et_2O, washed 4x with H_2O and once with brine, dried over $MgSO_4$ and solvent was removed *in vacuo*. Purification by column chromatography (5:95 Et_2O:pentane) afforded the title compound as a white solid (429 mg, 1.13 mmol, 94%). ^1H NMR (400 MHz, Chloroform-*d*) δ 7.48 – 7.37 (m, 2H), 7.33 – 7.20 (m, 3H), 5.64 (d, *J* = 5.7 Hz, 1H), 4.38 (qd, *J* = 6.3, 1.5 Hz, 1H), 4.28 (ddd, *J* = 11.8, 4.7, 3.3 Hz, 1H), 3.56 (dt, *J* = 2.9, 1.3 Hz, 1H), 2.43 (ddd, *J* = 13.5, 11.9, 5.8 Hz, 1H), 1.96 (ddt, *J* = 13.4, 4.7, 1.2 Hz, 1H), 1.25 (d, *J* = 6.4 Hz, 3H), 0.93 (s, 9H), 0.15 (d, *J* = 2.5 Hz, 6H). ^{13}C NMR (101 MHz, $CDCl_3$) δ 130.8, 129.1, 127.1, 84.2, 68.9, 66.4, 66.0, 34.8, 25.9, 18.0, -4.4. HRMS: $[M-N_2 +H]^+$ calculated for $C_{24}H_{34}NO_4Si$ 352.1761; found 352.1772.

***o*-Cyclopropylethynylbenzoyl-3-*O*-*tert*-butyldimethylsilyl-4-azido-2,4-deoxy-L-fucopyranoside (S23)**

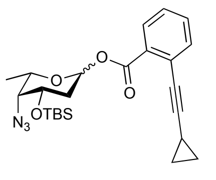

Thioglycoside **S22** (410 mg, 1.09 mmol, 1 eq) was dissolved in THF/H_2O (6 mL, 9:1 v/v), to which $AgNO_3$ (648 mg, 3.82 mmol, 3.5 eq) was added. After stirring in the dark overnight under regular atmosphere, ethyl acetate (60 mL) and Na_2SO_4 were added and the reaction mixture was stirred for 1 hour, filtered over Celite and concentrated *in vacuo*. Column chromatography (20:80 Et_2O:pentane) afforded the crude hemiacetal. This was esterified according to general procedure B. Column chromatography (0:100 - 5:95 Et_2O:pentane) afforded the title compound as a colourless oil (297 mg, 0.598 mmol, 65%, 1:3 α:β). Spectral data for the β-anomer: ^1H NMR (400 MHz, Chloroform-*d*) δ 7.98 (ddd, *J* = 8.0, 1.4, 0.6 Hz, 1H), 7.48 (ddd, *J* = 7.8, 1.6, 0.6 Hz, 1H), 7.42 (td, *J* = 7.5, 1.4 Hz, 1H), 7.33 – 7.26 (m, 2H), 5.88 (dd, *J* = 10.2, 2.5 Hz, 1H), 4.10 (ddd, *J* = 11.6, 4.8, 3.4 Hz, 1H), 3.69 (qd, *J* = 6.3, 1.4 Hz, 1H), 3.49 (dt, *J* = 3.4, 1.2 Hz, 1H), 2.16 (td, *J* = 11.9, 10.1 Hz, 1H), 2.01 (dddd, *J* = 12.3, 4.8, 2.5, 1.1 Hz, 1H), 1.57 – 1.45 (m, 1H), 1.35 (d, *J* = 6.4 Hz, 3H), 0.93 (s, 9H), 0.91 – 0.88 (m, 4H), 0.14 (d, *J* = 2.7 Hz, 6H). ^{13}C NMR (101 MHz, $CDCl_3$) δ 164.4, 134.4, 132.2, 131.0, 130.7, 127.1, 125.3, 99.9, 92.6, 74.6, 71.0, 70.3, 65.1, 34.5, 25.8, 17.9, 9.0, 0.8, -4.4, -4.6. HRMS: $[M +Na]^+$ calculated for $C_{24}H_{33}N_3O_4SiNa$ 478.2133; found 478.2141.

7-[3-*O-tert*-butylsilyldimethylsilyl-4-azido-2,4-deoxy-α-L-fucopyranoside]-14-*O*-tert-butyldimethylsilyldoxorubicinone (S24)

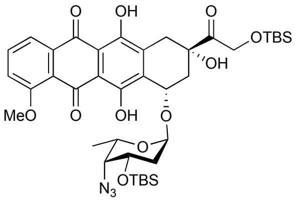

According to general procedure C, glycosyl donor **S23** (266 mg, 0.585 mmol) was coupled to 14-O-*tert*-butyldimethylsilyl-doxorubicinone **S8** (464 mg, 0.877 mmol, 1.5 eq). Column chromatography (15:85 Et_2O:pentane and then 1:99 - 10:90 acetone:toluene) of the residue gave the title compound as a red solid (440 mg, 0.551 mmol, 94%). ^{1}H NMR (400 MHz, Chloroform-*d*) δ 13.84 (s, 1H), 13.07 (s, 1H), 7.91 (dd, *J* = 7.7, 1.1 Hz, 1H), 7.73 (dd, *J* = 8.4, 7.7 Hz, 1H), 7.36 (dd, *J* = 8.7, 1.1 Hz, 1H), 5.47 (d, *J* = 4.0 Hz, 1H), 5.18 (dd, *J* = 4.0, 2.1 Hz, 1H), 4.96 – 4.77 (m, 2H), 4.50 (s, 1H), 4.07 (s, 3H), 4.05 – 3.94 (m, 2H), 3.54 (d, *J* = 3.1 Hz, 1H), 3.09 (dd, *J* = 18.9, 1.8 Hz, 1H), 2.80 (d, *J* = 18.9 Hz, 1H), 2.25 (dt, *J* = 14.8, 2.1 Hz, 1H), 2.19 – 2.12 (m, 1H), 2.03 (ddd, *J* = 13.4, 11.8, 4.2 Hz, 1H), 1.83 – 1.69 (m, 1H), 1.31 (d, *J* = 6.4 Hz, 3H), 0.96 (s, 9H), 0.85 (s, 9H), 0.14 (d, *J* = 1.4 Hz, 6H). ^{13}C NMR (101 MHz, $CDCl_3$) δ 211.1, 186.8, 186.5, 161.0, 156.3, 155.6, 135.7, 135.3, 134.0, 133.8, 120.7, 119.8, 118.5, 111.3, 111.3, 101.3, 77.5, 77.3, 77.2, 76.8, 69.6, 67.9, 66.6, 66.1, 56.7, 35.5, 34.0, 33.8, 25.9, 25.7, 18.7, 18.1, 18.0, -4.4, -4.7, -5.2, -5.3. HRMS: $[M+Na]^+$ calculated for $C_{39}H_{55}N_3O_{11}Si_2Na$ 820.3267; found 820.3287.

3'-Desamino-3'-hydroxy-4'-deoxy-4'-aminodoxorubicin hydrochloride (13)

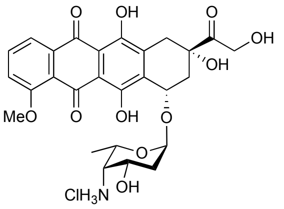

To a solution of **S24** (110 mg, 0.138 mmol, 1 eq) in THF/H_2O (20 mL, 10:1 v/v) was added triphenylphosphine (108 mg, 0.412 mmol, 3 eq) and the reaction mixture was stirred for a week at 50 °C. Solvent was removed *in vacuo* and the residue was subjected to column chromatography (30:70 EtOAc:pentane, then 10:90 – 20:80 acetone:toluene) to give the crude amine. The amine thus obtained was desilylated using HF·pyridine (70 wt% HF, 0.24 mL) according to general procedure E. Column chromatography on neutral silica (0:100 – 10:90 MeOH:DCM) afforded the title compound as a red solid (27 mg, 49.7 µmol, 36% over 2 steps). The corresponding HCl salt was prepared by lyophilization from aqueous 10 µM HCl (1 mL/µmol, 2 eq). ^{1}H NMR (500 MHz, DMSO-d_6) δ 14.06 (s, 1H), 13.26 (s, 1H), 7.92 (q, *J* = 4.0, 3.2 Hz, 2H), 7.87 – 7.75 (m, 3H), 7.71 – 7.62 (m, 1H), 5.69 (d, *J* = 26.2 Hz, 1H), 5.51 (s, 1H), 5.29 (d, *J* = 3.9 Hz, 1H), 4.95 (t, *J* = 4.5 Hz, 1H), 4.58 (s, 2H), 4.37 (q, *J* = 6.5 Hz, 1H), 3.99 (s, 5H), 3.19 (d, *J* = 27.2 Hz, 2H), 3.05 – 2.88 (m, 2H), 2.12 (d, *J* = 4.4 Hz, 2H), 1.75 (dd, *J* = 13.5, 5.4 Hz, 1H), 1.65 (td, *J* = 13.1, 4.0 Hz, 1H), 1.21 (d, *J* = 6.7 Hz, 3H). ^{13}C NMR (126 MHz, DMSO) δ 213.9, 186.6, 186.6, 160.8, 156.0, 154.4, 136.3, 135.1, 134.7, 134.2, 120.0, 119.8, 119.1, 110.8, 100.2, 74.8, 70.2, 63.7, 63.0, 61.5, 56.6, 54.1, 36.5, 32.4, 32.0, 16.9. HRMS: $[M+H]^+$ calculated for $C_{27}H_{30}NO_{11}$ 544.1813; found 544.1816.

7-[3-*O-tert*-butyldimethylsilyl-4-dimethylamino-2,4-deoxy-α-L-fucopyranoside]-14-*O*-tert-butyldimethylsilyldoxorubicinone (S25)

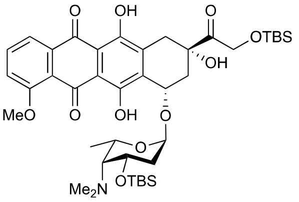

To a solution of **S24** (130 mg, 0.160 mmol, 1 eq) in THF/H_2O (26 mL, 10:1 v/v) was added triphenylphosphine (129 mg, 49 mmol, 3 eq) and the reaction mixture was stirred for four days at 50 °C. Solvent was removed *in vacuo* and the red residue was subjected to column chromatography (1:99 – 15:85 acetone:toluene). To the amine thus obtained (65 mg, 83 µmol) in EtOH (6.8 mL) were added aq. CH_2O (aq. 37% w/v, 0.20 mL, 30 eq) and $NaBH(OAc)_3$ (34.6 mg, 16.4 mmol, 1.95 eq). The reaction mixture was stirred for two hours and partitioned between DCM and sat. aq. $NaHCO_3$. The aqueous layer was extracted thrice with DCM and combined organics were dried over Na_2SO_4 and concentrated *in vacuo*. Purification by column chromatography (1:199 - 5:95 acetone:toluene) afforded the title compound as

a red solid (55 mg, 69 µmol, 43% over 2 steps). ^{1}H NMR (400 MHz, Chloroform-*d*) δ 13.90 (s, 1H), 13.23 (s, 1H), 8.00 (dd, *J* = 7.7, 1.1 Hz, 1H), 7.76 (dd, *J* = 8.5, 7.7 Hz, 1H), 7.38 (dd, *J* = 8.6, 1.2 Hz, 1H), 5.51 (t, *J* = 4.0 Hz, 1H), 5.26 (dd, *J* = 3.9, 2.2 Hz, 1H), 5.02 – 4.83 (m, 2H), 4.78 (s, 1H), 4.20 (ddq, *J* = 18.2, 9.1, 4.5, 4.1 Hz, 2H), 4.09 (s, 3H), 3.17 (dd, *J* = 19.0, 1.9 Hz, 1H), 2.97 (d, *J* = 18.9 Hz, 1H), 2.53 (s, 6H), 2.47 (t, *J* = 2.1 Hz, 1H), 2.42 – 2.34 (m, 1H), 2.16 – 2.04 (m, 2H), 1.96 (s, 1H), 1.69 (dt, *J* = 13.7, 4.5 Hz, 1H), 1.40 (d, *J* = 6.7 Hz, 3H), 0.96 (s, 9H), 0.90 (s, 9H), 0.14 (d, *J* = 2.8 Hz, 6H), 0.05 (s, 6H). ^{13}C NMR (101 MHz, $CDCl_3$) δ 211.9, 187.1, 186.7, 161.1, 156.5, 156.0, 135.7, 135.6, 134.6, 134.1, 121.1, 119.9, 118.5, 111.5, 111.4, 99.6, 77.3, 70.1, 69.7, 69.2, 66.8, 65.4, 56.8, 44.9, 37.7, 35.4, 34.0, 26.0, 18.7, 18.1, 17.1, -4.3, -4.6, -5.1, -5.2. HRMS: [M+H$^+$] calculated for $C_{41}H_{62}NO_{11}Si_2$ 800.3861; found 800.3852.

***N,N*-dimethylisodoxorubicin (14)**

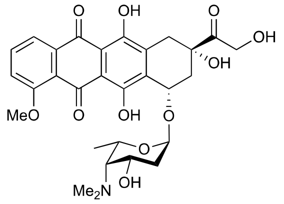

S25 (29 mg, 36 µmol) was desilylated using HF·pyridine (70 wt% HF, 0.14 mL) according to general procedure E. Column chromatography on neutral silica (0:100 – 95:5 MeOH:DCM) afforded the title compound as a red solid (15 mg, 26 µmol, 72%). ^{1}H NMR (500 MHz, Chloroform-*d*) δ 13.92 (s, 1H), 13.22 (s, 1H), 8.01 (dd, *J* = 7.7, 1.1 Hz, 1H), 7.77 (dd, *J* = 8.4, 7.7 Hz, 1H), 7.39 (dd, *J* = 8.5, 1.1 Hz, 1H), 5.55 (t, *J* = 4.2 Hz, 1H), 5.30 (dd, *J* = 3.9, 2.2 Hz, 1H), 4.81 (s, 1H), 4.77 (d, *J* = 1.3 Hz, 2H), 4.29 (qd, *J* = 7.0, 3.8 Hz, 1H), 4.08 (s, 3H), 3.94 (dt, *J* = 9.0, 4.8 Hz, 1H), 3.24 (dd, *J* = 18.9, 2.0 Hz, 1H), 2.99 (d, *J* = 18.8 Hz, 1H), 2.54 (s, 6H), 2.49 – 2.43 (m, 2H), 2.12 (dd, *J* = 14.7, 3.9 Hz, 1H), 1.91 (ddd, *J* = 14.0, 8.5, 3.8 Hz, 1H), 1.78 (dt, *J* = 14.0, 4.8 Hz, 1H), 1.52 (d, *J* = 7.0 Hz, 3H). ^{13}C NMR (126 MHz, $CDCl_3$) δ 214.4, 187.1, 186.8, 161.2, 156.4, 155.8, 135.8, 135.6, 134.0, 133.8, 121.1, 119.9, 118.6, 111.7, 111.6, 99.1, 70.0, 69.4, 65.7, 64.4, 64.0, 56.8, 44.7, 35.9, 35.4, 34.1, 17.4. HRMS: [M+H]$^+$ calculated $C_{29}H_{35}NO_{11}$ for 572.2126; found 572.2142.

alpha-anomer

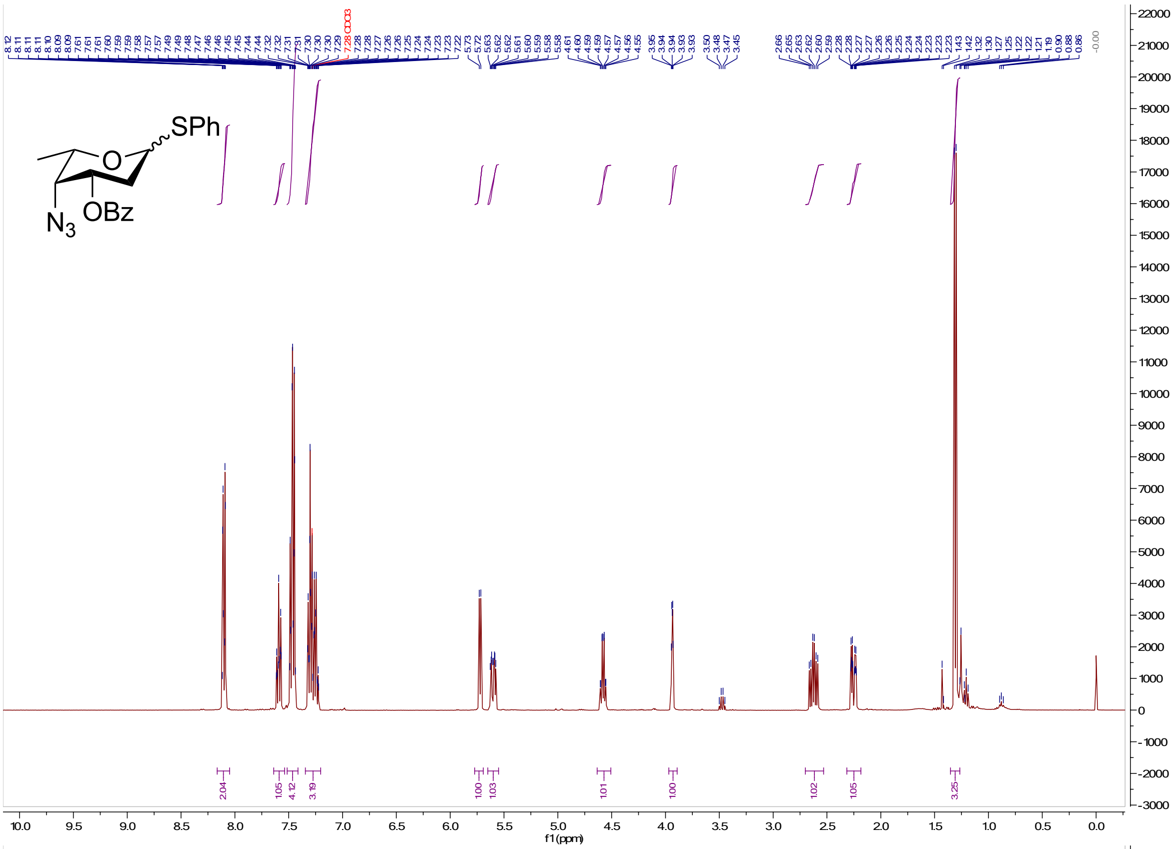

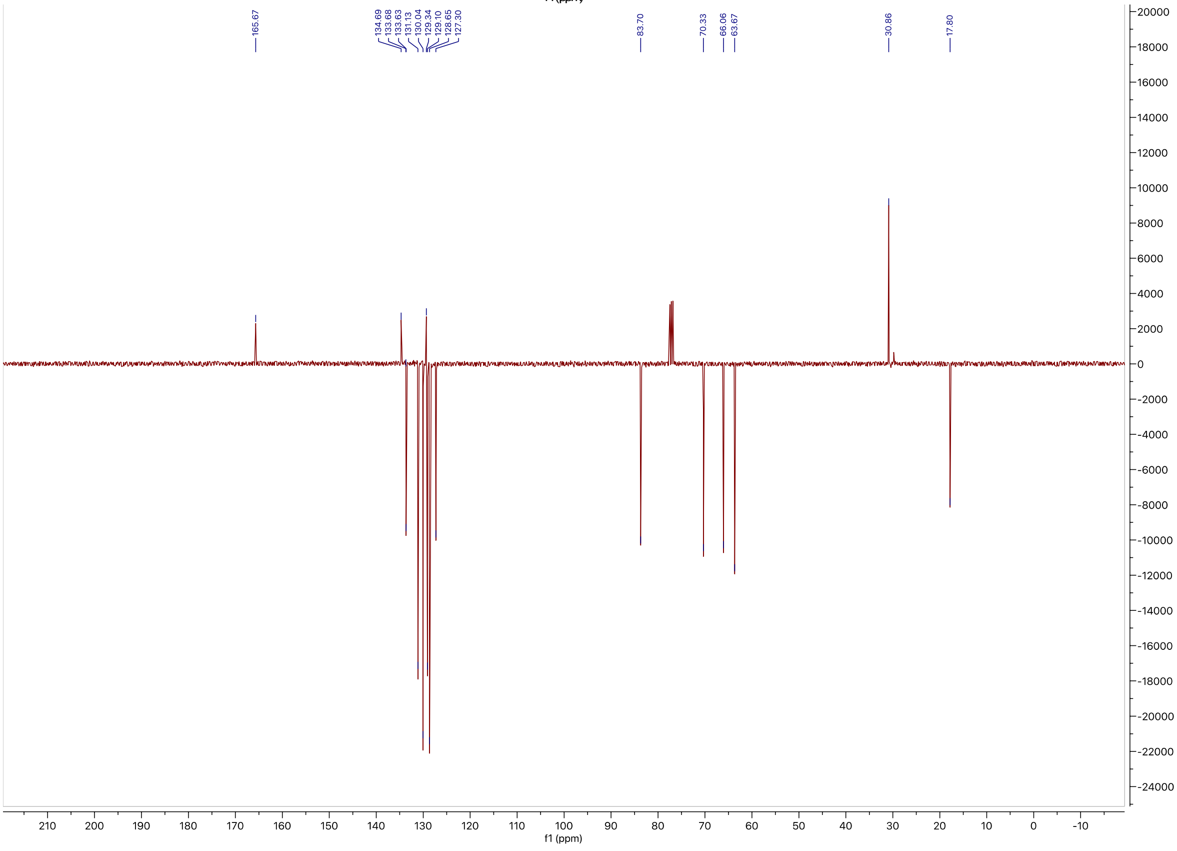

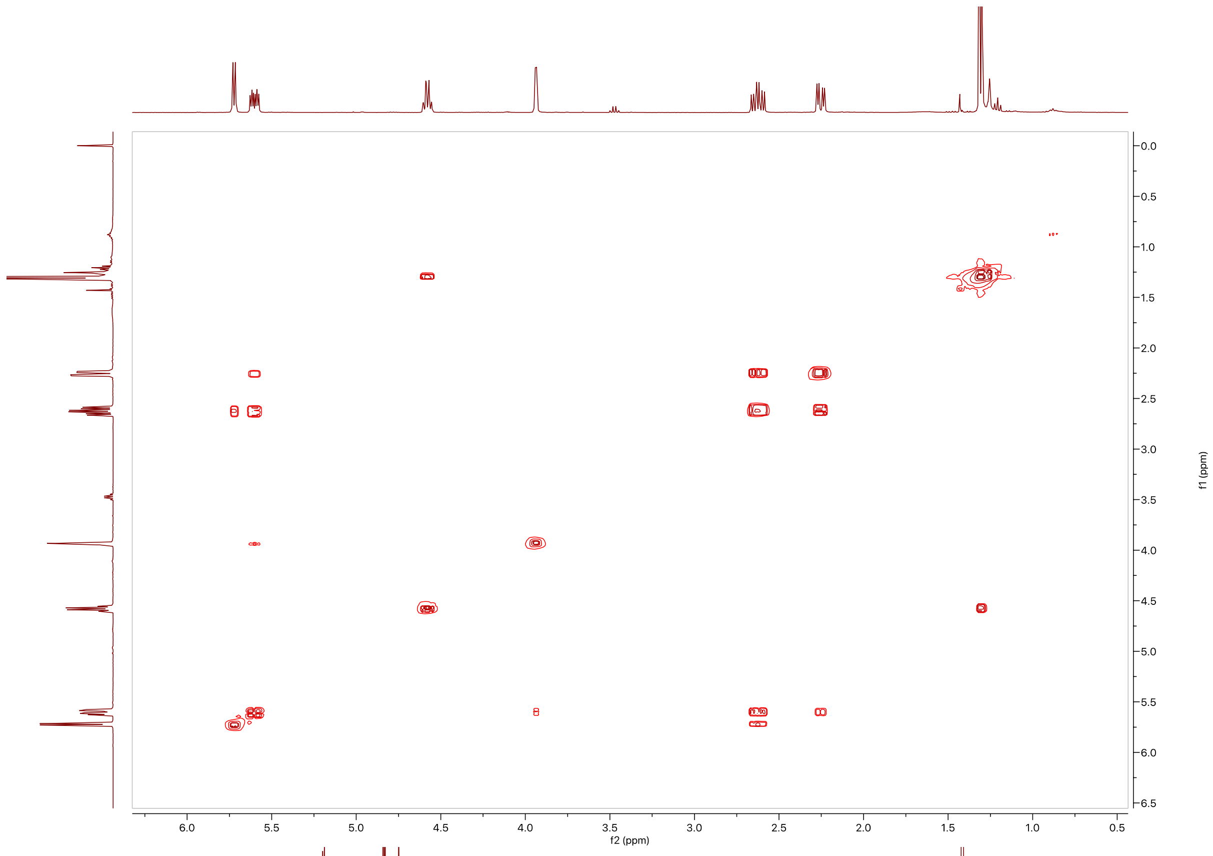
f2 (ppm)
f1 (ppm)

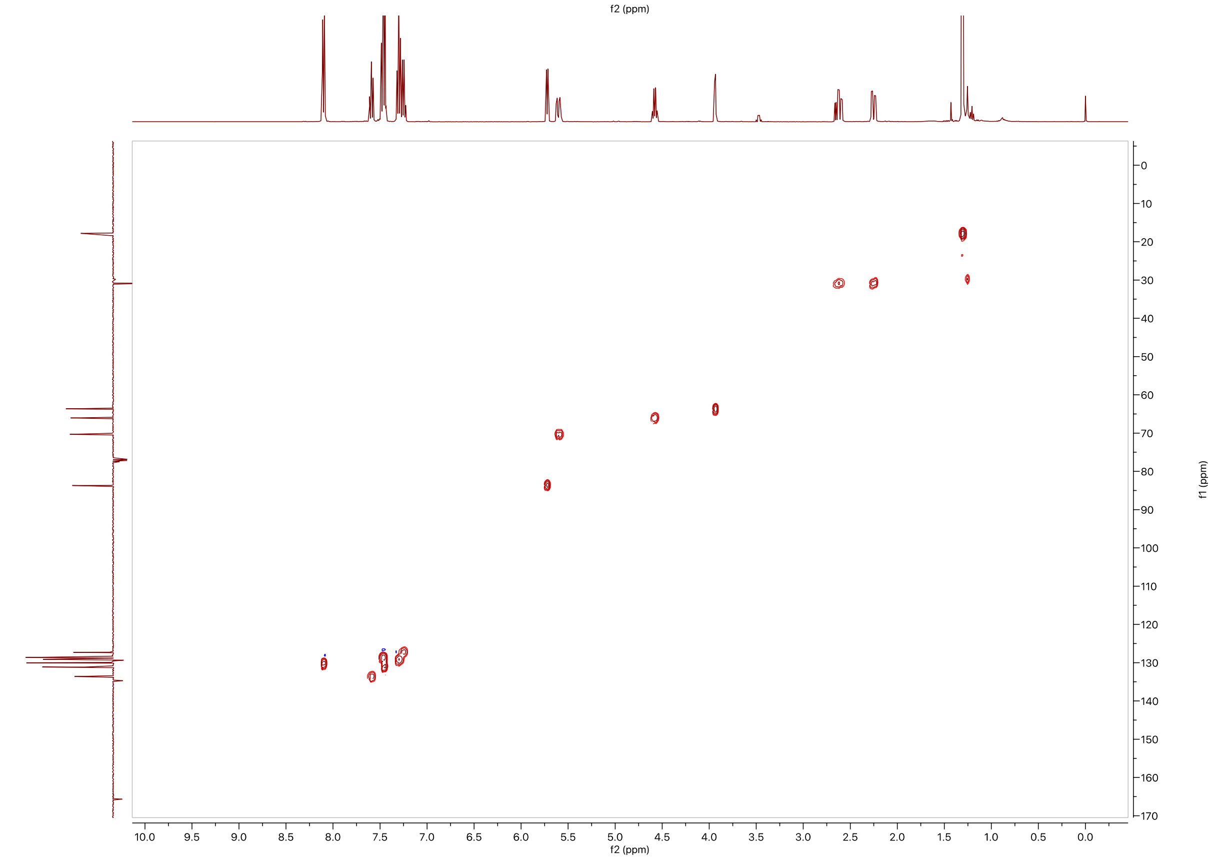
f2 (ppm)
f1 (ppm)

beta-anomer

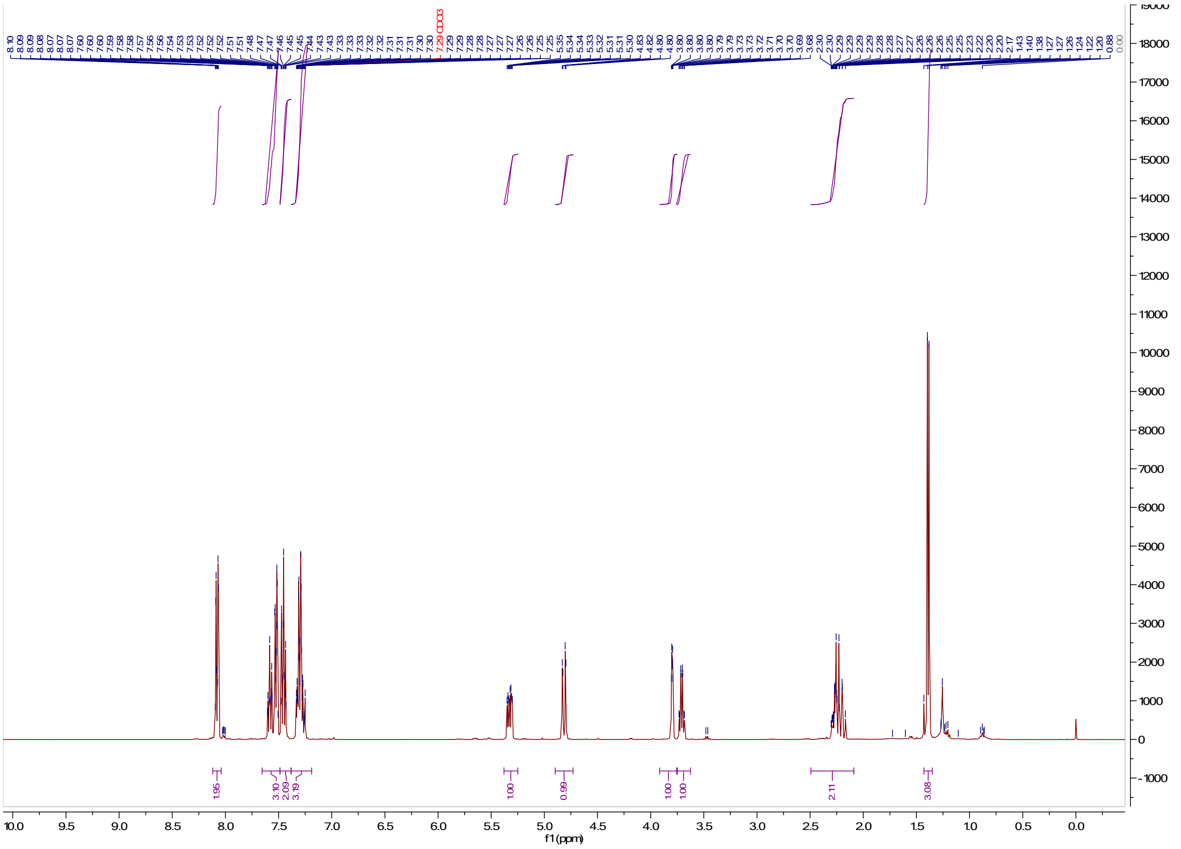

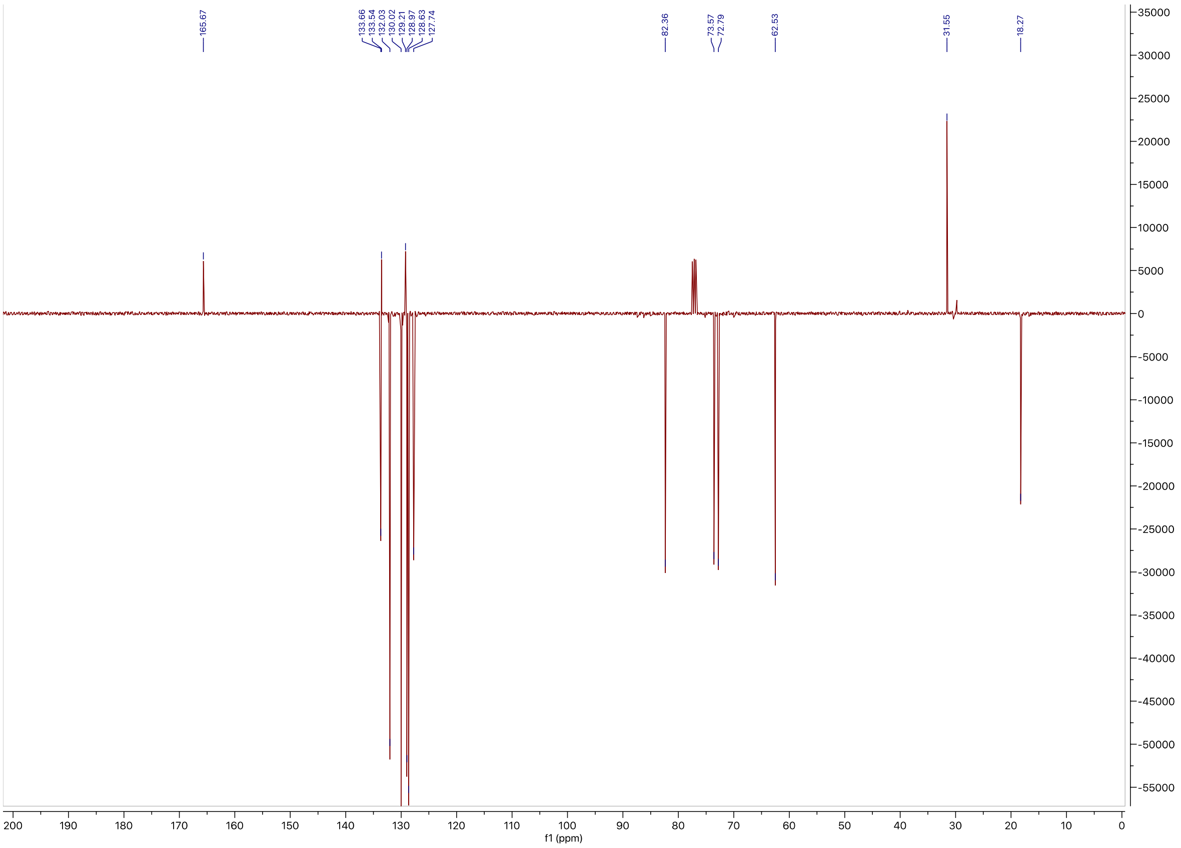

165.67
133.66
133.54
132.03
130.02
129.21
128.97
128.63
127.74
82.36
73.57
72.79
62.53
31.55
18.27
f1 (ppm)

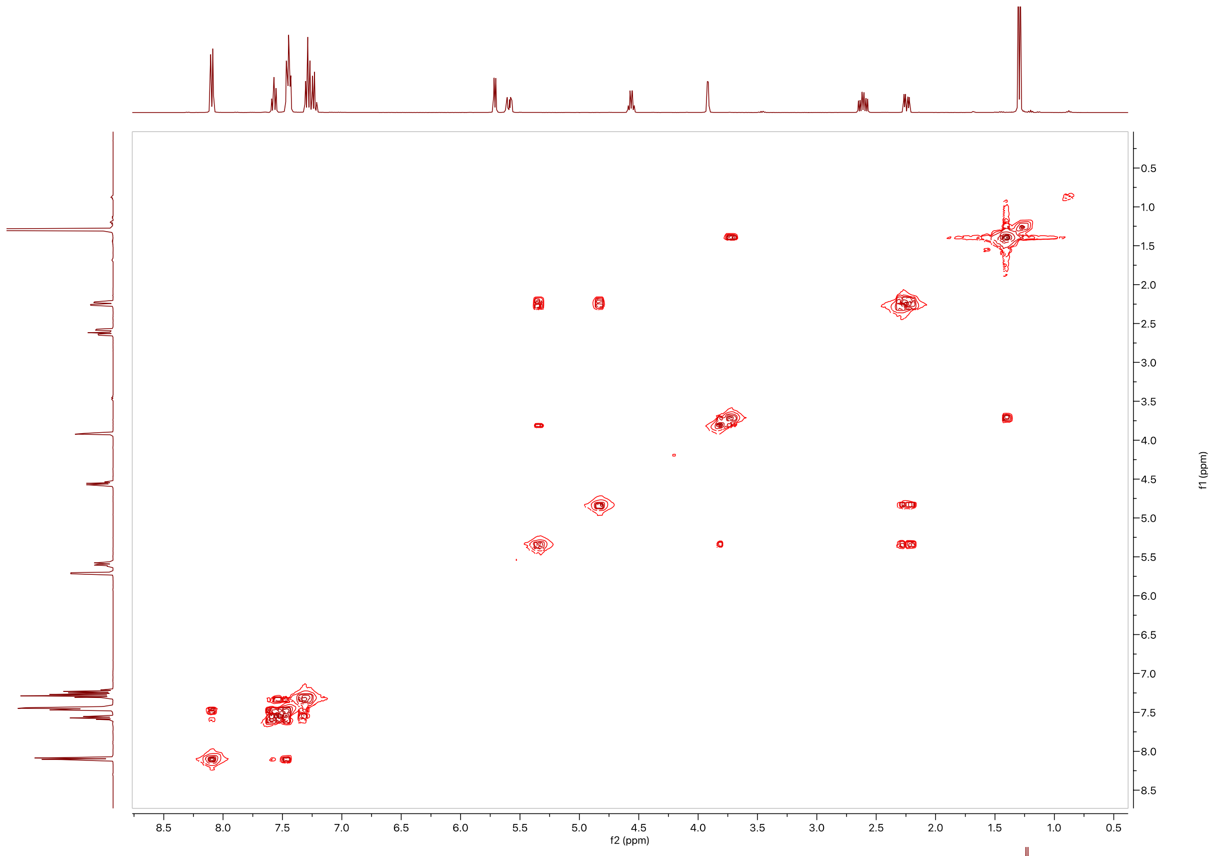

f2 (ppm)
f1 (ppm)

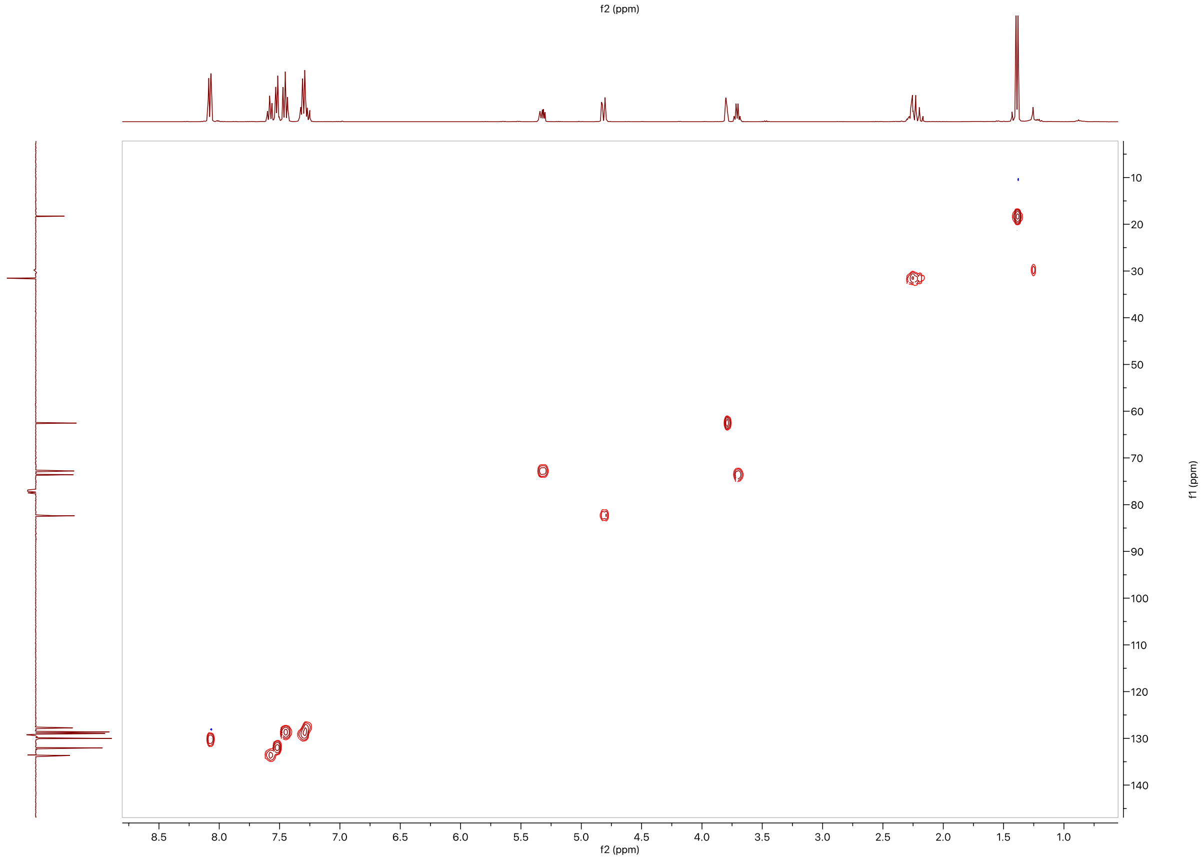

f2 (ppm)
f1 (ppm)

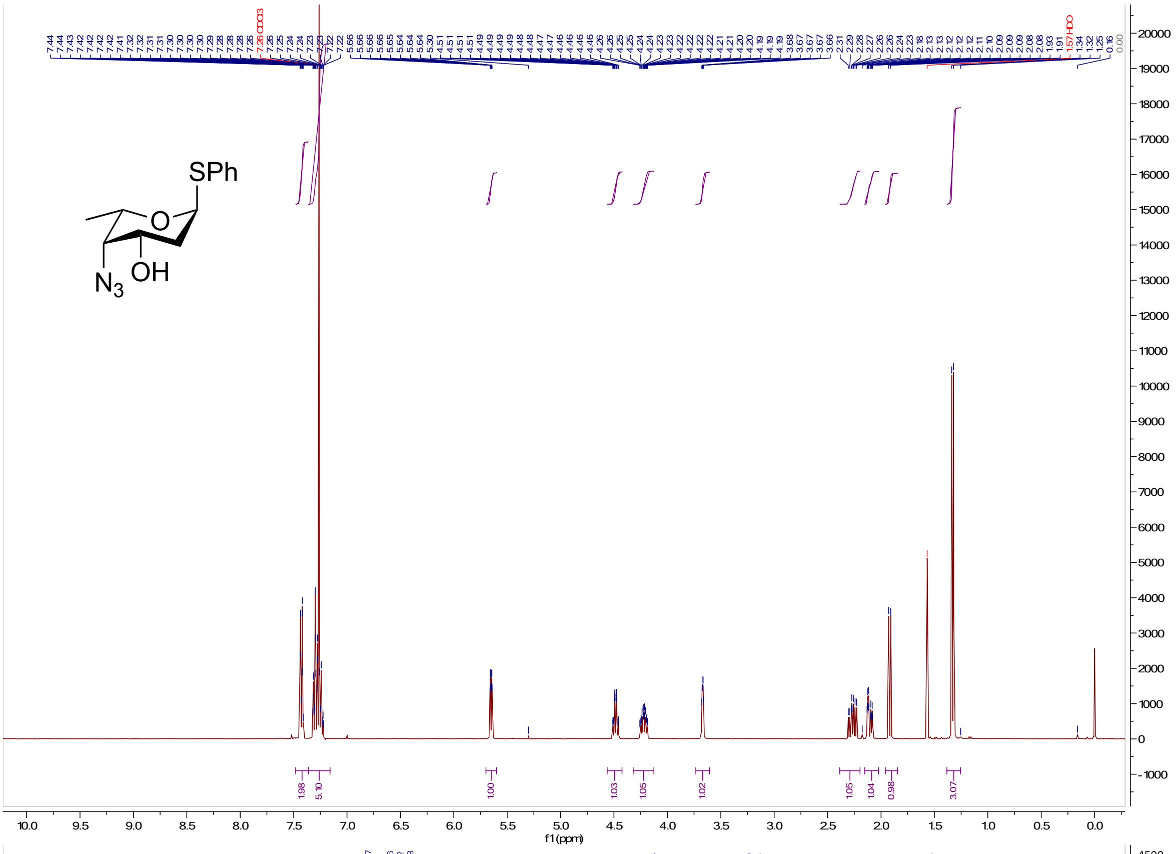
SPh
O
N3
OH
1.98
5.10
1.00
1.03
1.05
1.02
1.05
1.04
0.98
3.07
7.26 CDCl3
1.57 HDO
f1 (ppm)

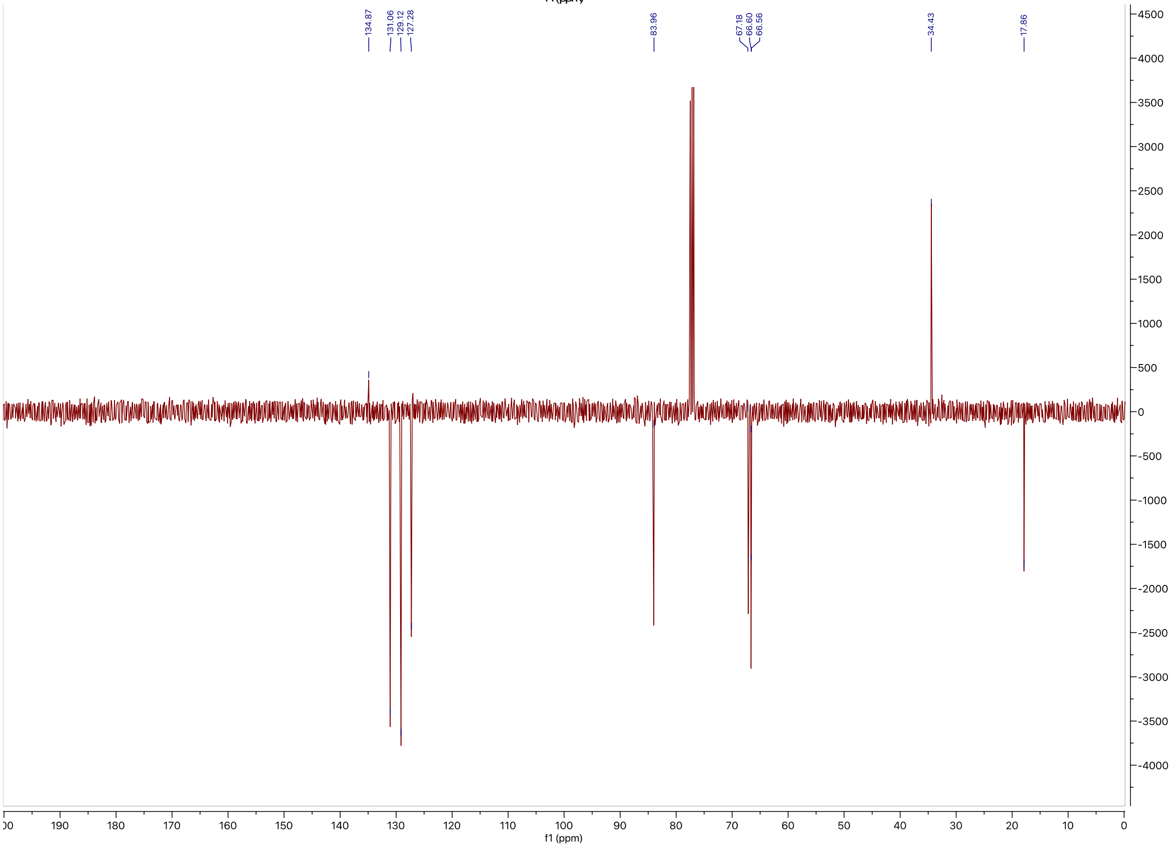
134.87
131.06
129.12
127.28
83.96
67.18
66.60
66.56
34.43
17.86
f1 (ppm)

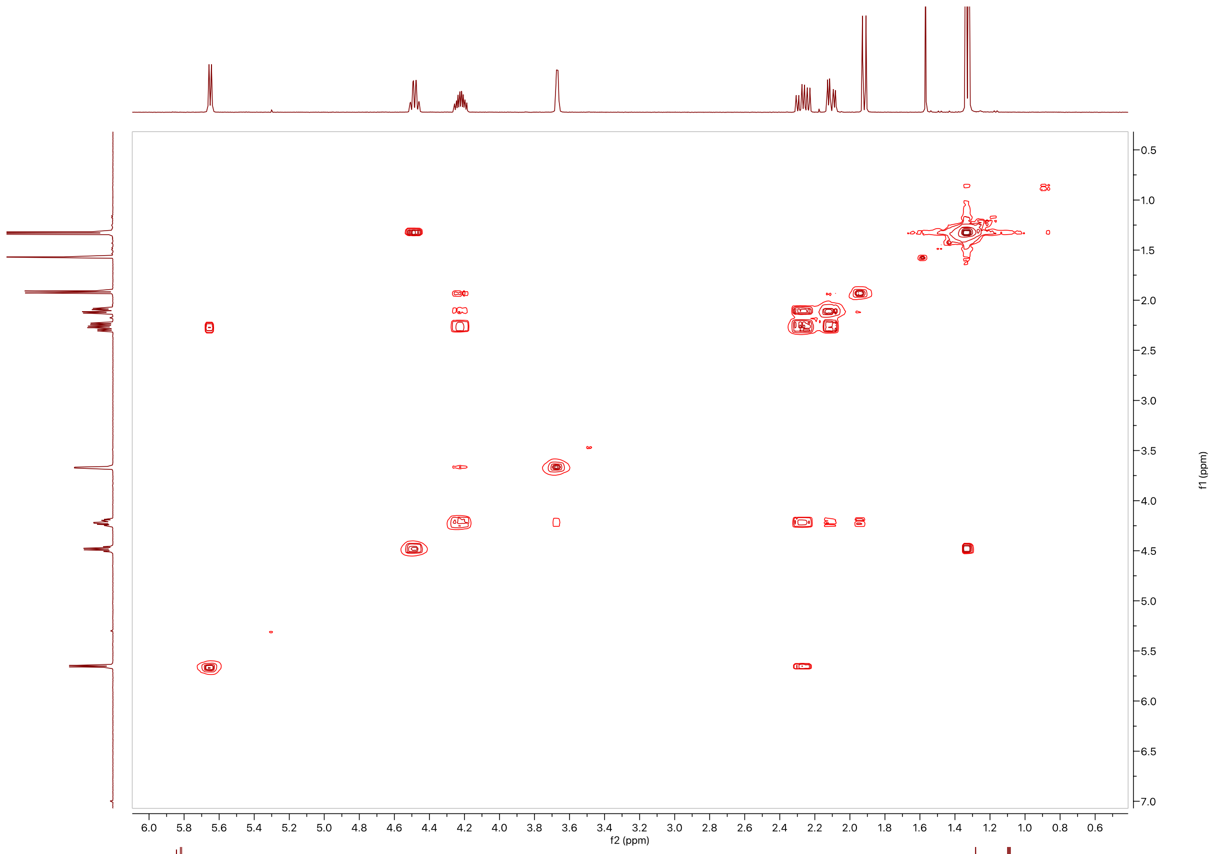
f1 (ppm)
f2 (ppm)

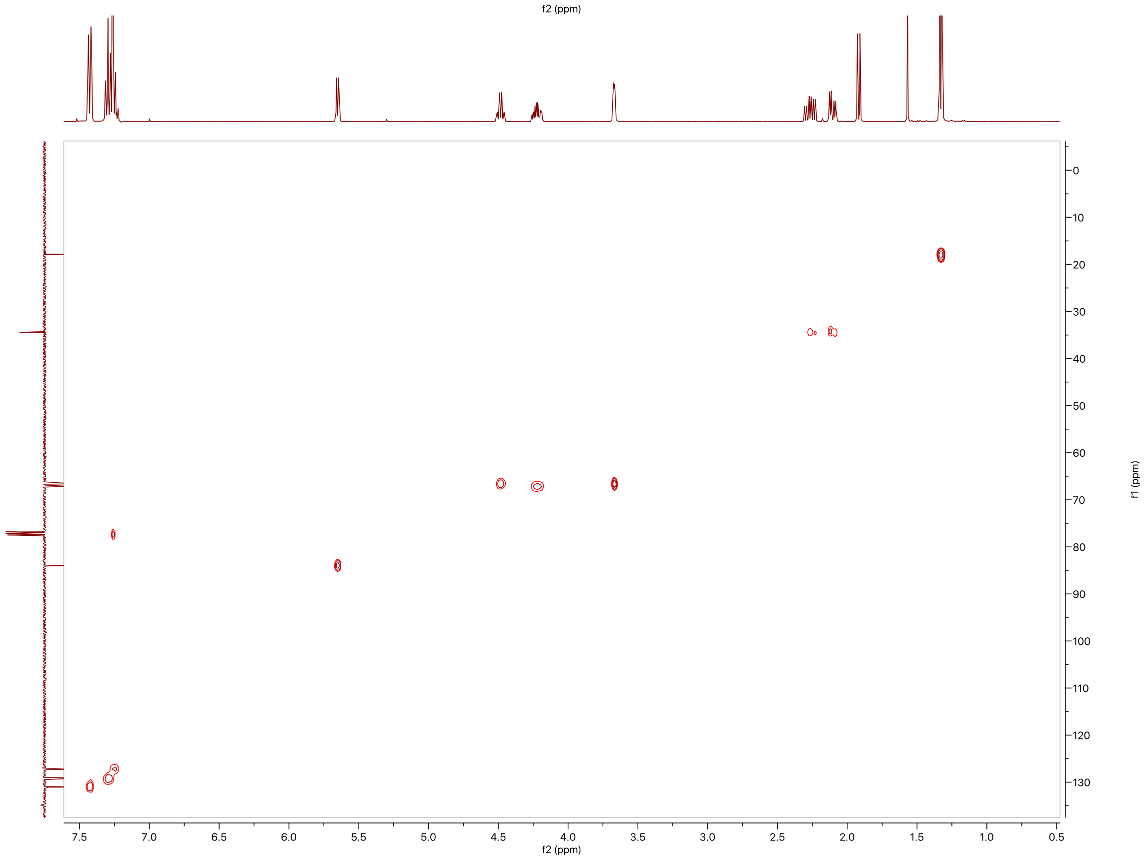
f1 (ppm)
f2 (ppm)

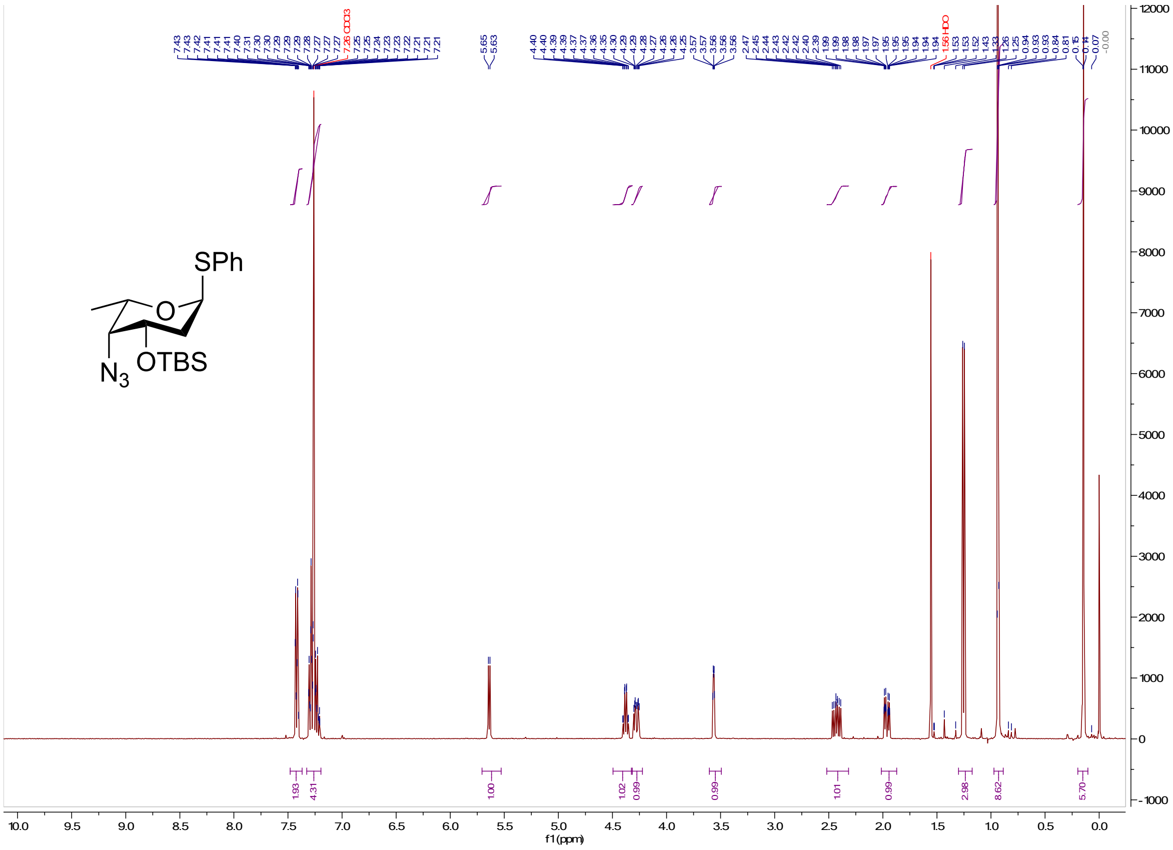
SPh
O
N3
OTBS
1.93
4.31
1.00
1.02
0.99
0.99
1.01
0.99
2.98
8.62
5.70
f1 (ppm)

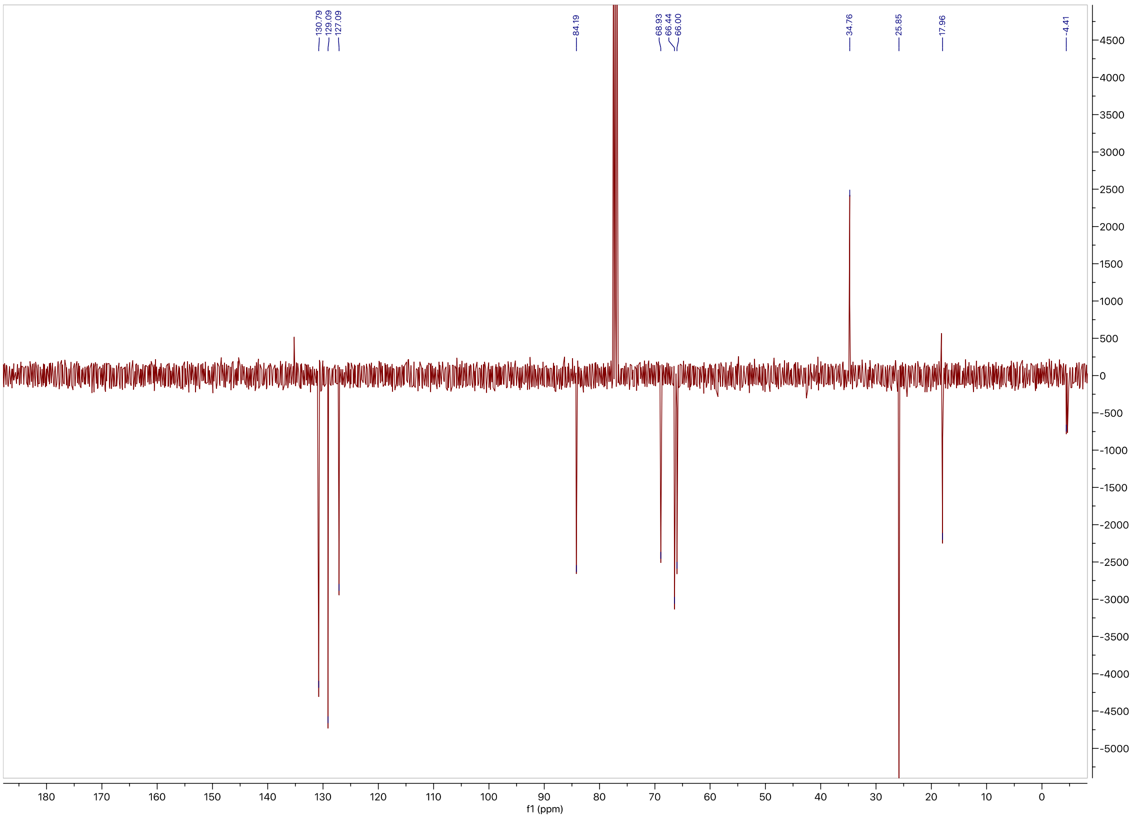
130.79
129.09
127.09
84.19
68.93
66.44
66.00
34.76
25.85
17.96
-4.41
f1 (ppm)

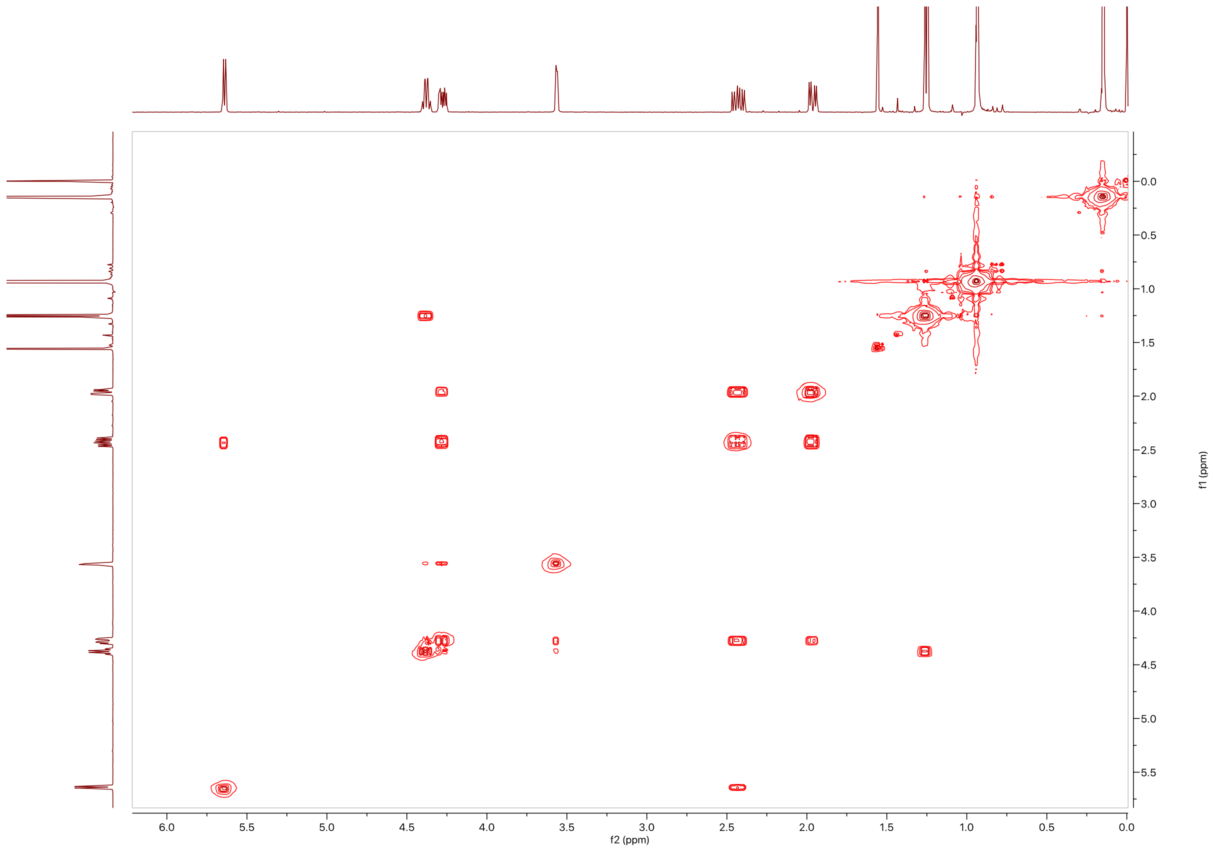
f2 (ppm)
f1 (ppm)

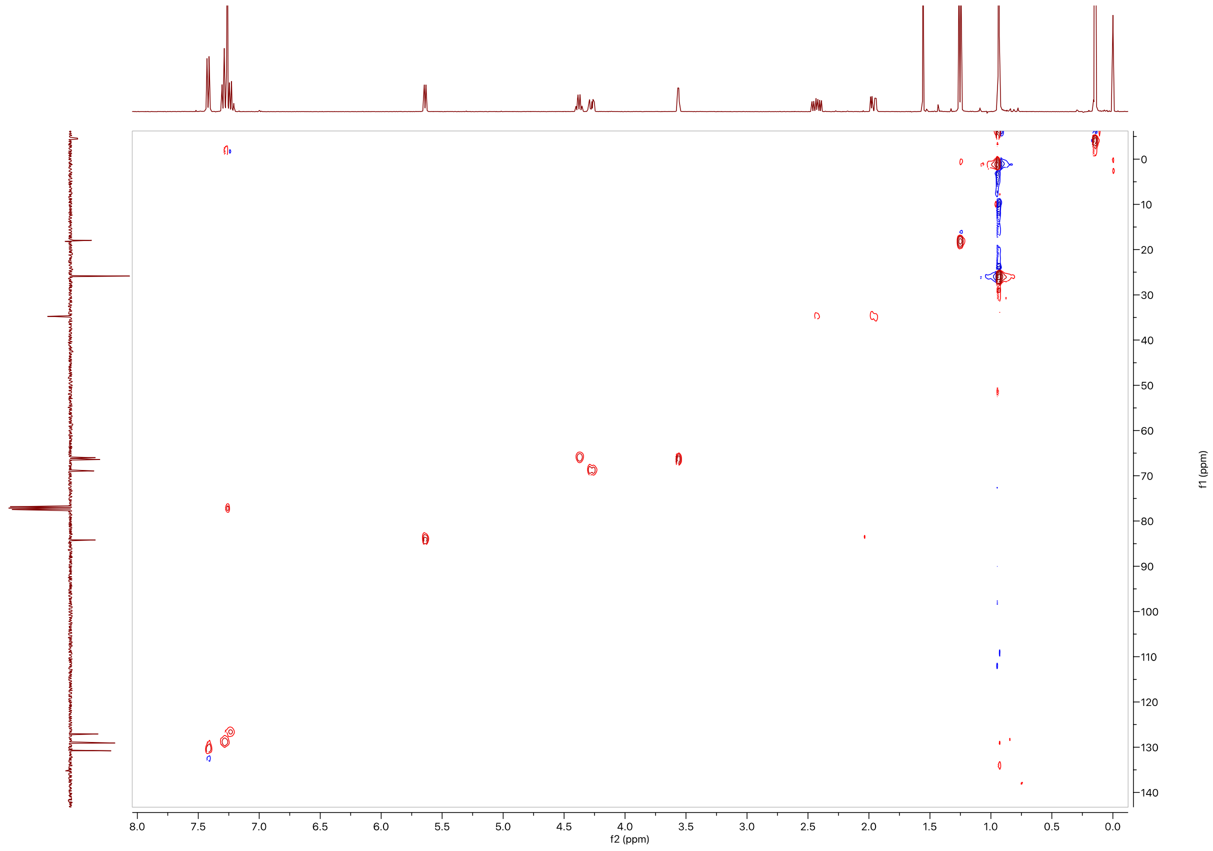
f2 (ppm)
f1 (ppm)

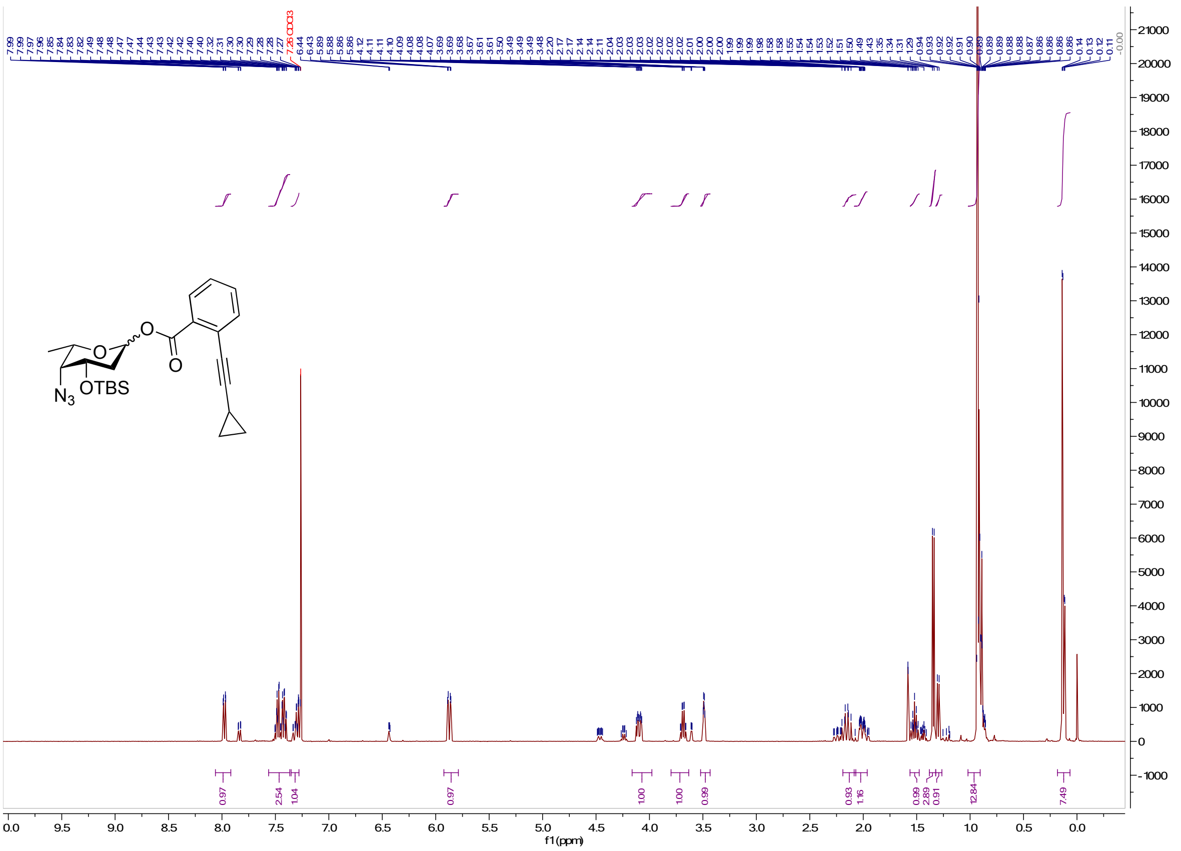
O
O
O
N3
OTBS
0.97
2.54
1.04
0.97
1.00
1.00
0.99
0.93
1.16
0.99
2.89
0.91
12.84
7.49
f1 (ppm)

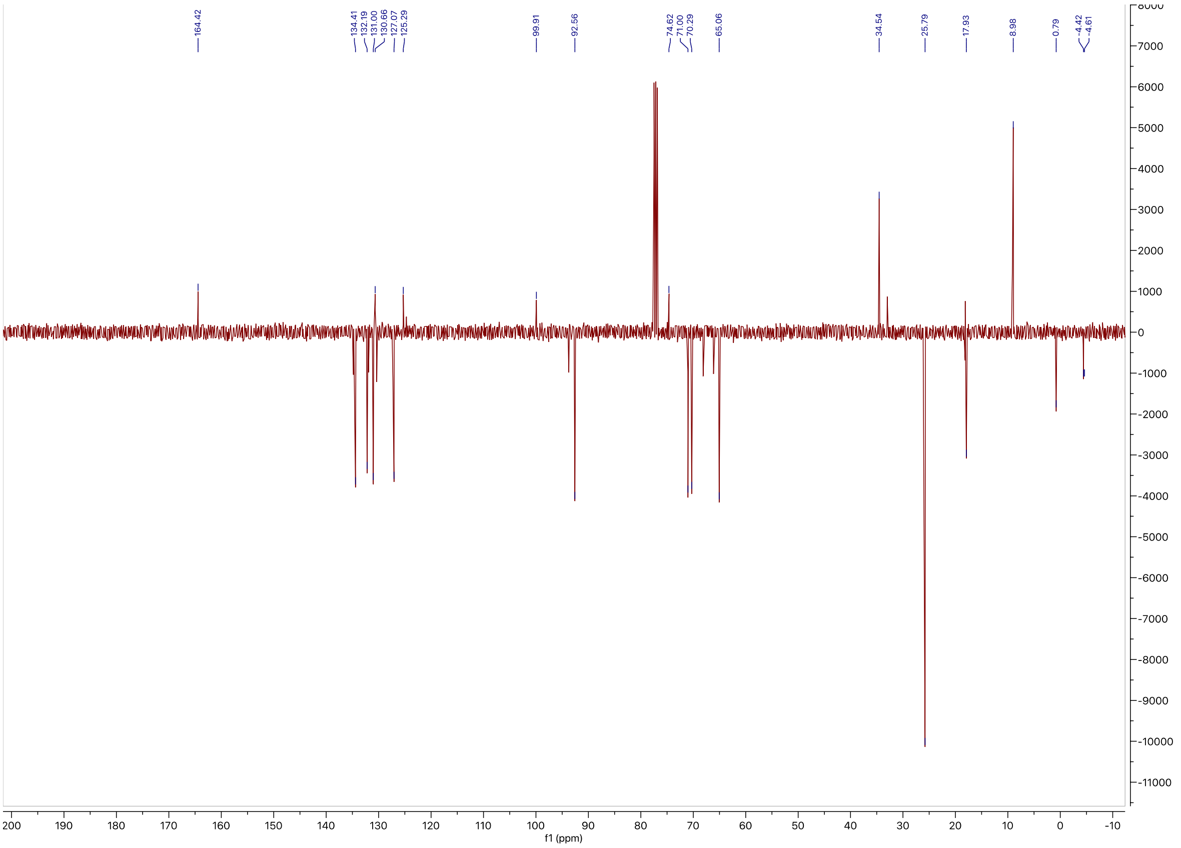
164.42
134.41
132.19
131.00
130.66
127.07
125.29
99.91
92.56
74.62
71.00
70.29
65.06
34.54
25.79
17.93
8.98
0.79
-4.42
-4.61
f1 (ppm)

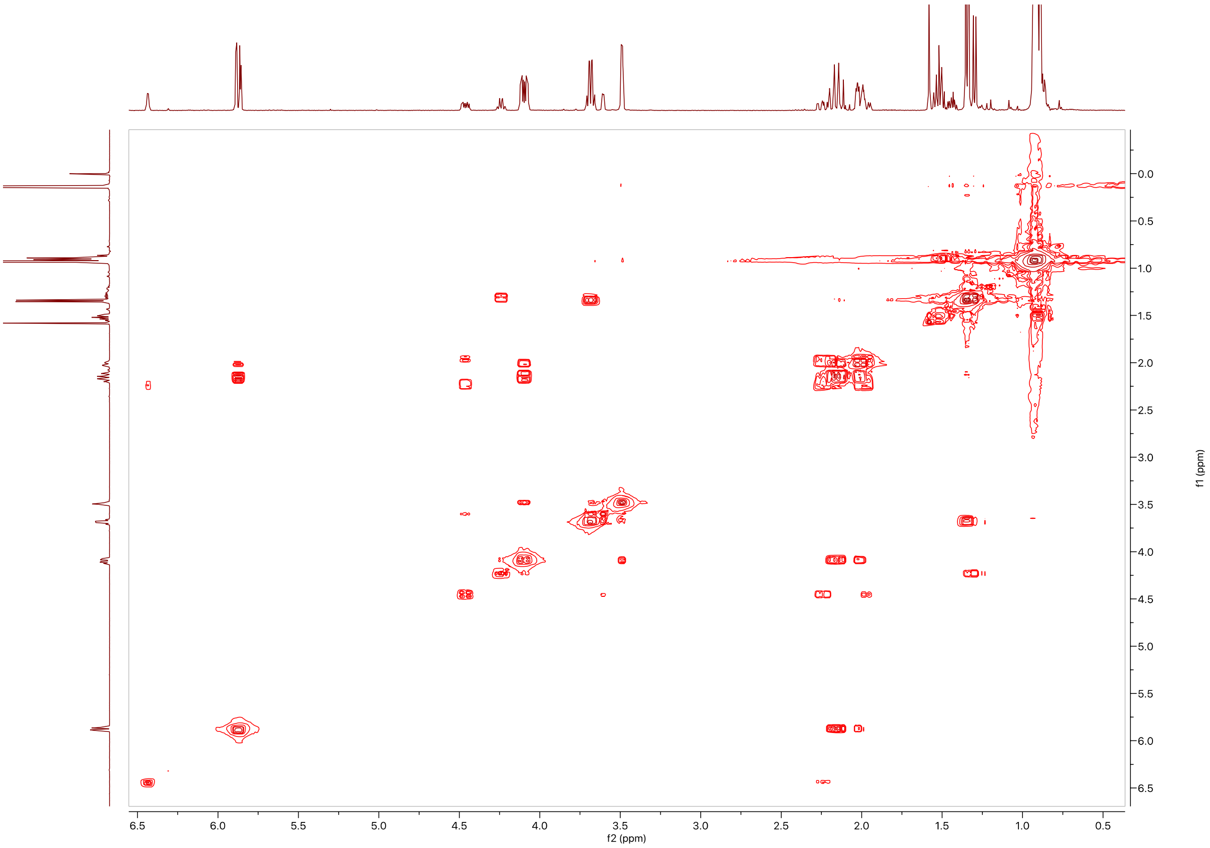
6.5 6.0 5.5 5.0 4.5 4.0 3.5 3.0 2.5 2.0 1.5 1.0 0.5
f2 (ppm)
0.0 0.5 1.0 1.5 2.0 2.5 3.0 3.5 4.0 4.5 5.0 5.5 6.0 6.5
f1 (ppm)

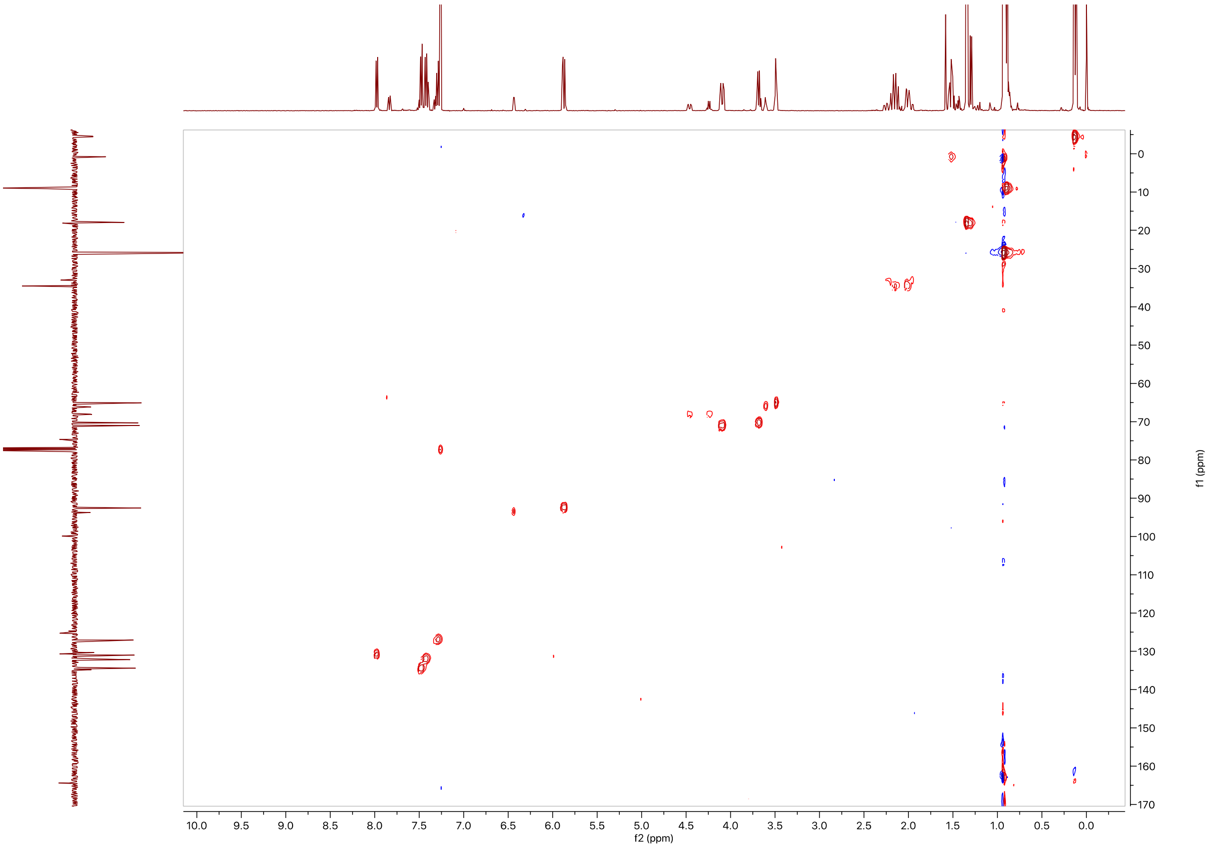
10.0 9.5 9.0 8.5 8.0 7.5 7.0 6.5 6.0 5.5 5.0 4.5 4.0 3.5 3.0 2.5 2.0 1.5 1.0 0.5 0.0
f2 (ppm)
0 10 20 30 40 50 60 70 80 90 100 110 120 130 140 150 160 170
f1 (ppm)

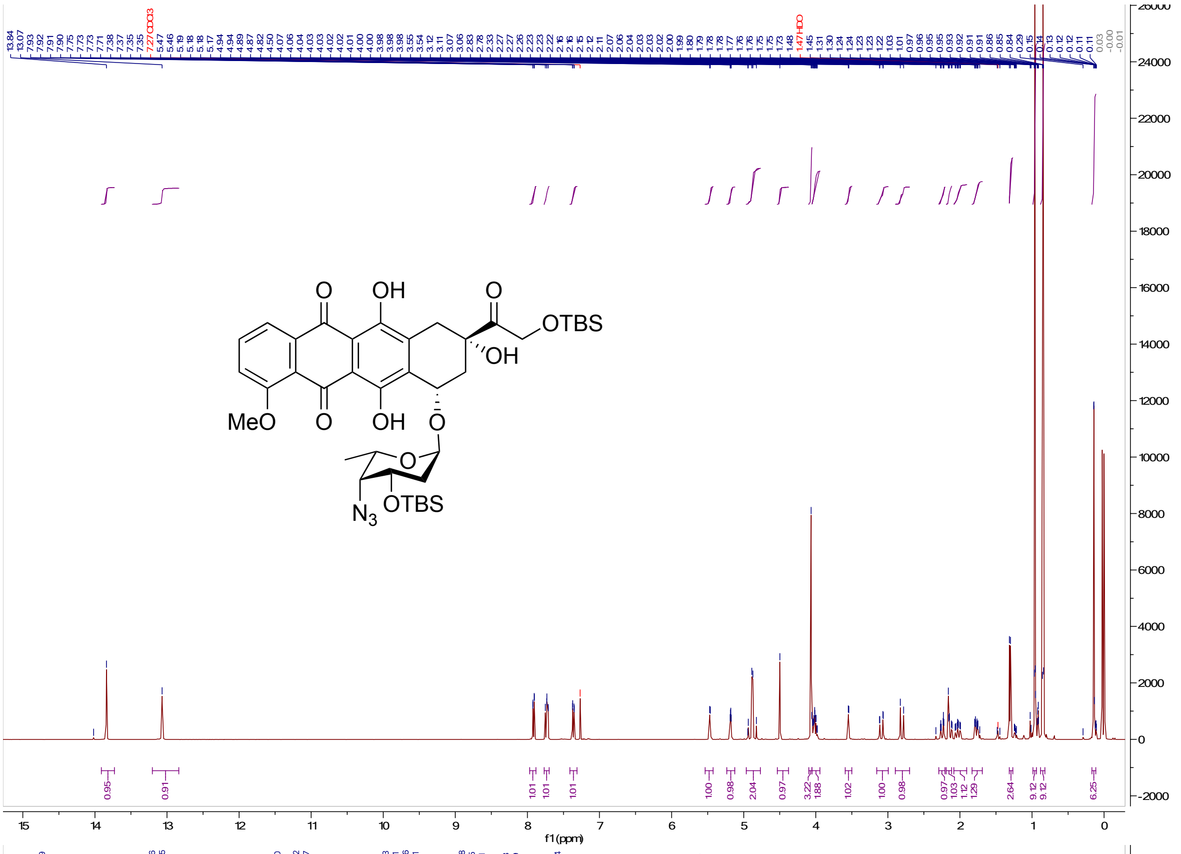
OH
O
OTBS
OH
MeO
O
OH
O
O
N3
OTBS
f1 (ppm)

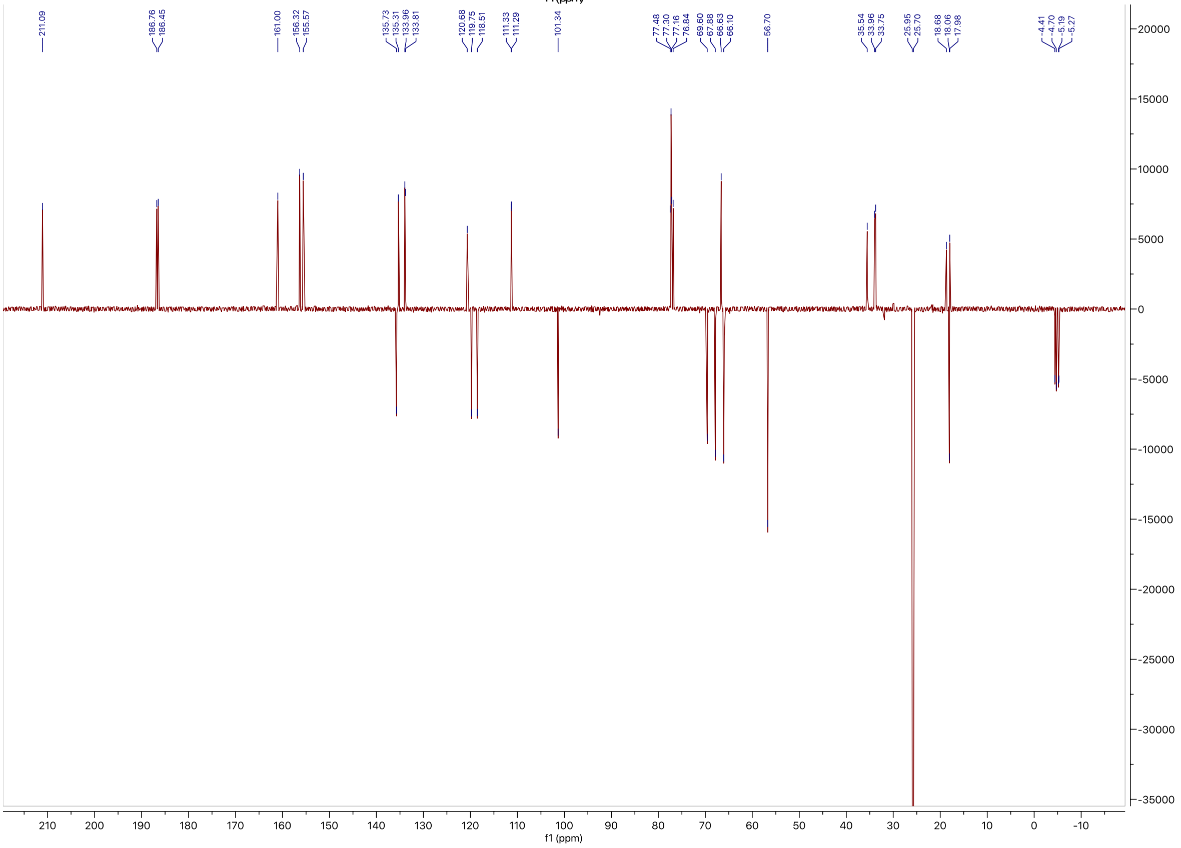
211.09
186.76
186.45
161.00
156.32
155.57
135.73
135.31
133.96
133.81
120.68
119.75
118.51
111.33
111.29
101.34
77.48
77.30
77.16
76.84
69.60
67.88
66.63
66.10
56.70
35.54
33.96
33.75
25.95
25.70
18.68
18.06
17.98
-4.41
-4.70
-5.19
-5.27
f1 (ppm)

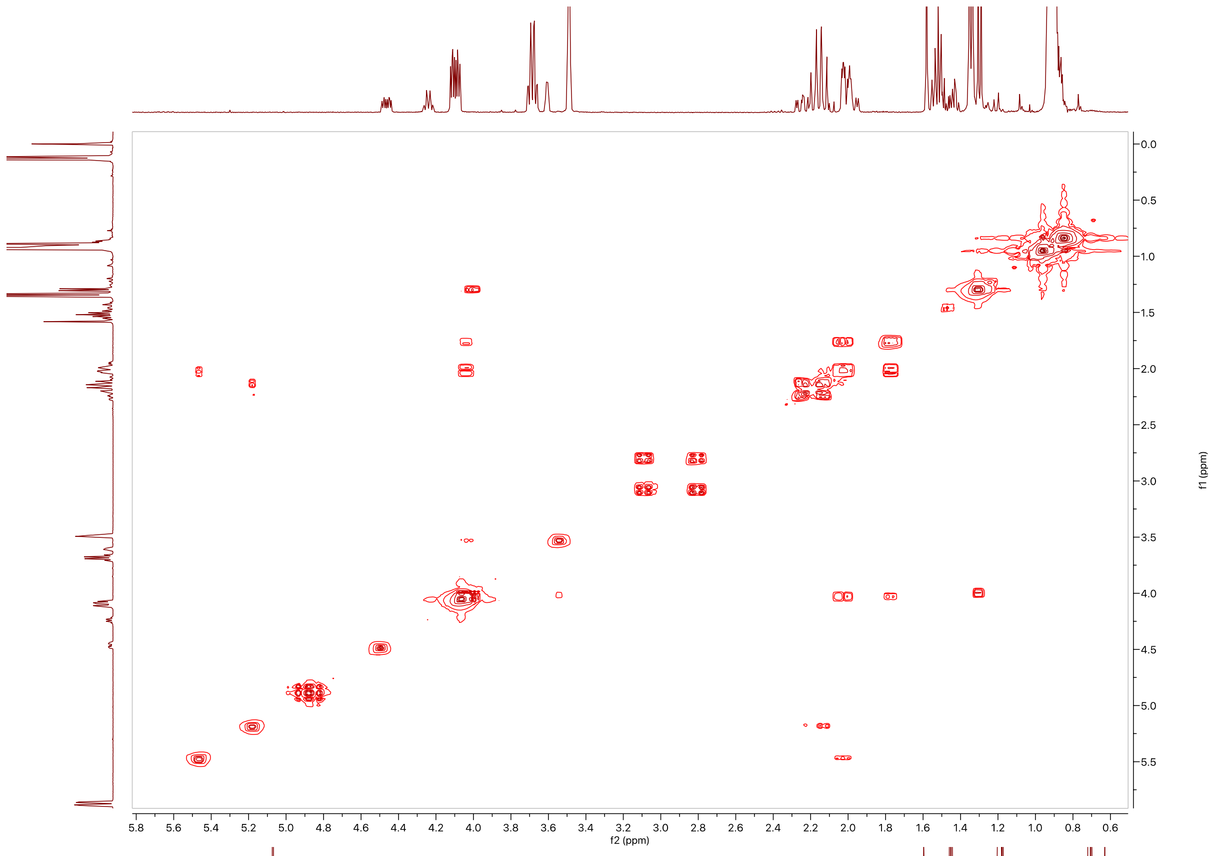
f2 (ppm)
f1 (ppm)

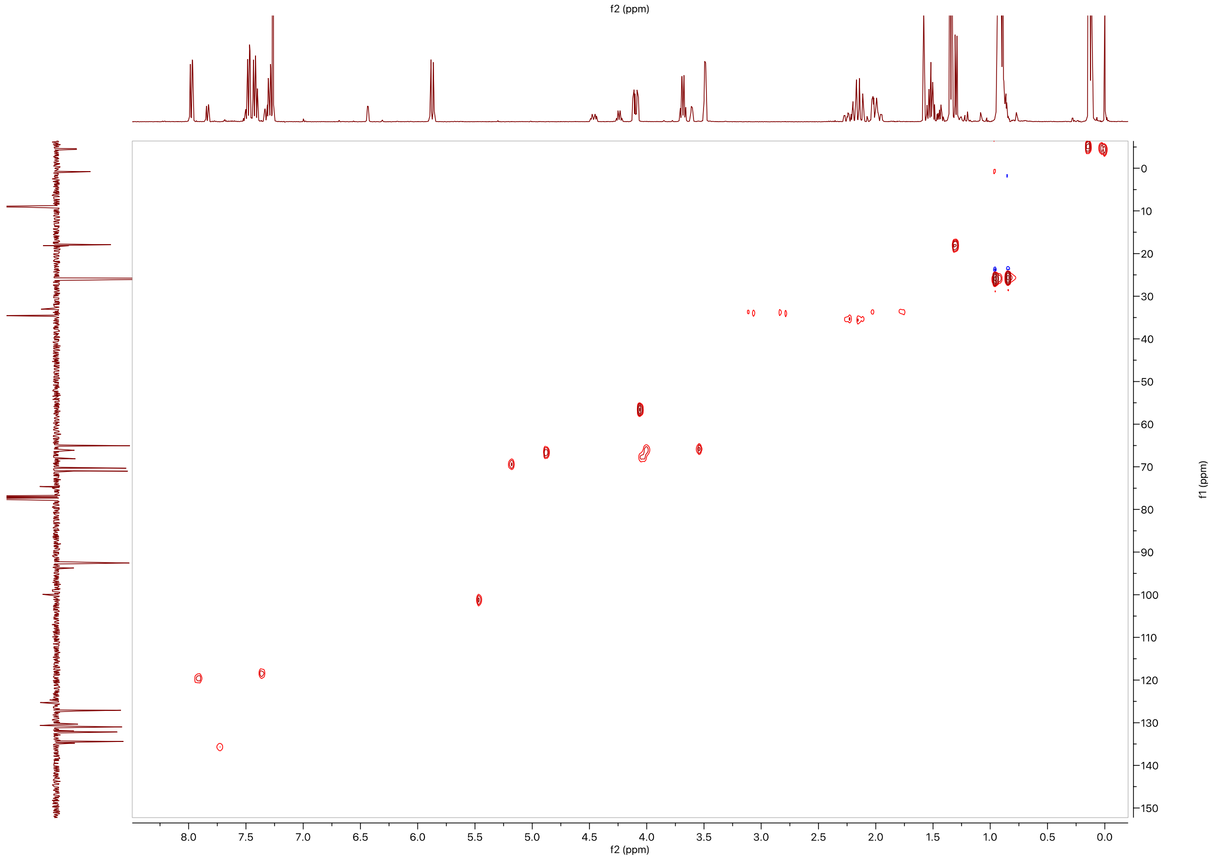
f2 (ppm)
f1 (ppm)

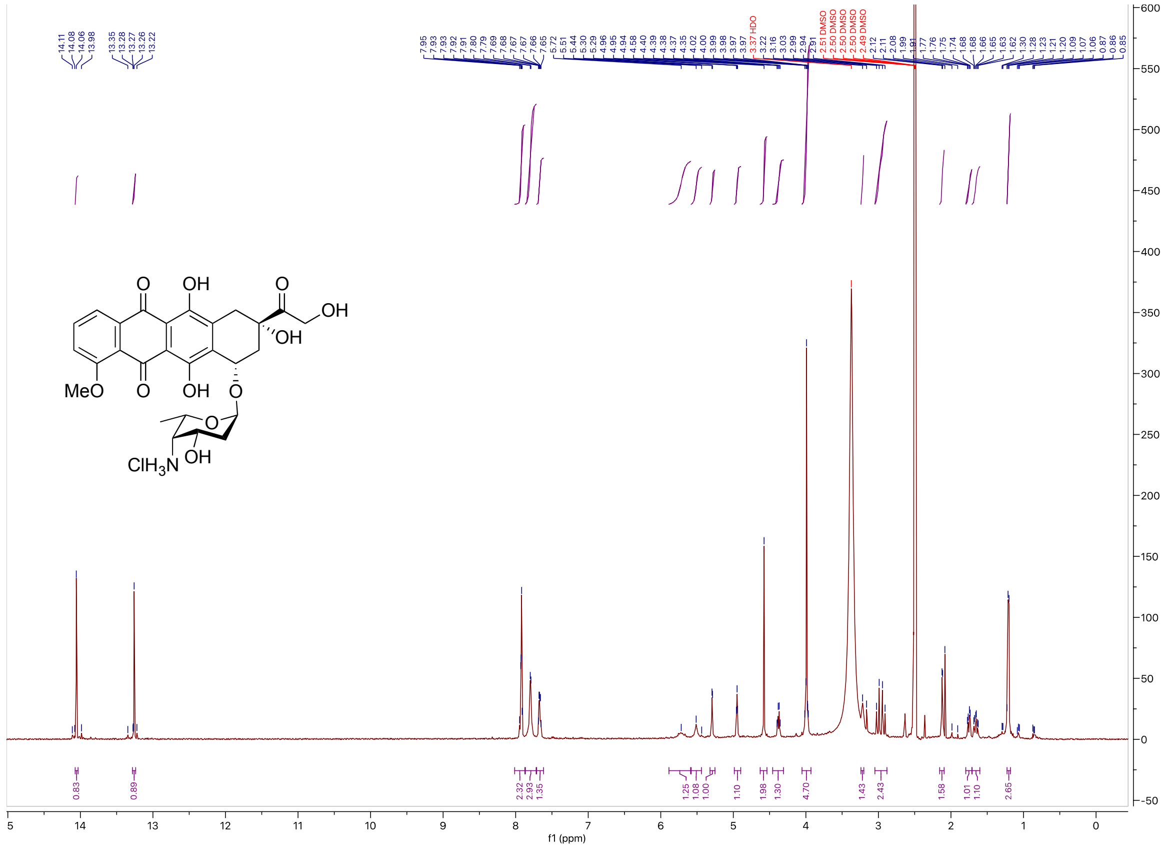

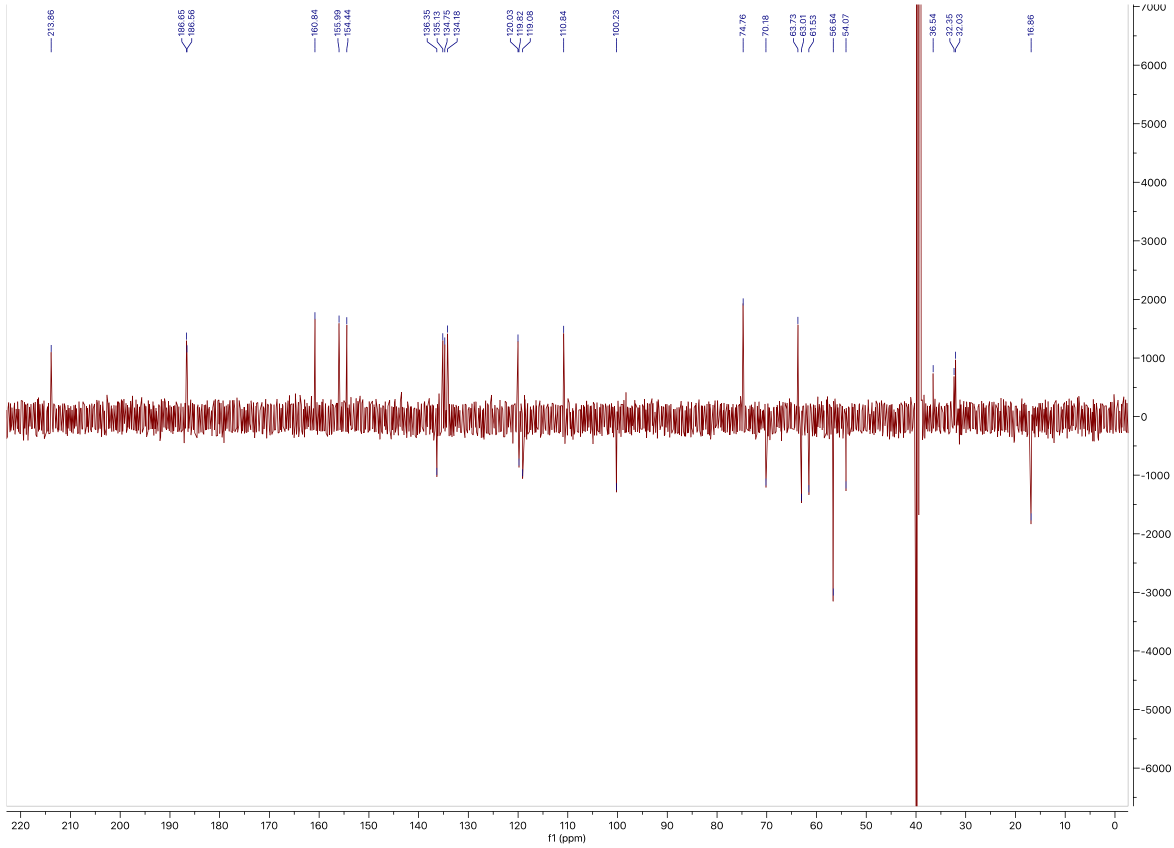

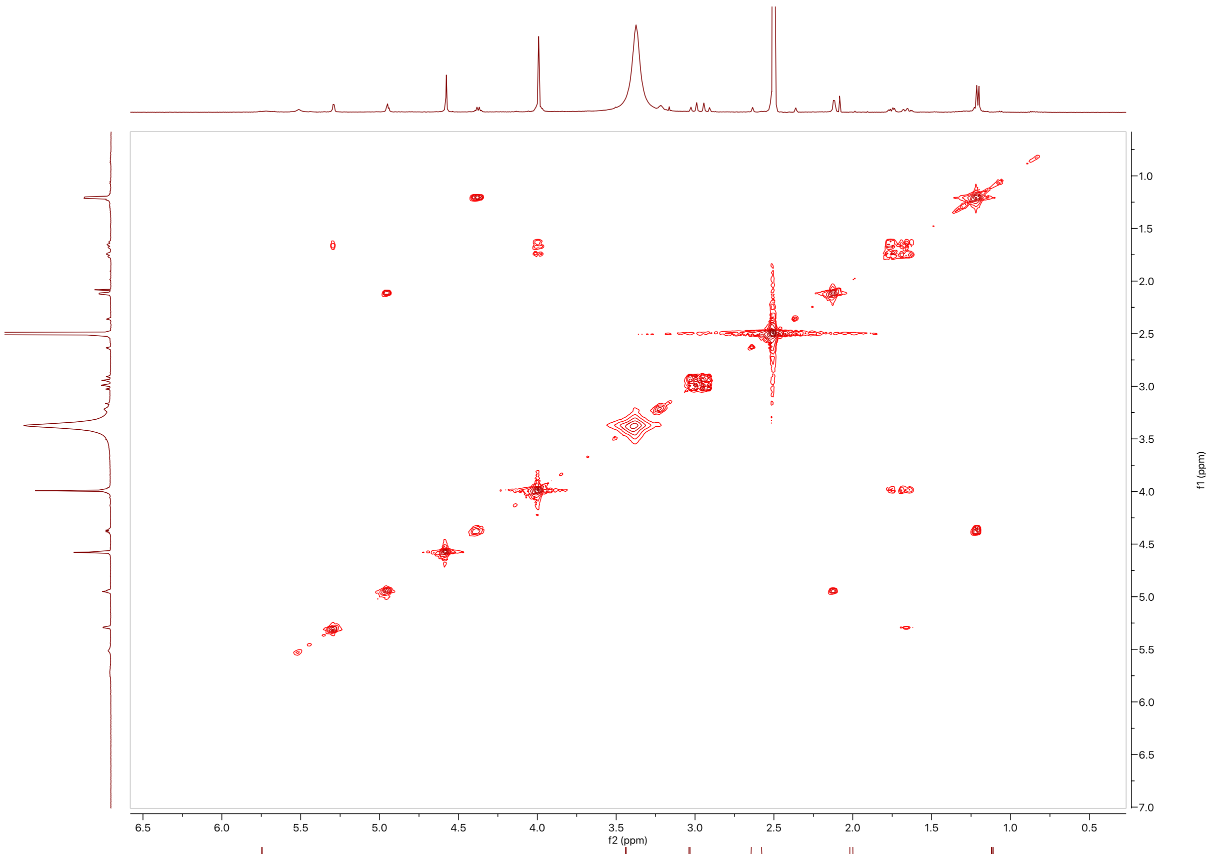
f2 (ppm)
f1 (ppm)

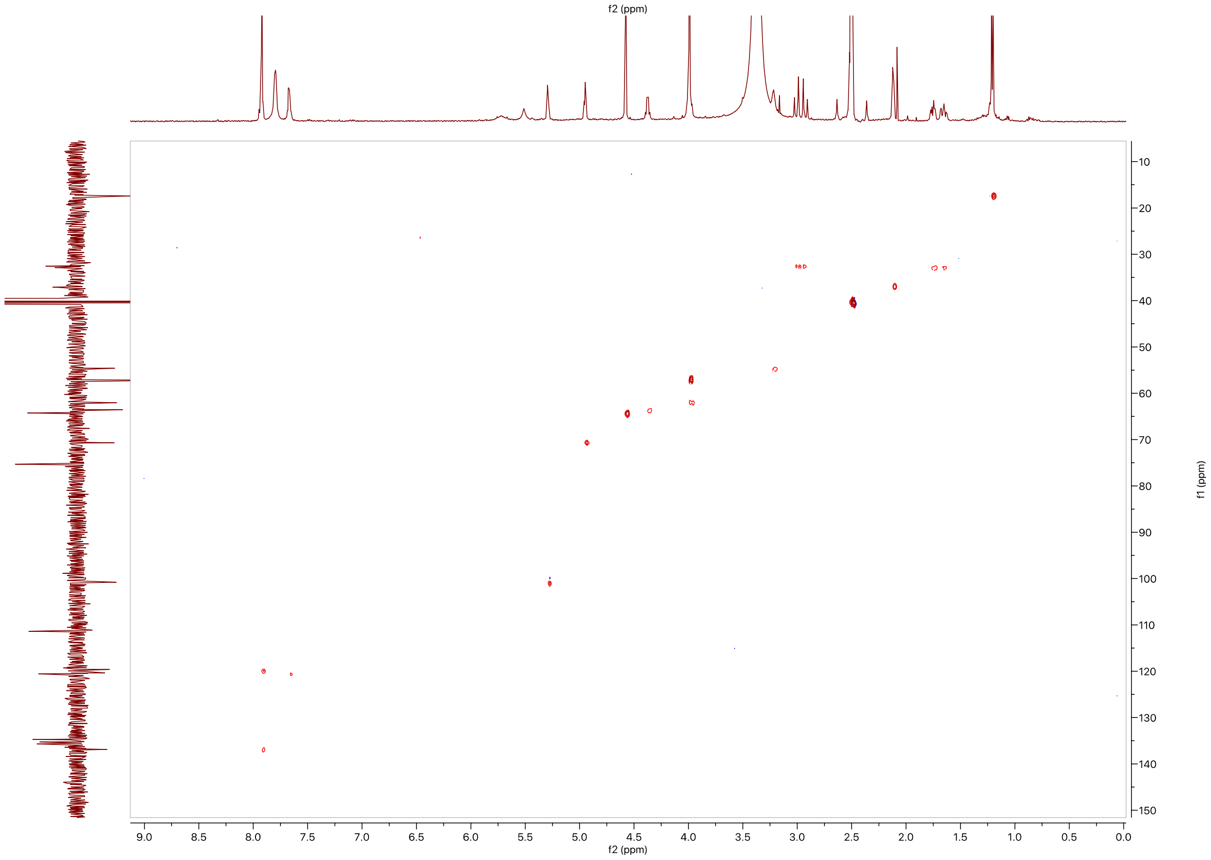
f2 (ppm)
f1 (ppm)

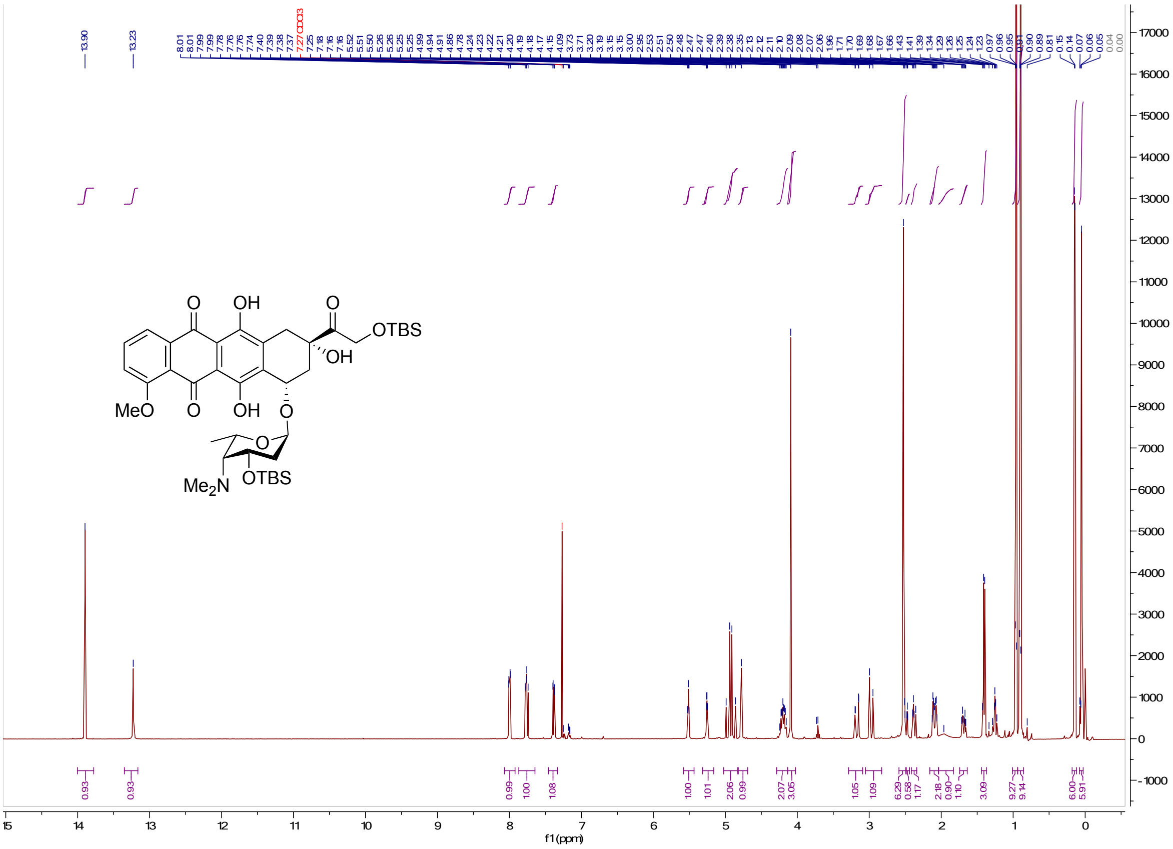
OTBS
OH
MeO
Me2N
f1 (ppm)

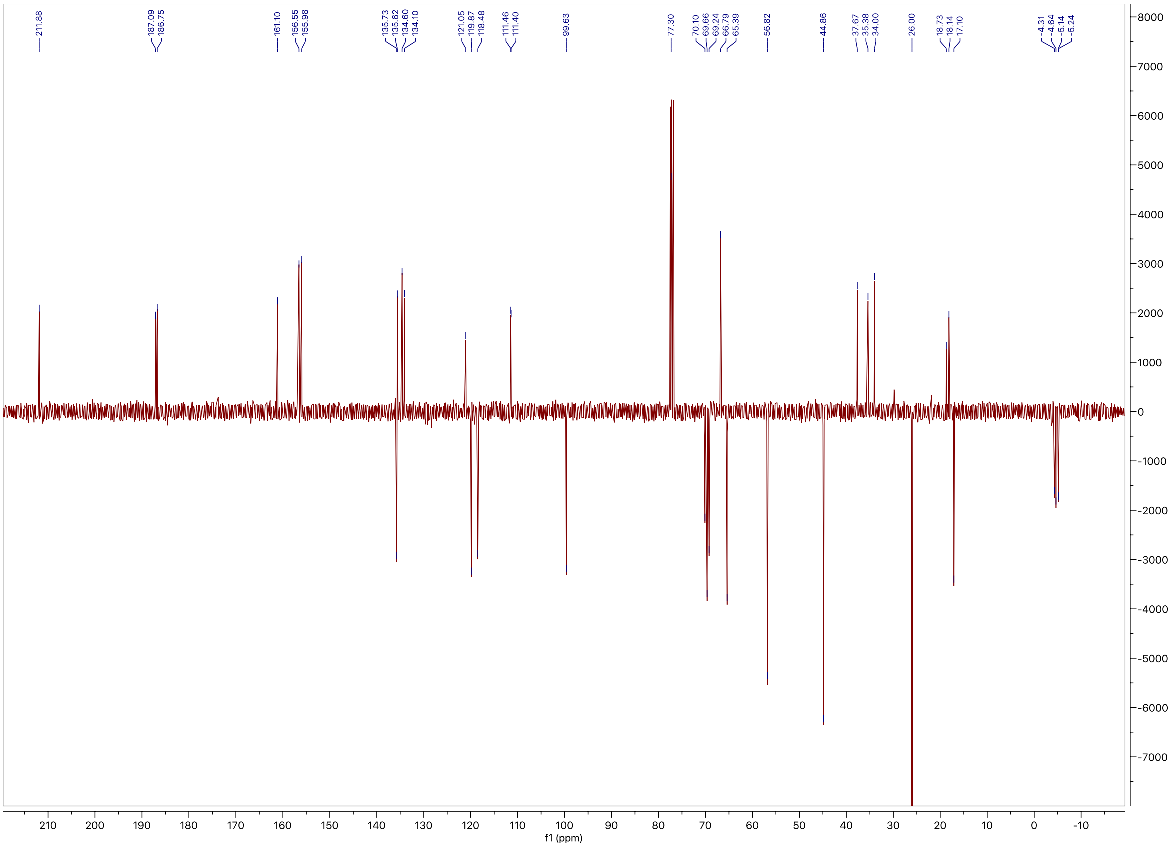
211.88
187.09
186.75
161.10
156.55
155.98
135.73
135.62
134.60
134.10
121.05
119.87
118.48
111.46
111.40
99.63
77.30
70.10
69.66
69.24
66.79
65.39
56.82
44.86
37.67
35.38
34.00
26.00
18.73
18.14
17.10
-4.31
-4.64
-5.14
-5.24
f1 (ppm)

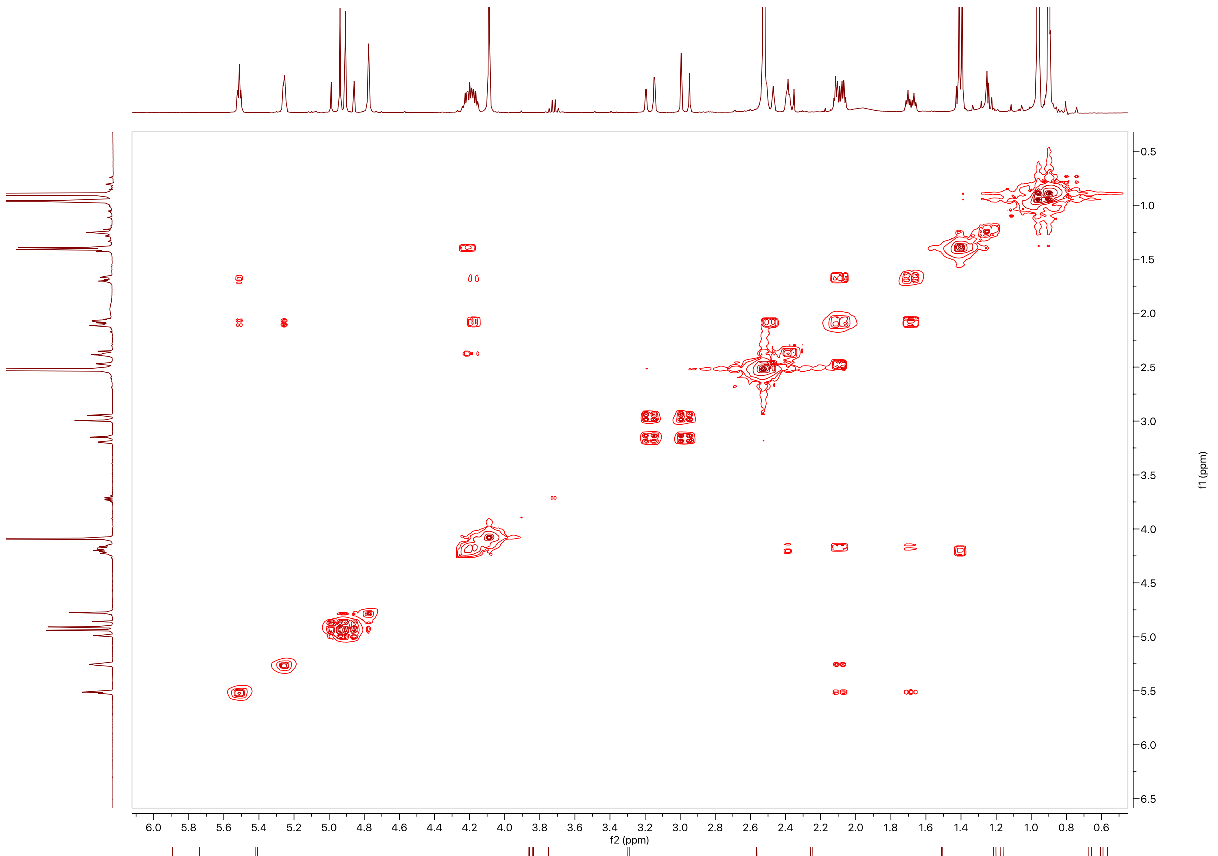
f2 (ppm)
f1 (ppm)

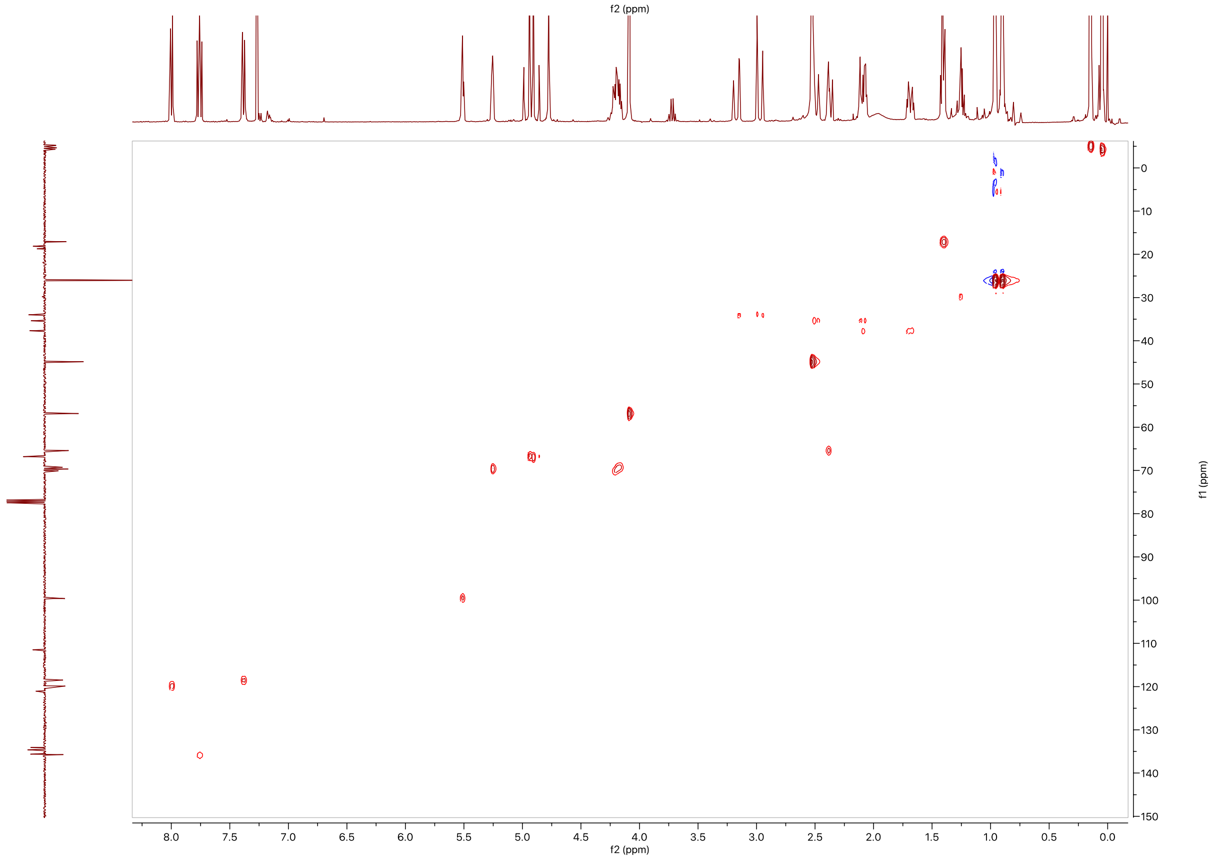
f2 (ppm)
f1 (ppm)

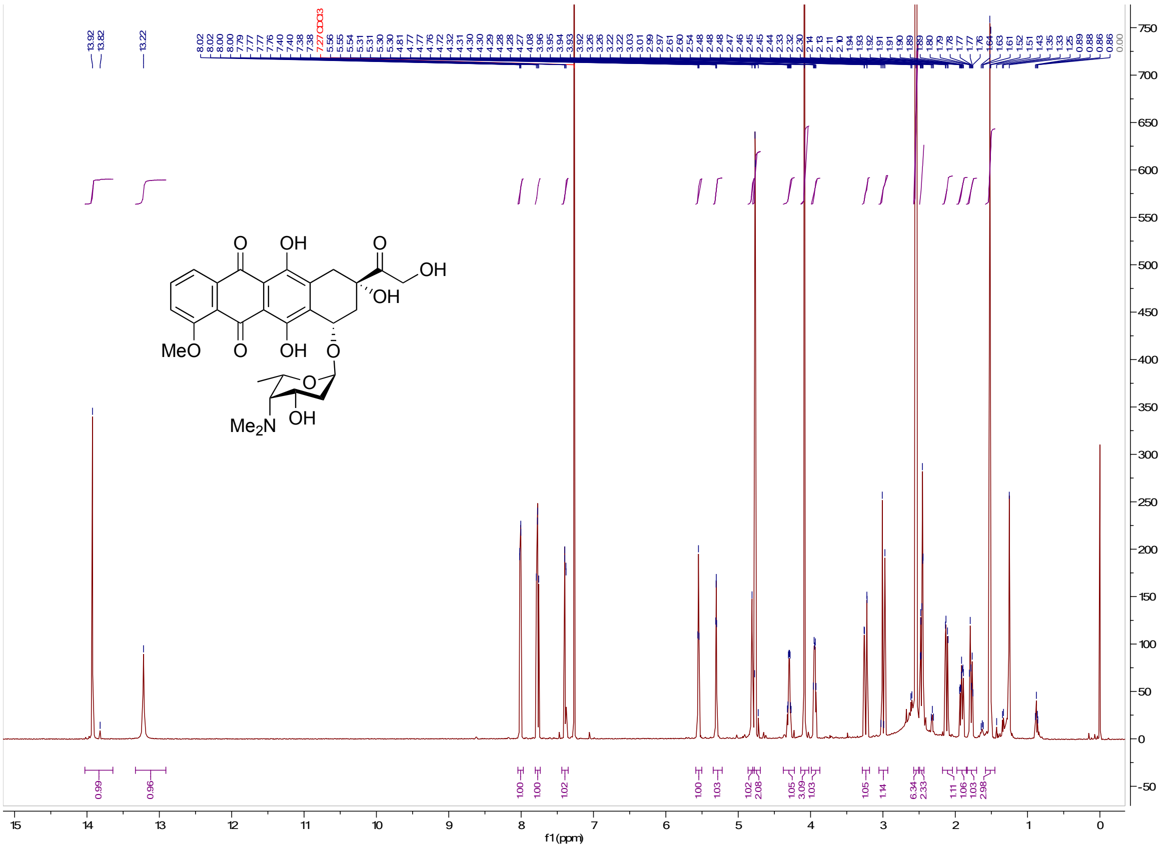
O
OH
O
OH
OH
MeO
O
OH
O
O
Me2N
OH
f1 (ppm)

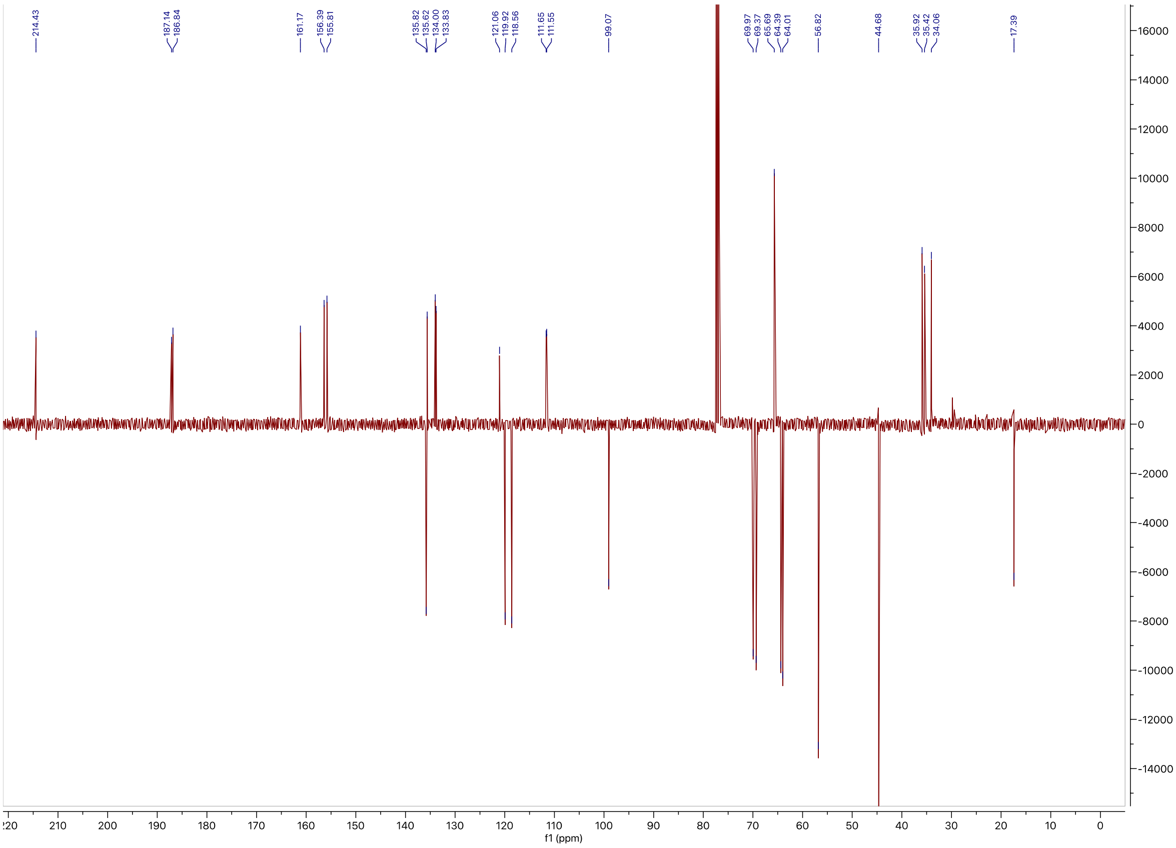
214.43
187.14
186.84
161.17
156.39
155.81
135.82
135.62
134.00
133.83
121.06
119.92
118.56
111.65
111.55
99.07
69.97
69.37
65.69
64.39
64.01
56.82
44.68
35.92
35.42
34.06
17.39
f1 (ppm)

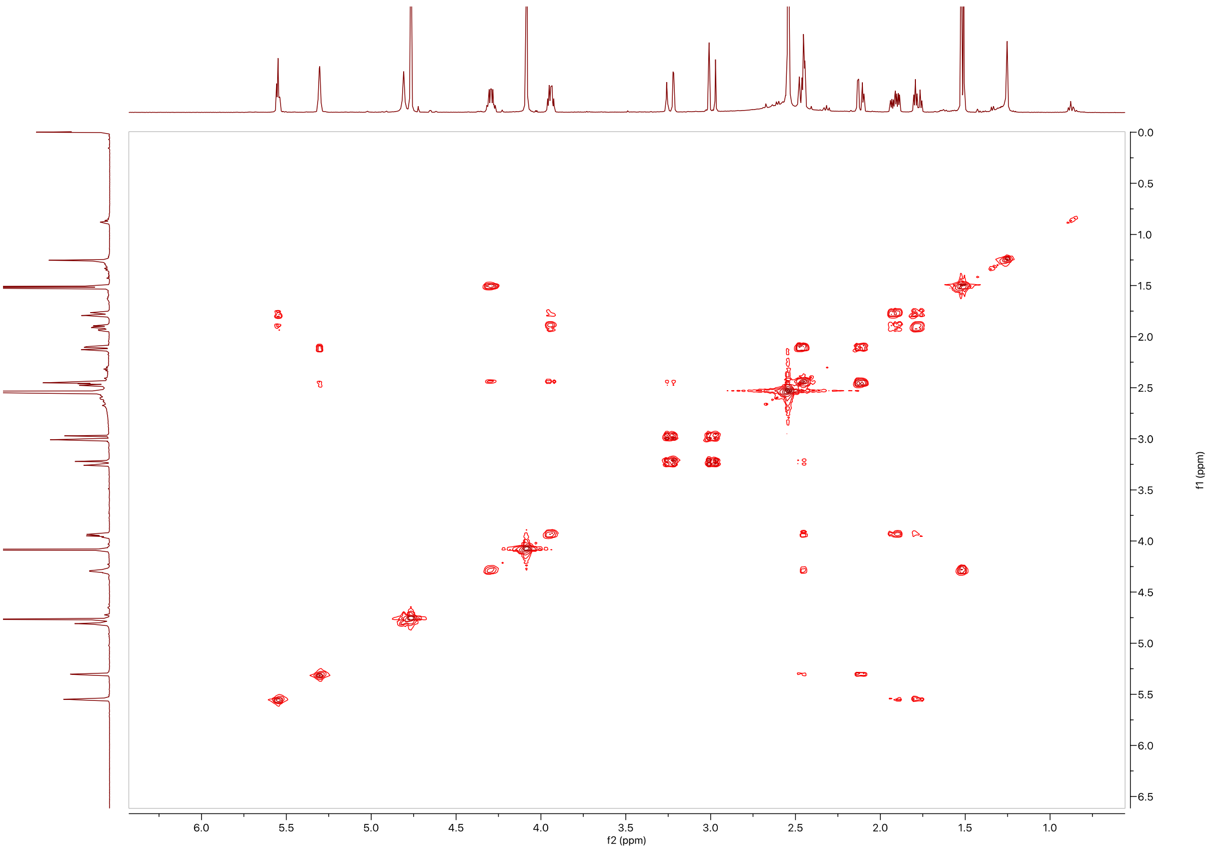
f2 (ppm)
6.0 5.5 5.0 4.5 4.0 3.5 3.0 2.5 2.0 1.5 1.0
f1 (ppm)
0.0 0.5 1.0 1.5 2.0 2.5 3.0 3.5 4.0 4.5 5.0 5.5 6.0 6.5

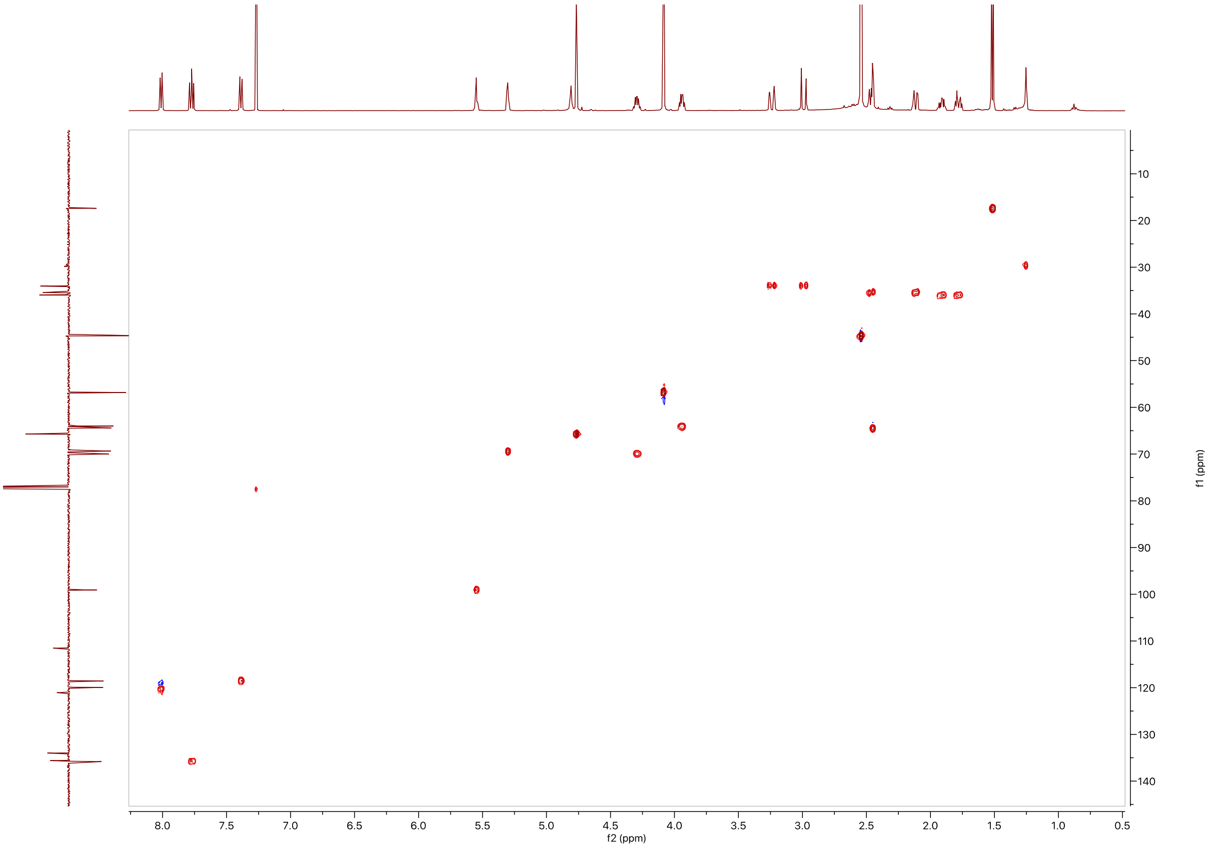
f2 (ppm)
8.0 7.5 7.0 6.5 6.0 5.5 5.0 4.5 4.0 3.5 3.0 2.5 2.0 1.5 1.0 0.5
f1 (ppm)
10 20 30 40 50 60 70 80 90 100 110 120 130 140

C: Synthesis of (*N,N*-dimethyl) doxorubicin derivatives 15-24, differing in the aglycone part and accompanying analytical data

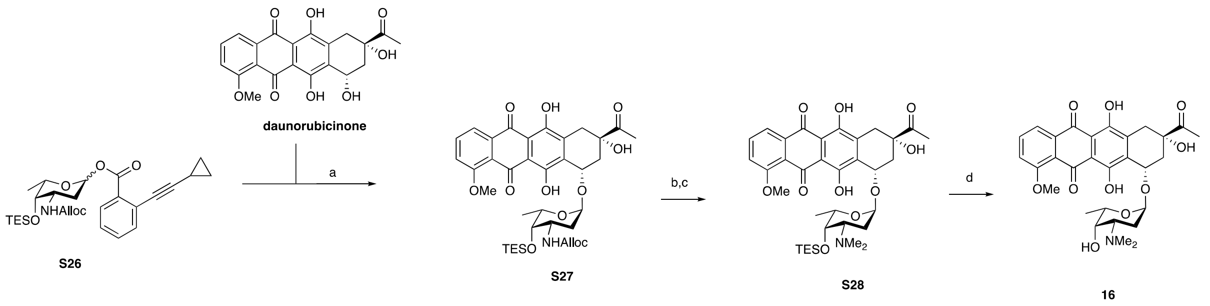

Scheme S4. Synthesis of *N,N*-dimethyldaunorubicin (**16**). *Reagents and conditions:* (a) idarubicinone, PPh_3AuNTf_2, DCM, 70%; (b) $Pd(PPh_3)_4$, NDMBA, DCM, 84%; (c) aq. CH_2O, $NaBH(OAc)_3$, EtOH, 37% (53% BRSM); (d) HF·pyridine, pyridine, 81%.

7-[3-*N*-allyloxycarbonyl-2,3-dideoxy-α-L-fucopyranoside]-daunomycinone (S27)

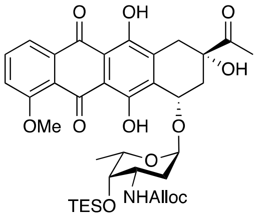

According to general procedure C, glycosyl donor **S26**[4] (256 mg, 0.500 mmol) was glycosylated to commercially available daunorubicinone (179 mg, 0.750 mmol, 1.5 eq). Column chromatography (5:95 EtOAc:pentane and then 2:98 – 4:96 acetone:toluene) of the residue gave the title compound as a red solid (255 mg, 0.351 mmol, 70%). ^{1}H NMR (500 MHz, Chloroform-*d*) δ 13.85 (s, 1H), 13.12 (s, 1H), 7.93 (d, *J* = 7.7 Hz, 1H), 7.72 (t, *J* = 8.0 Hz, 1H), 7.34 (d, *J* = 8.5 Hz, 1H), 5.86 (ddt, *J* = 16.4, 10.8, 5.6 Hz, 1H), 5.50 (d, *J* = 3.9 Hz, 1H), 5.33 – 5.12 (m, 3H), 4.67 (d, *J* = 8.6 Hz, 1H), 4.54 (s, 1H), 4.54 – 4.44 (m, 2H), 4.13 – 4.01 (m, 4H), 3.88 (tt, *J* = 12.9, 10.0, 4.7 Hz, 1H), 3.83 – 3.79 (m, 1H), 3.12 (dd, *J* = 18.6, 2.1 Hz, 1H), 2.79 (d, *J* = 18.7 Hz, 1H), 2.41 (s, 3H), 2.36 – 2.26 (m, 1H), 2.09 (dd, *J* = 14.7, 4.2 Hz, 1H), 1.92 (td, *J* = 12.6, 4.1 Hz, 2H), 1.68 (dd, *J* = 12.8, 4.4 Hz, 1H), 1.24 (d, *J* = 6.5 Hz, 3H), 1.00 (t, *J* = 8.0 Hz, 9H), 0.66 (qd, *J* = 7.8, 2.9 Hz, 6H). ^{13}C NMR (126 MHz, $CDCl_3$) δ 212.4, 186.8, 186.4, 161.0, 156.5, 155.8, 155.2, 135.6, 135.4, 134.5, 134.3, 132.9, 120.8, 119.8, 118.4, 117.7, 111.3, 111.1, 100.9, 71.0, 69.9, 67.7, 65.6, 56.7, 47.5, 35.0, 33.4, 30.0, 25.0, 17.6, 7.1, 5.3. HRMS: $[M + Na]^+$ calculated for $C_{37}H_{47}NO_{12}Si$ 748.2765, found 748.2776.

7-[3-*N,N*-dimethyl-2,3-dideoxy-α-L-fucopyranoside]-daunomycinone (S28)

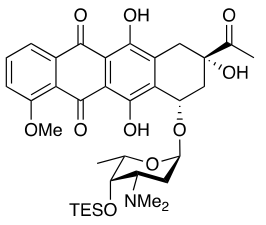

According to general procedure D, **S27** (245 mg, 0.338 mmol) was deprotected. Column chromatography (pentane – toluene – 5:95 – 20:80 acetone:toluene) gave the crude amine (182 mg, 0.283 mmol, 84%). To a solution of the above crude amine (139 mg, 0.217 mmol) in EtOH (56.0 mL) and 37% aq. CH_2O (683 µL, 38 eq) was added $NaBH(OAc)_3$ (114 mg, 0.543 mmol, 2.5 eq). The mixture was stirred for 2 hours before being poured into sat. aq. $NaHCO_3$. This was extracted with DCM, dried over Na_2SO_4 and concentrated *in vacuo*. Column chromatography (5:95 – 10:90 acetone:toluene) gave the title compound as a red solid (54 mg, 80.6 µmol, 37%). ^{1}H NMR (500 MHz, Chloroform-*d*) δ 13.86 (s, 1H), 13.18 (s, 1H), 7.96 (dd, *J* = 7.7, 1.1 Hz, 1H), 7.74 (dd, *J* = 8.5, 7.7 Hz, 1H), 7.36 (dd, *J* = 8.6, 1.1 Hz, 1H), 5.52 (d, *J* = 3.9 Hz, 1H), 5.23 (dd, *J* = 4.0, 2.1 Hz, 1H), 4.90 (s, 1H), 4.07 (s, 3H), 3.94 (q, *J* = 6.5 Hz, 1H), 3.74 (s, 1H), 3.15 (dd, *J* = 18.8, 2.0 Hz, 1H), 2.87 (d, *J* = 18.7 Hz, 1H), 2.42 (s, 3H), 2.37 (dt, *J* = 14.7, 2.2 Hz, 1H), 2.17 – 2.09 (m, 7H), 2.06 (dd, *J* = 14.7, 4.1 Hz, 1H), 1.97 (td, *J* = 12.9, 4.1 Hz, 1H), 1.69 (ddt, *J* = 12.7, 4.1, 1.3 Hz, 1H), 1.26 (d, *J* = 6.4 Hz, 3H), 0.98 (t, *J* = 7.9 Hz, 9H), 0.65 (qd, *J* = 7.9, 3.3 Hz, 6H). ^{13}C NMR (126 MHz, $CDCl_3$) δ 212.1, 186.9, 186.6, 161.1, 156.6, 156.0, 135.7, 135.5, 134.7, 134.5, 120.9, 119.8, 118.4, 111.3, 111.2, 101.7, 69.7, 69.7, 69.0, 61.4, 56.7, 42.9, 34.9, 33.4, 28.0, 24.9, 17.9, 7.2, 5.6. HRMS: $[M + H]^+$ calculated for $C_{35}H_{47}NO_{10}Si$ 670.3048, found 670.3058.

***N,N*-dimethyldaunorubicin (16)**

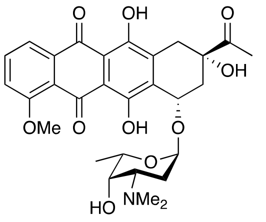

According to general procedure E, **S28** (49 mg, 0.0731 mmol) was desilylated. Column chromatography (2:98 – 10:90 MeOH:DCM) gave the title compound as a red solid (32.7 mg, 58.8 µmol, 76%). ^{1}H NMR (500 MHz, Chloroform-*d*) δ 13.94 (s, 1H), 13.21 (s, 1H), 7.99 (dd, *J* = 7.7, 1.1 Hz, 1H), 7.77 (dd, *J* = 8.5, 7.7 Hz, 1H), 7.38 (dd, *J* = 8.6, 1.1 Hz, 1H), 5.55 (t, *J* = 2.8 Hz, 1H), 5.25 (dd, *J* = 4.2, 2.1 Hz, 1H), 4.67 (s, 1H), 4.08 (s, 3H), 4.07 – 4.01 (m, 1H), 3.78 (dd, *J* = 2.7, 1.4 Hz, 1H), 3.27 (s, 1H), 3.17 (dd, *J* = 18.8, 2.1 Hz, 1H), 2.88 (d, *J* = 18.8 Hz, 1H), 2.42 (s, 3H), 2.38 (dt, *J* = 4.6, 2.5 Hz, 1H), 2.36 – 2.32 (m, 1H), 2.31 (s, 6H), 2.10 (dd, *J* = 14.8, 4.2 Hz, 1H), 1.86 (dd, *J* = 9.1, 3.2 Hz, 2H), 1.38 (d, *J* = 6.6 Hz, 3H). ^{13}C NMR (126 MHz, $CDCl_3$) δ 211.9, 187.1, 186.7, 161.1, 156.6, 155.9, 135.8, 135.6, 134.5, 134.4, 120.9, 119.9, 118.5, 111.5, 111.3, 101.0, 70.1, 66.9, 66.0, 60.2, 56.8, 42.1, 35.0, 33.3, 28.3, 24.9, 17.2. HRMS: $[M + H]^+$ calculated for $C_{29}H_{33}NO_{10}$ 556.2183, found 556.2115.

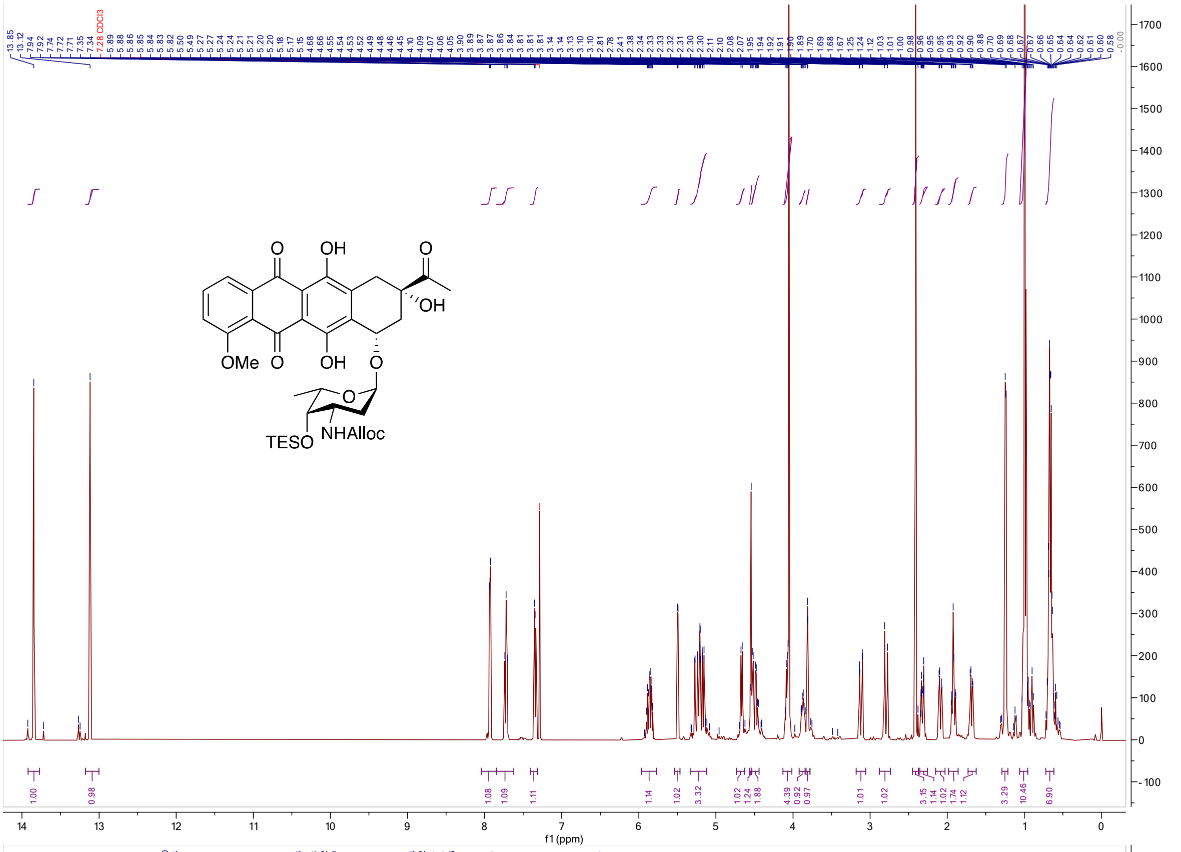
OMe
O
OH
OH
TESO
NHAlloc
f1 (ppm)

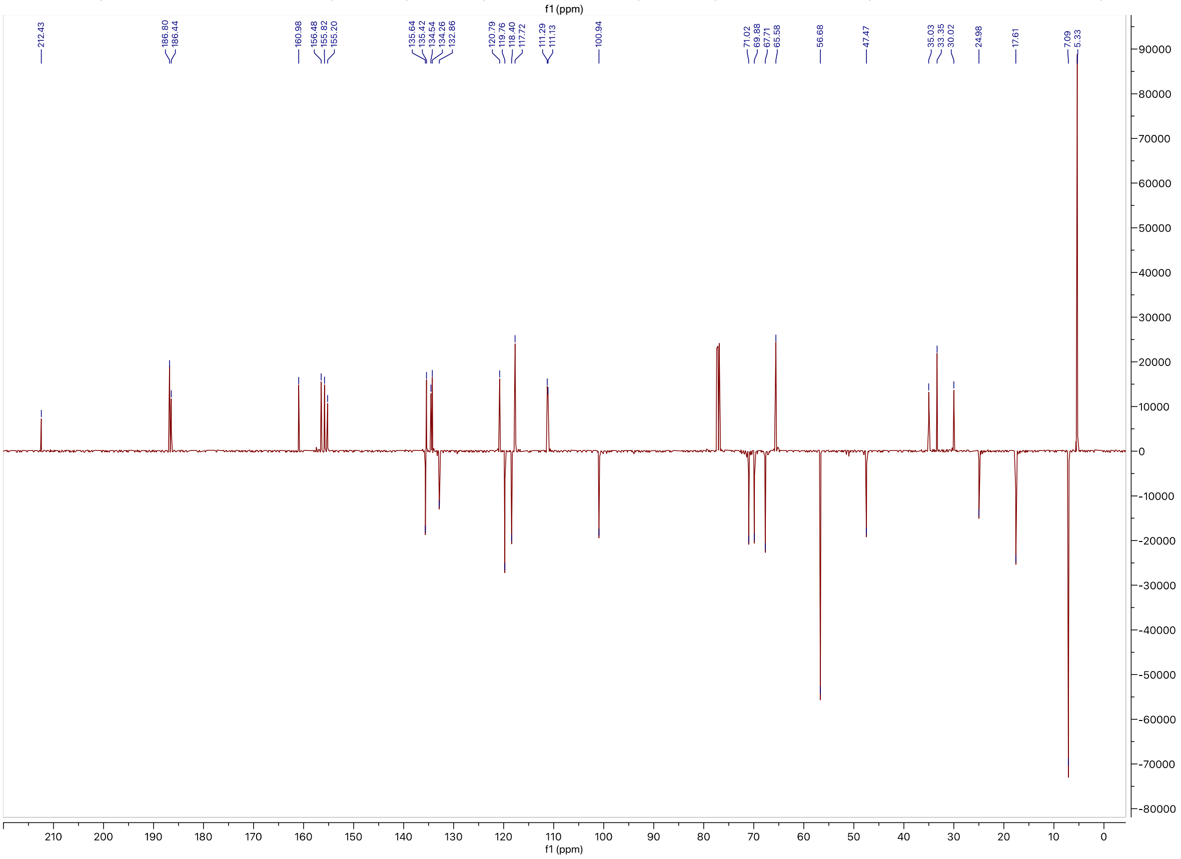
212.43
186.80
186.44
160.98
156.48
155.82
155.20
135.64
135.42
134.54
134.26
132.86
120.79
119.76
118.40
117.72
111.29
111.13
100.94
71.02
69.98
67.71
65.58
56.68
47.47
35.03
33.35
30.02
24.98
17.61
7.09
5.33
f1 (ppm)

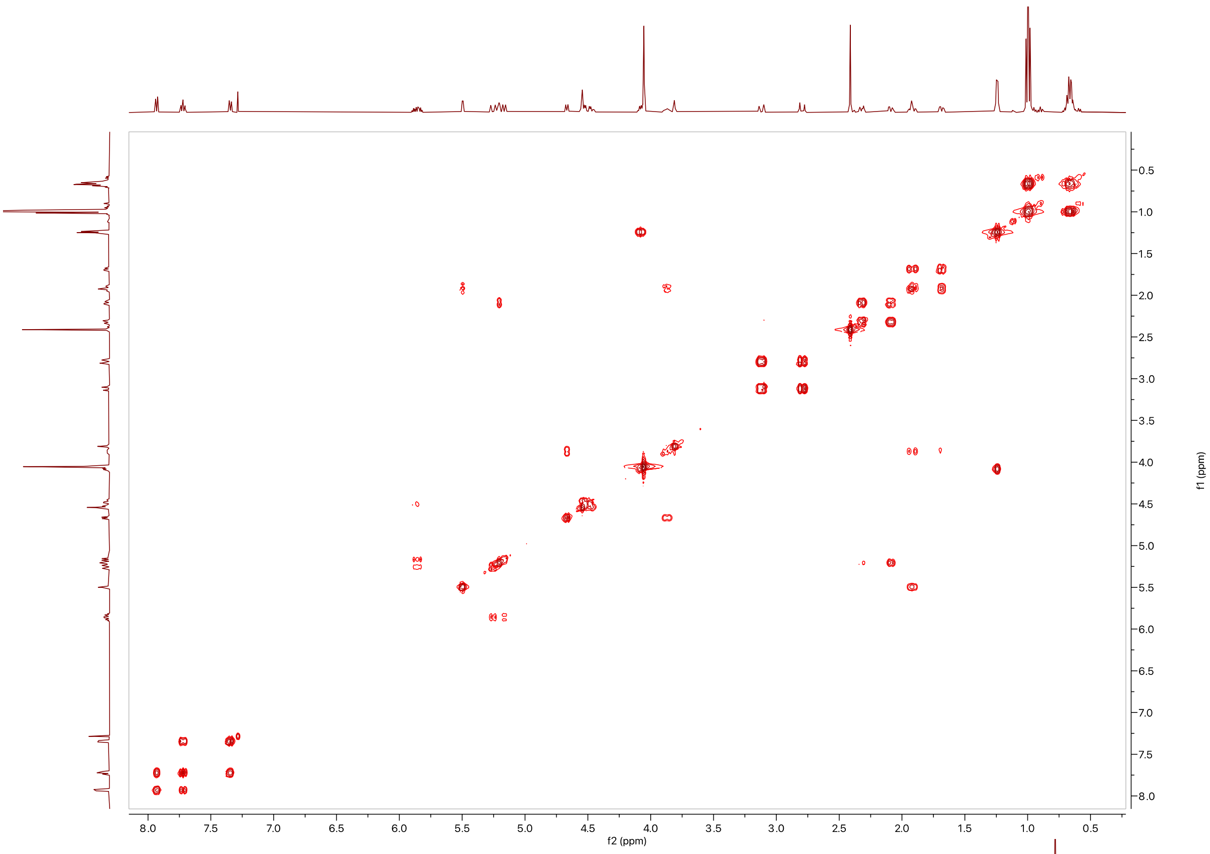
f2 (ppm)
f1 (ppm)

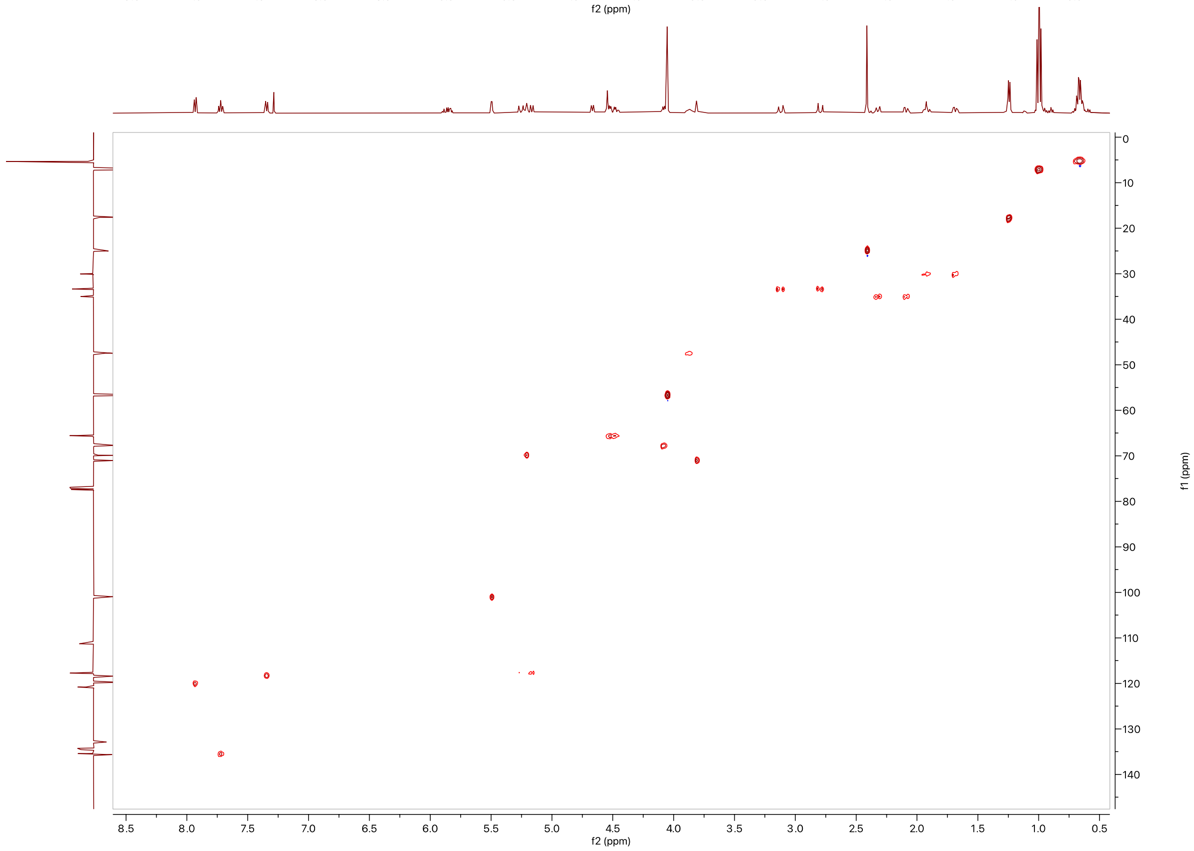
f2 (ppm)
f1 (ppm)

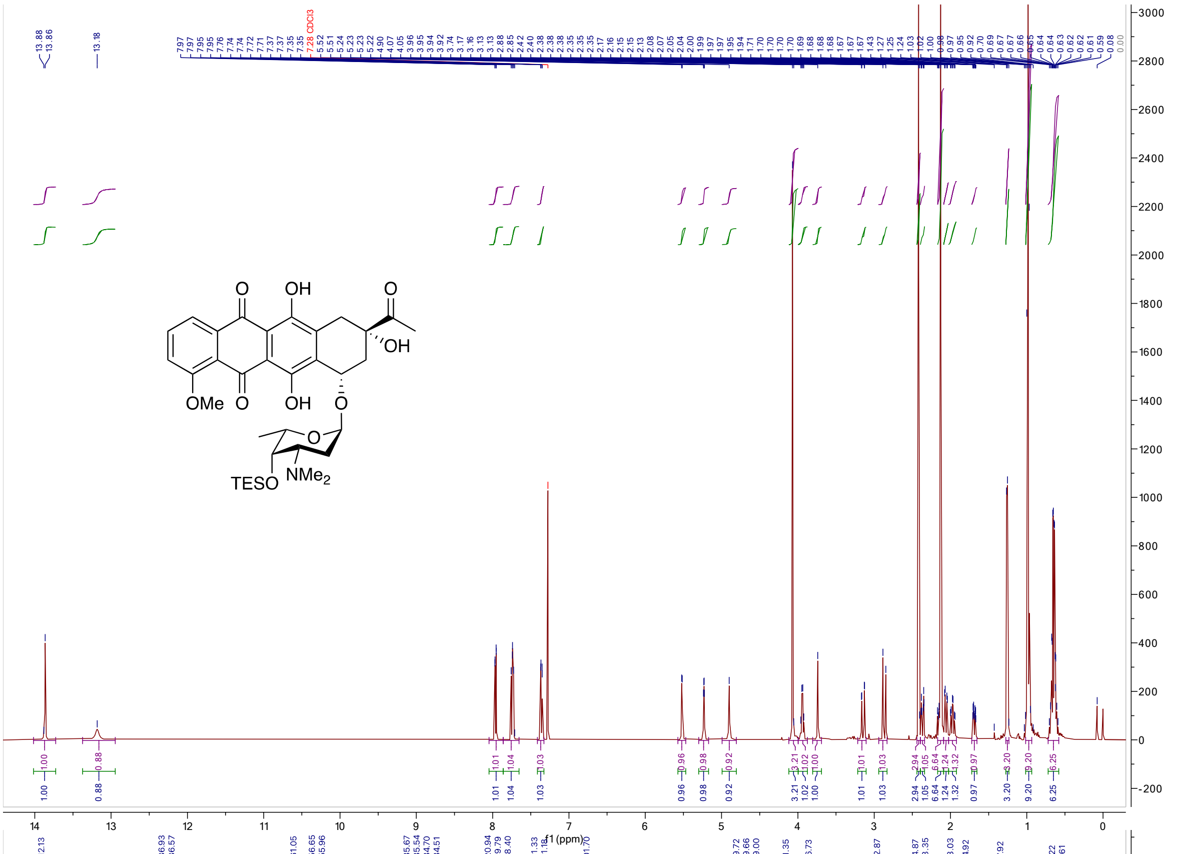
OH
O
OH
OMe
NMe2
TESO
CDCl3
f1 (ppm)

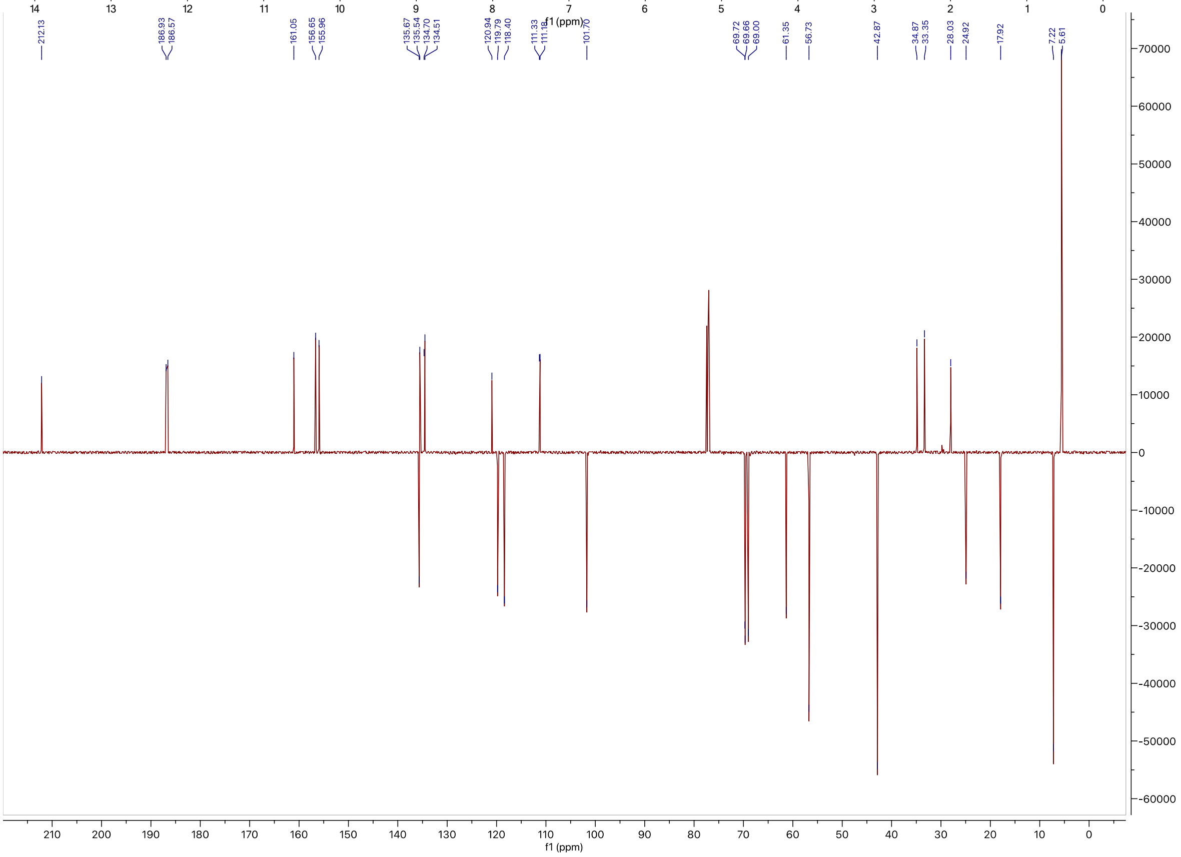
212.13
186.93
186.57
161.05
156.65
155.96
135.67
135.54
134.70
134.51
120.94
119.79
118.40
111.33
111.18
101.70
69.72
69.66
69.00
61.35
56.73
42.87
34.87
33.35
28.03
24.92
17.92
7.22
5.61
f1 (ppm)

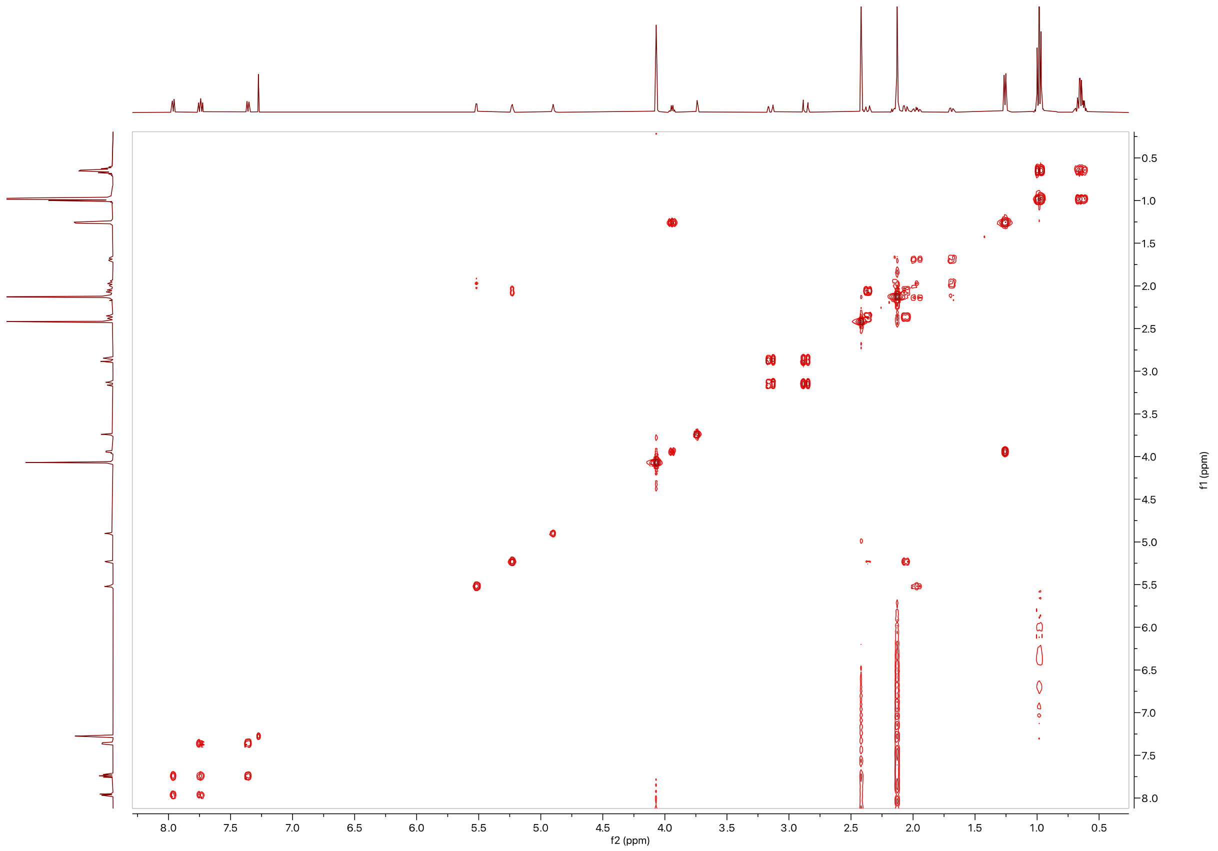
f2 (ppm)
f1 (ppm)

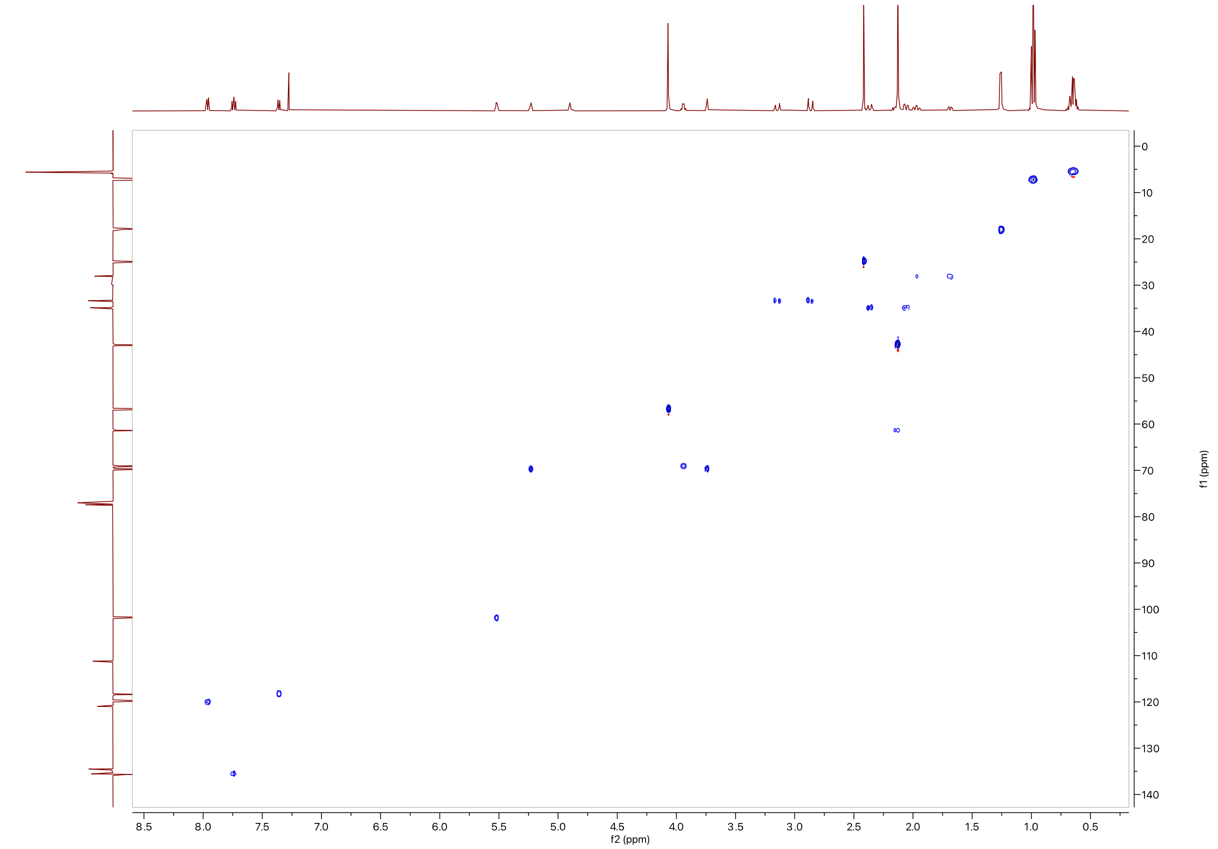
f2 (ppm)
f1 (ppm)

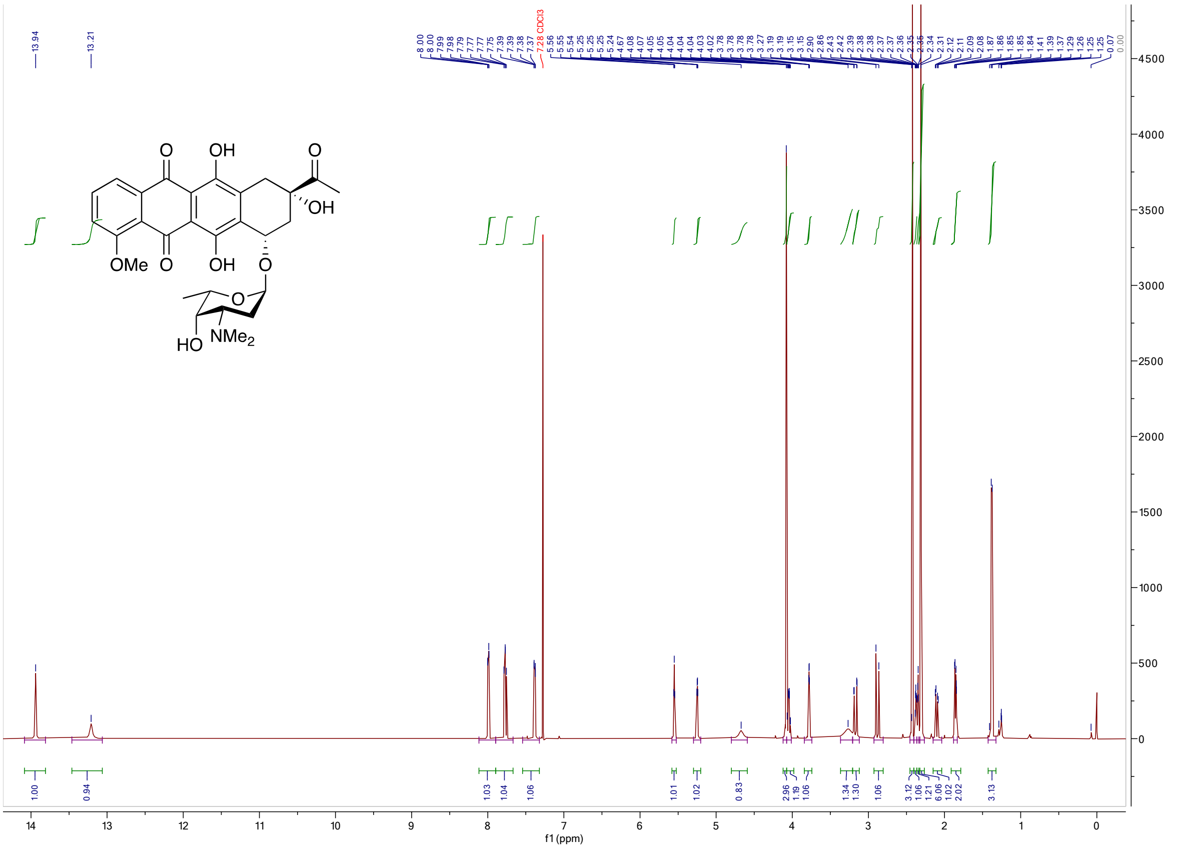

13.94
13.21
8.00 8.00 7.99 7.98 7.79 7.77 7.77 7.75 7.39 7.39 7.38 7.37
7.28 CDCl3
5.56 5.55 5.54 5.25 5.25 5.25 5.24 4.67 4.08 4.07 4.05 4.05 4.04 4.04 4.04 4.03 4.02 3.78 3.78 3.78 3.78 3.27 3.19 3.19 3.15 3.15 2.90 2.86 2.43 2.42 2.39 2.38 2.37 2.37 2.36 2.35 2.35 2.34 2.31 2.12 2.11 2.09 2.08 1.87 1.86 1.85 1.85 1.84 1.41 1.39 1.37 1.29 1.26 1.25 0.07 0.00
OH
O
OH
OMe
O
OH
O
O
HO
NMe2
1.00
0.94
1.03
1.04
1.06
1.01
1.02
0.83
2.96
1.19
1.06
1.34
1.30
1.06
3.12
1.06
1.21
6.06
1.02
2.02
3.13
f1 (ppm)

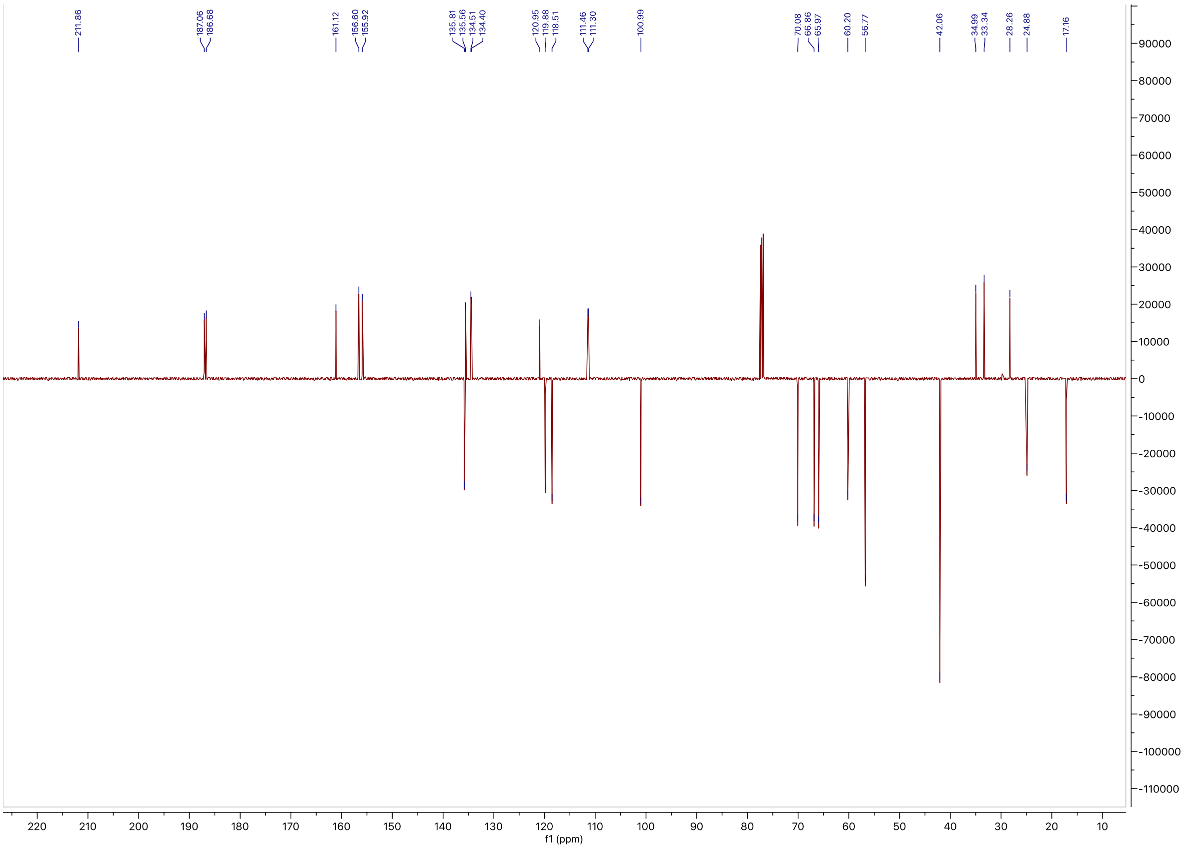

211.86
187.06
186.68
161.12
156.60
155.92
135.81
135.56
134.51
134.40
120.95
119.88
118.51
111.46
111.30
100.99
70.08
66.86
65.97
60.20
56.77
42.06
34.99
33.34
28.26
24.88
17.16
f1 (ppm)

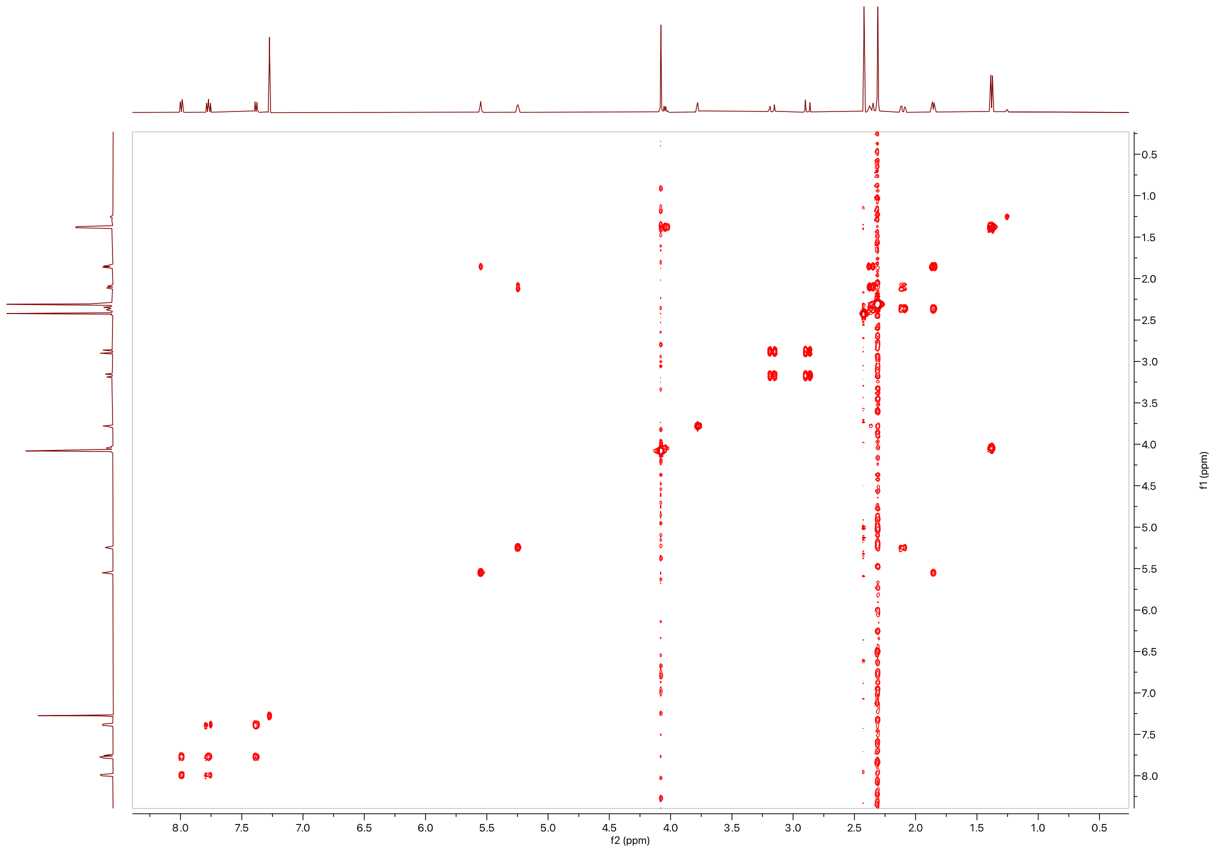
f2 (ppm)
f1 (ppm)

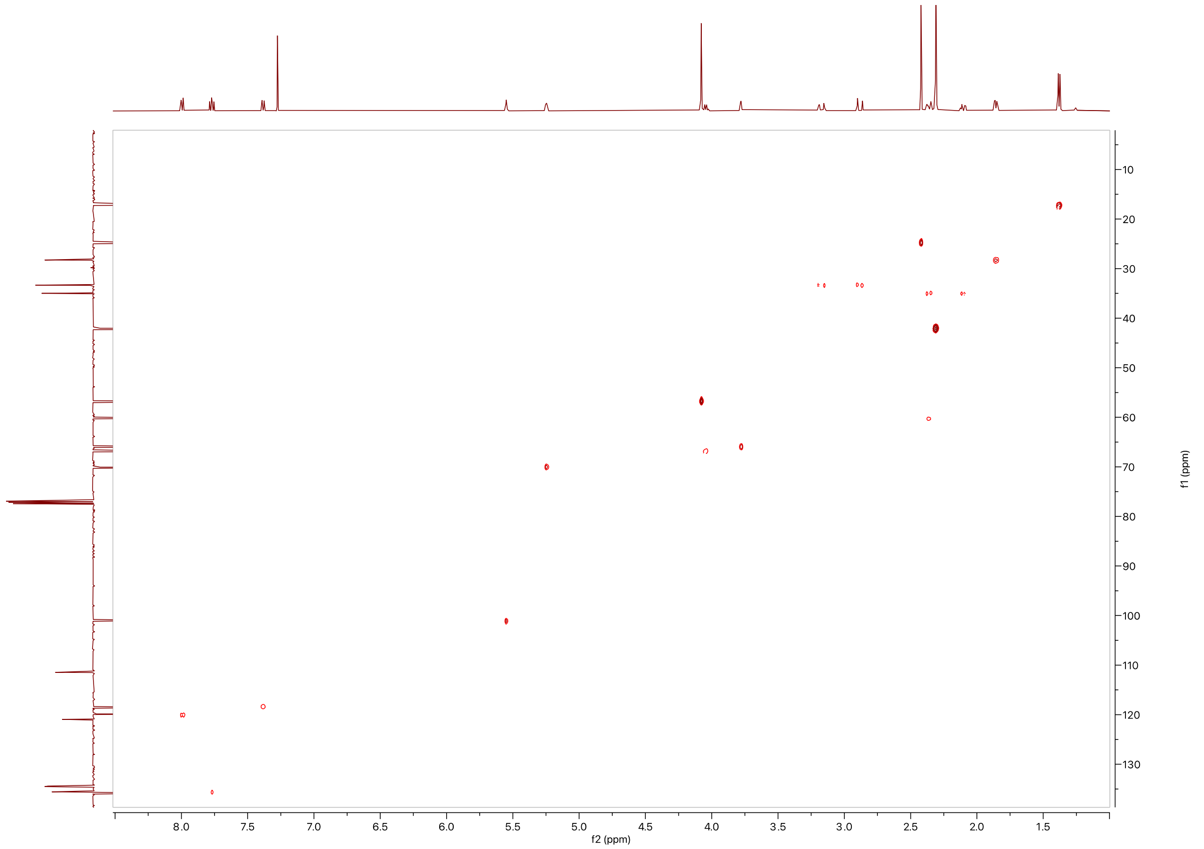
f2 (ppm)
f1 (ppm)

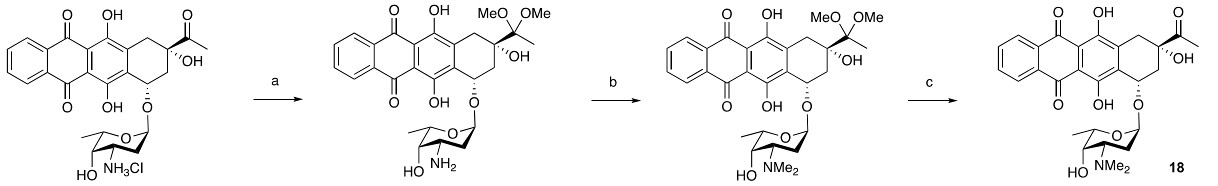

idarubicin-HCl

18

Scheme S5. Synthesis of *N,N*-dimethylidarubicin (**18**). *Reagents and conditions:* (a) (±)-camphorsulfonic acid, MeOH, $CH(OEt)_3$); (b) aq. CH_2O, $NaBH_3CN$, MeOH; (c) 0.25M aq. HBr, acetone, 37% over 3 steps.

***N,N*-dimethylidarubicin (18)**

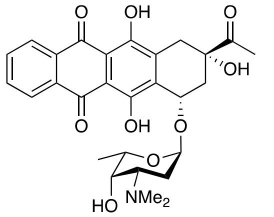

To a solution of idarubicin hydrochloride (50.0 mg, 0.0936 mmol) in MeOH (3 mL) and trimethylorthoformate (1 mL) was added (±)-camphorsulfonic acid (50 mg, 0.215 mmol, 2.3 eq) and the mixture was stirred overnight. It was then poured into sat. aq. $NaHCO_3$ (10 mL) and extracted with $CHCl_3$ (20 mL, 2x). The organic layers were dried over Na_2SO_4 and concentrated *in vacuo* to give the crude dimethyl acetal as a yellow solid.

This was redissolved in a mixture of MeOH (3 mL), DCM (2 mL) and 37% aq. CH_2O (stabilised with 10-15% MeOH), after which $NaBH_3CN$ (29.3 mg, 0.47 mmol, 5 eq) was added. After stirring for 2 hours, the reaction was poured into sat. aq. $NaHCO_3$ (10 mL). This was then extracted with $CHCl_3$ (20 mL), dried over Na_2SO_4 and concentrated *in vacuo*. This procedure was performed twice to afford the crude dimethylamine as a yellow solid.

A solution of the above acetal in acetone (8 mL) and 0.25M aq. HBr (8 mL) was stirred for 1 hour, after which it was diluted with H_2O (20 mL). This was then washed with DCM (30 mL, 3x) basified by addition of sat. aq. $NaHCO_3$ (10 mL) and extracted with $CHCl_3$ (30 mL, 2x). Drying over Na_2SO_4 and concentration *in vacuo* afforded the title compound as a yellow solid (18 mg, 34 umol, 37% over 3 steps). ^{1}H NMR (400 MHz, $CDCl_3$) δ 14.05 – 12.81 (m, 2H), 8.32 (ddd, *J* = 6.3, 5.0, 3.3 Hz, 2H), 7.87 – 7.80 (m, 2H), 5.66 – 5.47 (m, 1H), 5.27 (dd, *J* = 4.1, 2.1 Hz, 1H), 4.77 (s, 1H), 4.09 – 4.02 (m, 1H), 3.76 – 3.70 (m, 1H), 3.29 – 2.94 (m, 2H), 2.44 (s, 3H), 2.38 (dt, *J* = 14.8, 2.2 Hz, 1H), 2.24 (s, 6H), 2.10 (dd, *J* = 14.8, 4.2 Hz, 1H), 1.92 – 1.76 (m, 2H), 1.40 (d, *J* = 6.6 Hz, 3H). ^{13}C NMR (101 MHz, $CDCl_3$) δ 211.8, 186.9, 186.7, 156.8, 156.5, 136.1, 134.7, 134.0, 133.6, 133.4, 127.2, 127.1, 111.5, 110.8, 101.1, 77.5, 77.2, 77.0, 76.8, 69.8, 66.8, 66.1, 59.8, 42.1, 35.0, 33.7, 29.8, 28.7, 24.9, 17.2. HRMS: $[M + H]^+$ calculated for $C_{28}H_{31}NO_9$ 526.2077; found 526.2069.

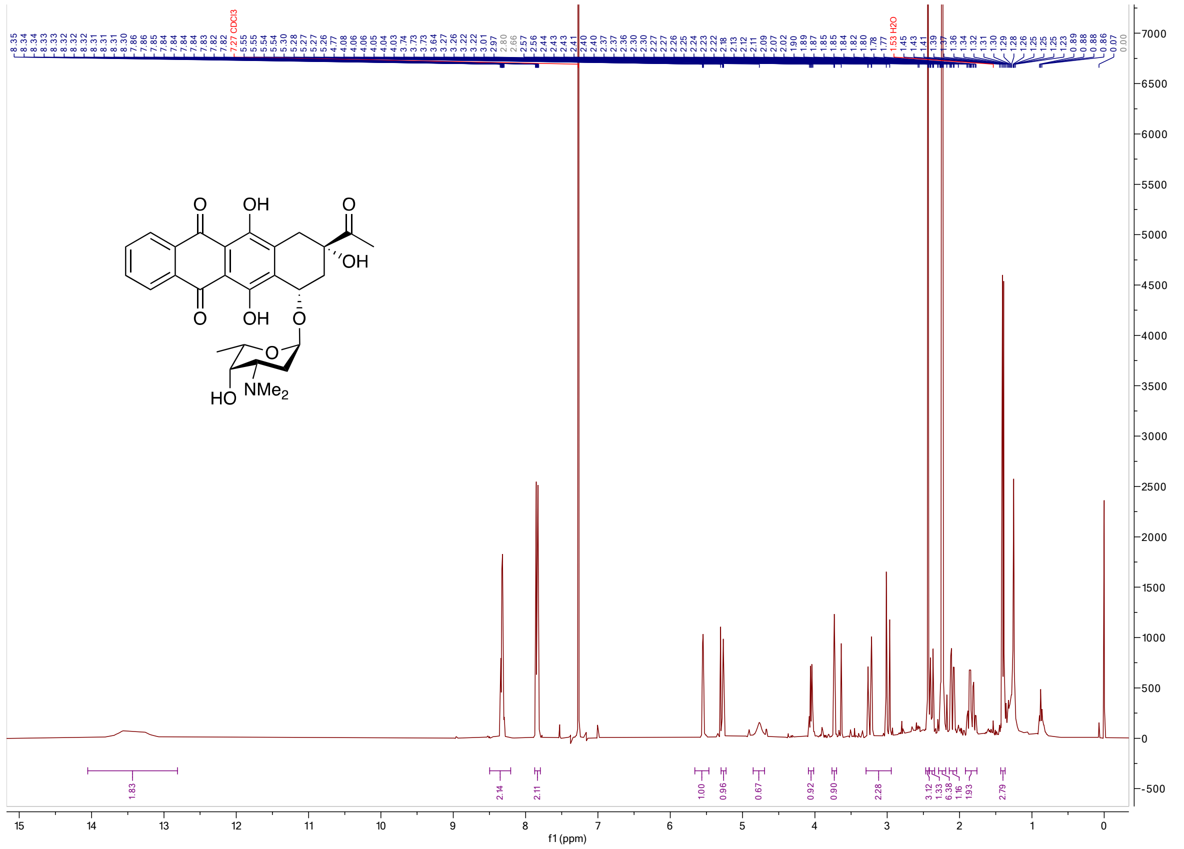

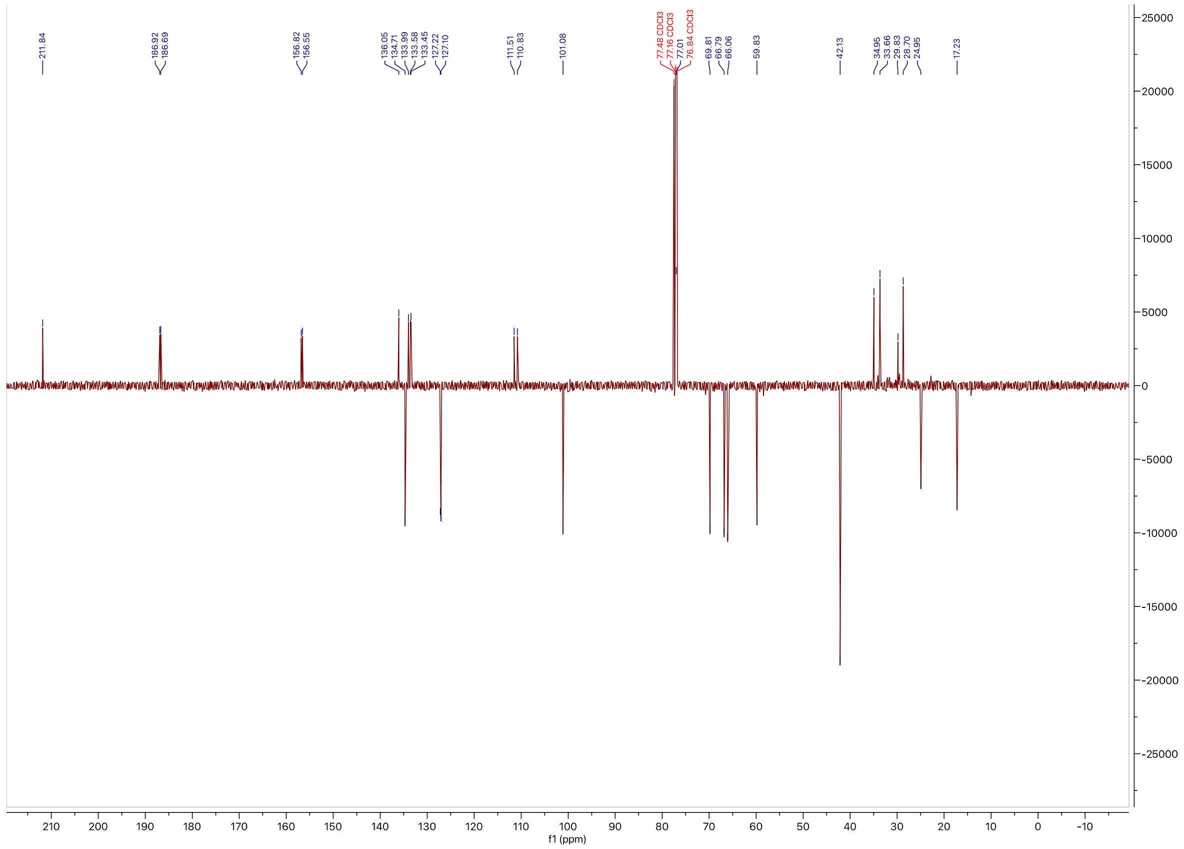

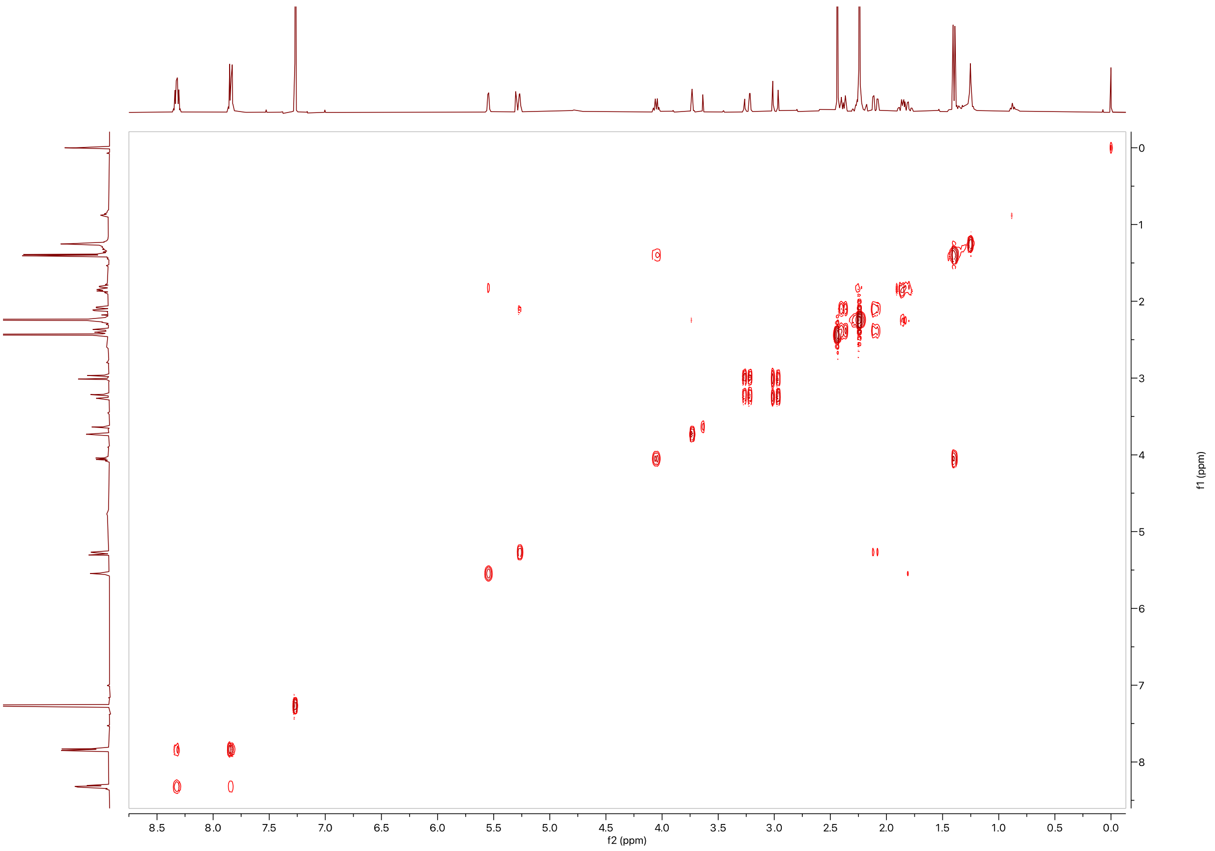
8.5
8.0
7.5
7.0
6.5
6.0
5.5
5.0
4.5
4.0
3.5
3.0
2.5
2.0
1.5
1.0
0.5
0.0
f2 (ppm)
f1 (ppm)

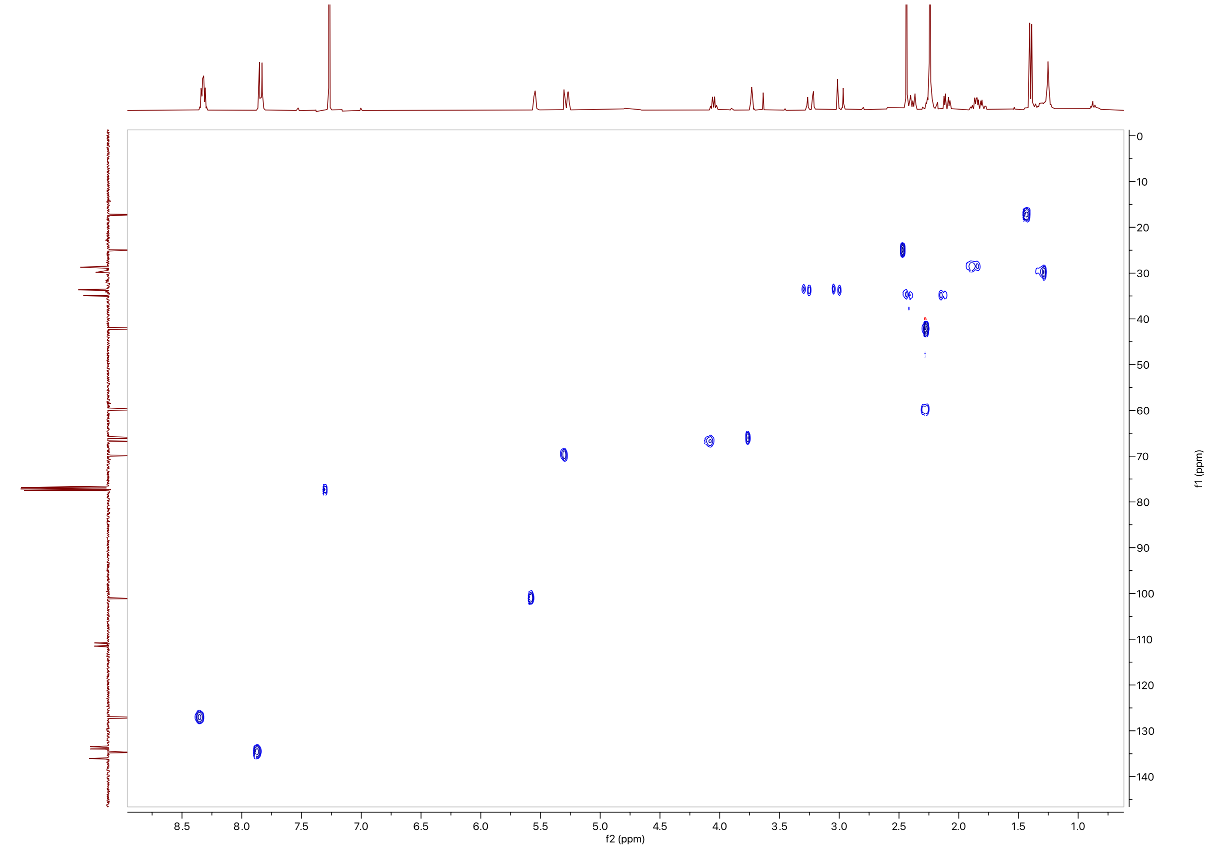
8.5
8.0
7.5
7.0
6.5
6.0
5.5
5.0
4.5
4.0
3.5
3.0
2.5
2.0
1.5
1.0
f2 (ppm)
f1 (ppm)

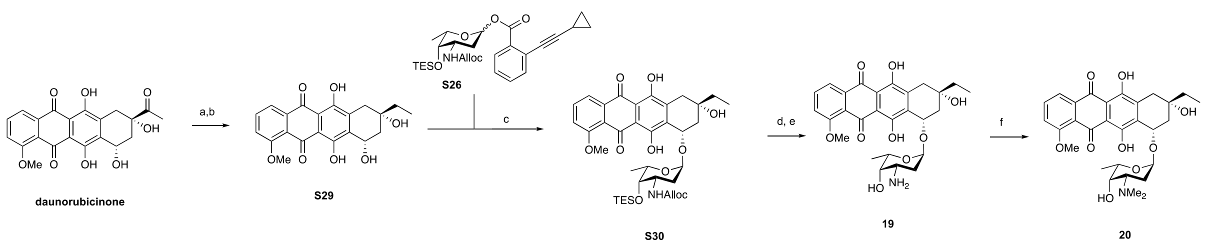

Scheme S6. Synthesis of (*N,N*-dimethyl) 13-deoxydaunorubicin (**19**) and (**20**). *Reagents and conditions:* (a) tosylhydrazide, MeOH/DCM; (b) $NaCNBH_3$, PPTSA, MeOH, 35% over 2 steps; (c) PPh_3AuNTf_2, DCM, 51%; (d) $Pd(PPh_3)_4$, NDMBA, DCM; (e) HF-pyr, pyr, 90% over 2 steps; (f) aq. CH_2O, $NaBH(OAc)_3$, EtOH, 86%.

13-deoxy-daunorubicinone (S29)

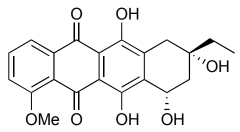

A solution of commercially available daunorubicinone (1.66 g, 4.17 mmol) and *p*-toluenesulfonylhydrazide (1.94 g, 10.4 mmol, 2.5 eq) in MeOH (85 mL) and DCM (85 mL) was stirred overnight. Then, addition of Et_2O (3.2 L) gave a red precipitate that was washed with Et_2O to give the crude hydrazone as a red solid.

The above hydrazone was dissolved in MeOH (210 mL) and THF (210 mL) at 0°C, after which $NaCNBH_3$ (3.13 g, 50.0 mmol, 12 eq) and pyridinium *p*-toluenesulfonate (2.10 g, 8.34 mmol, 2 eq) were added at that temperature. The mixture was then refluxed for 45 minutes, after which it was cooled down to room temperature and poured into DCM. This mixture was then washed with H_2O and brine, dried over Na_2SO_4 and concentrated *in vacuo*. Column chromatography (DCM and then 0:100 - 30:70 acetone:toluene) gave the title compound as a red solid (561 mg, 1.46 mmol, 35% over two steps). ^1H NMR (400 MHz, $CDCl_3$) δ 13.72 (s, 1H), 13.16 (s, 1H), 7.86 (dd, *J* = 7.7, 1.1 Hz, 1H), 7.70 (dd, *J* = 8.4, 7.7 Hz, 1H), 7.30 (dd, *J* = 8.5, 1.1 Hz, 1H), 5.18 (s, 1H), 4.03 (s, 3H), 3.94 (d, *J* = 4.4 Hz, 1H), 3.80 (s, 1H), 3.14 – 3.05 (m, 1H), 2.54 – 2.40 (m, 1H), 2.33 (dt, *J* = 14.6, 2.1 Hz, 1H), 1.86 – 1.62 (m, 4H), 1.08 (t, *J* = 7.5 Hz, 3H). ^{13}C NMR (101 MHz, $CDCl_3$) δ 186.7, 186.4, 161.0, 156.4, 156.2, 137.1, 135.7, 135.6, 135.5, 120.7, 119.7, 118.3, 111.1, 110.8, 77.5, 77.2, 76.8, 70.1, 62.7, 56.7, 37.5, 36.1, 35.6, 7.7. HRMS: $[M + Na]^+$ calculated for $C_{21}H_{20}O_7$ 407.1107; found 407.1102.

7-[3-*N*-allyloxycarbonyl-2,3-dideoxy-4-*O*-triethylsilyl-α-L-fucopyranoside]-13-deoxy-daunorubicinone (S30)

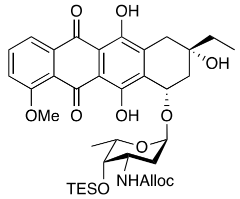

According to general procedure C, glycosyl donor **S26** (257 mg, 0.500 mmol) was glycosylated to acceptor **S30** (288 mg, 0.75 mmol, 1.5 eq). Column chromatography (20:80 EtOAc:pentane and then 1:99 – 20:80 EtOAc:toluene) afforded the title compound as a red solid (180 mg, 0.25 mmol, 51%). ^1H NMR (400 MHz, $CDCl_3$) δ 13.90 (s, 1H), 13.30 (s, 1H), 7.98 (dd, *J* = 7.7, 1.0 Hz, 1H), 7.74 (t, *J* = 8.1 Hz, 1H), 7.40 – 7.30 (m, 1H), 5.93 – 5.76 (m, 1H), 5.47 (d, *J* = 3.8 Hz, 1H), 5.31 – 5.12 (m, 3H), 4.62 (d, *J* = 8.7 Hz, 1H), 4.49 (qdt, *J* = 13.4, 5.7, 1.5 Hz, 2H), 4.15 (q, *J* = 6.4 Hz, 1H), 4.06 (s, 3H), 4.01 (s, 1H), 3.86 (tt, *J* = 7.9, 4.7 Hz, 1H), 3.78 (s, 1H), 3.28 – 3.14 (m, 1H), 2.57 – 2.46 (m, 1H), 2.36 (dt, *J* = 14.8, 2.4 Hz, 1H), 1.98 – 1.56 (m, 6H), 1.25 (d, *J* = 6.6 Hz, 3H), 1.07 (t, *J* = 7.4 Hz, 3H), 0.99 (t, *J* = 7.9 Hz, 9H), 0.66 (qd, *J* = 7.9, 2.0 Hz, 6H). ^{13}C NMR (101 MHz, $CDCl_3$) δ 187.0, 186.6, 161.0, 157.0, 156.3, 155.1, 136.4, 135.7, 135.6, 135.4, 132.9, 121.1, 119.8, 118.3, 117.7, 111.2, 111.0, 101.1, 71.1, 71.1, 69.3, 67.5, 65.5, 56.7, 47.4, 37.3, 36.3, 35.4, 30.3, 17.6, 7.5, 7.1, 5.3. HRMS: $[M + Na]^+$ calculated for $C_{37}H_{49}NO_{11}Si$ 734.2973; found 734.2966.

13-deoxy-daunorubicin (19)

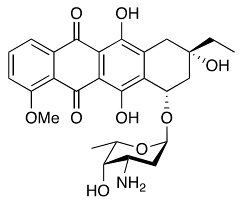

According to general procedure D, **S30** (180 mg, 0.25 mmol) was deprotected. Column chromatography (30:70 acetone:DCM) gave the crude amine.

The silyl ether was then removed according to general procedure E. Column chromatography on neutral silica gel (0:100 – 20:80 MeOH:DCM) gave the title compound as a red solid (115 mg, 0.22 mmol, 90% over two steps). ^{1}H NMR (400 MHz, $CDCl_3$ + pyridine-*d*5) δ 7.98 (d, *J* = 7.7 Hz, 1H), 7.75 (t, *J* = 8.0 Hz, 1H), 7.35 (d, *J* = 8.5 Hz, 1H), 5.47 (d, *J* = 3.8 Hz, 1H), 5.19 (s, 1H), 4.16 (q, *J* = 6.6 Hz, 1H), 4.05 (s, 3H), 3.48 (d, *J* = 2.5 Hz, 1H), 3.27 – 3.07 (m, 2H), 2.60 – 2.46 (m, 1H), 2.35 (d, *J* = 14.7 Hz, 1H), 1.89 – 1.59 (m, 5H), 1.35 (d, *J* = 6.6 Hz, 3H), 1.07 (t, *J* = 7.4 Hz, 3H). ^{13}C NMR (101 MHz, $CDCl_3$ + pyridine-*d*5) δ 186.7, 161.1, 156.9, 156.3, 136.3, 135.7, 135.5, 123.9, 121.1, 119.8, 118.4, 111.2, 101.1, 70.8, 69.6, 67.0, 56.8, 46.4, 37.1, 36.4, 35.4, 32.3, 17.0, 7.6. HRMS: $[M + H]^+$ calculated for $C_{27}H_{31}NO_{19}$ 514.2077; found 514.2069.

***N,N*-dimethyl-13-deoxy-daunorubicin (20)**

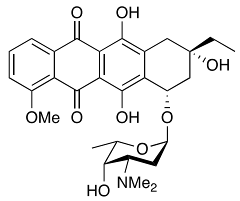

To a solution of **19** (77 mg, 0.15 mmol) in EtOH (15 mL) was added 37% aq. CH_2O (0.4 mL) and the mixture was stirred for 10 minutes before addition of $NaBH(OAc)_3$ (159 mg, 0.75 mmol, 5 eq). After stirring for 70 minutes, the mixture was poured into sat. aq. $NaHCO_3$ and extracted with $CHCl_3$. The organic layer was dried over Na_2SO_4 and concentrated *in vacuo*. Column chromatography on neutral silica gel (5:95 – 20:80 MeOH:DCM) gave the title compound as a red solid (70 mg, 0.13 mmol, 86%). ^{1}H NMR (400 MHz, $CDCl_3$) δ 13.92 (s, 1H), 13.27 (s, 1H), 7.97 (dd, *J* = 7.8, 1.1 Hz, 1H), 7.75 (dd, *J* = 8.4, 7.7 Hz, 1H), 7.36 (dd, *J* = 8.5, 1.1 Hz, 1H), 5.66 – 5.37 (m, 1H), 5.17 (dd, *J* = 4.3, 2.1 Hz, 1H), 4.24 (d, *J* = 12.2 Hz, 1H), 4.15 – 4.08 (m, 1H), 4.07 (s, 3H), 3.73 (s, 1H), 3.23 – 3.15 (m, 1H), 2.55 – 2.45 (m, 1H), 2.37 (dt, *J* = 14.7, 2.2 Hz, 1H), 2.29 (ddd, *J* = 11.4, 5.9, 2.6 Hz, 1H), 2.24 (s, 7H), 1.85 – 1.64 (m, 5H), 1.39 (d, *J* = 6.6 Hz, 3H), 1.09 (t, *J* = 7.4 Hz, 3H). ^{13}C NMR (101 MHz, $CDCl_3$) δ 186.9, 186.6, 161.0, 156.9, 156.2, 136.2, 135.6, 135.6, 135.4, 121.0, 119.8, 118.3, 111.1, 111.0, 101.0, 71.0, 69.6, 66.5, 66.0, 59.9, 56.7, 42.0, 37.1, 36.2, 35.4, 28.5, 17.1, 7.5. HRMS: $[M + H]^+$ calculated for $C_{29}H_{35}NO_9$ 542.2390; found 542.2381.

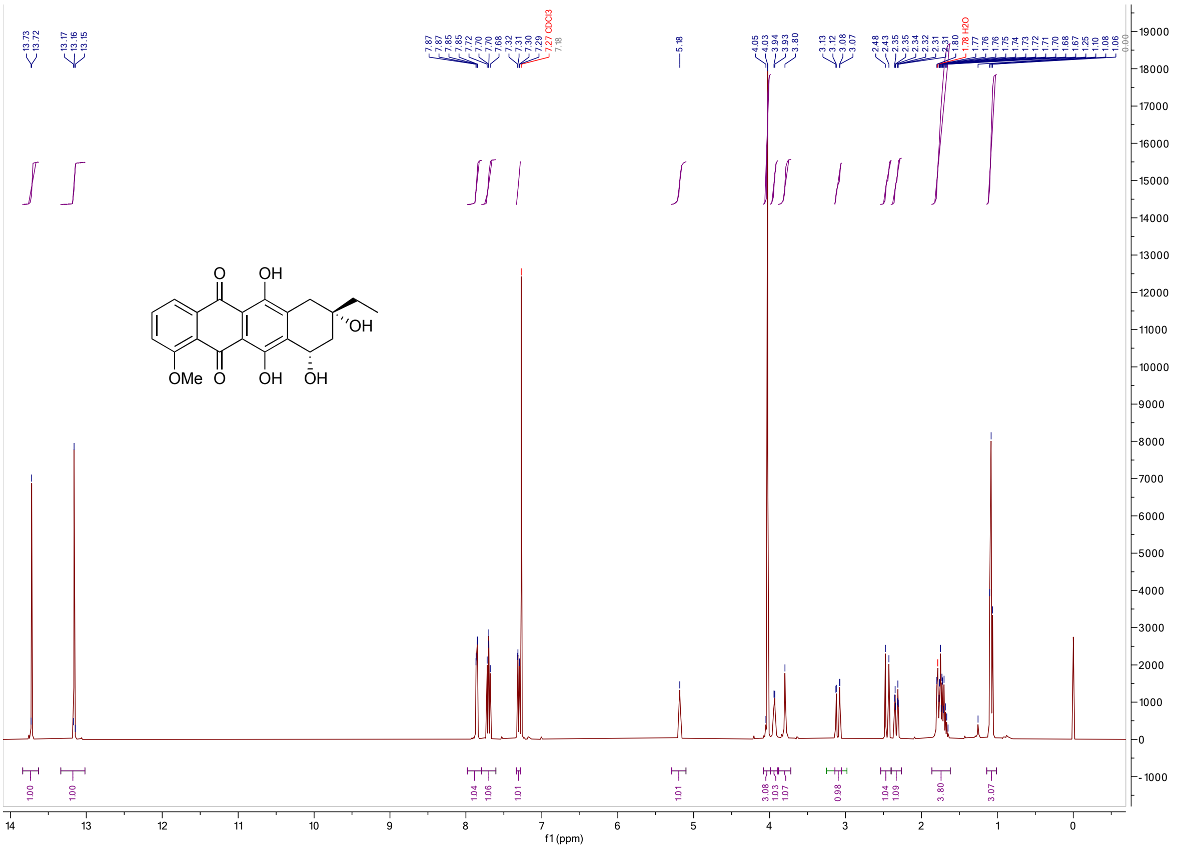

13.73
13.72
13.17
13.16
13.15
7.27 CDCl3
5.18
1.78 H2O
O
OH
OH
OMe
O
OH
OH
1.00
1.00
1.04
1.06
1.01
1.01
3.08
1.03
1.07
0.98
1.04
1.09
3.80
3.07
f1 (ppm)

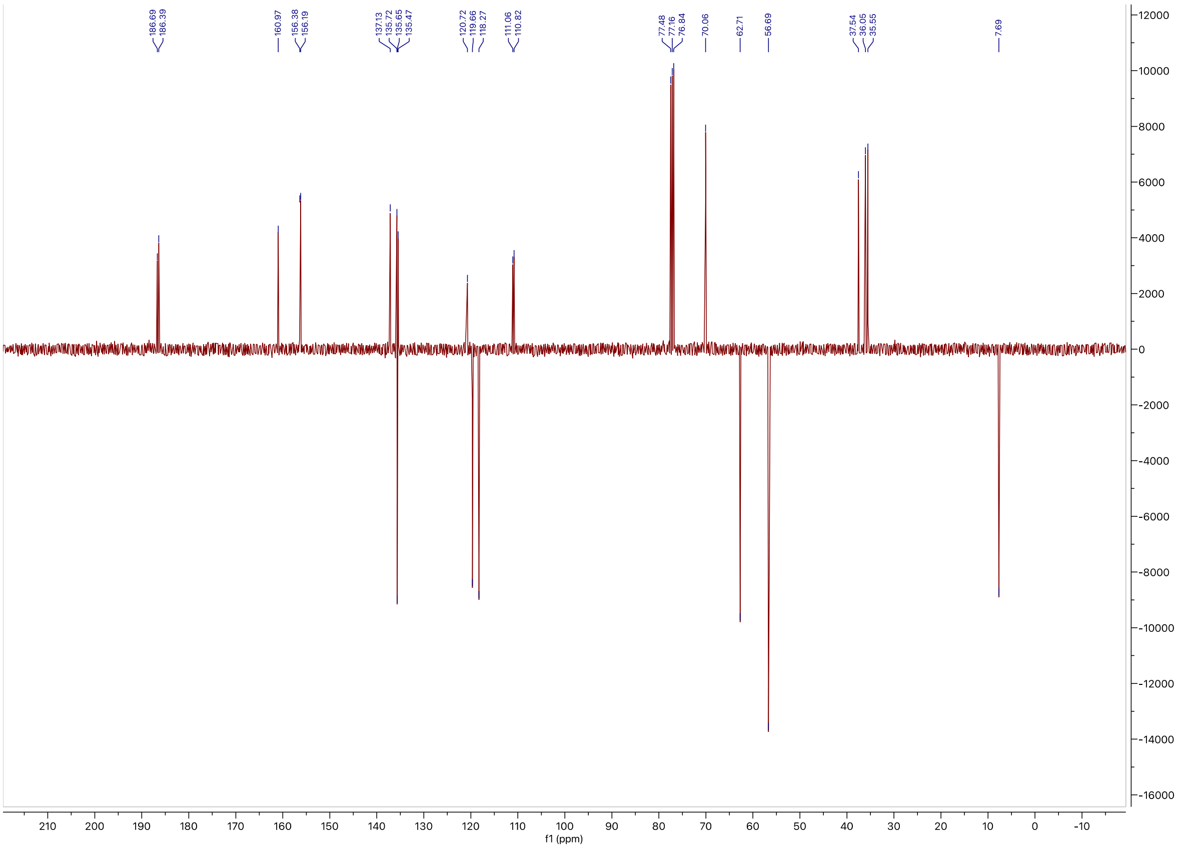

186.69
186.39
160.97
156.38
156.19
137.13
135.72
135.65
135.47
120.72
119.66
118.27
111.06
110.82
77.48
77.16
76.84
70.06
62.71
56.69
37.54
36.05
35.55
7.69
f1 (ppm)

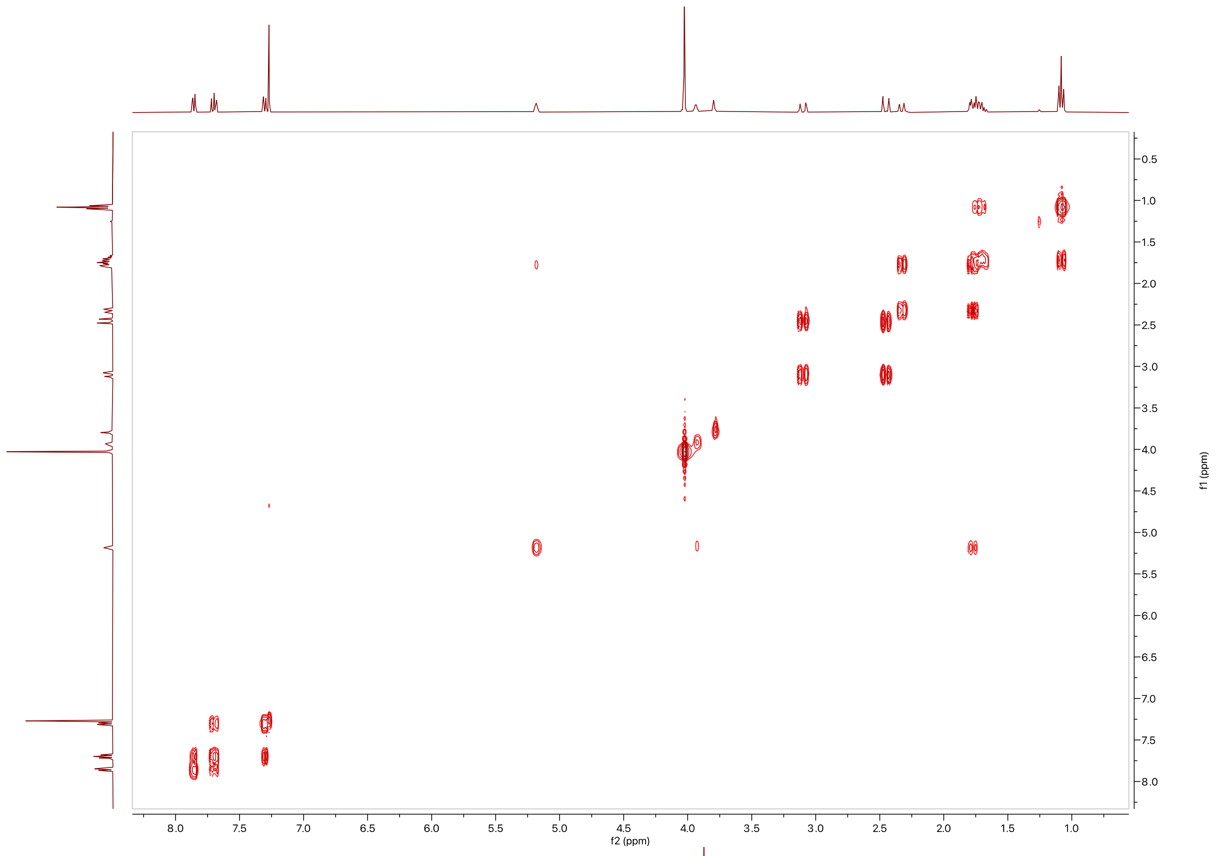
f2 (ppm)
f1 (ppm)

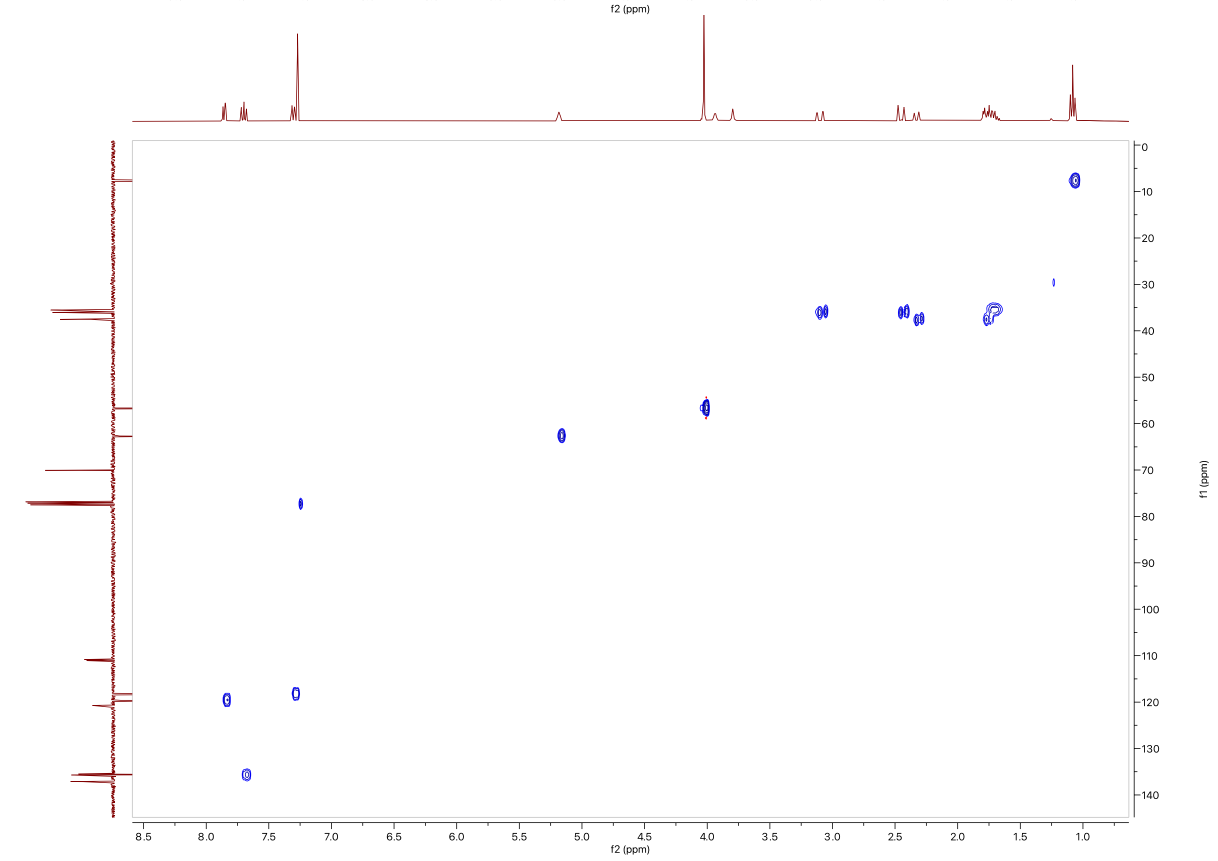
f2 (ppm)
f1 (ppm)

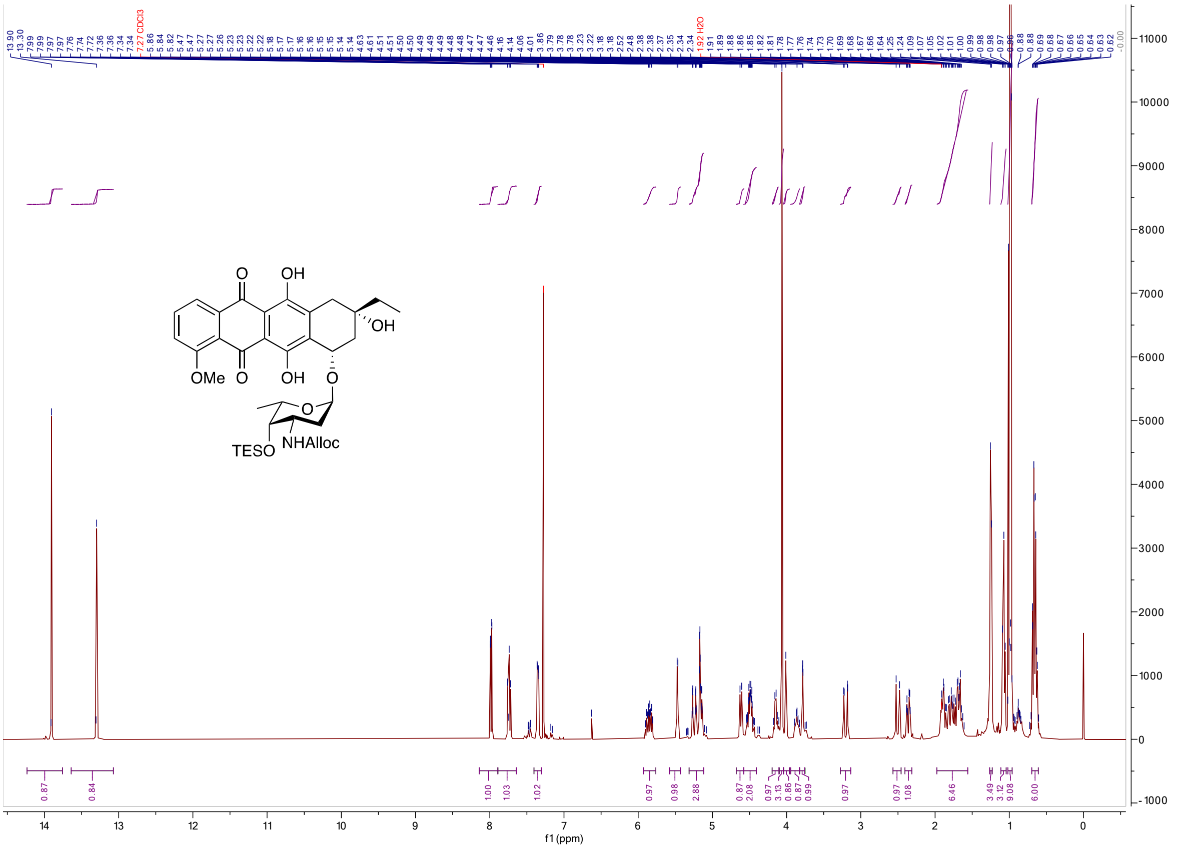
O
OH
OH
OMe
O
OH
O
O
TESO
NHAlloc
f1 (ppm)

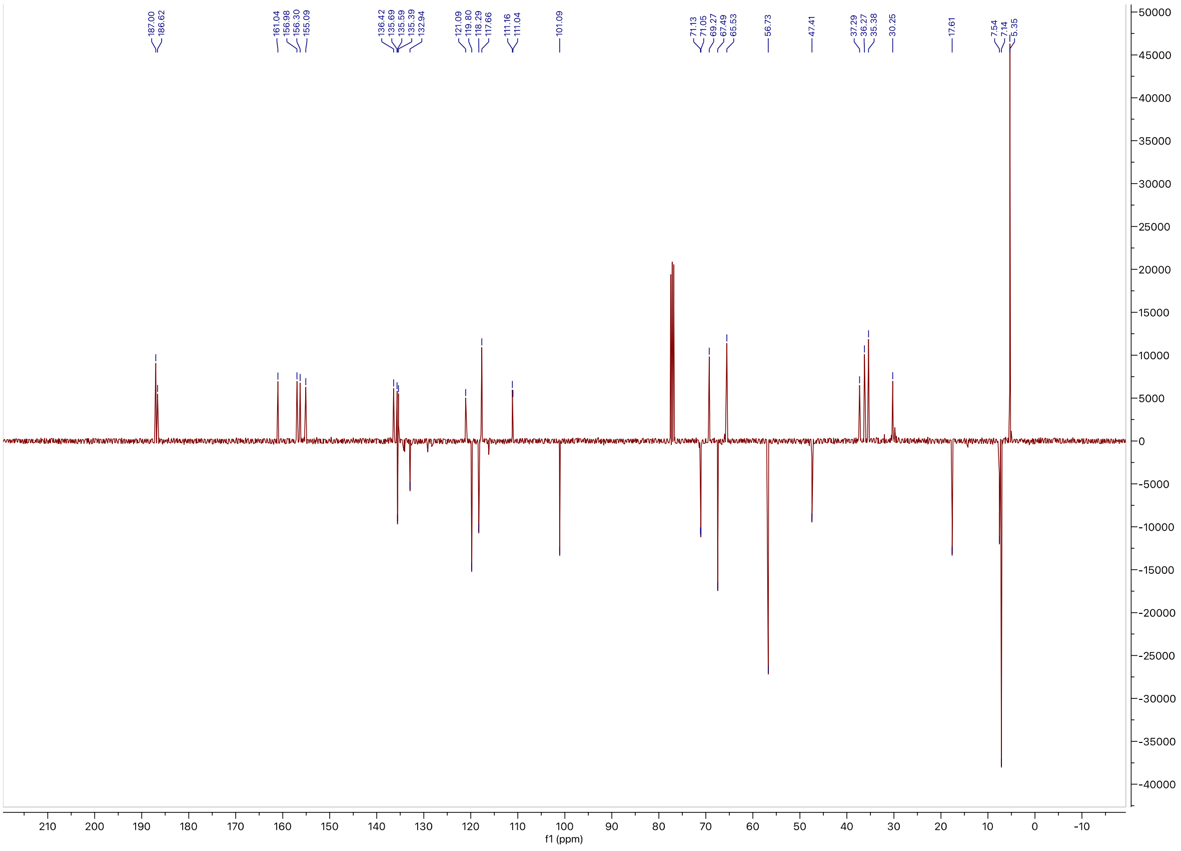
f1 (ppm)

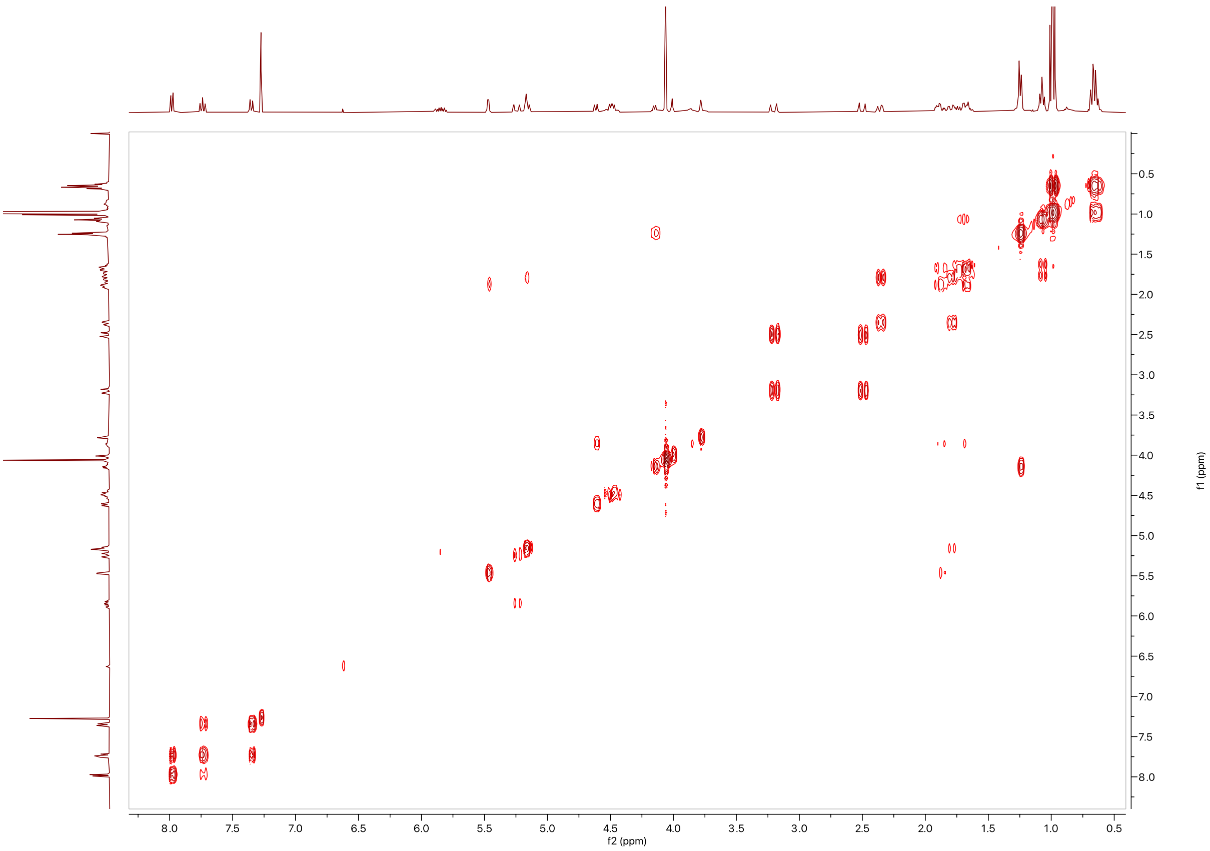
f2 (ppm)
f1 (ppm)

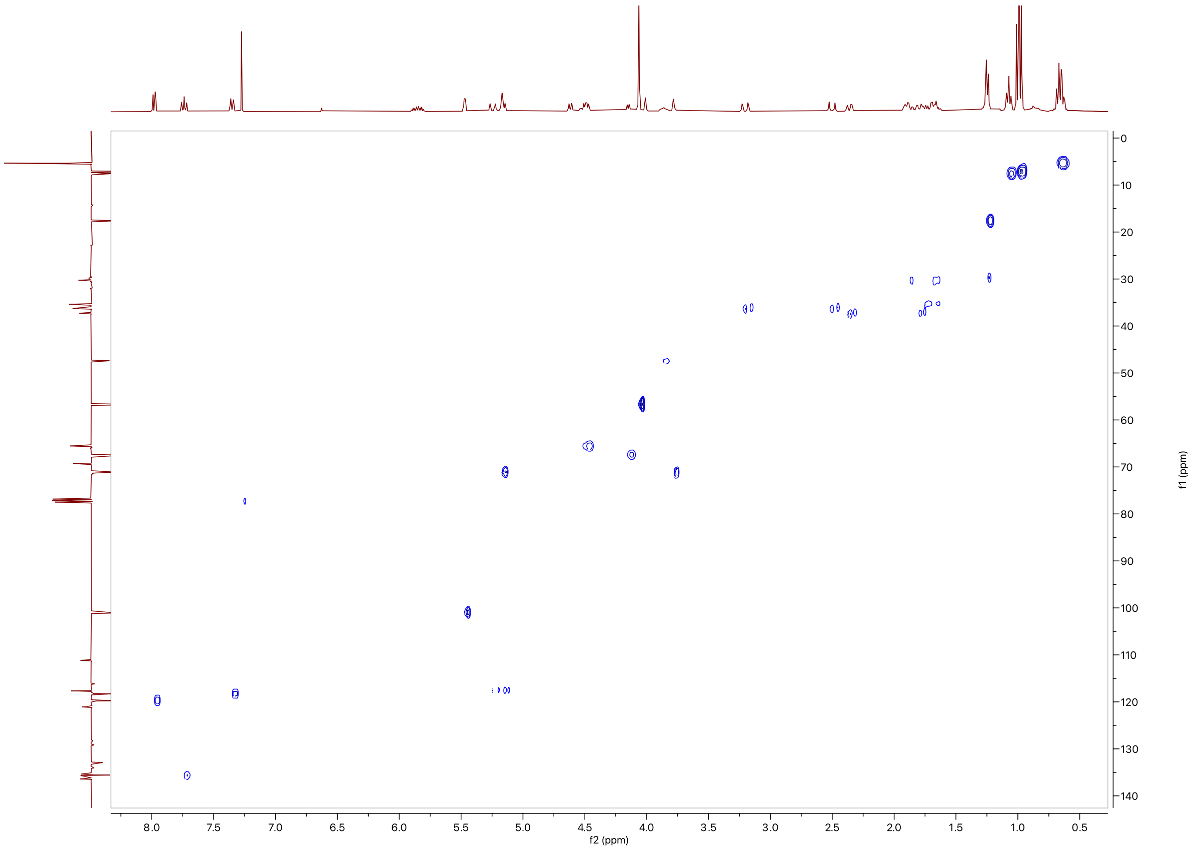
f2 (ppm)
f1 (ppm)

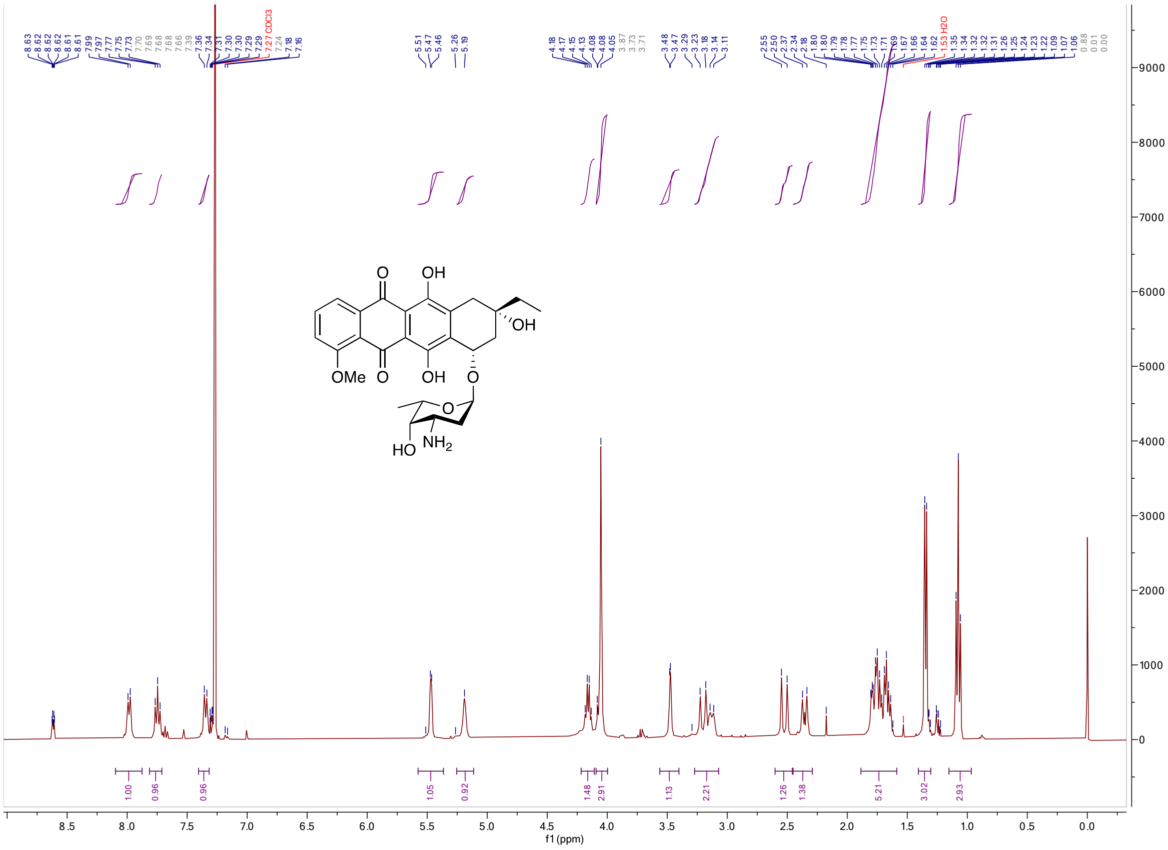
OMe
OH
NH2
HO
f1 (ppm)

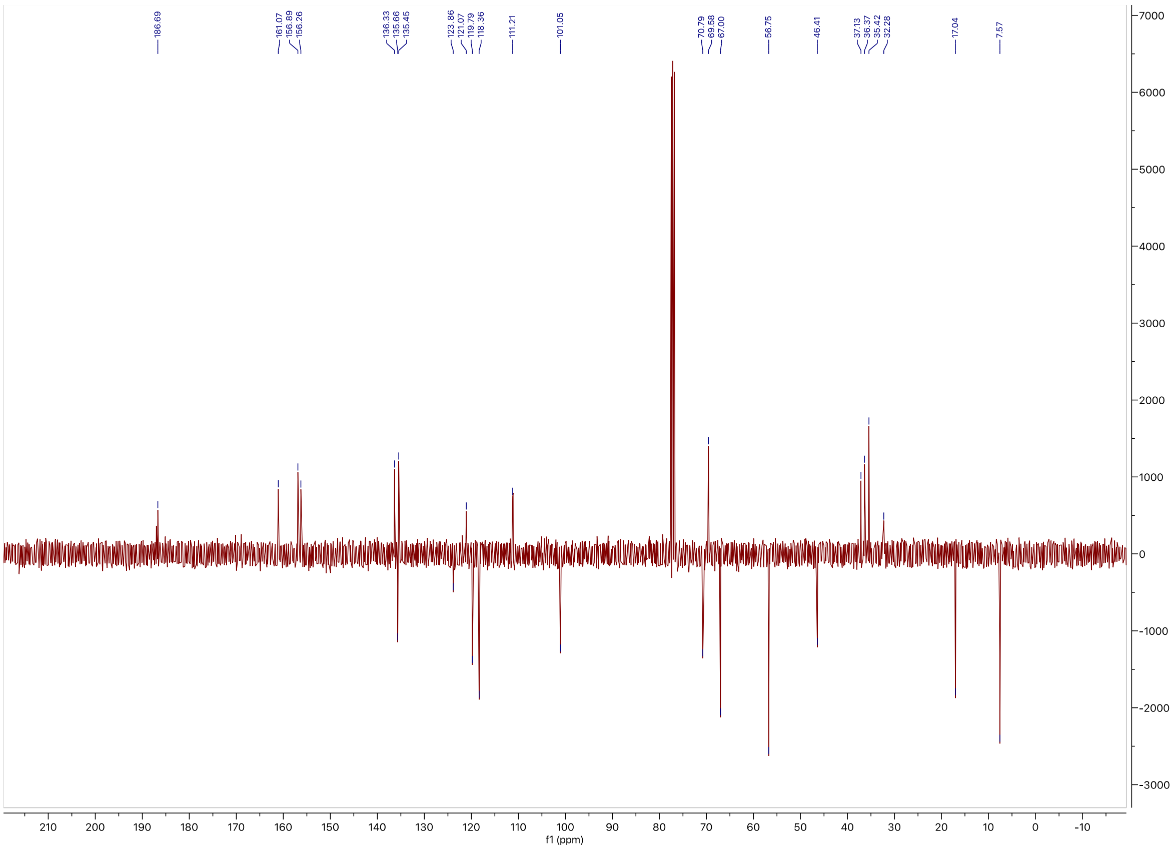
f1 (ppm)

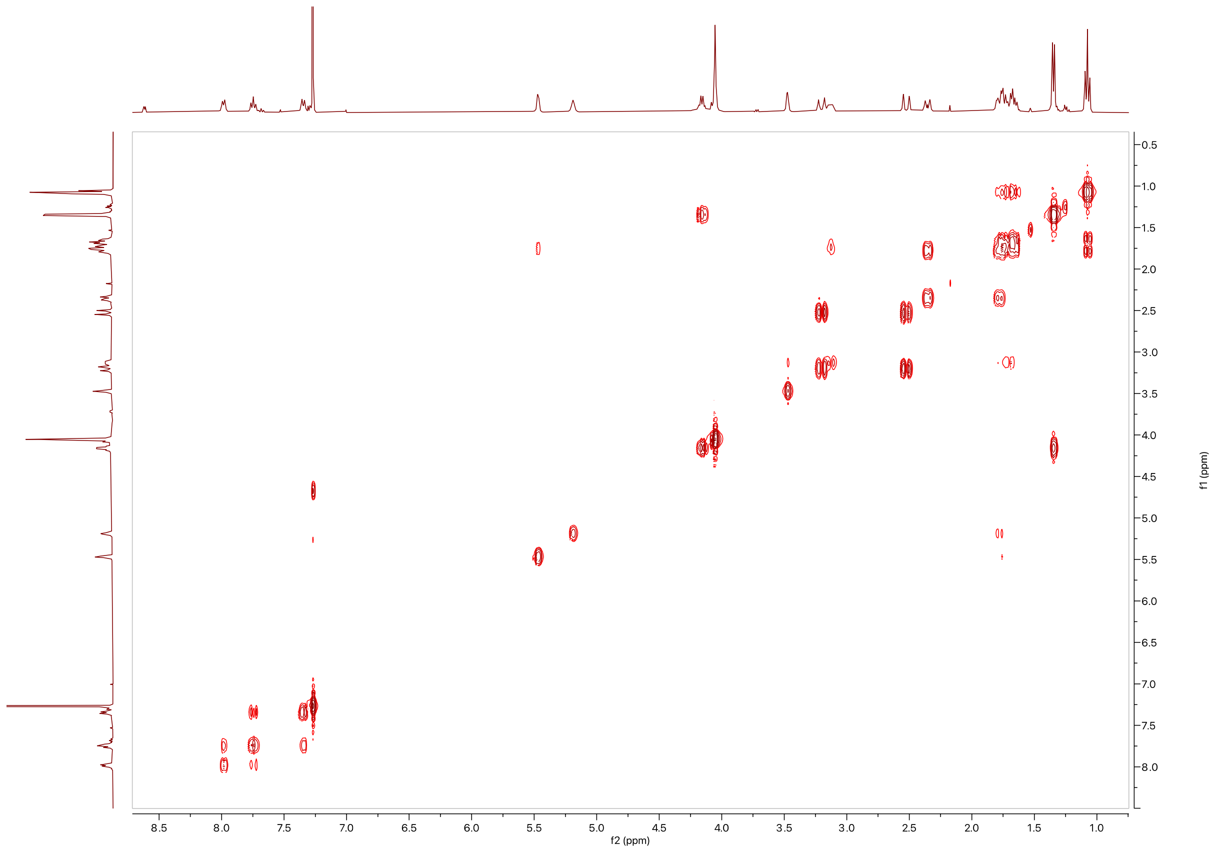
f2 (ppm)
f1 (ppm)

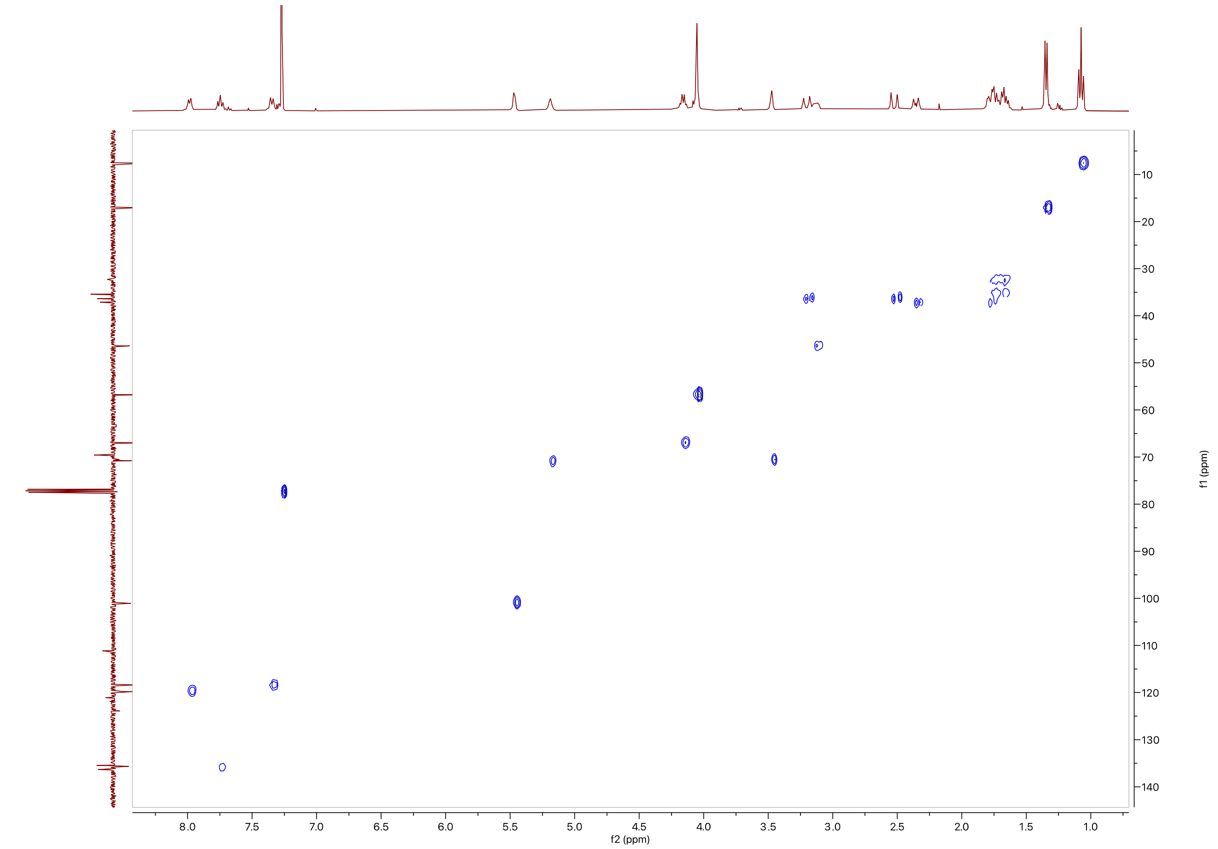
f2 (ppm)
f1 (ppm)

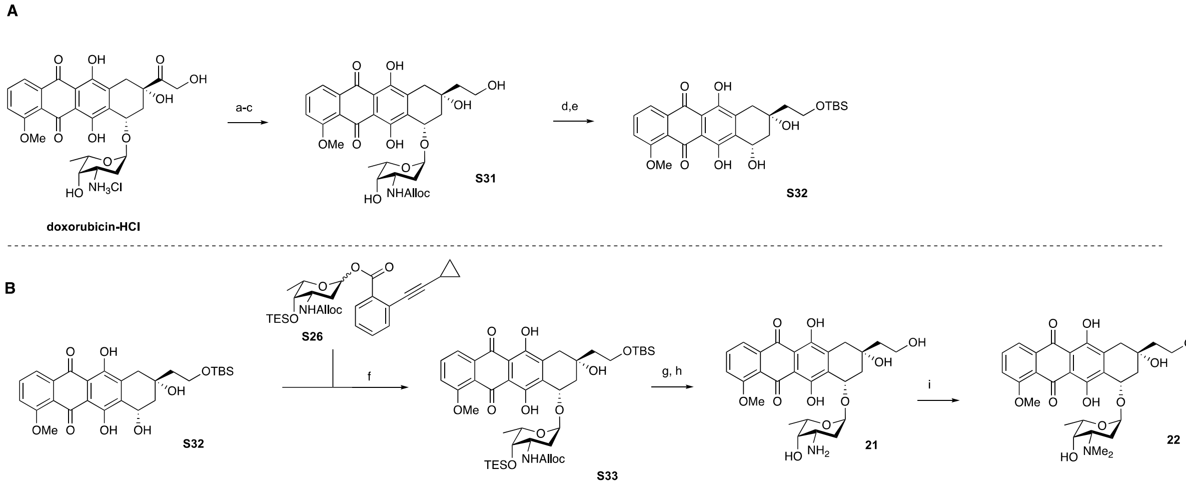

Scheme S7. Synthesis of (*N,N*-dimethyl) 13-deoxydoxorubicin **(21)** and **(22)**. (A) Synthesis of 13-deoxydoxorubicinone acceptor **S32**; (B) Glycosylation, *N,N*-dimethylation and deprotections. *Reagents and conditions:* (a) Alloc-OSu, $NaHCO_3$, THF/H_2O; (b) benzenesulfonyl hydrazide, MeOH; (c) $NaCNBH_3$, PPTSA, MeOH, 45% over 3 steps; (d) HCl, MeOH; (e) TBS-Cl, imidazole, DMF (22% over 3 steps); (f) PPh_3AuNTf_2, DCM, 59%; (d) $Pd(PPh_3)_4$, NDMBA, DCM; (e) HF-pyr, pyr, 63% over 2 steps; (f) aq. CH_2O, $NaBH(OAc)_3$, EtOH, 43%.

7-[3-*N*-allyloxycarbonyl-2,3-dideoxy-α-L-fucopyranoside]-13-deoxy-doxorubicinone (S31)

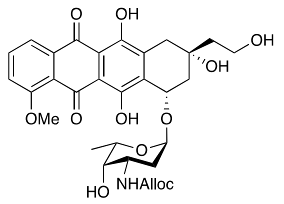

To a solution of commercially available doxorubicin hydrochloride (2.90 g, 5.00 mmol) in THF (70 mL) and H_2O (70 mL) were added $NaHCO_3$ (1.35 g, 16.1 mmol, 3.2 eq) and *N*-(allyloxycarbonyloxy)succinimide (2.03 g, 10.2 mmol, 2.1 eq) and the resultant mixture was stirred overnight. It was then poured into DCM, washed with 1M HCl and H_2O successively, dried over Na_2SO_4 and concentrated *in vacuo* to give the crude Alloc protected anthracycline. This was redissolved in MeOH (100 mL) after which benzenesulfonyl hydrazide (4.31 g, 25.0 mmol, 5 eq) was added. After stirring for 3 days, it was concentrated *in vacuo* and coevaporated with toluene thrice to give the crude hydrazone. This was then was dissolved in MeOH (250 mL) at 0ºC, after which $NaCNBH_3$ (3.77 g, 60.0 mmol, 12 eq) and pyridinium *p*-toluenesulfonate (2.52 g, 10.0 mmol, 2 eq) were added at that temperature. The mixture was then refluxed for 30 minutes, after which it was cooled down to room temperature and poured into DCM. This mixture was then washed with H_2O and brine, dried over Na_2SO_4 and concentrated *in vacuo*. Column chromatography (30:70 - 40:60 acetone:toluene) gave the title compound as a red solid (1.40 g, 2.28 mmol, 46% over three steps). ^1H NMR (400 MHz, $CDCl_3$) δ 13.96 (s, 1H), 13.31 (s, 1H), 8.02 (dd, *J* = 7.7, 1.1 Hz, 1H), 7.77 (t, *J* = 8.1 Hz, 1H), 7.37 (d, *J* = 8.5 Hz, 1H), 5.85 (td, *J* = 10.9, 5.3 Hz, 1H), 5.49 (d, *J* = 3.9 Hz, 1H), 5.34 – 5.00 (m, 3H), 4.48 (d, *J* = 5.7 Hz, 1H), 4.39 (d, *J* = 5.1 Hz, 1H), 4.25 (d, *J* = 7.1 Hz, 1H), 4.07 (s, 3H), 4.02 (d, *J* = 14.5 Hz, 3H), 3.93 (t, *J* = 6.7 Hz, 1H), 3.84 (s, 1H), 3.66 (d, *J* = 7.1 Hz, 1H), 3.48 – 3.34 (m, 2H), 2.61 – 2.47 (m, 2H), 2.20 – 2.11 (m, 1H), 2.02 (dt, *J* = 11.3, 5.8 Hz, 1H), 1.92 – 1.63 (m, 6H), 1.57 (q, *J* = 7.1 Hz, 2H), 1.34 (d, *J* = 6.5 Hz, 3H). ^{13}C NMR (101 MHz, $CDCl_3$) δ 187.2, 186.8, 161.1, 156.7, 156.1, 155.5, 135.8, 135.7, 135.3, 134.9, 134.9, 129.2, 128.4, 125.4, 121.1, 119.9, 118.5, 117.9, 111.5, 111.4, 111.4, 101.0, 77.5, 77.4, 77.2, 76.8, 71.2, 69.9, 69.8, 67.3, 66.7, 65.7, 59.1, 56.8, 43.4, 38.1, 36.9, 30.5, 22.4, 16.9, 10.4.

13-deoxy-14-*O*-*tert*-butyldimethylsilyl doxorubicinone (S32)

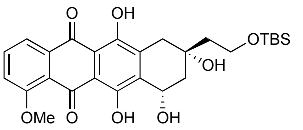

To a solution of **S31** (1.40 g, 2.28 mmol) in DCM (50 mL) and MeOH (50 mL) was added 3M HCl in MeOH (2.3 mL, 6.84 mmol, 3 eq) and the mixture was stirred for 3.5 hours after which it was concentrated *in vacuo* and thrice coevaporated with toluene. To a solution of the above compound in DMF (23 mL) were added *tert*-butyl-dimethylsilylchloride (361 mg, 2.39 mmol, 1.05 eq) and imidazole (404 mg, 5.93 mmol, 2.6 eq) and the mixture was stirred for 50 minutes. Then, equal portions of the above reagents were added and after an additional hour, the mixture was poured into DCM. This was washed with with 0.3M HCl, H_2O twice and brine, dried over Na_2SO_4 and concentrated *in vacuo*. Column chromatography (0:100 – 20:80 acetone:toluene) followed by size-exclusion chromatography (Sephadex LH-20, eluent 1:1 MeOH:DCM) gave the title compound as a red solid (260 mg, 0.51 mmol, 22% over two steps). ^{1}H NMR (400 MHz, $CDCl_3$) δ 13.95 (s, 1H), 13.35 (s, 1H), 7.97 (dd, *J* = 7.7, 1.1 Hz, 1H), 7.73 (dd, *J* = 8.4, 7.7 Hz, 1H), 7.35 (dd, *J* = 8.5, 1.1 Hz, 1H), 5.17 (ddd, *J* = 7.8, 4.7, 2.4 Hz, 1H), 4.72 (s, 1H), 4.50 (d, *J* = 8.3 Hz, 1H), 4.17 – 4.03 (m, 5H), 3.37 – 3.26 (m, 1H), 2.65 – 2.49 (m, 1H), 2.44 (dt, *J* = 14.2, 2.3 Hz, 1H), 1.98 (ddd, *J* = 14.4, 8.1, 4.5 Hz, 1H), 1.93 – 1.77 (m, 2H), 0.91 (s, 9H), 0.14 (d, *J* = 1.2 Hz, 6H). ^{13}C NMR (101 MHz, $CDCl_3$) δ 186.9, 186.7, 161.1, 156.9, 156.3, 138.3, 135.7, 135.5, 134.7, 121.2, 119.7, 118.3, 111.3, 110.9, 71.4, 62.7, 60.2, 56.8, 42.2, 39.5, 36.1, 25.9, -5.4. HRMS: $[M + Na]^+$ calculated for $C_{27}H_{34}O_8Si$ 537.1921; found 537.1917.

7-[3-*N*-allyloxycarbonyl-2,3-dideoxy-4-*O*-triethylsilyl-α-L-fucopyranoside]-13-deoxy-14-*O*-*tert*-butyldimethylsilyldoxorubicinone (S33)

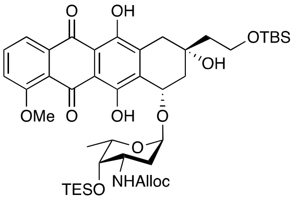

According to general procedure C, glycosyl donor **S26** (175 mg, 0.34 mmol) was glycosylated to acceptor **S32** (260 mg, 0.51 mmol, 1.5 eq). Column chromatography (0:100 – 2:98 acetone:DCM) afforded the title compound as a red solid (170 mg, 0.20 mmol, 59%). ^{1}H NMR (500 MHz, $CDCl_3$) δ 13.98 (s, 1H), 13.35 (s, 1H), 8.04 (dd, *J* = 7.7, 1.1 Hz, 1H), 7.76 (dd, *J* = 8.4, 7.7 Hz, 1H), 7.37 (dd, *J* = 8.6, 1.1 Hz, 1H), 5.86 (ddt, *J* = 16.4, 10.8, 5.6 Hz, 1H), 5.48 (d, *J* = 3.8 Hz, 1H), 5.30 – 5.07 (m, 3H), 4.65 – 4.40 (m, 3H), 4.19 (s, 1H), 4.13 (q, *J* = 6.5 Hz, 1H), 4.08 (s, 3H), 4.01 – 3.85 (m, 3H), 3.78 (s, 1H), 3.29 – 3.17 (m, 1H), 2.79 – 2.66 (m, 1H), 2.36 (d, *J* = 14.5 Hz, 1H), 1.99 (dd, *J* = 14.5, 4.8 Hz, 1H), 1.95 – 1.75 (m, 3H), 1.68 (dd, *J* = 12.7, 4.5 Hz, 1H), 1.60 (s, 3H), 1.24 (d, *J* = 6.5 Hz, 3H), 0.99 (t, *J* = 7.9 Hz, 9H), 0.93 (s, 9H), 0.65 (qd, *J* = 7.8, 3.1 Hz, 6H), 0.11 (d, *J* = 1.3 Hz, 6H). ^{13}C NMR (126 MHz, $CDCl_3$) δ 187.2, 186.8, 161.1, 157.3, 156.3, 155.1, 136.5, 135.9, 135.6, 135.6, 133.0, 121.3, 119.9, 118.7, 117.7, 111.3, 111.1, 100.9, 71.2, 71.1, 69.3, 67.5, 65.6, 59.3, 56.8, 47.5, 44.2, 39.2, 36.6, 30.3, 26.1, 17.7, 7.2, 5.4. HRMS: $[M + Na]^+$ calculated for $C_{43}H_{63}NO_{12}Si_2$ 864.3787; found 864.3777.

13-deoxy-doxorubicin (21)

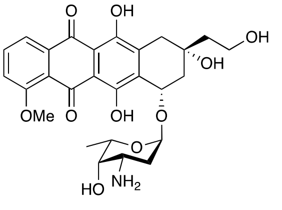

According to general procedure D, **S33** (156 mg, 0.185 mmol) was deprotected to give the crude amine. The silyl ether was then removed according to general procedure E. Column chromatography on neutral silica gel (0:100 – 20:80 MeOH:DCM) gave the title compound as a red solid (62 mg, 0.12 mmol, 63% over two steps). ^{1}H NMR (400 MHz, MeOD) δ 7.89 (dd, *J* = 7.8, 1.1 Hz, 1H), 7.80 (t, *J* = 8.1 Hz, 1H), 7.61 – 7.47 (m, 1H), 5.44 (d, *J* = 3.6 Hz, 1H), 4.97 (dd, *J* = 5.3, 2.9 Hz, 1H), 4.25 (q, *J* = 6.5 Hz, 1H), 4.01 (s, 3H), 3.96 – 3.85 (m, 2H), 3.67 (d, *J* = 2.8 Hz, 1H), 3.56 (ddd, *J* = 12.7, 4.8, 2.9 Hz, 1H), 3.20 (s, 1H), 3.11 – 3.01 (m, 1H), 2.67 – 2.57 (m, 1H), 2.36 (d, *J* = 14.5 Hz, 1H), 2.12 – 1.97 (m, 2H), 1.97 – 1.81 (m, 3H), 1.31 (d, *J* = 6.6 Hz, 3H). ^{13}C NMR (101 MHz, MeOD) δ 188.2, 187.8, 162.5, 157.7, 156.4, 137.2, 137.0, 136.5, 136.4, 121.6,

120.5, 120.2, 112.3, 112.1, 101.2, 72.6, 70.2, 68.0, 67.9, 58.8, 57.1, 44.7, 39.9, 37.2, 29.5, 17.0. HRMS: $[M + H]^+$ calculated for $C_{27}H_{31}NO_{10}$ 530.2026; found 530.2017.

***N,N*-dimethyl-13-deoxy-doxorubicin (22)**

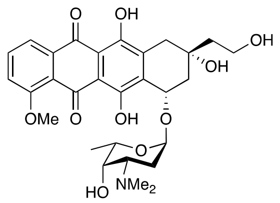

To a solution of **21** (41.0 mg, 0.08 mmol) in EtOH (7.5 mL) was added 37% aq. CH_2O (0.20 mL) and the mixture was stirred for 10 minutes before addition of $NaBH(OAc)_3$ (83 mg, 0.40 mmol, 5 eq). After stirring for 1.5 hours, the mixture was poured into sat. aq. $NaHCO_3$ and extracted with $CHCl_3$. The organic layer was dried over Na_2SO_4 and concentrated *in vacuo*. Column chromatography on neutral silica gel (10:90 – 40:60 MeOH:DCM) gave the title compound as a dark red solid (19 mg, 0.034 mmol, 45%). ^{1}H NMR (500 MHz, $CDCl_3$) δ 13.96 (s, 1H), 13.32 (s, 1H), 8.02 (dd, *J* = 7.7, 1.1 Hz, 1H), 7.78 (dd, *J* = 8.5, 7.7 Hz, 1H), 7.39 (dd, *J* = 8.6, 1.1 Hz, 1H), 5.74 – 5.39 (m, 1H), 5.25 (dd, *J* = 4.1, 2.2 Hz, 1H), 4.67 (s, 1H), 4.08 (s, 3H), 4.07 – 4.04 (m, 1H), 4.02 (t, *J* = 5.5 Hz, 2H), 3.70 (t, *J* = 2.0 Hz, 1H), 3.43 – 3.36 (m, 1H), 2.62 – 2.52 (m, 2H), 2.20 (s, 6H), 2.16 (ddd, *J* = 12.0, 5.4, 2.7 Hz, 2H), 2.08 – 2.00 (m, 1H), 1.93 – 1.74 (m, 4H), 1.42 (d, *J* = 6.6 Hz, 3H). ^{13}C NMR (126 MHz, $CDCl_3$) δ 187.2, 186.8, 161.2, 156.8, 156.1, 135.8, 135.7, 135.3, 121.1, 119.9, 118.5, 111.4, 111.3, 101.2, 71.4, 70.7, 66.7, 66.1, 59.8, 59.1, 56.8, 43.5, 42.1, 37.9, 37.0, 28.7, 17.2. HRMS: $[M + H]^+$ calculated for $C_{29}H_{35}NO_{10}$ 558.2339; found 558.2334.

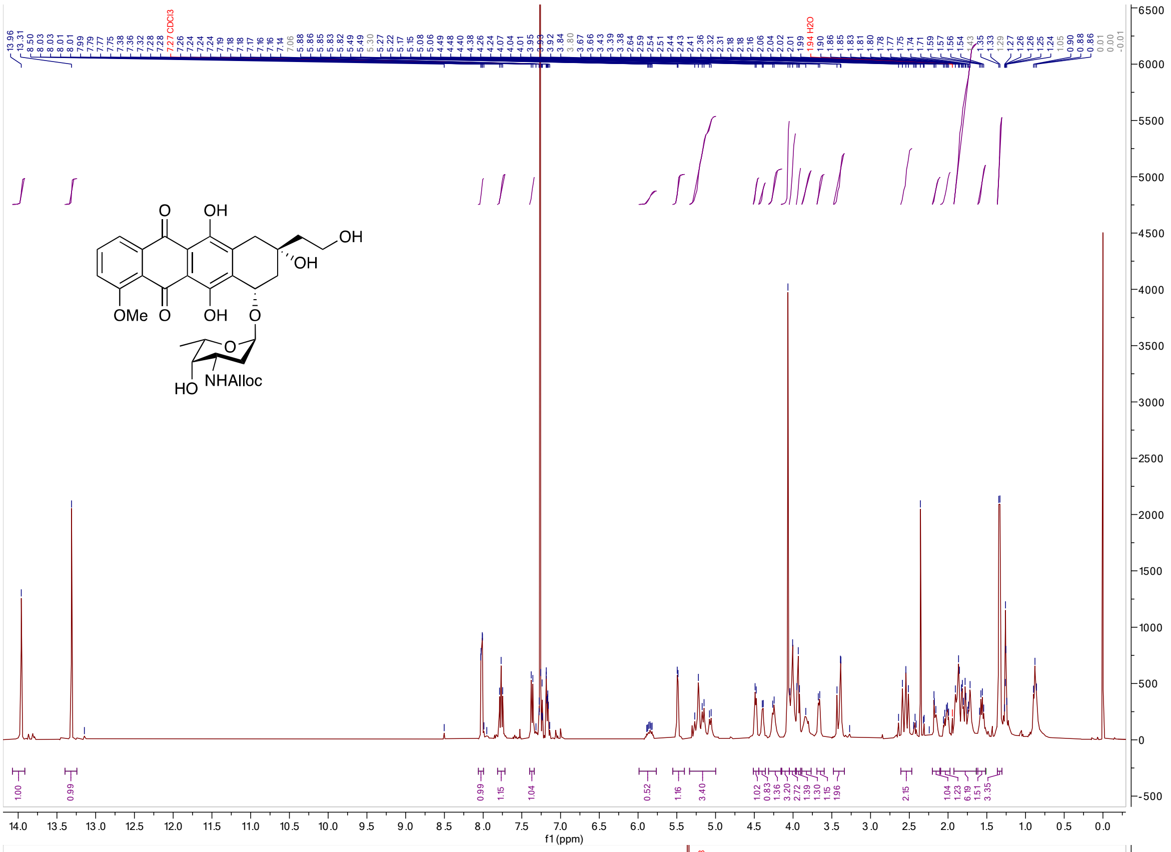

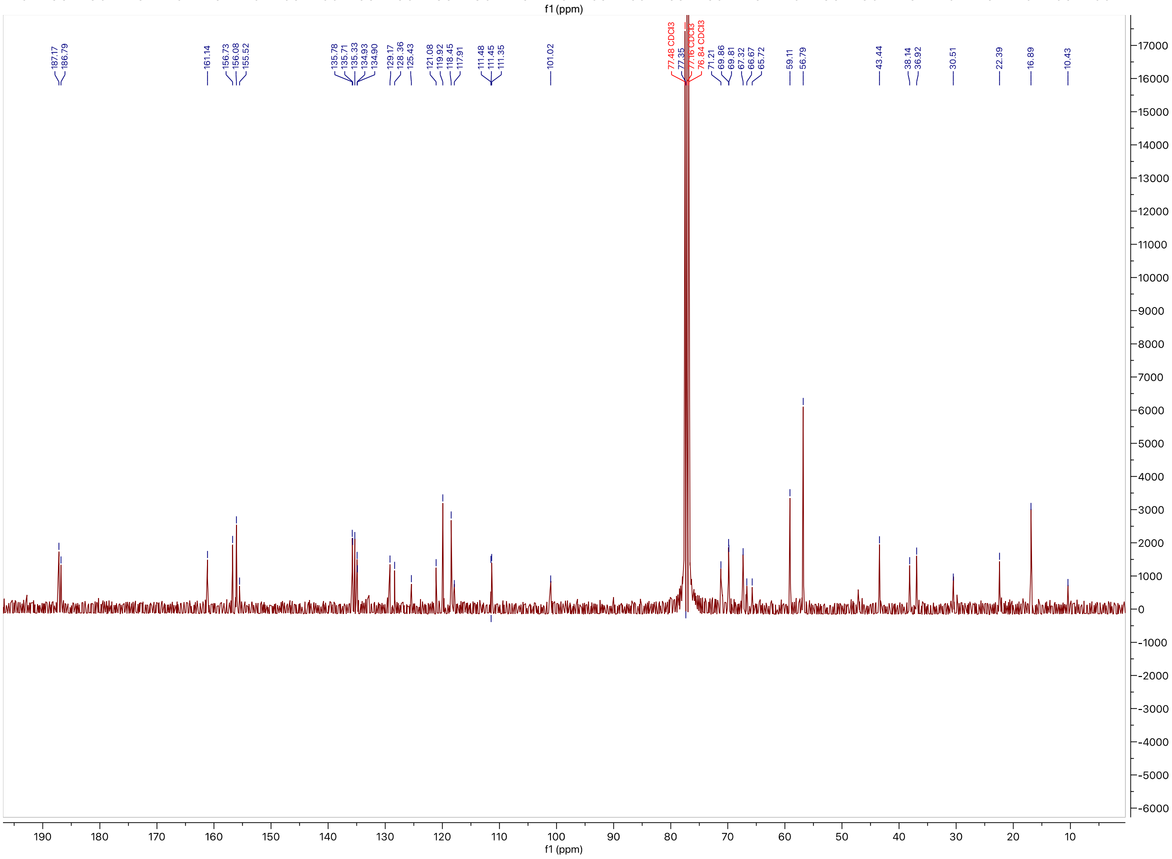

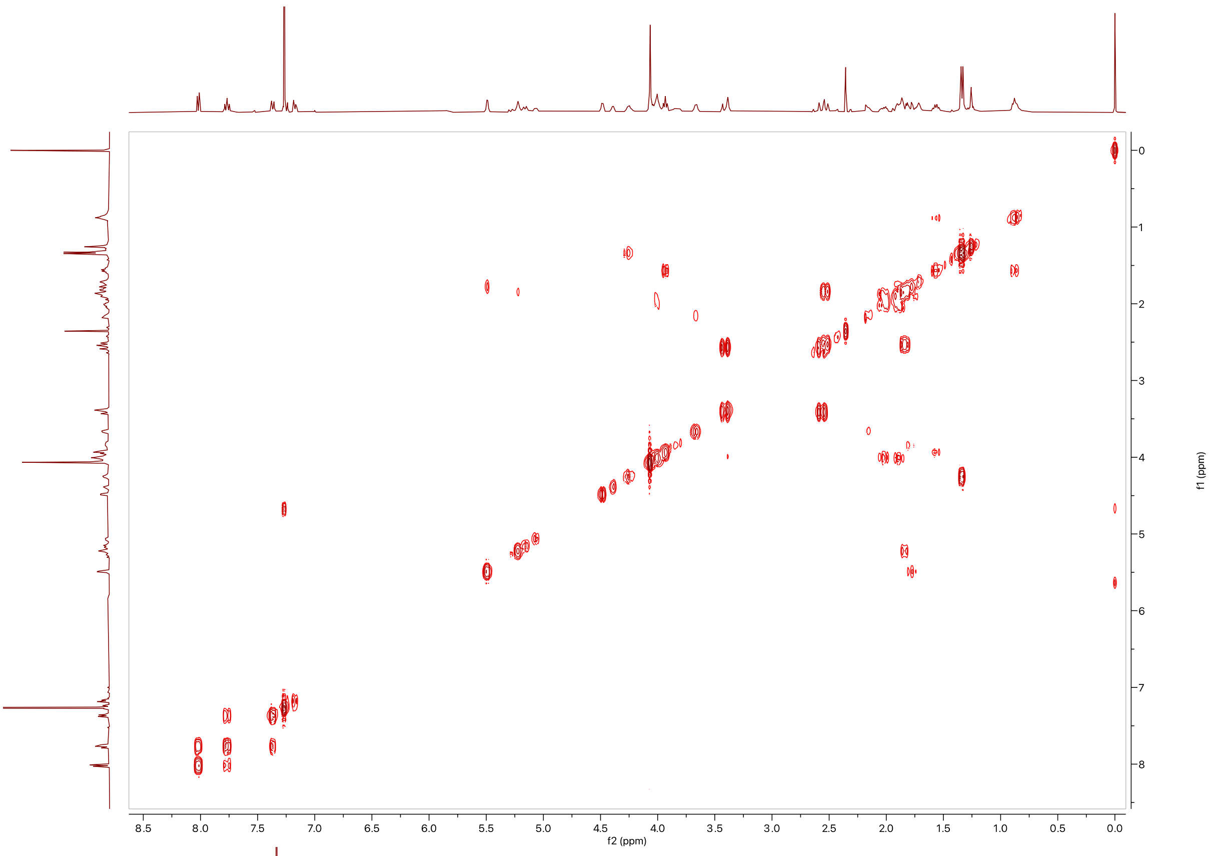
f2 (ppm)
f1 (ppm)

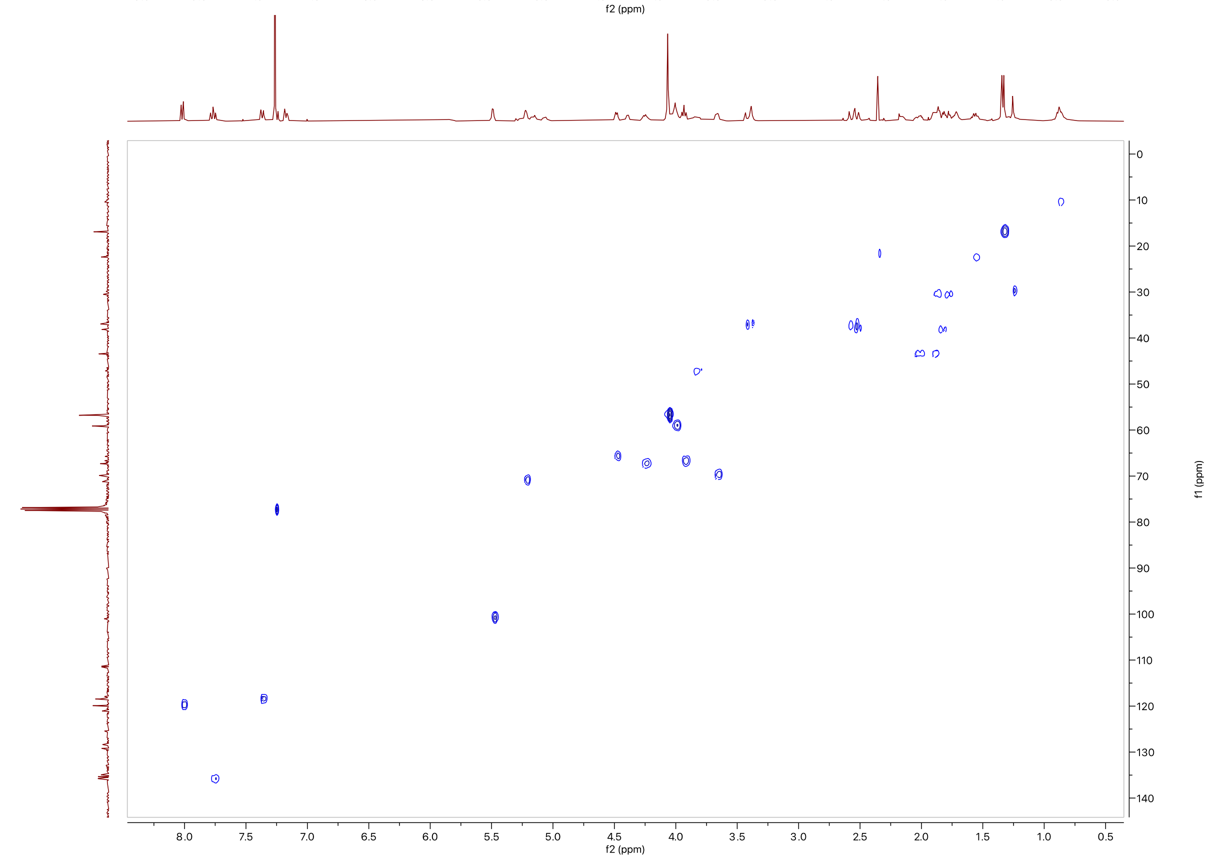
f2 (ppm)
f1 (ppm)

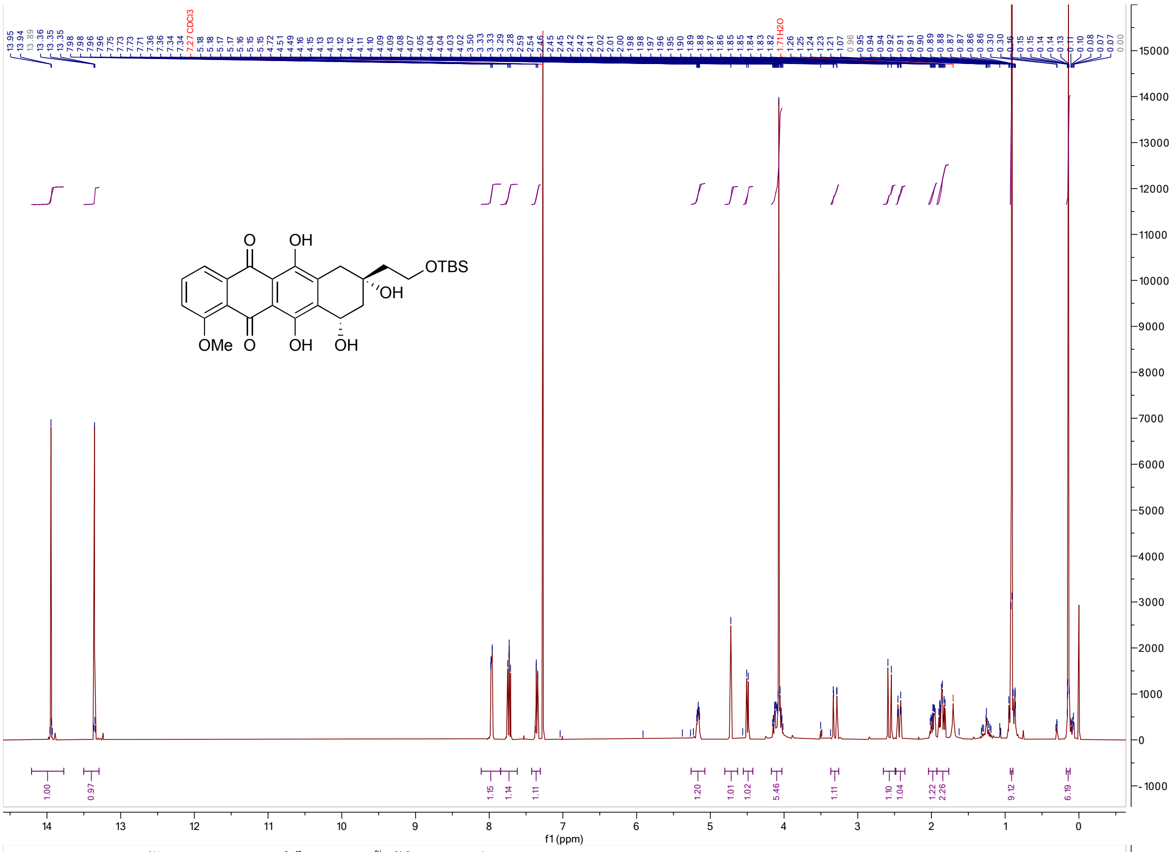
OTBS
OH
OH
OMe
O
O
OH
OH
1.00
0.97
1.15
1.14
1.11
1.20
1.01
1.02
5.46
1.11
1.10
1.04
1.22
2.26
9.12
6.19
f1 (ppm)

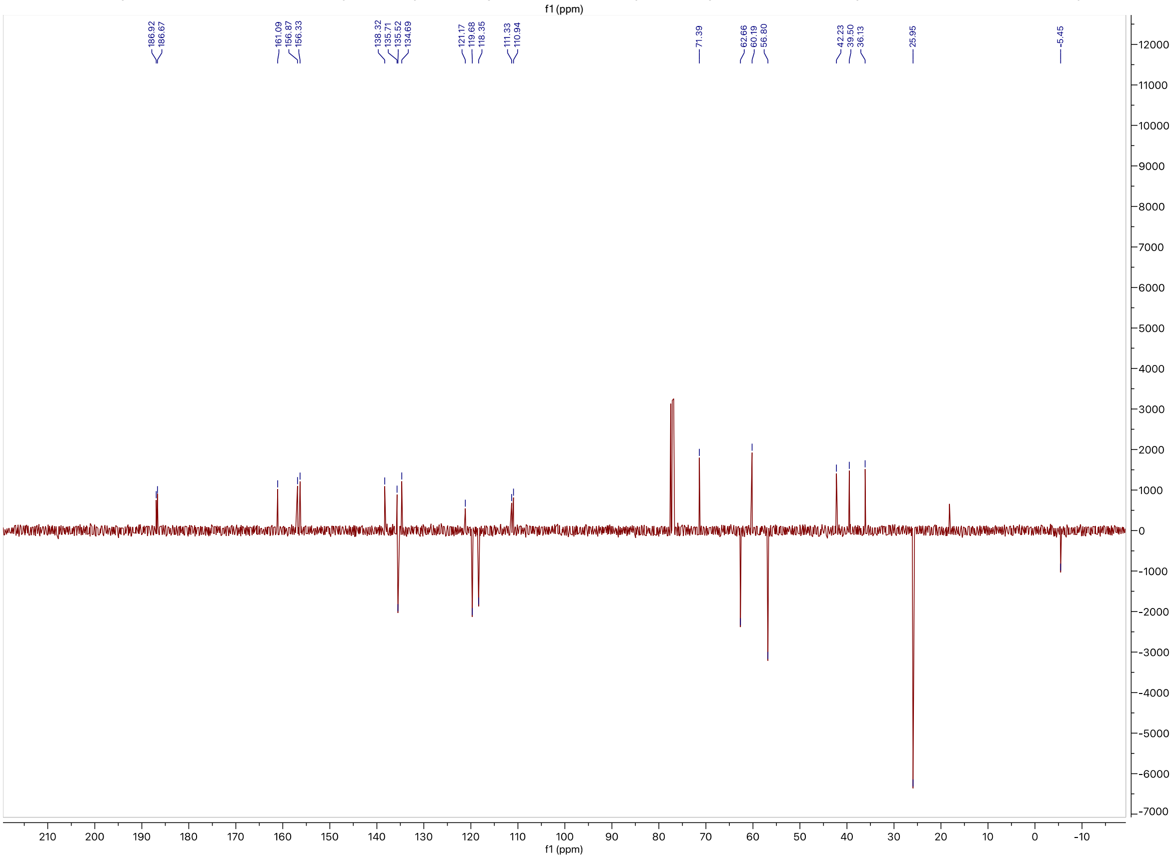
186.92
186.67
161.09
156.87
156.33
138.32
135.71
135.52
134.69
121.17
119.68
118.35
111.33
110.94
71.39
62.66
60.19
56.80
42.23
39.50
36.13
25.95
-5.45
f1 (ppm)

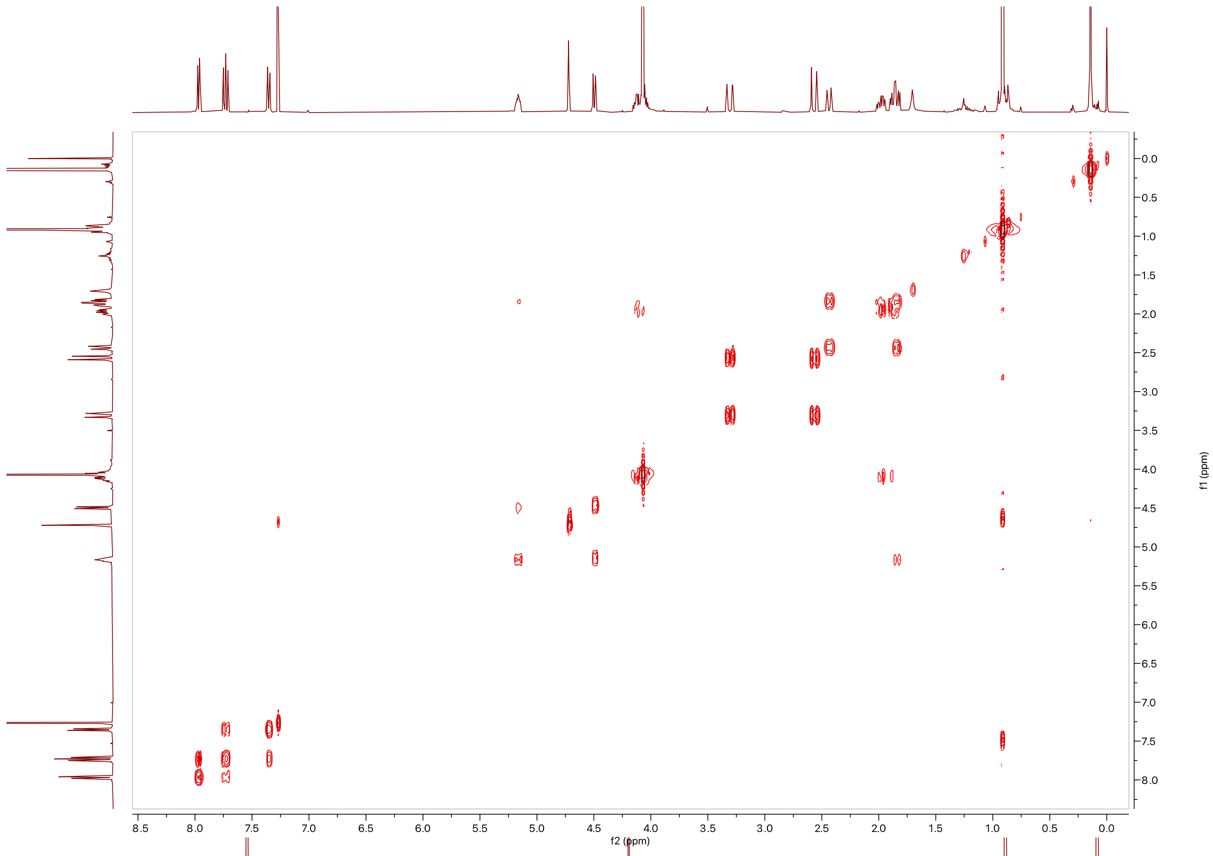

f2 (ppm)
f1 (ppm)

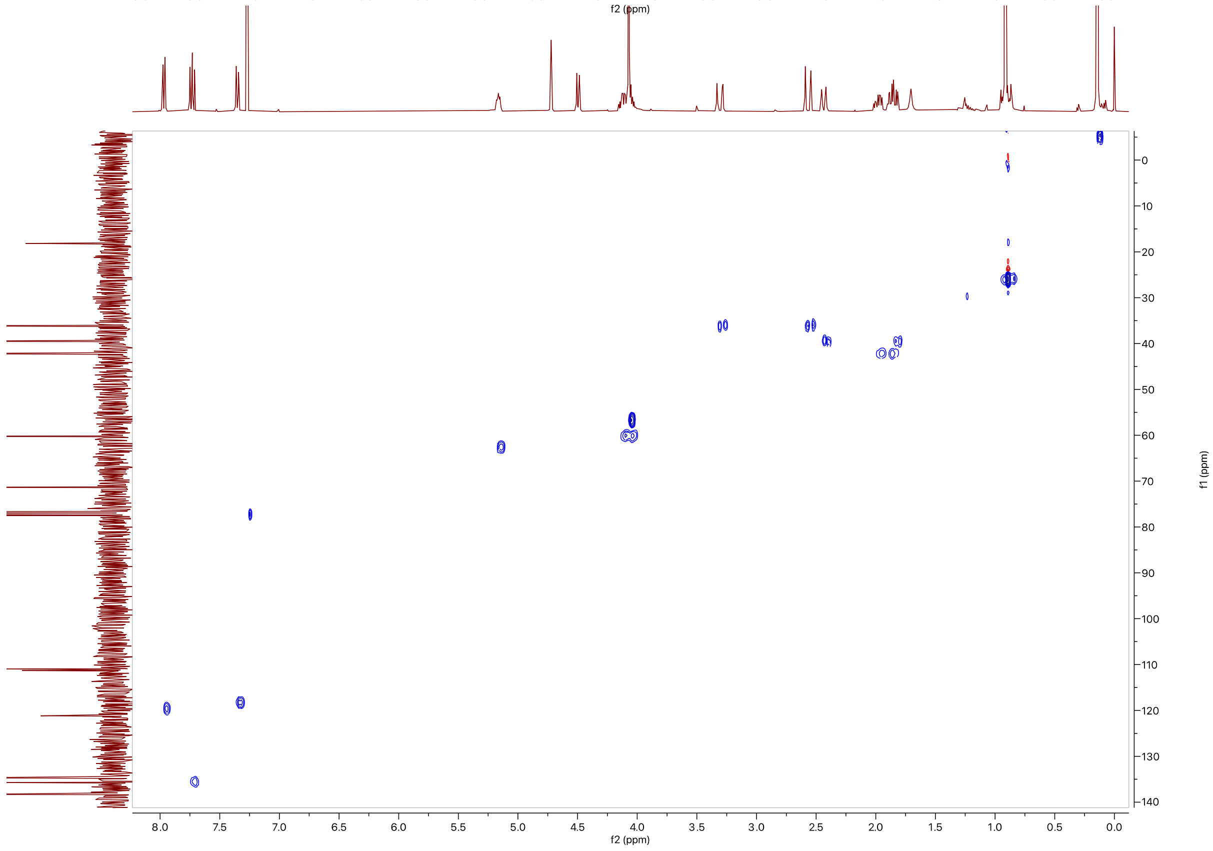

f2 (ppm)
f1 (ppm)

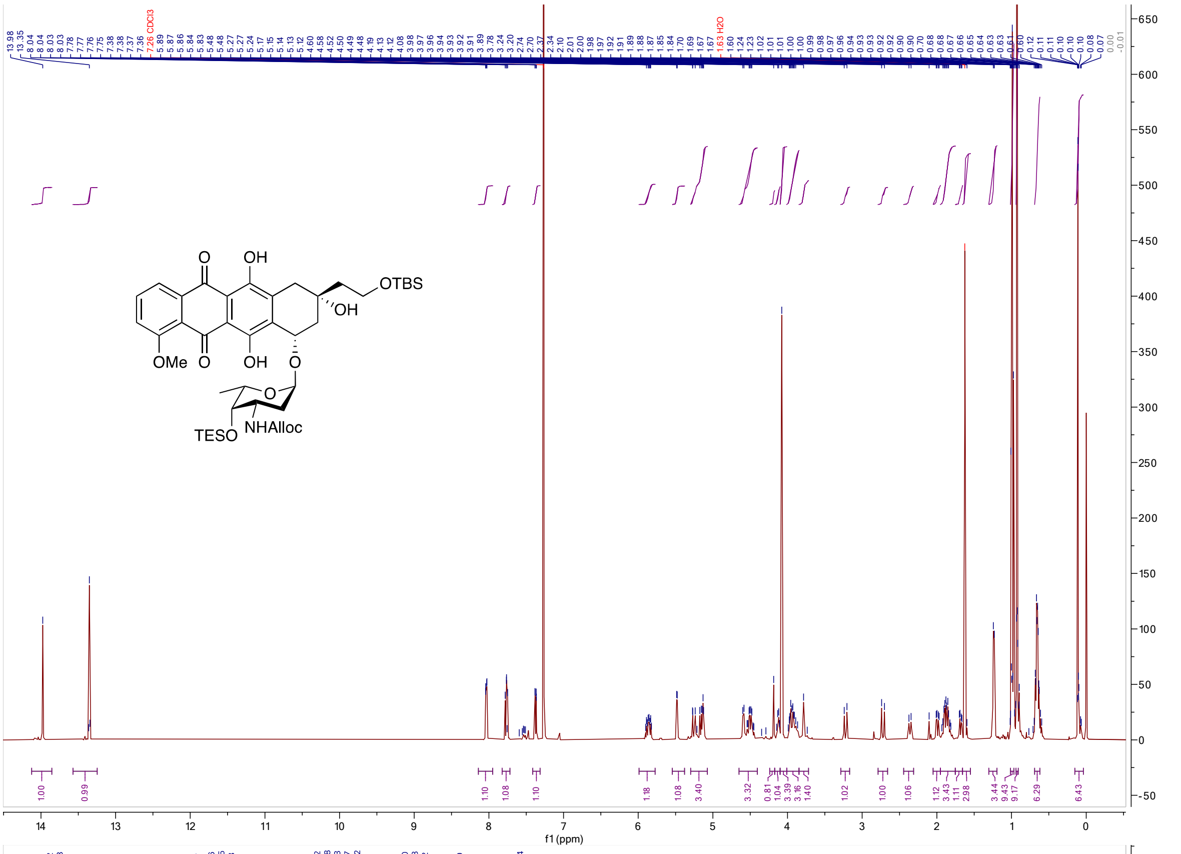
O
OH
OTBS
OH
OMe
O
OH
O
O
TESO
NHAlloc
f1 (ppm)

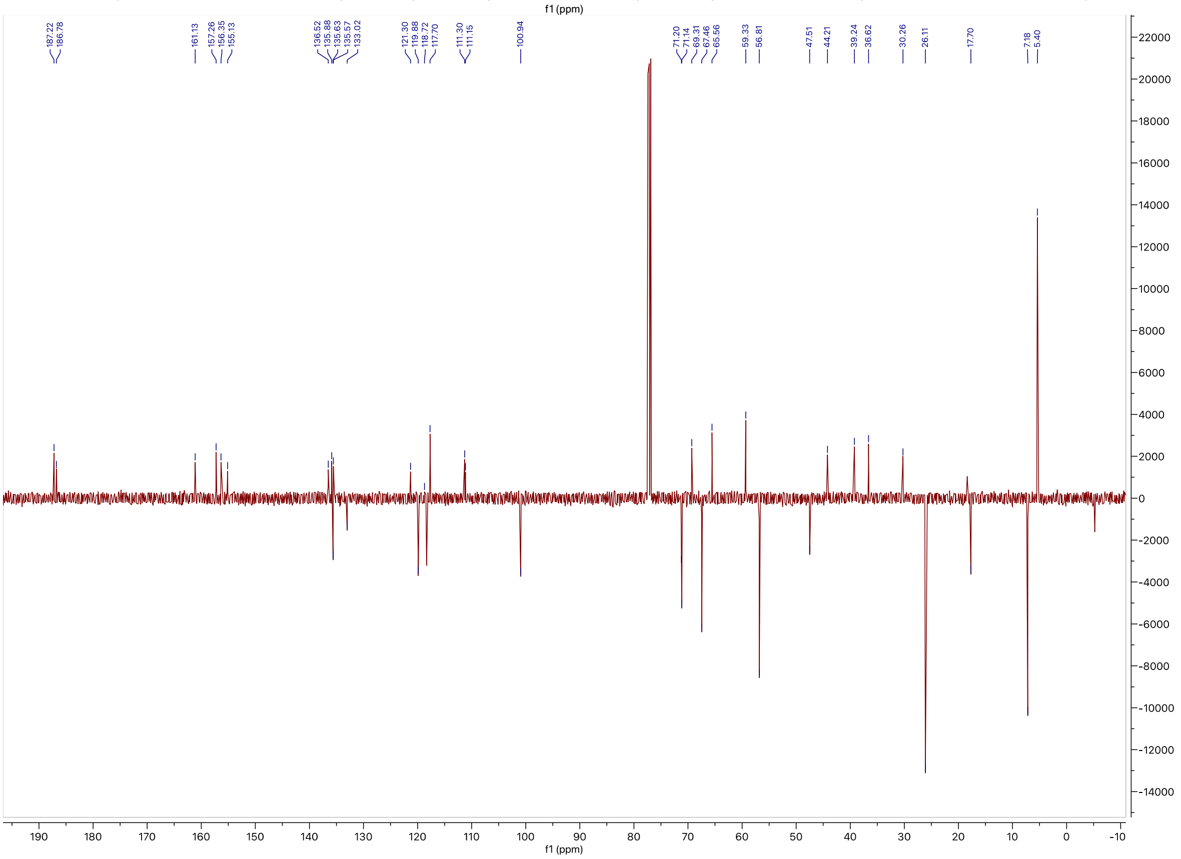
f1 (ppm)

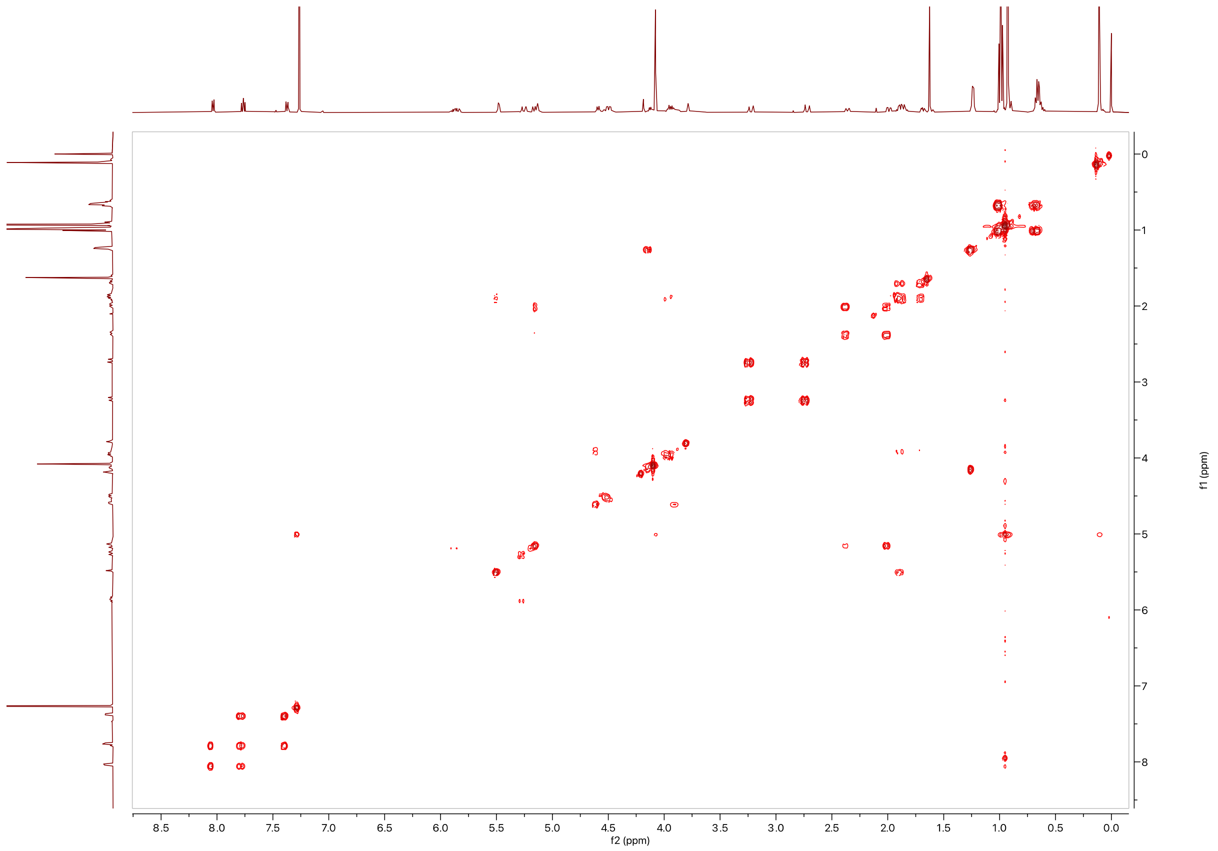
8.5
8.0
7.5
7.0
6.5
6.0
5.5
5.0
4.5
4.0
3.5
3.0
2.5
2.0
1.5
1.0
0.5
0.0
f2 (ppm)
f1 (ppm)

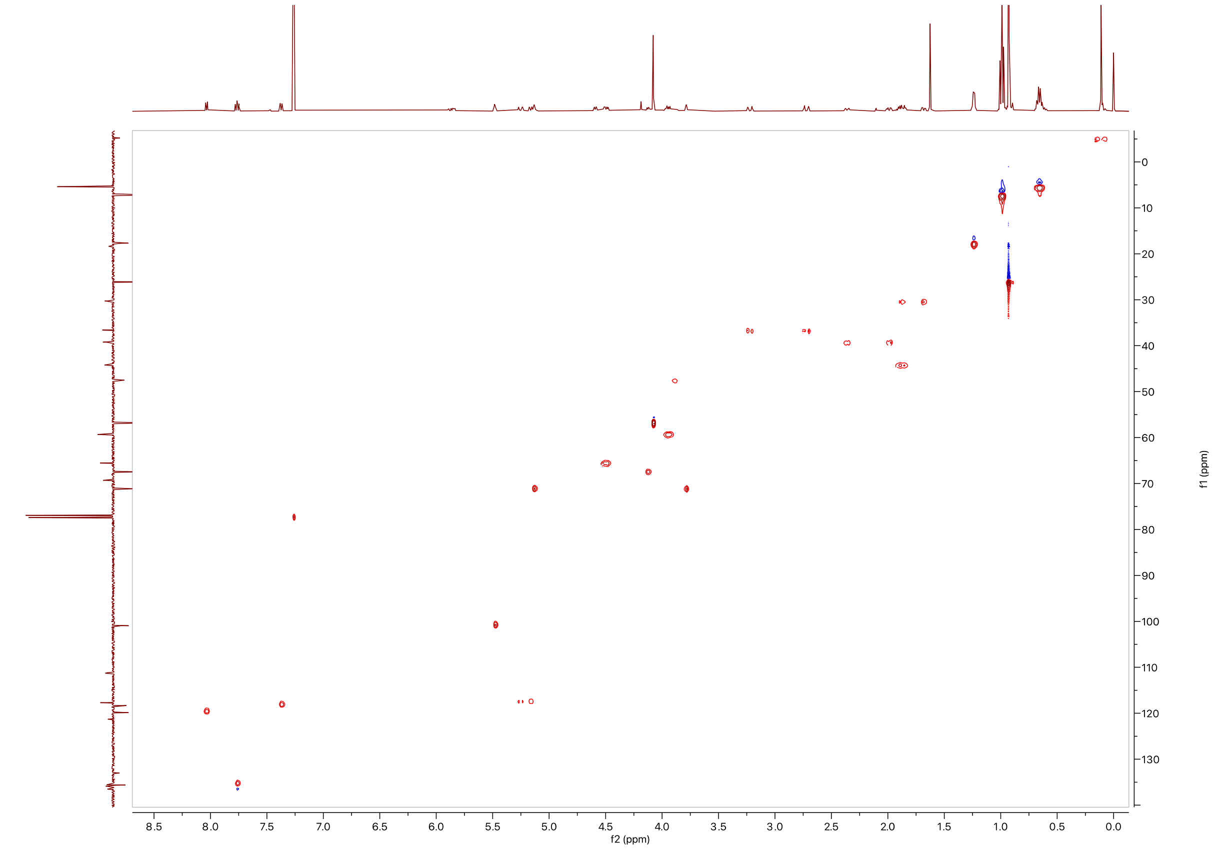
8.5
8.0
7.5
7.0
6.5
6.0
5.5
5.0
4.5
4.0
3.5
3.0
2.5
2.0
1.5
1.0
0.5
0.0
f2 (ppm)
f1 (ppm)

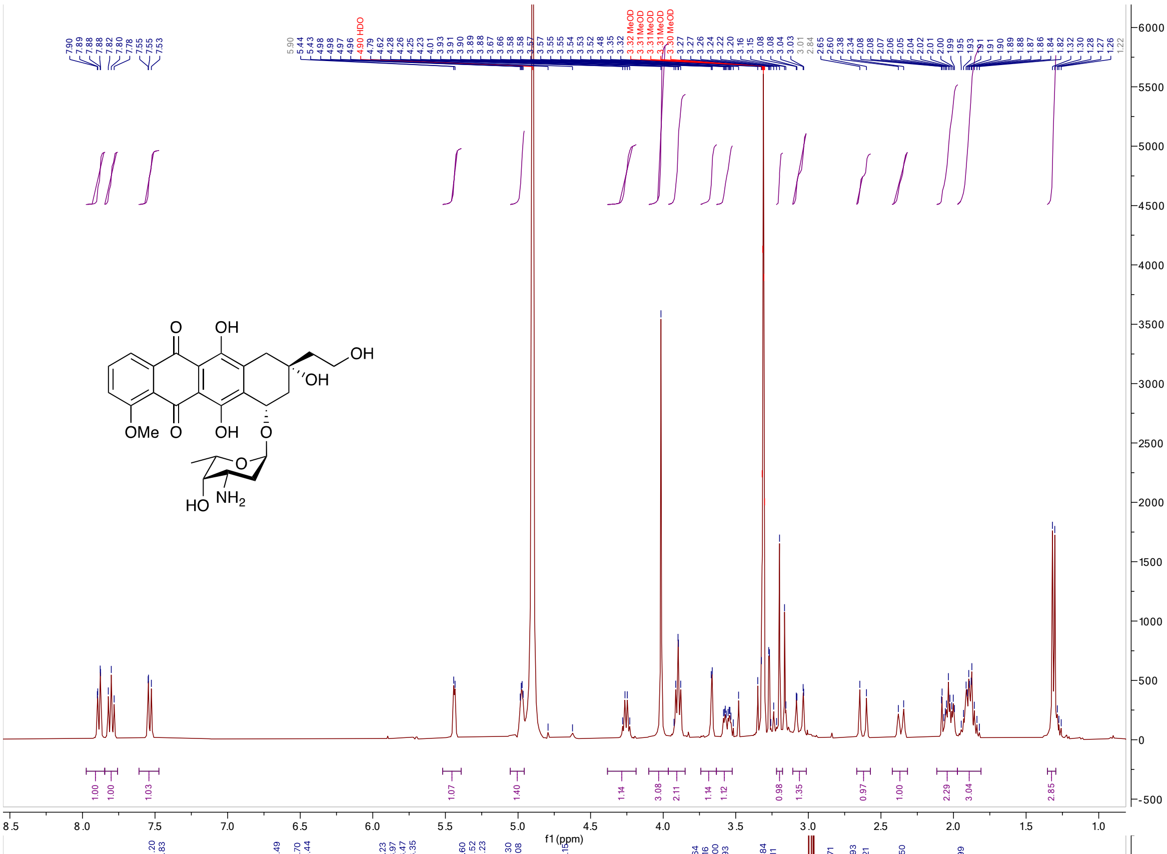
7.90 7.89 7.88 7.82 7.80 7.78 7.55 7.55 7.53
5.90 5.44 5.43 4.98 4.98 4.97 4.96 4.90 HDO 4.79 4.62 4.28 4.26 4.25 4.23 4.01 3.93 3.91 3.90 3.89 3.88 3.67 3.66 3.58 3.57 3.57 3.55 3.55 3.54 3.53 3.52 3.48 3.35 3.32 MeOD 3.31 MeOD 3.31 MeOD 3.31 MeOD 3.30 MeOD 3.27 3.27 3.26 3.24 3.22 3.20 3.16 3.15 3.08 3.08 3.04 3.03 3.01 2.84
2.65 2.60 2.38 2.34 2.08 2.08 2.07 2.06 2.05 2.04 2.02 2.01 2.00 1.99 1.95 1.93 1.91 1.91 1.90 1.89 1.88 1.87 1.86 1.84 1.82 1.32 1.30 1.28 1.27 1.26 1.22
O OH OH OH OMe O OH O O HO NH2
1.00 1.00 1.03 1.07 1.40 1.14 3.08 2.11 1.14 1.12 0.98 1.35 0.97 1.00 2.29 3.04 2.85
f1 (ppm)

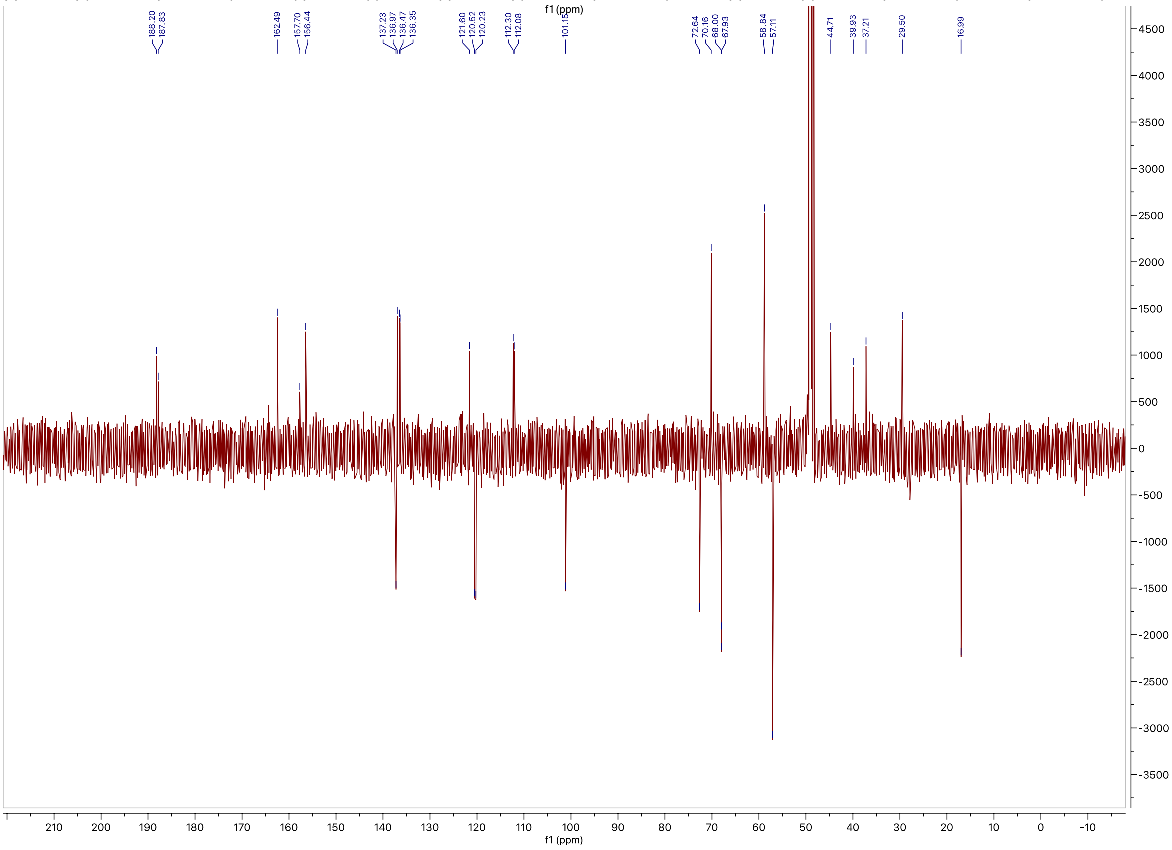
188.20 187.83 162.49 157.70 156.44 137.23 136.97 136.47 136.35 121.60 120.52 120.23 112.30 112.08 101.16 72.64 70.16 68.00 67.93 58.84 57.11 44.71 39.93 37.21 29.50 16.99
f1 (ppm)

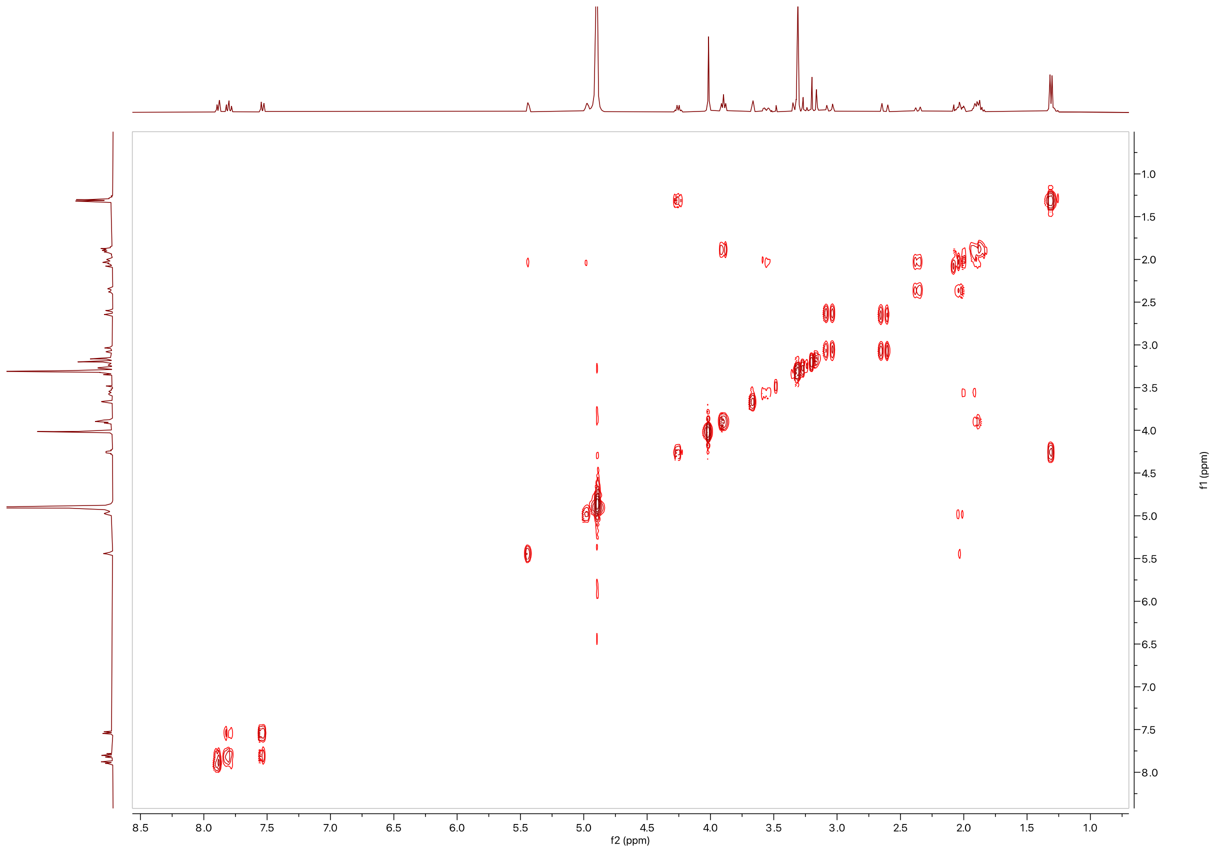

f2 (ppm)
f1 (ppm)

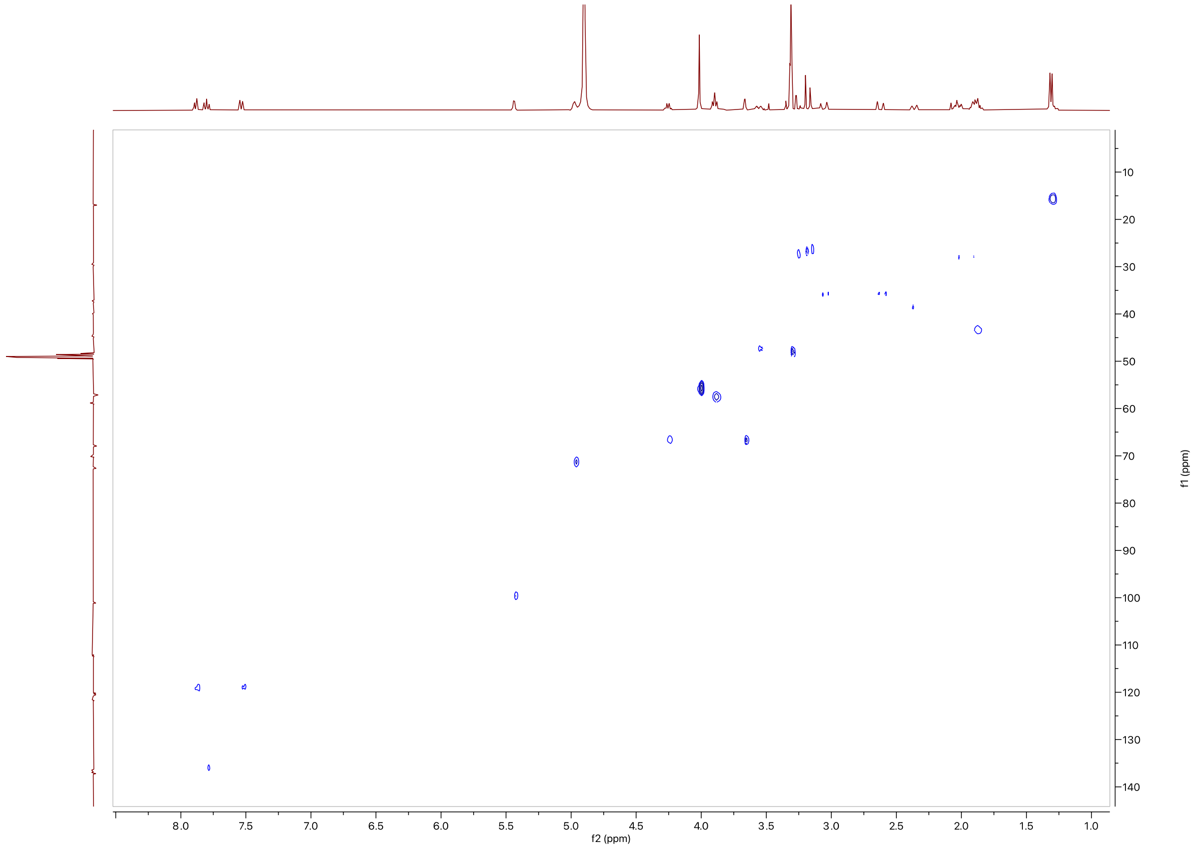

f2 (ppm)
f1 (ppm)

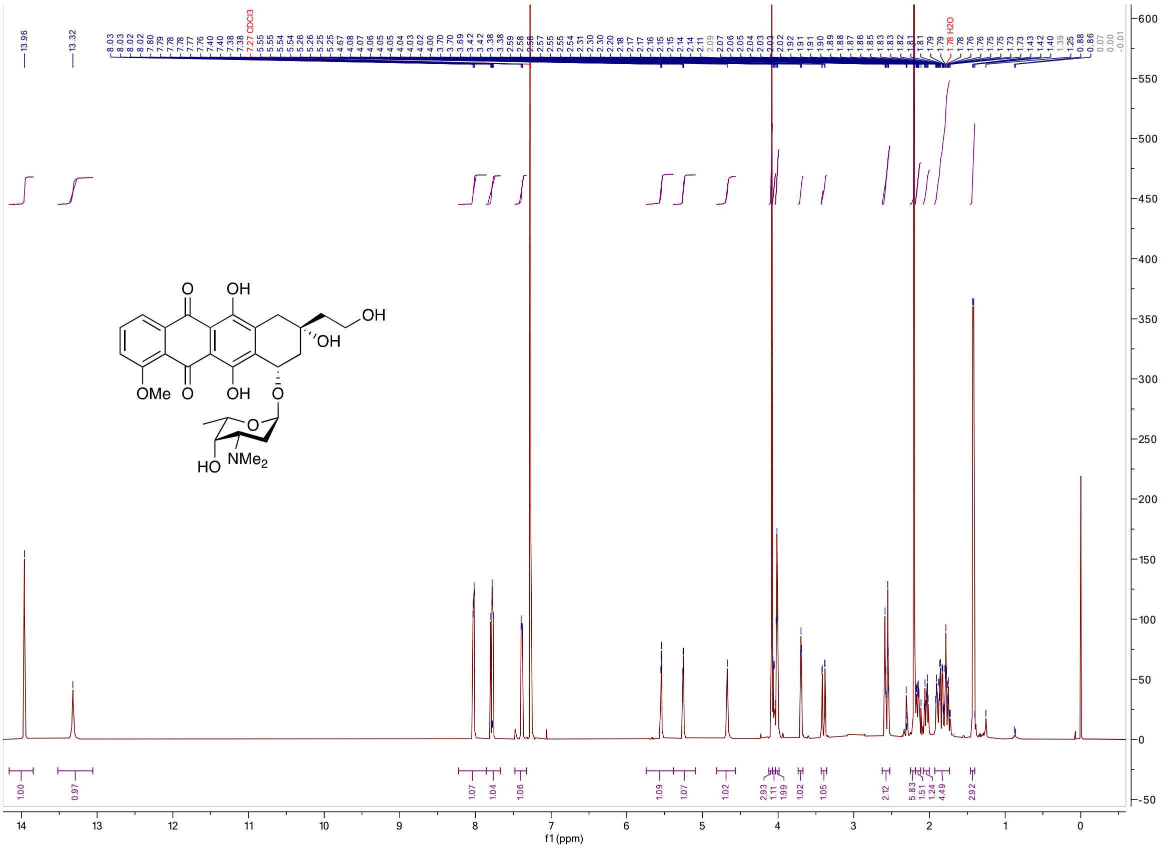

13.96
13.32
7.27 CDCl3
1.78 H2O
OH
OH
OH
OMe
O
O
OH
O
O
NMe2
HO
f1 (ppm)

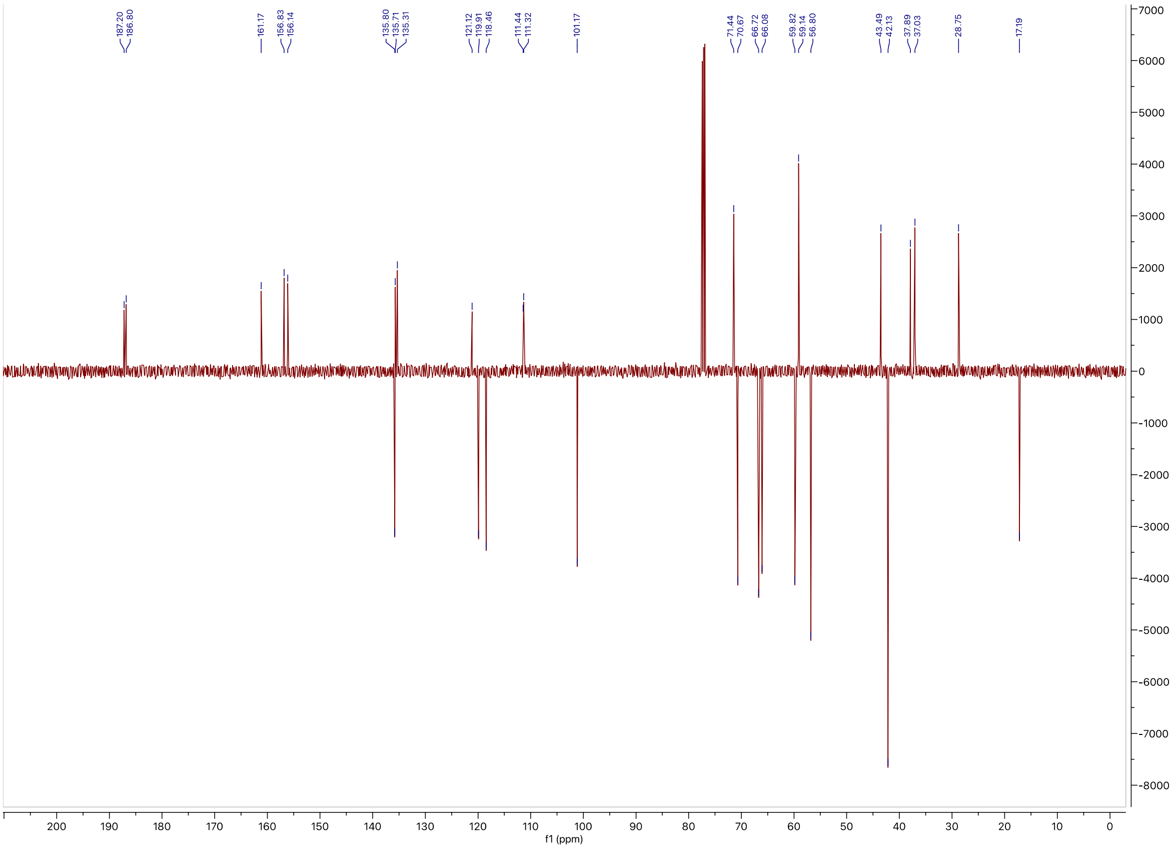

187.20
186.80
161.17
156.83
156.14
135.80
135.71
135.31
121.12
119.91
118.46
111.44
111.32
101.17
71.44
70.67
66.72
66.08
59.82
59.14
56.80
43.49
42.13
37.89
37.03
28.75
17.19
f1 (ppm)

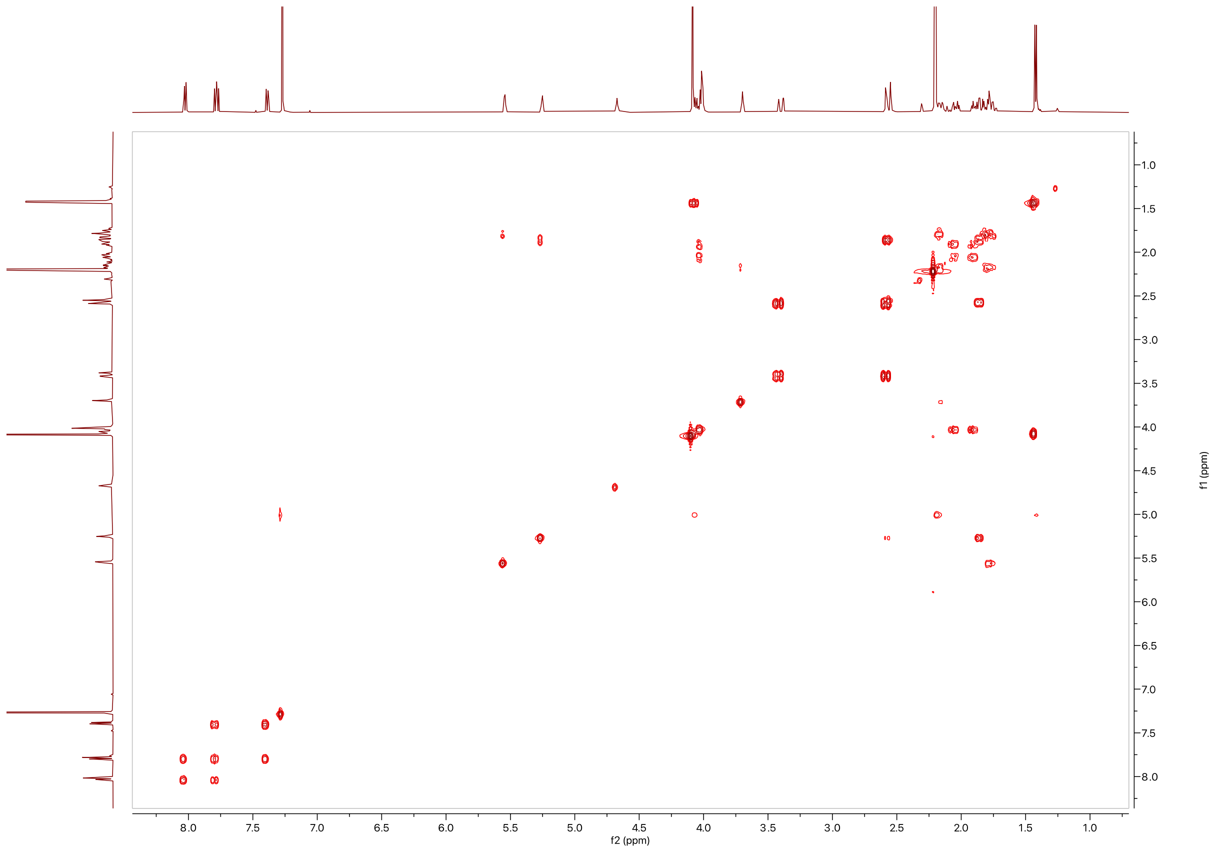
f2 (ppm)
f1 (ppm)

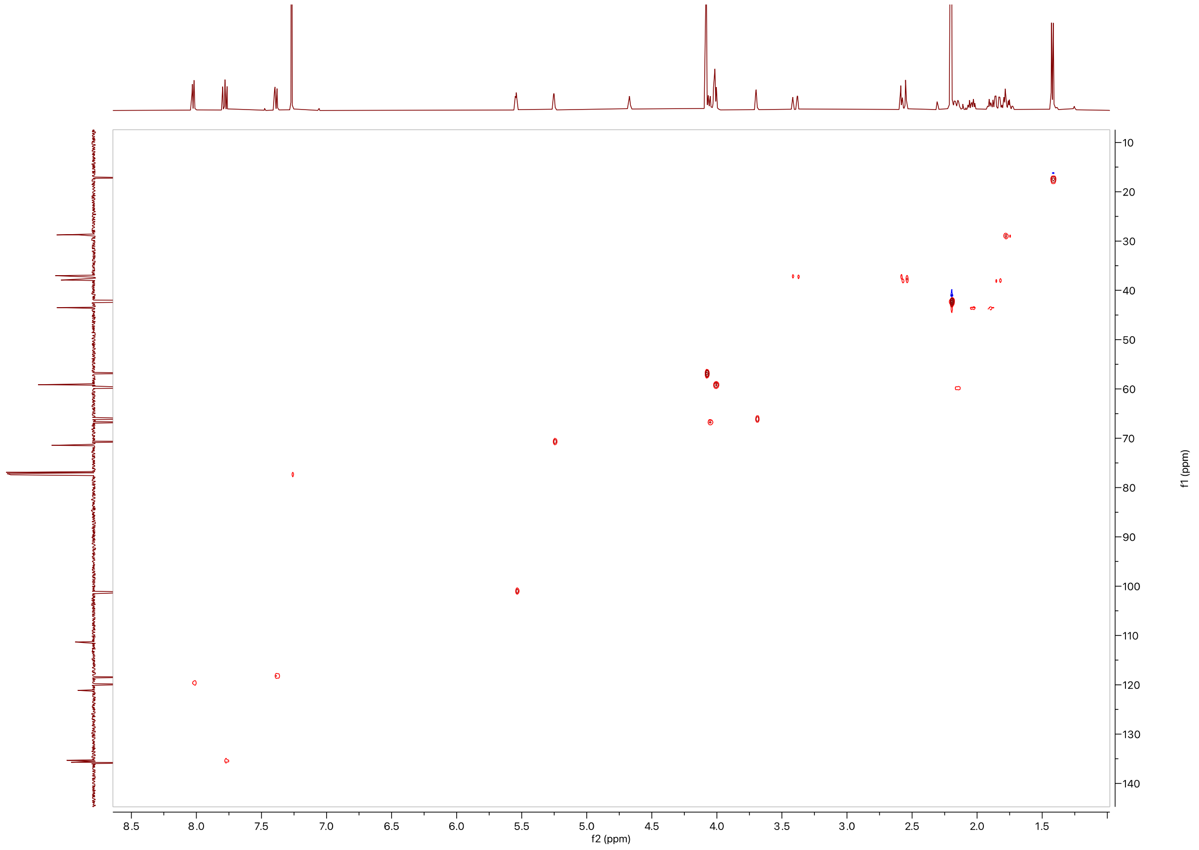
f2 (ppm)
f1 (ppm)

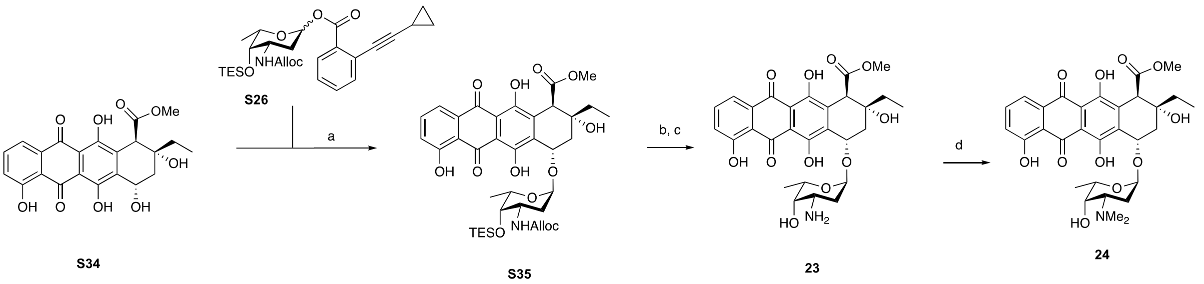

Scheme S8. Synthesis of rhodomycin D (**23**) and rhodomycin T (**24**). *Reagents and conditions:* (a) PPh_3AuNTf_2, DCM, 53%; (b) $Pd(PPh_3)_4$, NDMBA, DCM; (c) HF-pyr, pyr, 73% over 2 steps; (d) aq. CH_2O, $NaBH(OAc)_3$, EtOH, 98%.

ε-rhodomycinone (S34)

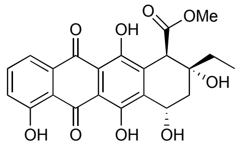

ε-rhodomycinone was obtained from a 4L fermentation by seeding 40 mL of *Streptomyces peucetius* mutant G006. LXA-1180 resin was added 7 days after inoculation of the fermentation and collected 3 days later. The resultant orange resin was four times suspended in 300 mL acetone, sonicated for 5 minutes, and filtered off. The thus obtained filtrates were concentrated *in vacuo* to yield crude ε-rhodomycinone. Column chromatography (0:100 – 5:95 MeOH:DCM) and subsequent column chromatography (2:98 – 4:96 acetone:toluene) gave the title compound as an orange solid (338 mg, 0.79 mmol). Analytical data were in agreement with literature precedence.[13]

7-[3-*N*-allyloxycarbonyl-2,3-dideoxy-4-*O*-triethylsilyl-α-L-fucopyranoside]-ε-rhodomycinone (S35)

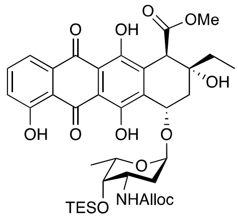

According to general procedure C, glycosyl donor **S26** (257 mg, 0.500 mmol) was glycosylated to acceptor **S34** (321 mg, 0.75 mmol, 1.5 eq). Column chromatography (4:96 Et_2O:pentane and then 0:100 – 2:98 EtOAc:toluene) afforded the title compound as an orange solid (199 mg, 0.26 mmol, 53%). ^1H NMR (400 MHz, $CDCl_3$) δ 13.47 (s, 1H), 12.82 (s, 1H), 12.09 (s, 1H), 7.82 (dd, *J* = 7.5, 1.2 Hz, 1H), 7.67 (dd, *J* = 8.4, 7.5 Hz, 1H), 7.27 (dd, *J* = 8.4, 1.2 Hz, 1H), 5.95 – 5.73 (m, 1H), 5.47 (d, *J* = 3.8 Hz, 1H), 5.30 – 5.11 (m, 3H), 4.67 (d, *J* = 8.7 Hz, 1H), 4.50 (qdt, *J* = 13.3, 5.7, 1.4 Hz, 3H), 4.29 (d, *J* = 1.2 Hz, 2H), 4.12 (q, *J* = 6.5 Hz, 1H), 3.99 – 3.84 (m, 1H), 3.84 – 3.77 (m, 1H), 3.72 (s, 4H), 2.47 – 2.28 (m, 2H), 2.21 (dd, *J* = 15.0, 4.3 Hz, 1H), 1.95 (td, *J* = 12.9, 4.1 Hz, 1H), 1.89 – 1.70 (m, 2H), 1.68 – 1.56 (m, 1H), 1.49 – 1.38 (m, 1H), 1.28 – 1.21 (m, 3H), 1.00 (t, *J* = 7.9 Hz, 9H), 0.92 – 0.81 (m, 3H), 0.66 (qd, *J* = 8.0, 1.9 Hz, 6H). ^{13}C NMR (101 MHz, $CDCl_3$) δ 190.8, 186.1, 171.6, 162.7, 157.0, 155.2, 137.1, 136.0, 135.5, 133.4, 132.9, 124.9, 119.7, 117.7, 116.1, 111.6, 111.3, 101.7, 71.3, 71.1, 71.0, 67.7, 65.6, 52.5, 52.1, 47.4, 34.0, 33.6, 32.4, 24.9, 17.6, 7.1, 6.9, 5.4. HRMS: $[M + Na]^+$ calculated for $C_{38}H_{49}NO_{13}Si$ 778.2871; found 778.2865.

E-Rhodomycin D (23)

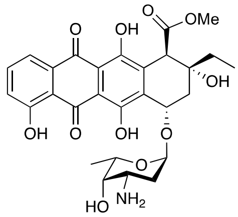

According to general procedure D, **S35** (194 mg, 0.26 mmol) was deprotected. Column chromatography (0:100 – 20:80 acetone:DCM) gave the crude amine. The silyl ether was then removed according to general procedure E. Column chromatography on neutral silica gel (0:100 – 20:80 MeOH:DCM) gave the title compound as a dark orange solid (106 mg, 0.190 mmol, 73% over two steps).

Analytical data were in agreement with literature precedence. [14] ^{1}H NMR (500 MHz, MeOD) δ 7.72 (d, *J* = 7.4 Hz, 1H), 7.66 (t, *J* = 7.9 Hz, 1H), 7.22 (d, *J* = 8.3 Hz, 1H), 5.45 (s, 1H), 5.14 (dd, *J* = 4.8, 1.7 Hz, 1H), 4.32 – 4.13 (m, 2H), 3.69 (s, 3H), 3.60 – 3.51 (m, 1H), 3.25 – 3.19 (m, 1H), 2.36 (d, *J* = 15.0 Hz, 1H), 2.18 (dd, *J* = 15.0, 4.8 Hz, 1H), 1.93 – 1.86 (m, 2H), 1.82 (dq, *J* = 14.5, 7.3 Hz, 1H), 1.49 (dq, *J* = 14.3, 7.2 Hz, 1H), 1.29 (d, *J* = 6.6 Hz, 4H), 1.13 (t, *J* = 7.3 Hz, 3H). ^{13}C NMR (126 MHz, MeOD) δ 187.4, 172.7, 163.8, 125.7, 120.3, 112.7, 112.4, 102.5, 72.4, 71.8, 70.5, 68.6, 53.1, 52.8, 48.1, 35.0, 33.6, 32.2, 17.2, 7.3. HRMS: $[M + H]^+$ calculated for $C_{28}H_{31}NO_{11}$ 558.1975; found 558.1969.

E-Rhodomycin T (24)

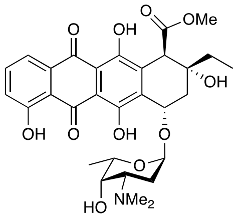

To a solution of 23 (61 mg, 0.11 mmol) in EtOH (11 mL) was added 37% aq. CH_2O (0.28 mL) and the mixture was stirred for 10 minutes before addition of $NaBH(OAc)_3$ (117 mg, 0.55 mmol, 5 eq). After stirring for 2 hours, the mixture was poured into sat. aq. $NaHCO_3$ and extracted with $CHCl_3$. The organic layer was dried over Na_2SO_4 and concentrated *in vacuo*. Column chromatography on neutral silica gel (5:95 – 10:90 MeOH:DCM) gave the title compound as a dark orange solid (62 mg, 0.11 mmol, 97%). ^{1}H NMR (500 MHz, $CDCl_3$) δ 13.47 (s, 1H), 12.85 (s, 1H), 12.08 (s, 1H), 7.82 (dd, *J* = 7.5, 1.1 Hz, 1H), 7.68 (dd, *J* = 8.4, 7.5 Hz, 1H), 7.29 (d, *J* = 1.1 Hz, 1H), 5.60 – 5.46 (m, 1H), 5.25 (dd, *J* = 4.2, 1.9 Hz, 1H), 4.54 (s, 1H), 4.28 (d, *J* = 1.2 Hz, 1H), 4.09 (qd, *J* = 6.5, 1.2 Hz, 1H), 3.72 (s, 4H), 2.39 (dt, *J* = 15.0, 1.8 Hz, 1H), 2.27 – 2.18 (m, 8H), 1.95 – 1.76 (m, 3H), 1.47 (dp, *J* = 14.3, 7.3 Hz, 1H), 1.40 (d, *J* = 6.6 Hz, 3H), 1.14 (t, *J* = 7.3 Hz, 3H). ^{13}C NMR (126 MHz, $CDCl_3$) δ 190.8, 186.1, 171.4, 162.7, 157.0, 157.0, 137.2, 136.1, 135.5, 133.4, 124.9, 119.8, 116.1, 111.6, 111.3, 101.7, 71.4, 71.2, 66.7, 66.1, 59.8, 52.6, 52.3, 42.1, 33.4, 32.4, 28.8, 17.1, 6.9. HRMS: $[M + H]^+$ calculated for $C_{30}H_{35}NO_{11}$ 586.2288; found 586.2282.

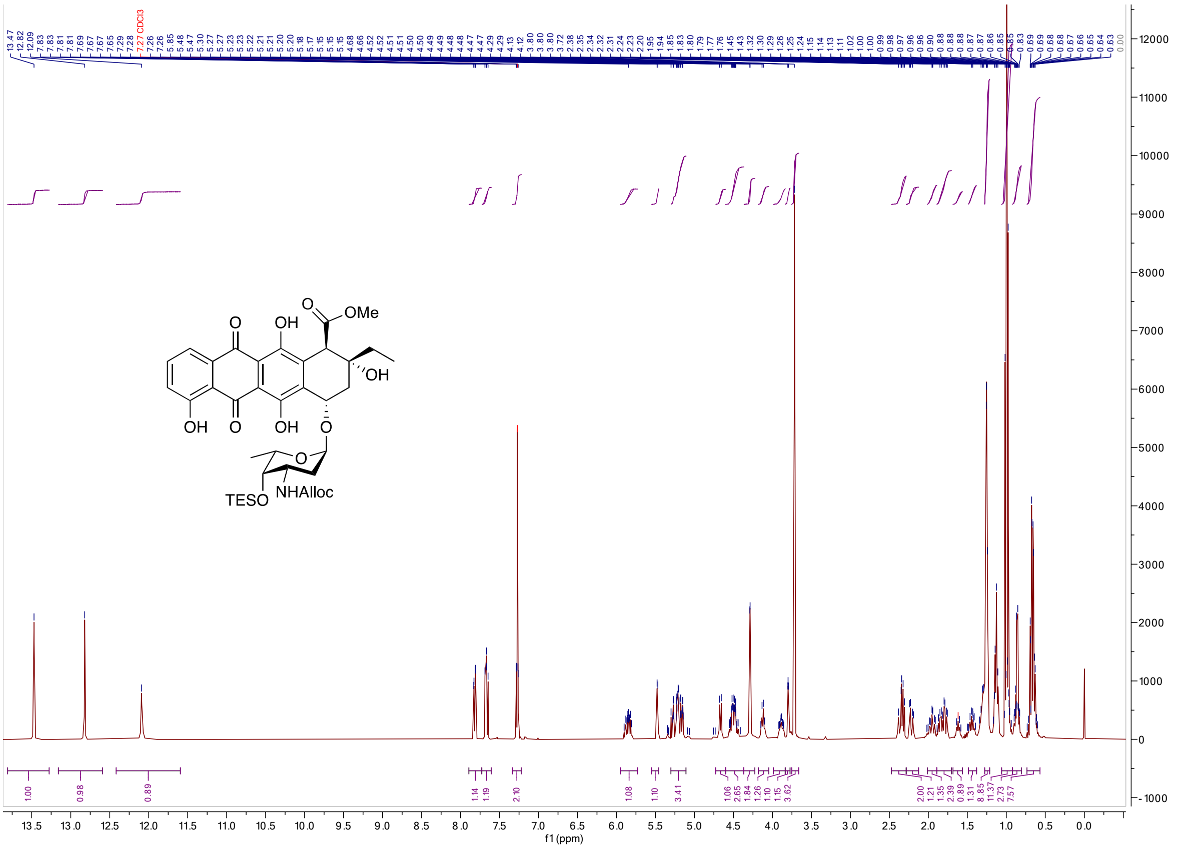
OMe
O
OH
O
OH
OH
O
OH
O
TESO
NHAlloc
7.27 CDCl3
1.00
0.98
0.89
1.14
1.19
2.10
1.08
1.10
3.41
1.06
2.65
1.84
1.26
1.10
1.15
3.62
2.00
1.21
1.35
2.39
0.89
1.31
8.85
11.37
2.73
7.57
f1 (ppm)

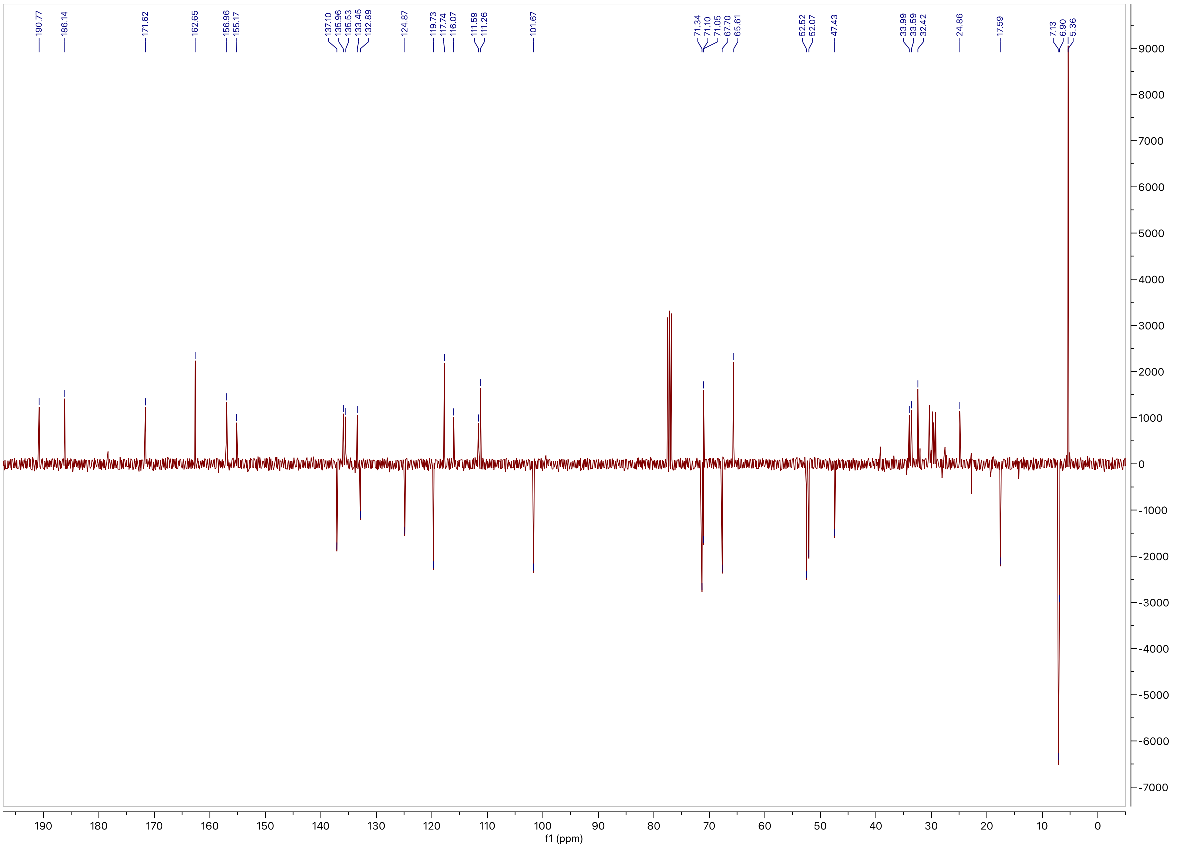
190.77
186.14
171.62
162.65
156.96
155.17
137.10
135.96
135.53
133.45
132.89
124.87
119.73
117.74
116.07
111.59
111.26
101.67
71.34
71.10
71.05
67.70
65.61
52.52
52.07
47.43
33.99
33.59
32.42
24.86
17.59
7.13
6.90
5.36
f1 (ppm)

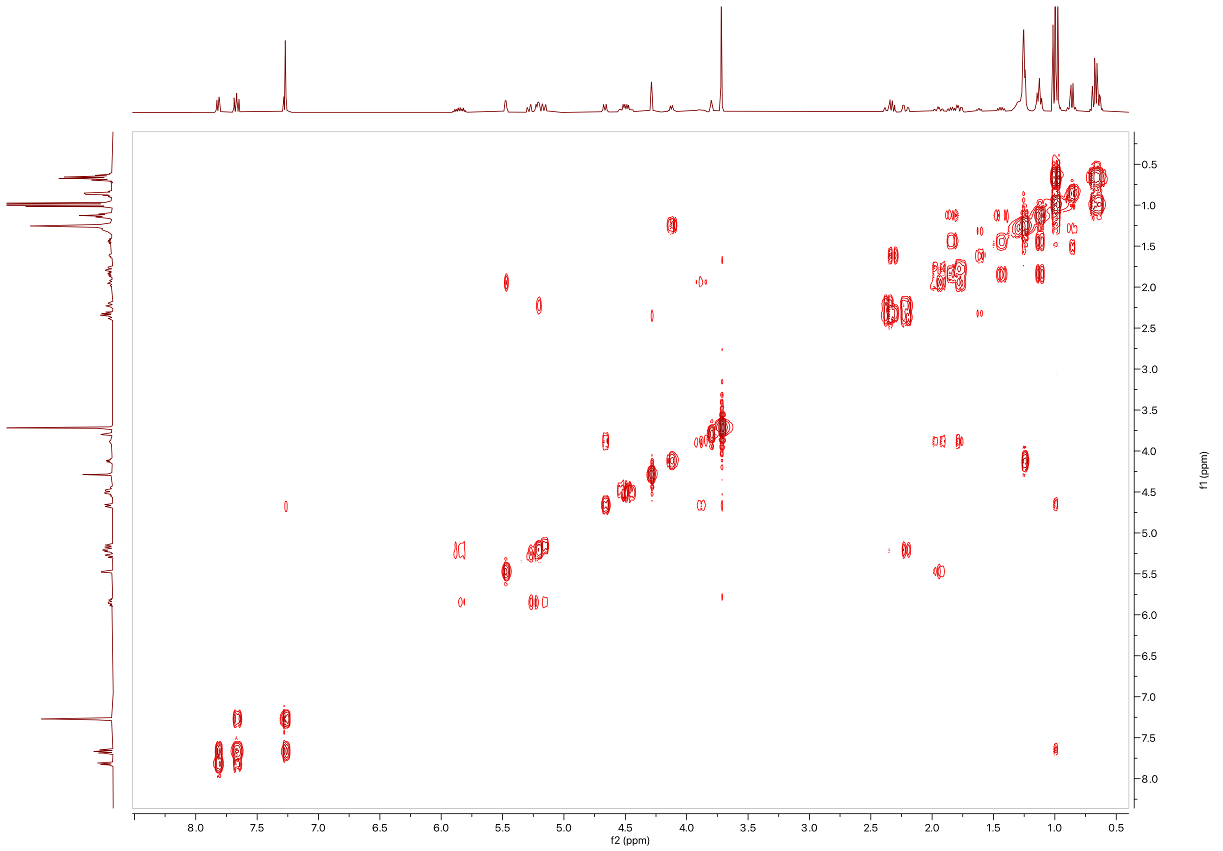
f2 (ppm)
f1 (ppm)

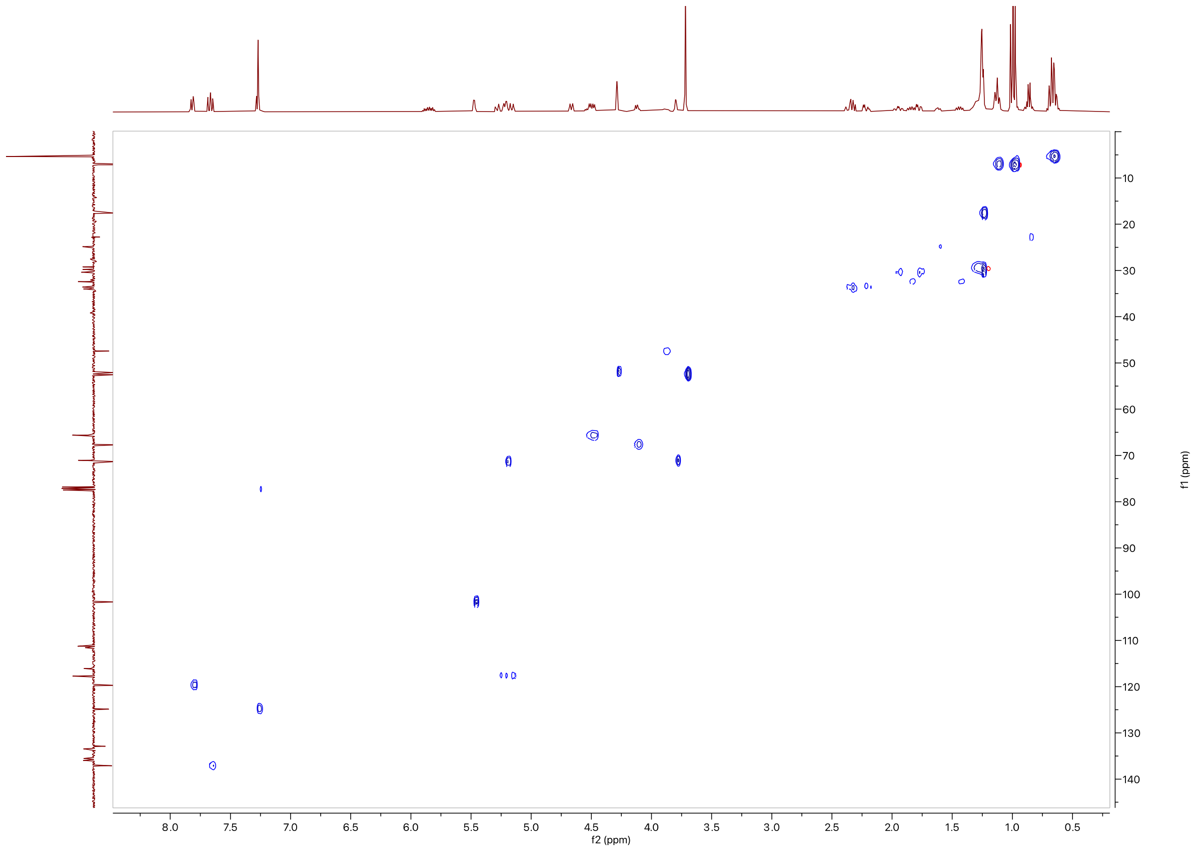
f2 (ppm)
f1 (ppm)

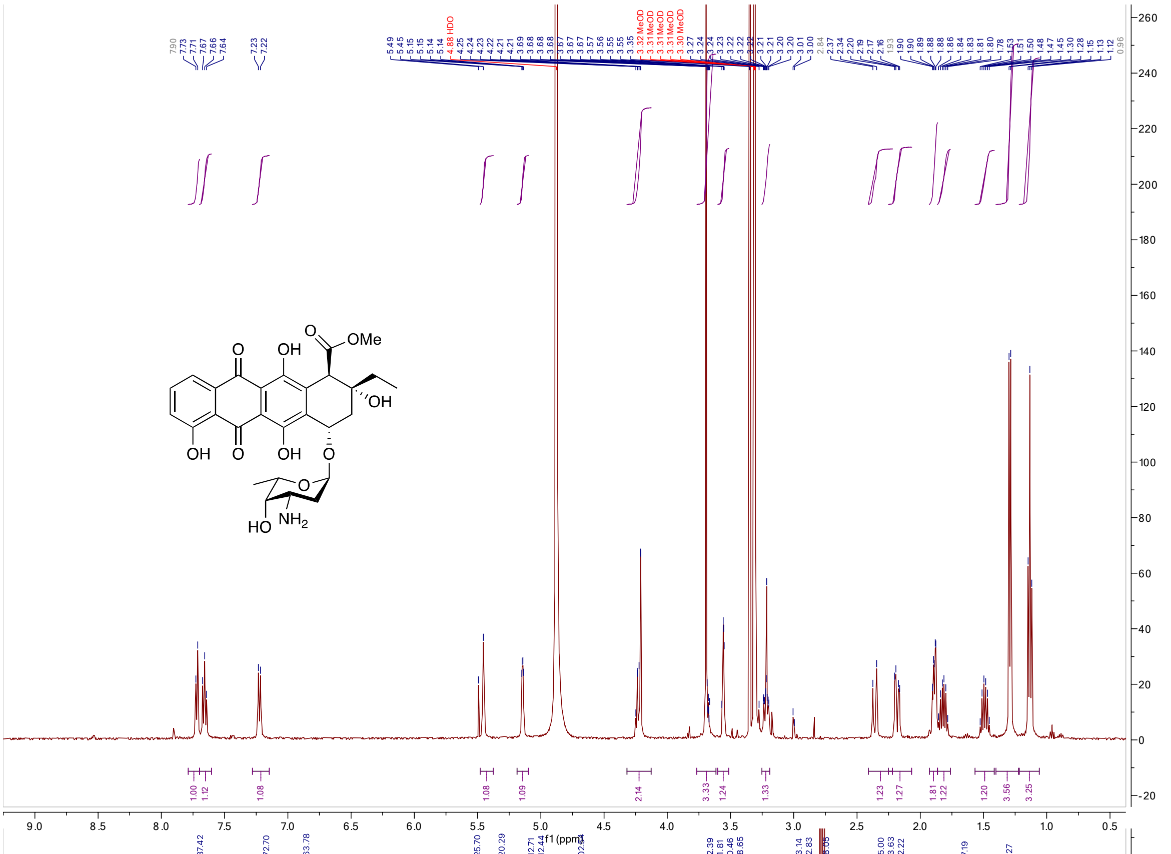
O
OMe
OH
OH
OH
O
OH
O
HO
NH2
f1 (ppm)

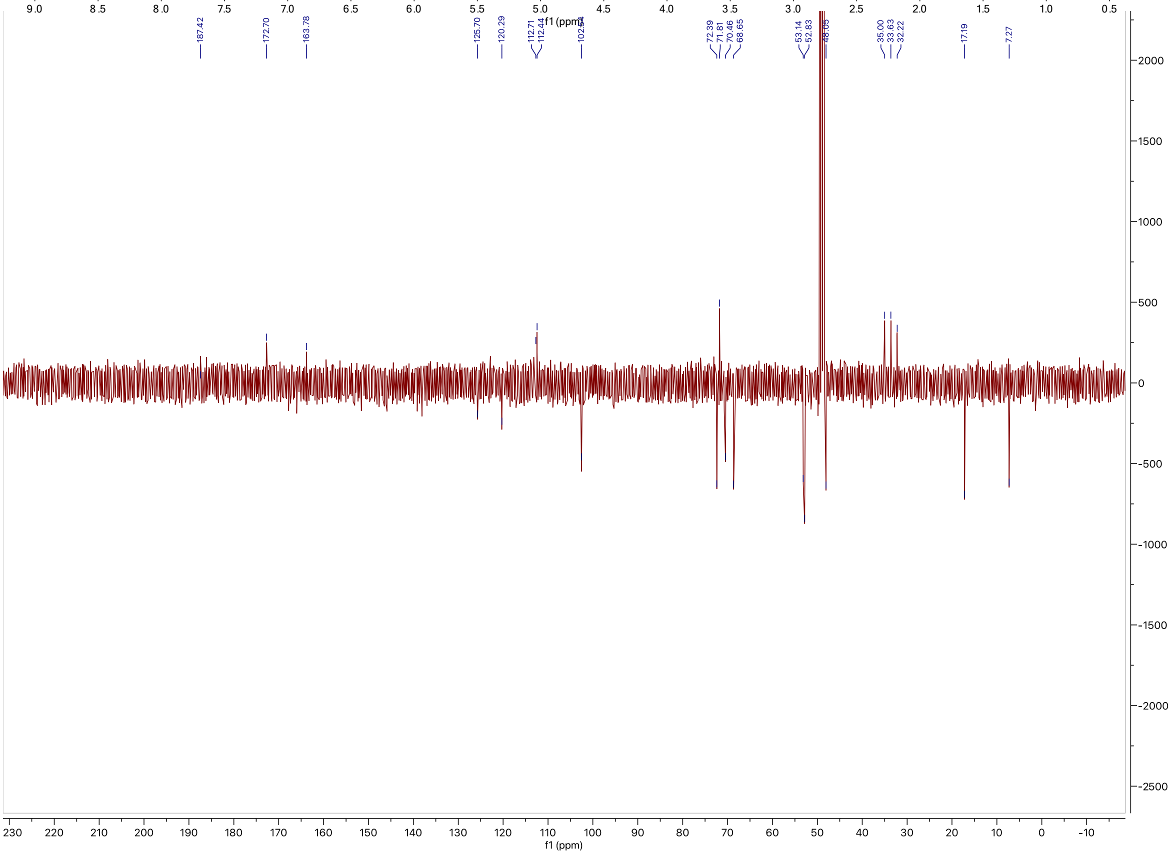
187.42
172.70
163.78
125.70
120.29
112.71
112.44
72.39
71.81
70.46
68.65
53.14
52.83
35.00
33.63
32.22
17.19
7.27
f1 (ppm)

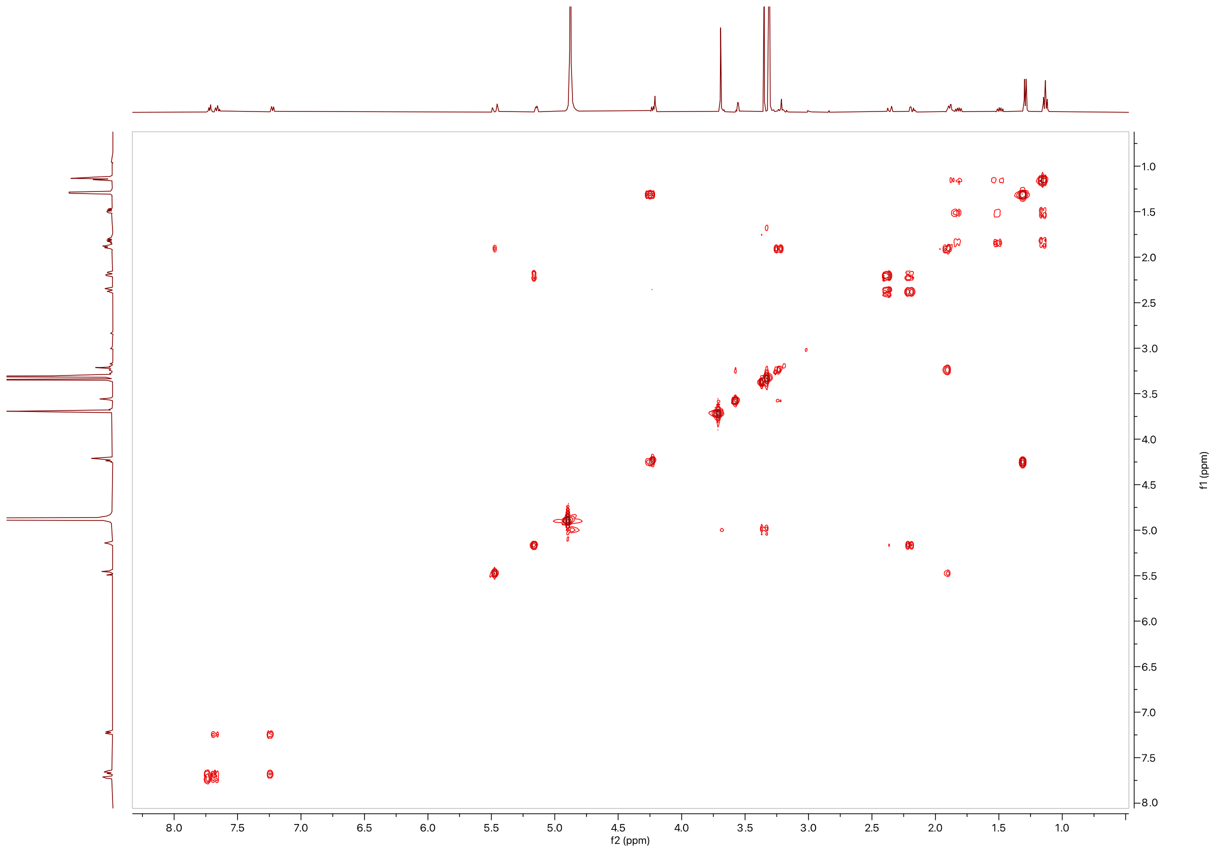
8.0
7.5
7.0
6.5
6.0
5.5
5.0
4.5
4.0
3.5
3.0
2.5
2.0
1.5
1.0
f2 (ppm)
f1 (ppm)

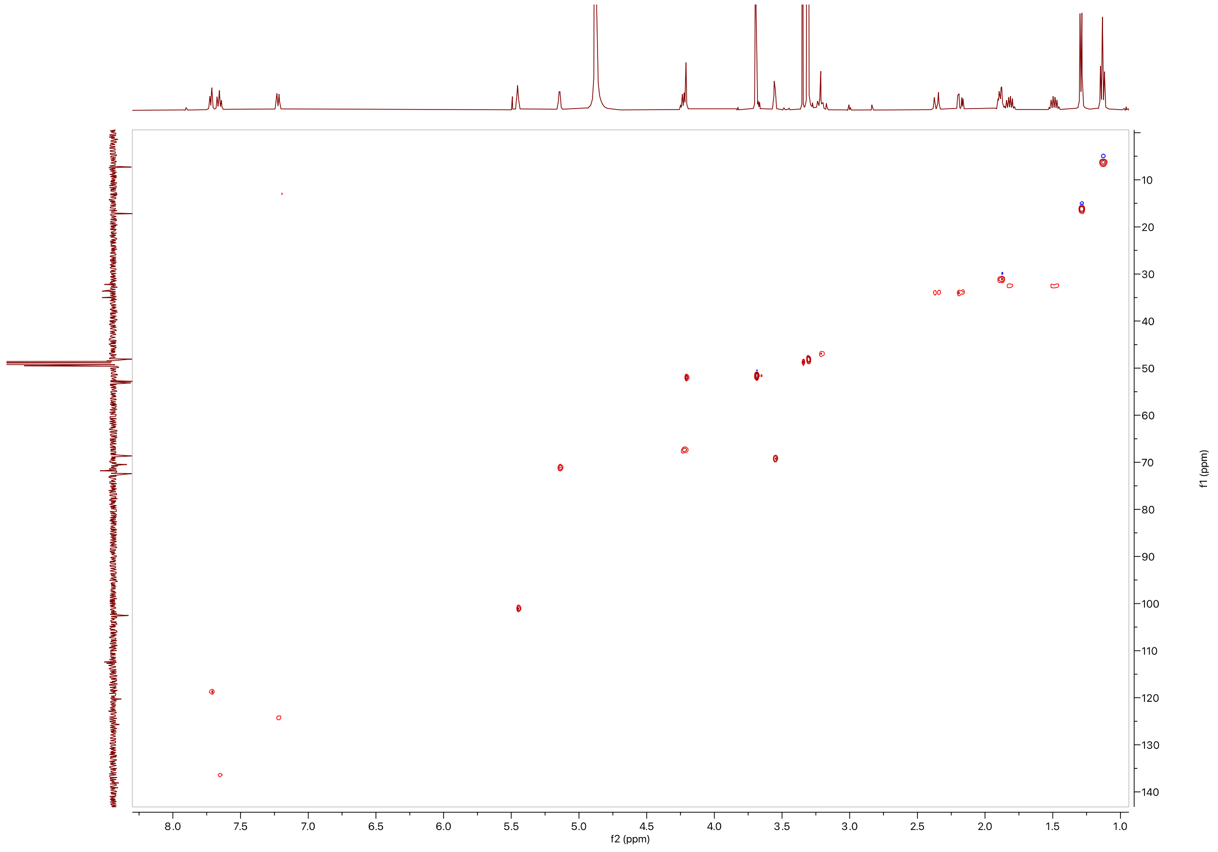
8.0
7.5
7.0
6.5
6.0
5.5
5.0
4.5
4.0
3.5
3.0
2.5
2.0
1.5
1.0
f2 (ppm)
f1 (ppm)

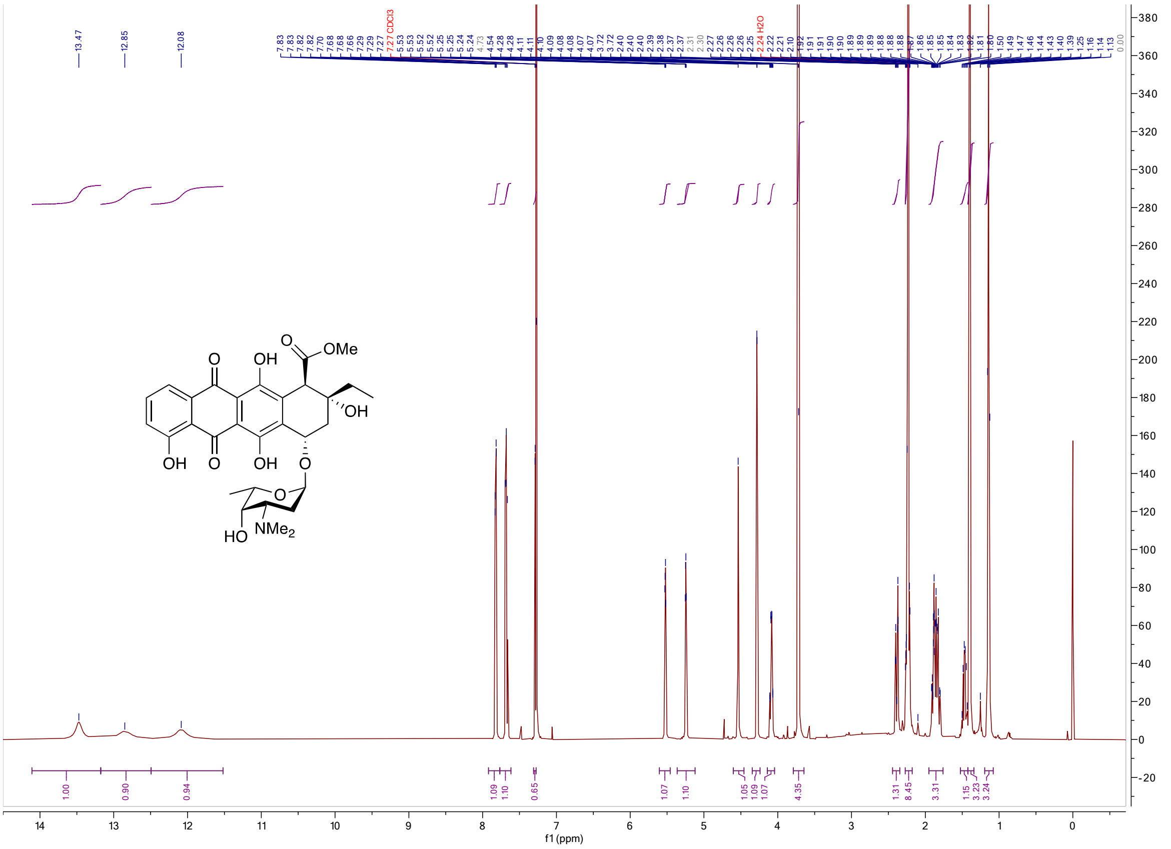
OMe
OH
OH
OH
OH
HO
NMe2
f1 (ppm)

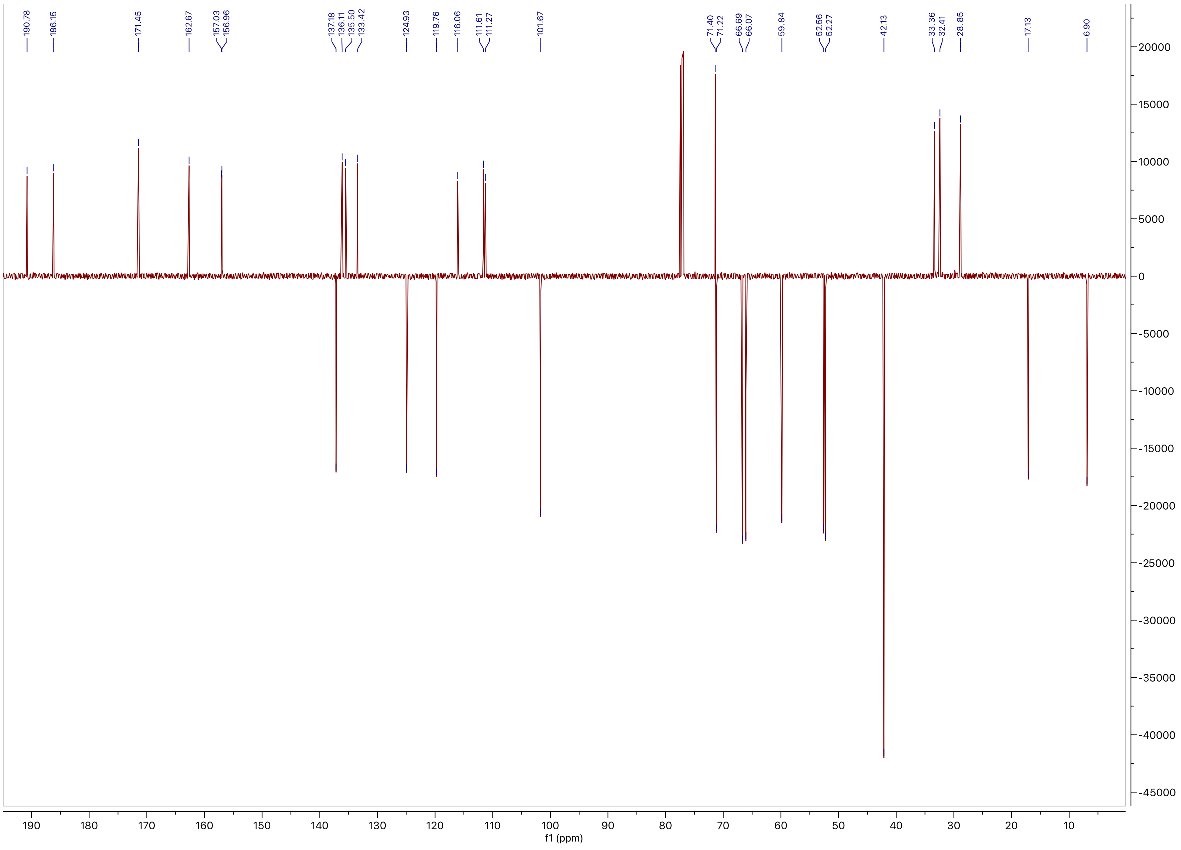
f1 (ppm)

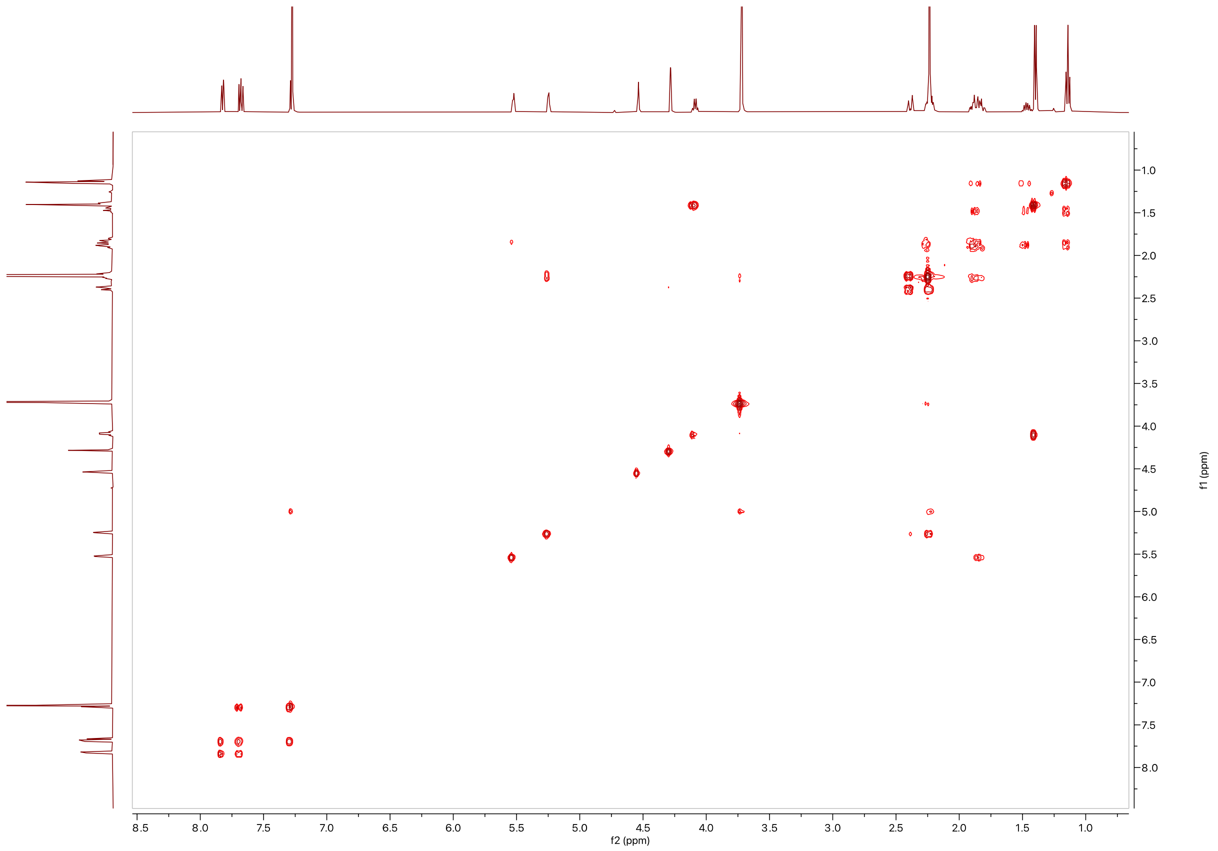
f2 (ppm)
f1 (ppm)

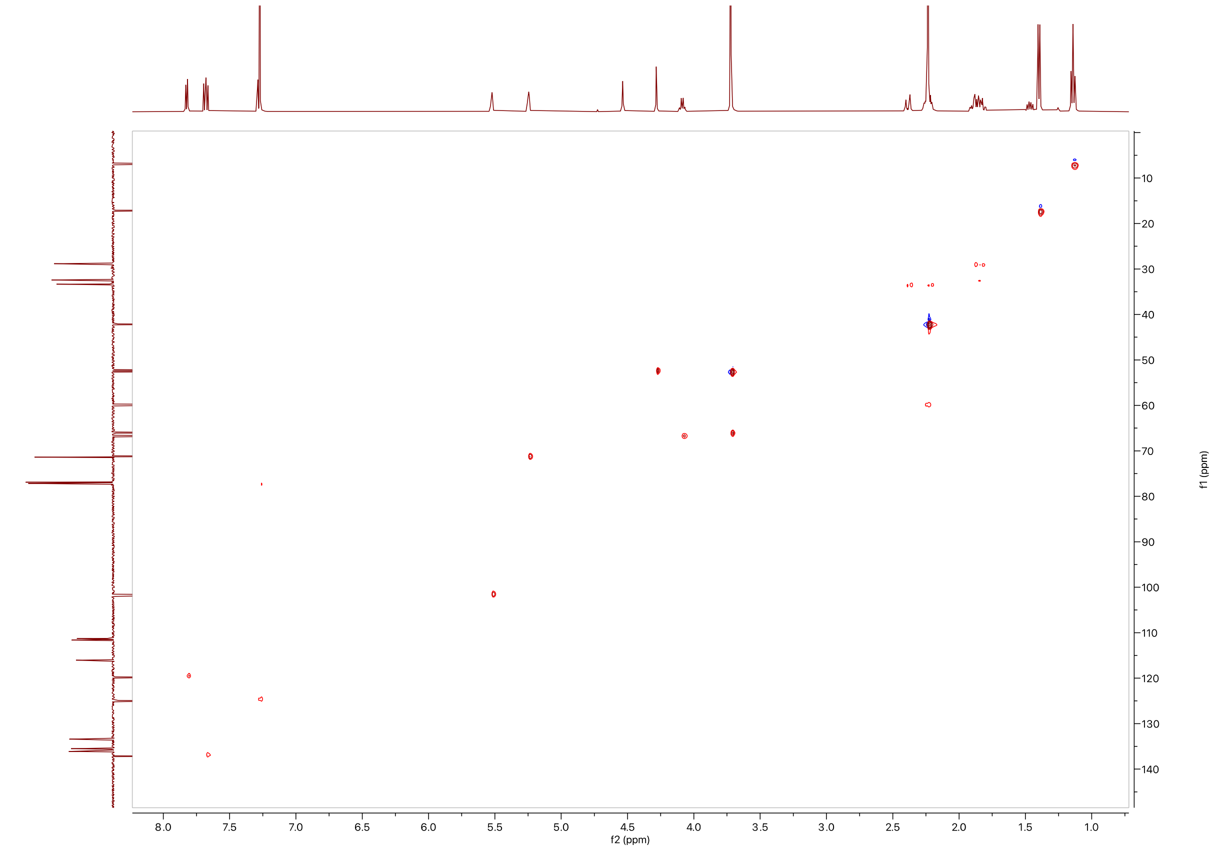
f2 (ppm)
f1 (ppm)

D: Synthesis of idarubicinone trisaccharides 25 and 26 and accompanying analytical data

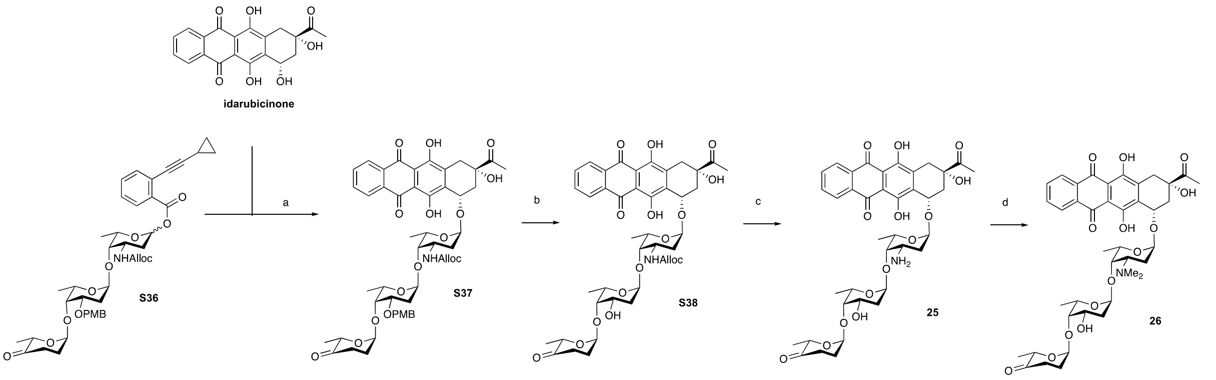

Scheme S9. Synthesis of idarubicinone-trisaccharides **(25)** and **(26)**. *Reagents and conditions:* (a) idarubicinone, PPh_3AuNTf_2, DCM, 79%; (b) DDQ, DCM/pH=7 phosphate buffer, 28%; (c) $Pd(PPh_3)_4$, NDMBA, DCM 49%; (d) aq. CH_2O, $NaBH(OAc)_3$, EtOH, 60%.

7-[2,3-Dideoxy-4-ulo-α-L-fucopyranosyl-2-deoxy-3-O-*p*-methoxybenzyl-α-L-fucopyranosyl-(1→4)-3-*N*-allyloxycarbonyl-2,3-dideoxy-α-L-fucopyranoside]-idarubicinone (S37)

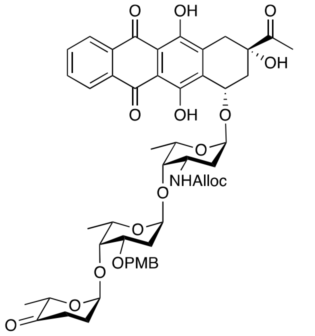

According to general procedure C, glycosyl donor **S36**[4] (278 mg, 0.362 mmol) was glycosylated to idarubicinone (160 mg, 0.434 mmol, 1.2 eq) in DCM (14.4 mL). Column chromatography (1:99 – 8:92 acetone:DCM) afforded the title compound as an orange solid (270 mg, 0.286 mmol, 79%). ^{1}H NMR (400 MHz, $CDCl_3$) δ 13.59 (d, *J* = 1.2 Hz, 1H), 13.34 (s, 1H), 8.35 (ddt, *J* = 6.9, 5.8, 3.4 Hz, 2H), 7.85 (ddd, *J* = 6.3, 3.3, 1.0 Hz, 2H), 7.26 (d, *J* = 8.7 Hz, 2H), 6.96 – 6.82 (m, 2H), 6.12 (d, *J* = 8.0 Hz, 1H), 5.84 (ddt, *J* = 16.3, 10.8, 5.6 Hz, 1H), 5.51 (d, *J* = 3.7 Hz, 1H), 5.30 (s, 1H), 5.27 – 5.12 (m, 2H), 5.10 (t, *J* = 4.4 Hz, 1H), 4.99 (s, 1H), 4.73 – 4.41 (m, 6H), 4.23 – 4.14 (m, 1H), 4.03 (t, *J* = 6.6 Hz, 1H), 3.99 (d, *J* = 2.8 Hz, 1H), 3.97 – 3.85 (m, 2H), 3.82 (s, 3H), 3.57 (s, 1H), 3.27 (d, *J* = 18.6 Hz, 1H), 2.99 (d, *J* = 19.0 Hz, 1H), 2.60 (ddd, *J* = 15.0, 8.7, 5.5 Hz, 1H), 2.43 (d, *J* = 6.6 Hz, 4H), 2.37 – 2.23 (m, 2H), 2.24 – 2.03 (m, 4H), 1.94 (dd, *J* = 12.9, 4.5 Hz, 1H), 1.75 (td, *J* = 12.9, 3.9 Hz, 1H), 1.35 – 1.19 (m, 6H), 0.98 (d, *J* = 6.7 Hz, 3H). ^{13}C NMR (101 MHz, $CDCl_3$) δ 212.2, 211.2, 187.0, 186.8, 159.3, 156.7, 156.6, 155.7, 136.1, 134.7, 133.6, 133.0, 130.4, 129.1, 127.3, 127.1, 117.6, 114.0, 111.6, 111.0, 101.5, 100.3, 98.0, 81.0, 75.1, 72.4, 71.9, 70.4, 69.2, 68.6, 68.1, 65.6, 55.4, 46.6, 35.1, 34.0, 33.9, 31.5, 31.1, 29.5, 25.0, 17.5, 17.3, 14.9. HRMS: $[M + Na]^+$ calculated for $C_{50}H_{57}NO_{17}$ 966.3524; found 966.3514.

7-[2,3-Dideoxy-4-ulo-α-L-fucopyranosyl-2-deoxy-α-L-fucopyranosyl-(1→4)-3-*N*-allyloxycarbonyl-2,3-dideoxy-α-L-fucopyranoside]-idarubicinone (S38)

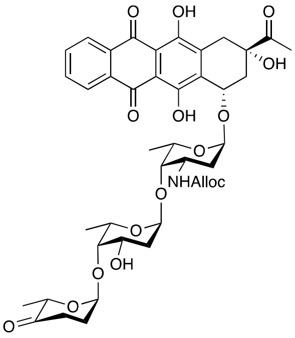

To a biphasic mixture compound **S37** (270 mg, 0.286 mmol) in DCM (48 mL) and phosphate buffer (4.8 mL, pH=7) was added DDQ (325 mg, 1.43 mmol, 5 eq) at 0ºC after which the mixture was stirred at that temperature for 5 hours. It was diluted with DCM, washed with H_2O four times, after which the organic layer was dried over Na_2SO_4 and concentrated *in vacuo*. Column chromatography (5:95 - 7:93 acetone:DCM) gave the title compound as a red solid (66 mg, 0.080 mmol, 28%). ^{1}H NMR (500 MHz, $CDCl_3$) δ 13.56 (s, 1H), 13.33 (s, 1H), 8.50 – 8.24 (m, 2H), 7.84 (dd, *J* = 5.9, 3.3 Hz, 2H), 6.05 (d, *J* = 7.9 Hz, 1H), 5.84 (ddt, *J* = 16.4, 10.8, 5.6 Hz, 1H), 5.51 (d, *J* = 3.7 Hz, 1H), 5.34 – 5.05 (m, 4H), 4.95 (d, *J* = 3.9 Hz, 1H), 4.59 (s, 1H), 4.55 – 4.40 (m, 3H), 4.20 (q, *J* = 6.4 Hz, 1H), 4.13 (dq, *J* = 13.7, 6.9 Hz, 2H), 3.95 – 3.84 (m, 1H), 3.75 (dd, *J* = 11.2, 6.2 Hz, 2H), 3.58 (s, 1H), 3.26 (dd, *J* = 19.1, 1.9 Hz, 1H), 2.98 (d, *J* = 19.0 Hz, 1H), 2.63 (s, 1H), 2.47 (dddd, *J* = 14.6, 12.0, 9.6, 5.4 Hz, 3H), 2.41 (s, 3H), 2.32 (d, *J* = 15.2 Hz, 1H), 2.22 – 2.01 (m, 4H), 2.01 – 1.85 (m, 2H), 1.78 (td, *J* = 12.9, 4.0 Hz, 1H), 1.41 – 1.21 (m, 9H). ^{13}C NMR (126 MHz, $CDCl_3$) δ 212.2, 209.9, 187.0, 186.8, 156.8, 156.6, 155.7, 136.1, 134.6, 134.6, 133.8, 133.7, 133.6, 133.0, 127.2, 127.1, 117.6, 111.6, 110.9, 101.8, 101.6, 100.7, 100.3, 82.2, 81.2, 77.4, 77.2, 76.9, 76.8, 72.0, 69.5, 68.0, 67.9, 65.6, 65.1, 53.9, 46.7, 35.1, 34.4, 33.8, 33.5, 31.9, 31.5, 30.1, 29.8, 29.4, 27.6, 25.0, 17.4, 16.9, 14.9. HRMS: [M + Na]$^+$ calculated for $C_{42}H_{49}NO_{16}$ 846.2949; found 846.2937.

7-[2,3-Dideoxy-4-ulo-α-L-fucopyranosyl-2-deoxy-α-L-fucopyranosyl-(1→4)-3-amino-2,3-dideoxy-α-L-fucopyranoside]-idarubicinone (25)

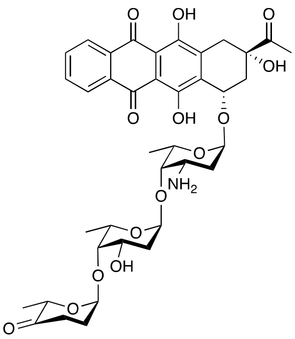

According to general procedure D, **S38** (66 mg, 0.089 mmol) was deprotected. Size-exclusion chromatography (Sephadex LH-20, 1:1 DCM:MeOH v/v) gave the crude amine. Then, silica gel column chromatography (20:80 – 80:20 acetone:DCM) gave the free amine (29.2 mg, 39.4 umol, 49%). ^{1}H NMR (500 MHz, $CDCl_3$) δ 8.32 (s, 2H), 7.90 – 7.76 (m, 2H), 5.51 (s, 1H), 5.26 (s, 1H), 5.09 (t, *J* = 6.2 Hz, 1H), 5.01 (s, 1H), 4.48 (q, *J* = 6.7 Hz, 1H), 4.23 (d, *J* = 6.7 Hz, 1H), 4.13 (d, *J* = 6.8 Hz, 2H), 3.72 (s, 1H), 3.57 (s, 1H), 3.24 (d, *J* = 18.9 Hz, 1H), 2.98 (d, *J* = 18.9 Hz, 1H), 2.57 – 2.37 (m, 6H), 2.33 (d, *J* = 14.8 Hz, 2H), 2.23 – 2.04 (m, 4H), 1.90 (td, *J* = 12.3, 3.7 Hz, 2H), 1.46 – 1.23 (m, 9H). ^{13}C NMR (126 MHz, $CDCl_3$) δ 212.0, 210.0, 187.0, 186.7, 156.8, 156.5, 136.0, 134.7, 134.6, 134.0, 133.6, 133.5, 127.2, 127.1, 111.6, 110.9, 100.9, 100.3, 82.2, 77.4, 77.2, 76.9, 76.6, 72.0, 69.3, 68.3, 67.7, 65.2, 46.9, 35.0, 34.5, 33.7, 33.6, 29.8, 27.7, 25.0, 17.6, 17.2, 14.9. HRMS: [M + H]$^+$ calculated for $C_{38}H_{45}NO_{14}$ 740.2918; found 740.2909.

7-[2,3-Dideoxy-4-ulo-α-L-fucopyranosyl-2-deoxy-α-L-fucopyranosyl-(1→4)- -2,3-dideoxy-3-dimethylamino-α-L-fucopyranoside]-idarubicinone (26)

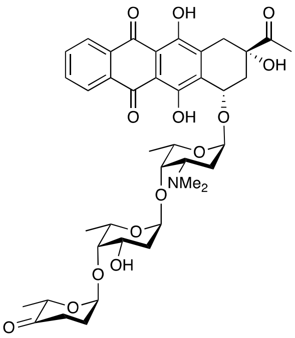

To a solution of **25** (20 mg, 27 umol) in EtOH (5.4 mL) and DCM (1.0 mL) was added 37% aq. CH_2O (0.20 mL) and the mixture was stirred for 40 minutes before addition of $NaBH(OAc)_3$ (11.2 mg, 52.8 umol, 1.95 eq). After stirring for 90 minutes, the mixture was poured into sat. aq. $NaHCO_3$ and extracted with DCM thrice. The organic layer was dried over Na_2SO_4 and concentrated *in vacuo*. Column chromatography on neutral silica gel (10:90 – 30:70 MeOH:DCM) gave the title compound as a red solid (12.4 mg, 16.1 umol, 60%). ^1H NMR (850 MHz, $CDCl_3$) δ 8.34 (dt, *J* = 5.9, 3.6 Hz, 2H), 7.91 – 7.78 (m, 2H), 5.61 – 5.50 (m, 1H), 5.28 (dd, *J* = 4.0, 2.1 Hz, 1H), 5.16 – 5.04 (m, 2H), 4.50 (dq, *J* = 10.3, 6.7 Hz, 2H), 4.17 – 4.10 (m, 1H), 4.02 (q, *J* = 6.5 Hz, 1H), 3.84 (d, *J* = 14.0 Hz, 1H), 3.70 (d, *J* = 2.8 Hz, 1H), 3.25 (dd, *J* = 18.7, 2.1 Hz, 1H), 3.00 (d, *J* = 18.6 Hz, 1H), 2.62 (s, 2H), 2.52 – 2.43 (m, 2H), 2.42 (s, 3H), 2.38 – 2.30 (m, 6H), 2.19 – 2.12 (m, 1H), 2.12 – 2.08 (m, 2H), 2.05 – 1.99 (m, 1H), 1.92 – 1.88 (m, 1H), 1.85 (td, *J* = 12.2, 3.7 Hz, 1H), 1.32 (d, *J* = 6.8 Hz, 3H), 1.30 (dd, *J* = 6.7, 3.2 Hz, 3H), 1.18 (d, *J* = 6.5 Hz, 3H). ^{13}C NMR (214 MHz, $CDCl_3$) δ 211.8, 210.3, 187.0, 186.8, 173.5, 156.8, 156.6, 136.0, 134.7, 134.7, 134.0, 133.7, 133.5, 127.2, 127.1, 111.6, 110.9, 100.9, 100.2, 99.4, 82.6, 73.8, 73.7, 71.9, 69.3, 68.6, 67.2, 65.3, 61.8, 43.0, 35.0, 34.3, 33.7, 33.7, 32.1, 29.8, 28.7, 27.8, 25.5, 24.9, 18.2, 17.1, 14.9. HRMS: $[M + H]^+$ calculated for $C_{40}H_{49}NO_{14}$ 768.3231; found 768.3221.

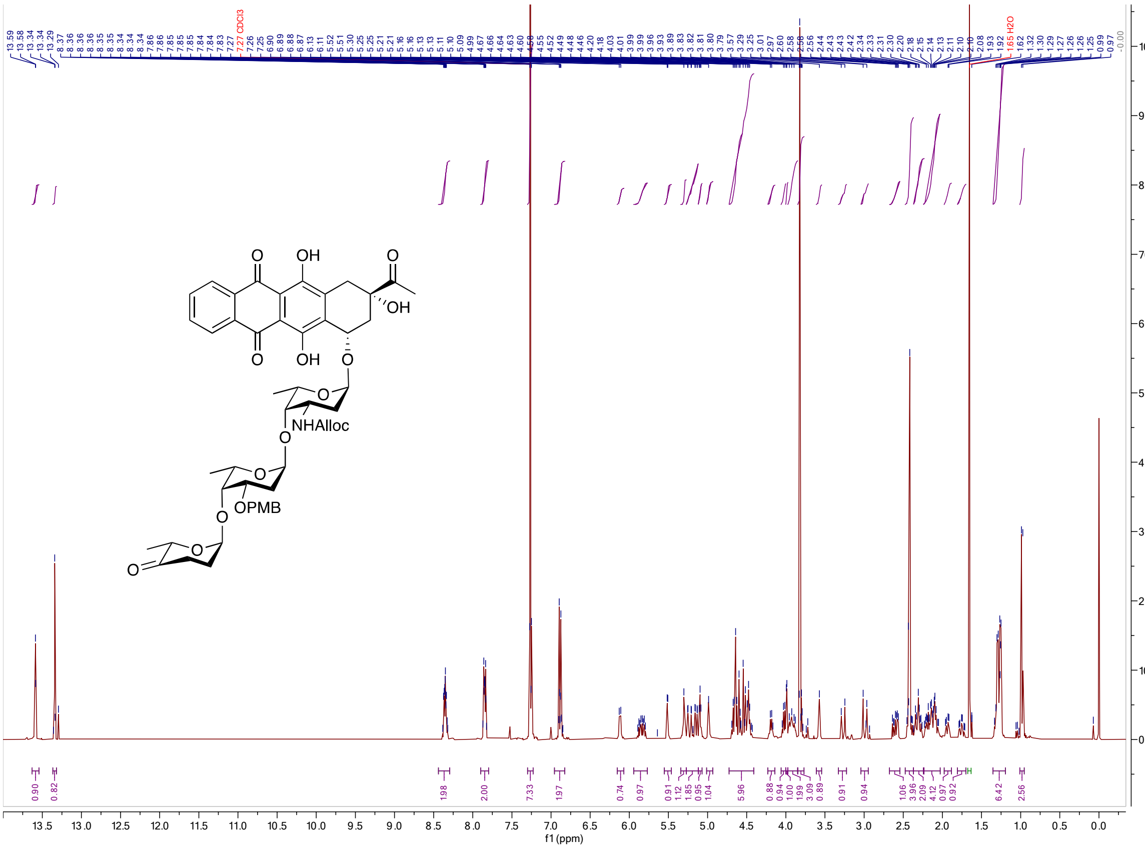
7.27 CDCl3
1.65 H2O
O
OH
O
OH
O
OH
O
OH
O
O
NHAlloc
O
O
OPMB
O
O
O
f1 (ppm)

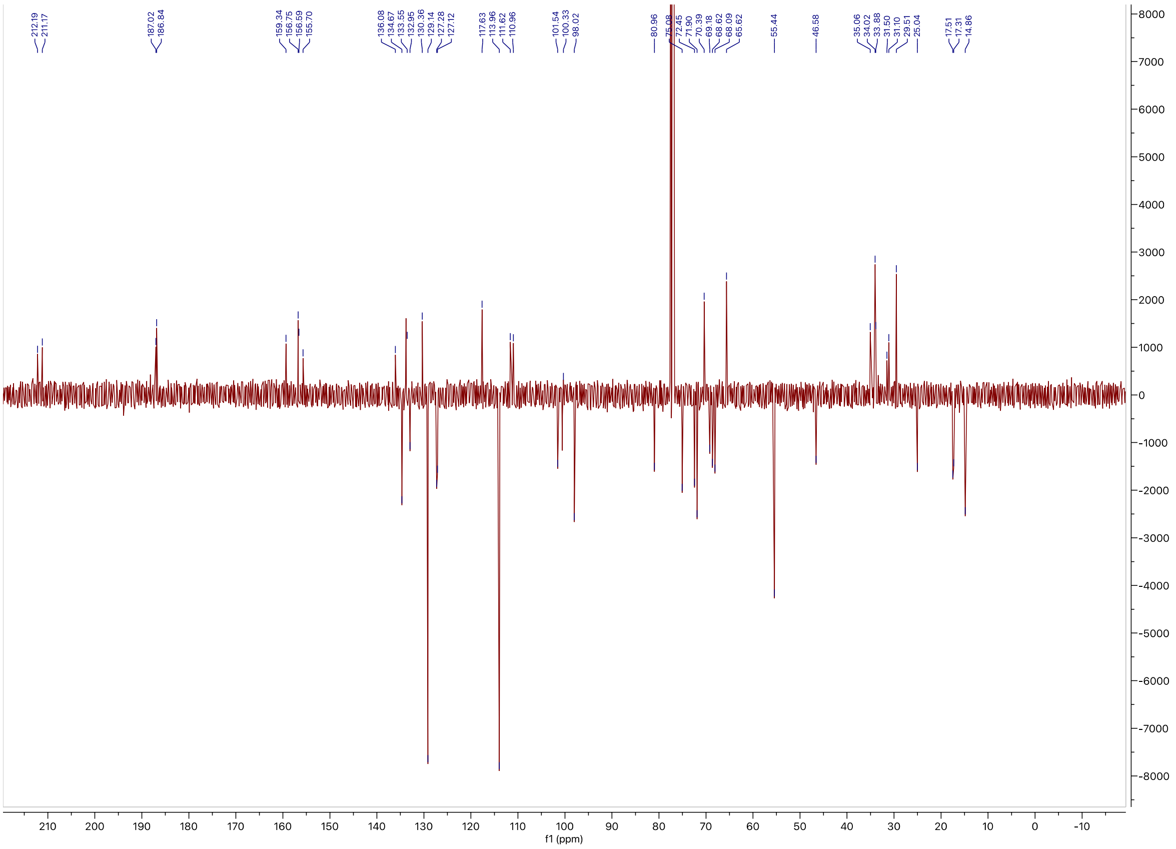
212.19
211.17
187.02
186.84
159.34
156.75
156.59
155.70
136.08
134.67
133.55
132.95
130.36
129.14
127.28
127.12
117.63
113.96
111.62
110.96
101.54
100.33
98.02
80.96
72.45
71.90
70.39
69.18
68.62
68.09
65.62
55.44
46.58
35.06
34.02
33.88
31.50
31.10
29.51
25.04
17.51
17.31
14.86
f1 (ppm)

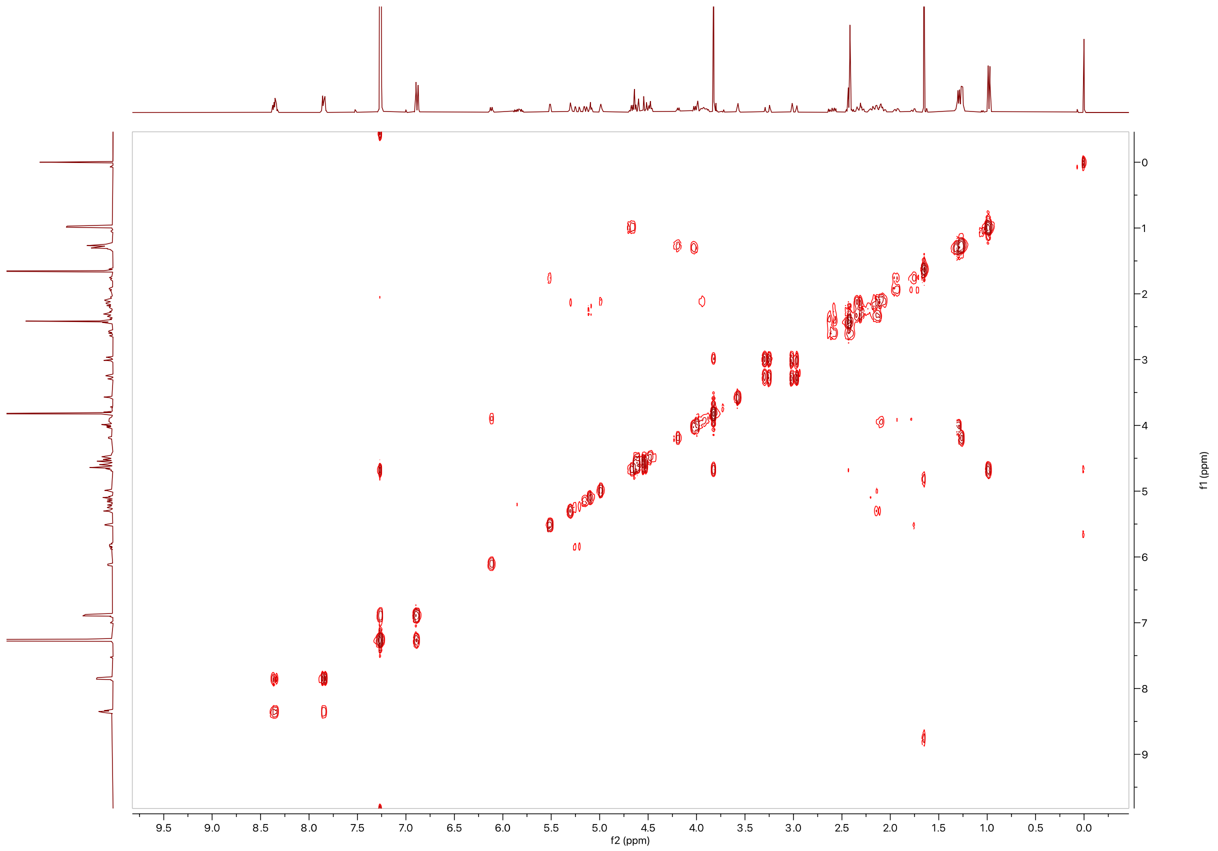
f2 (ppm)
f1 (ppm)

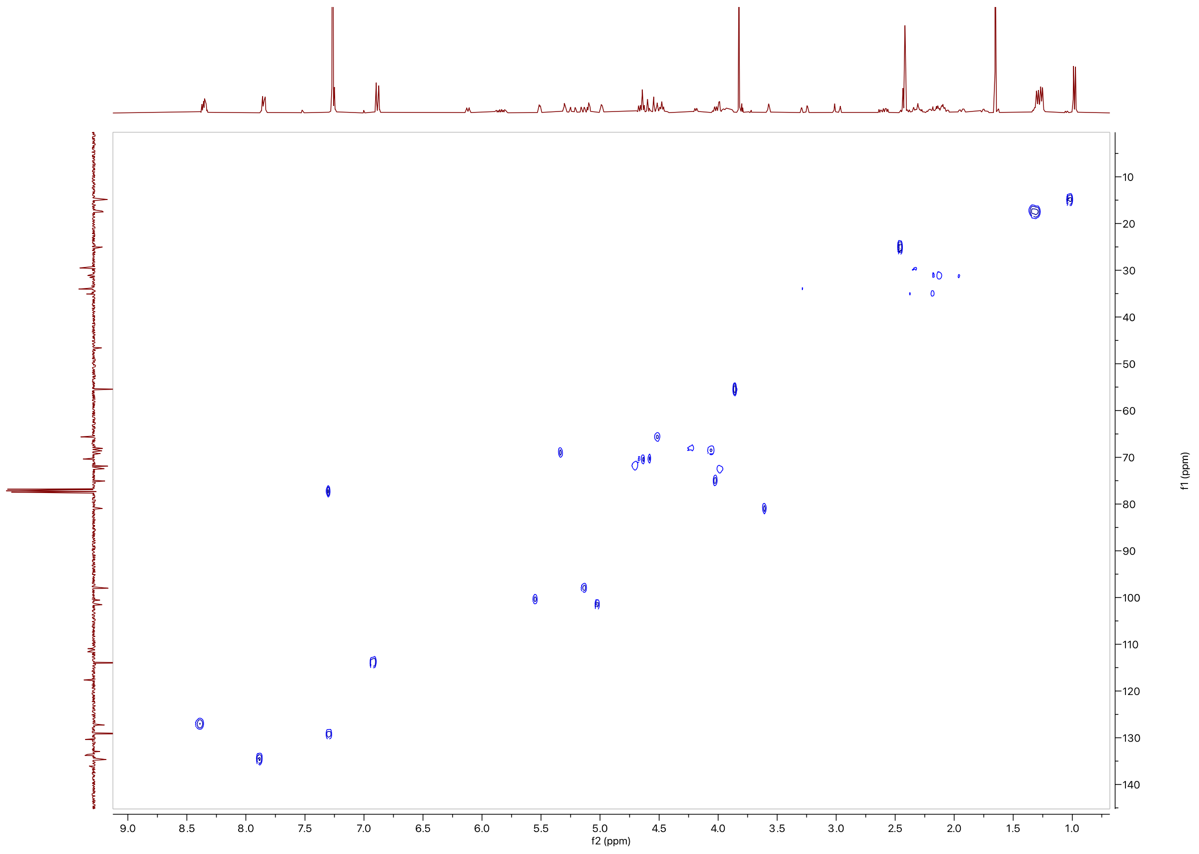
f2 (ppm)
f1 (ppm)

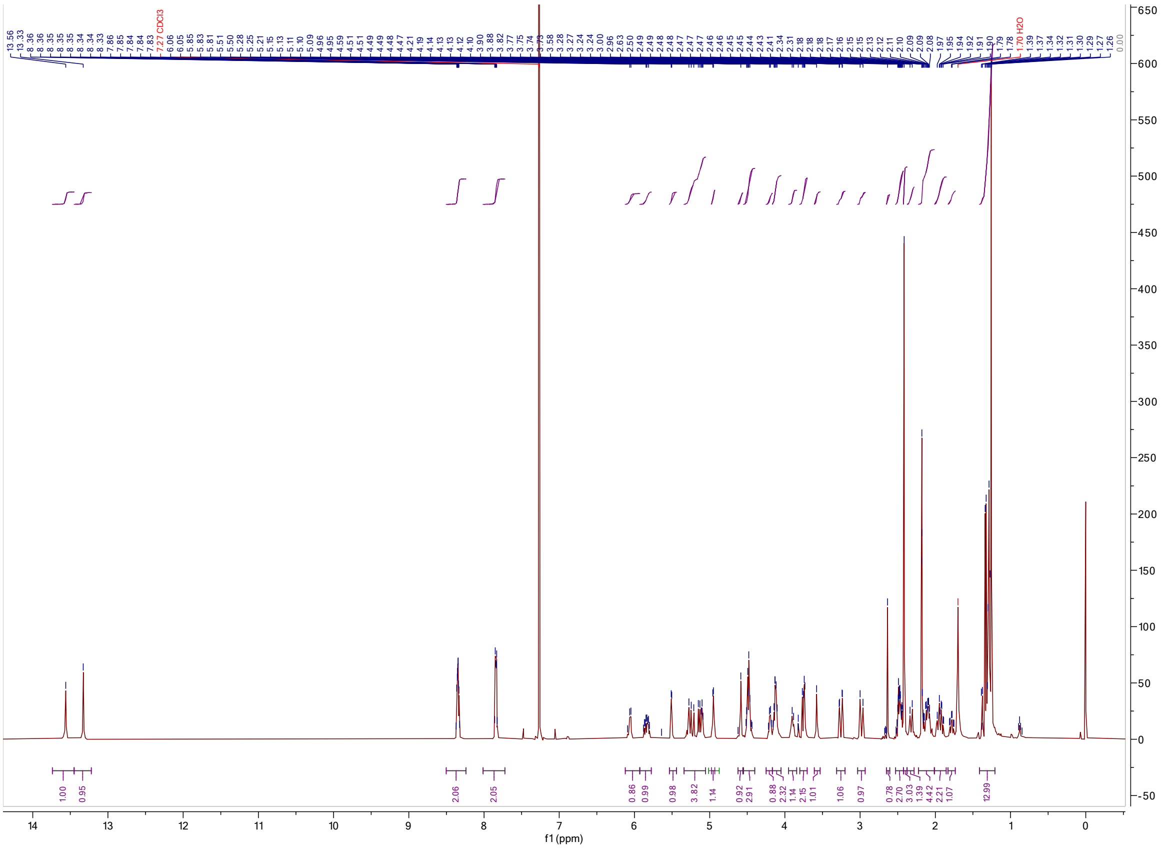

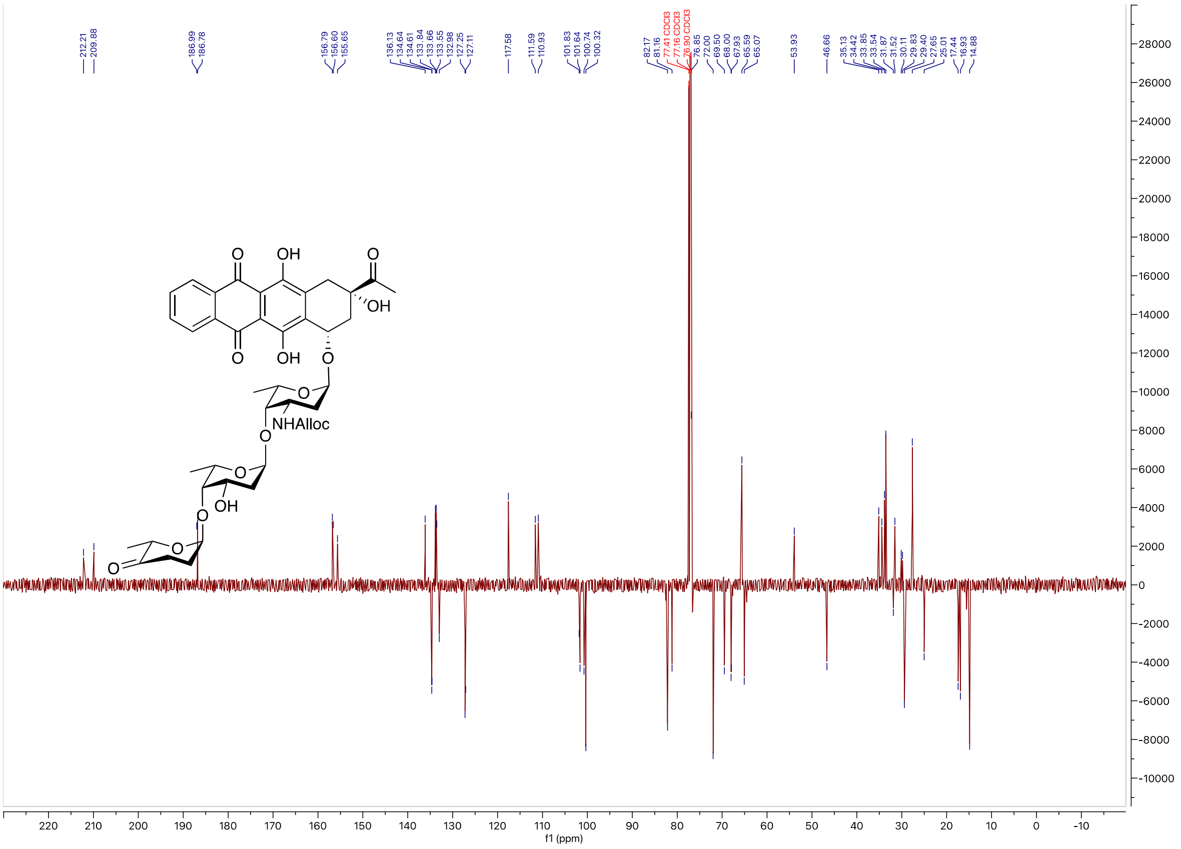
OH
O
OH
O
OH
O
O
NHAlloc
O
OH
O
O

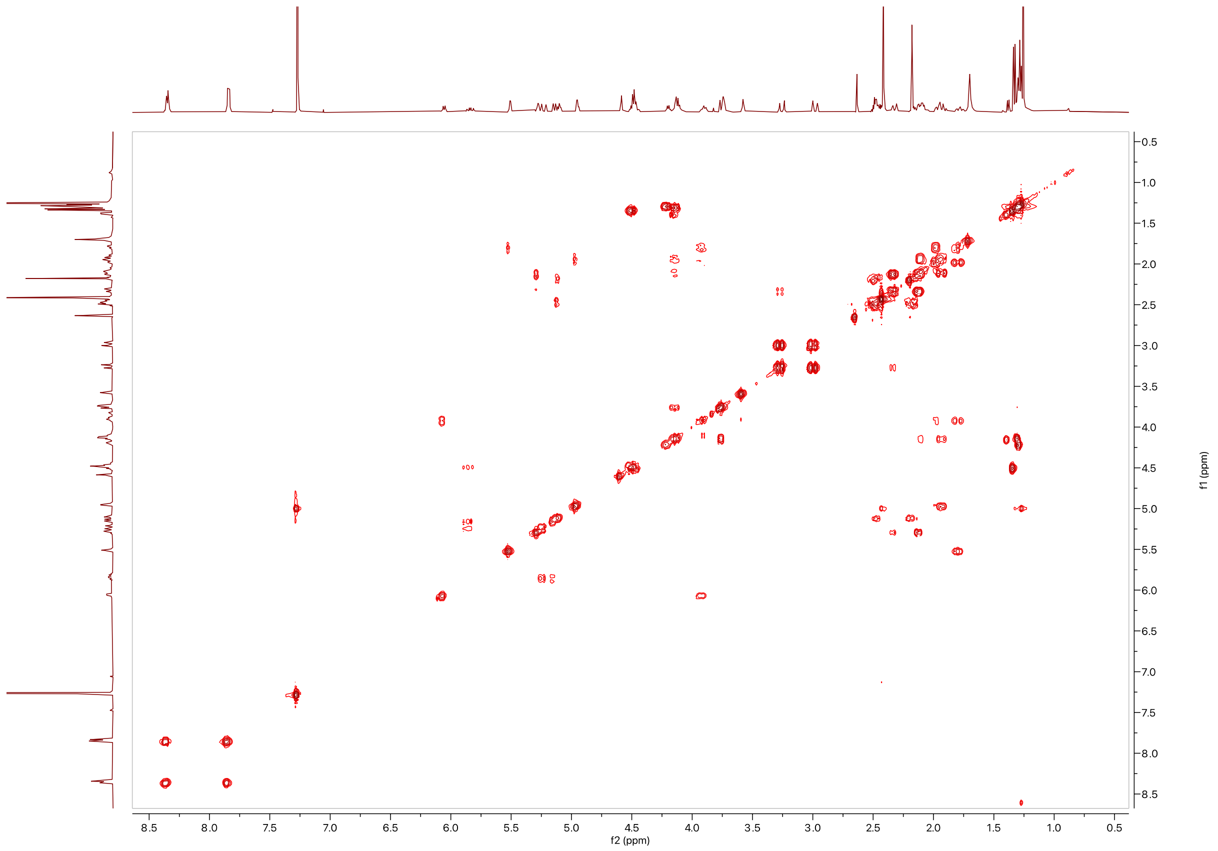
8.5
8.0
7.5
7.0
6.5
6.0
5.5
5.0
4.5
4.0
3.5
3.0
2.5
2.0
1.5
1.0
0.5
f2 (ppm)
f1 (ppm)

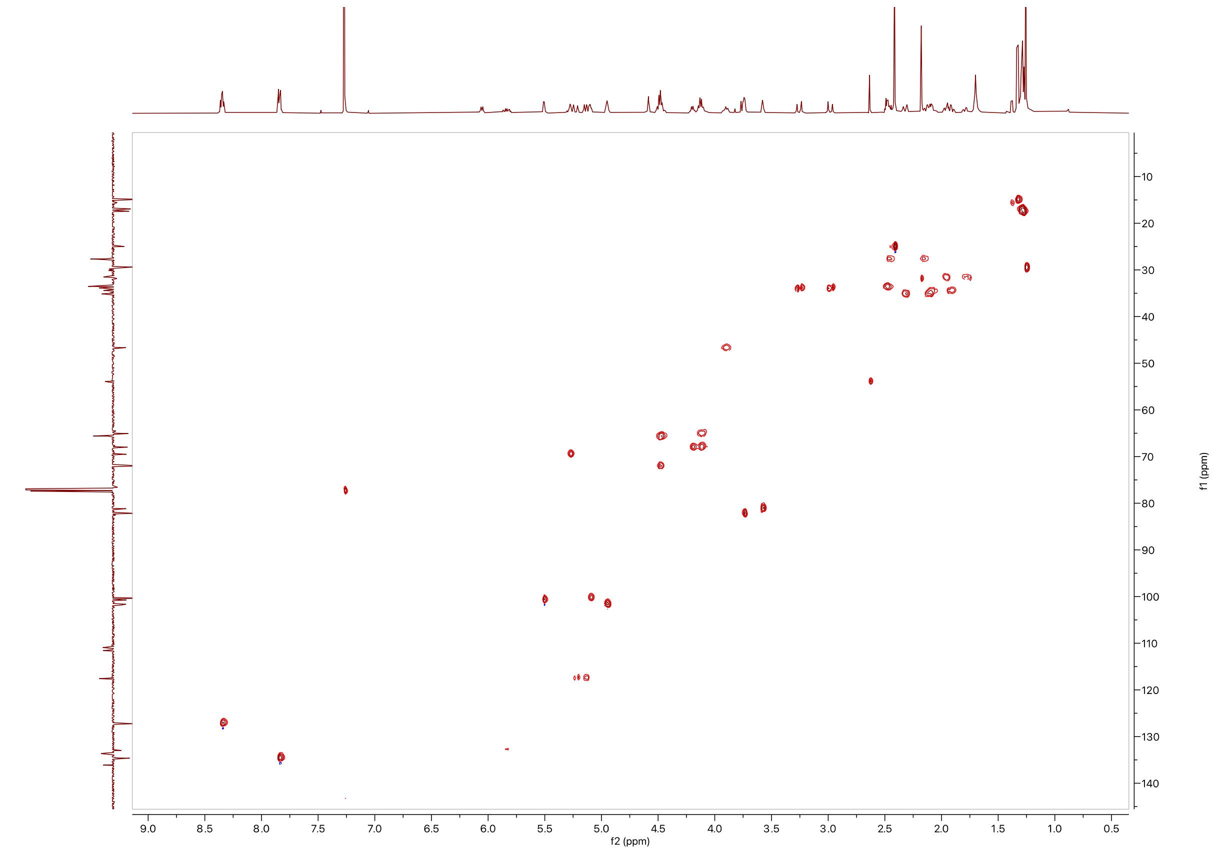
9.0
8.5
8.0
7.5
7.0
6.5
6.0
5.5
5.0
4.5
4.0
3.5
3.0
2.5
2.0
1.5
1.0
0.5
f2 (ppm)
f1 (ppm)

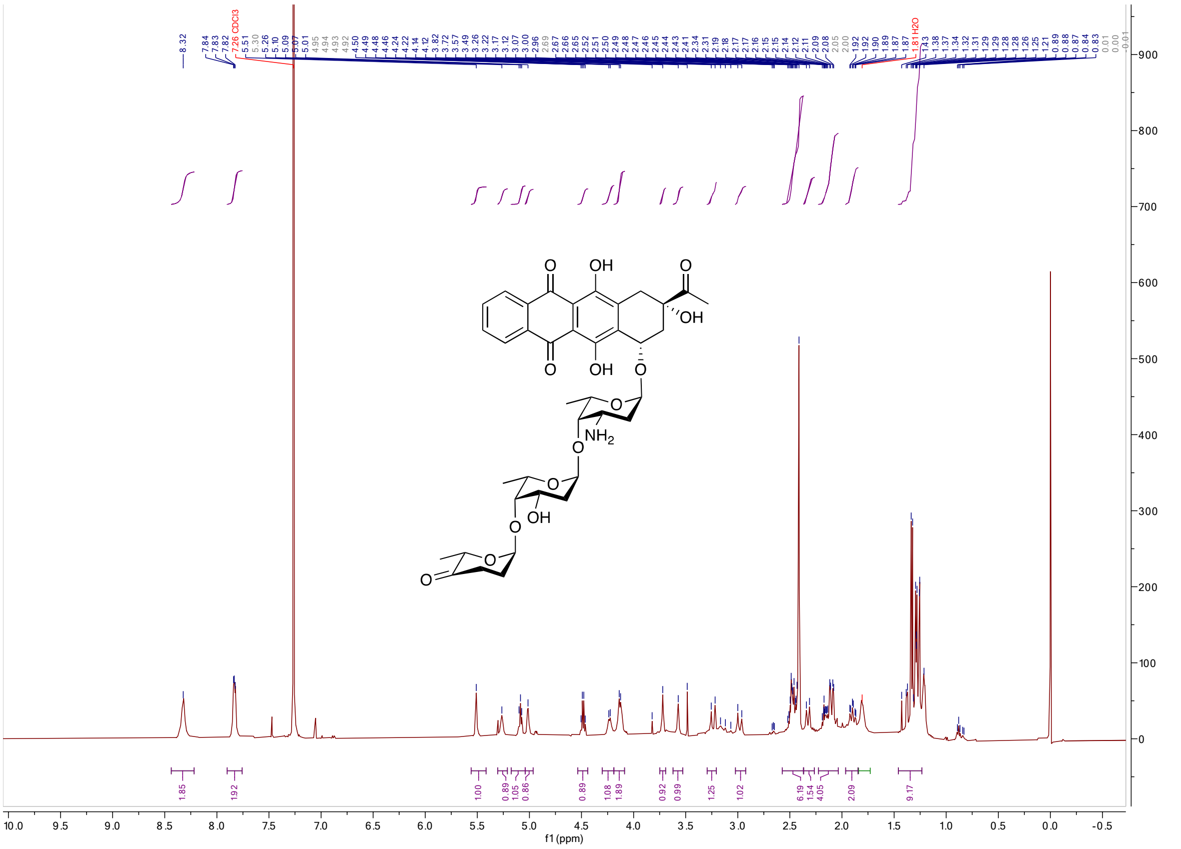
f1 (ppm)

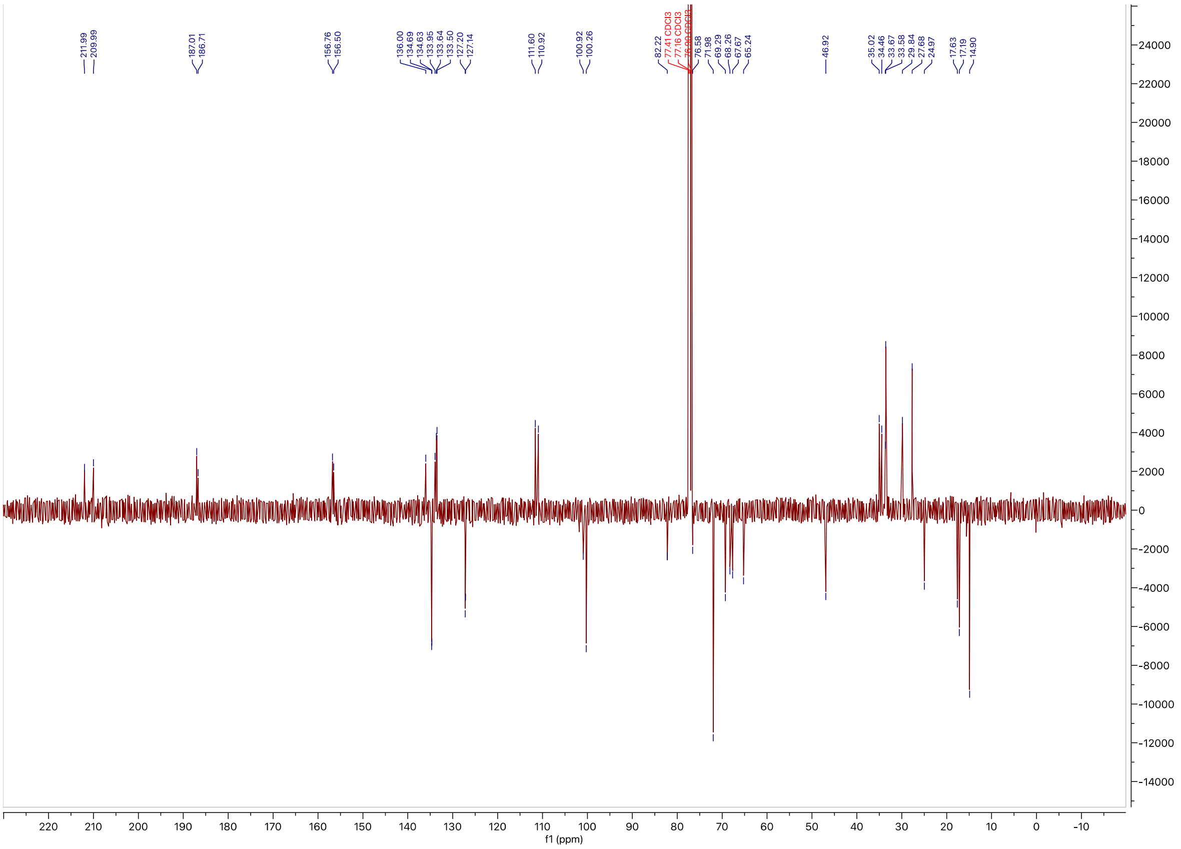
f1 (ppm)

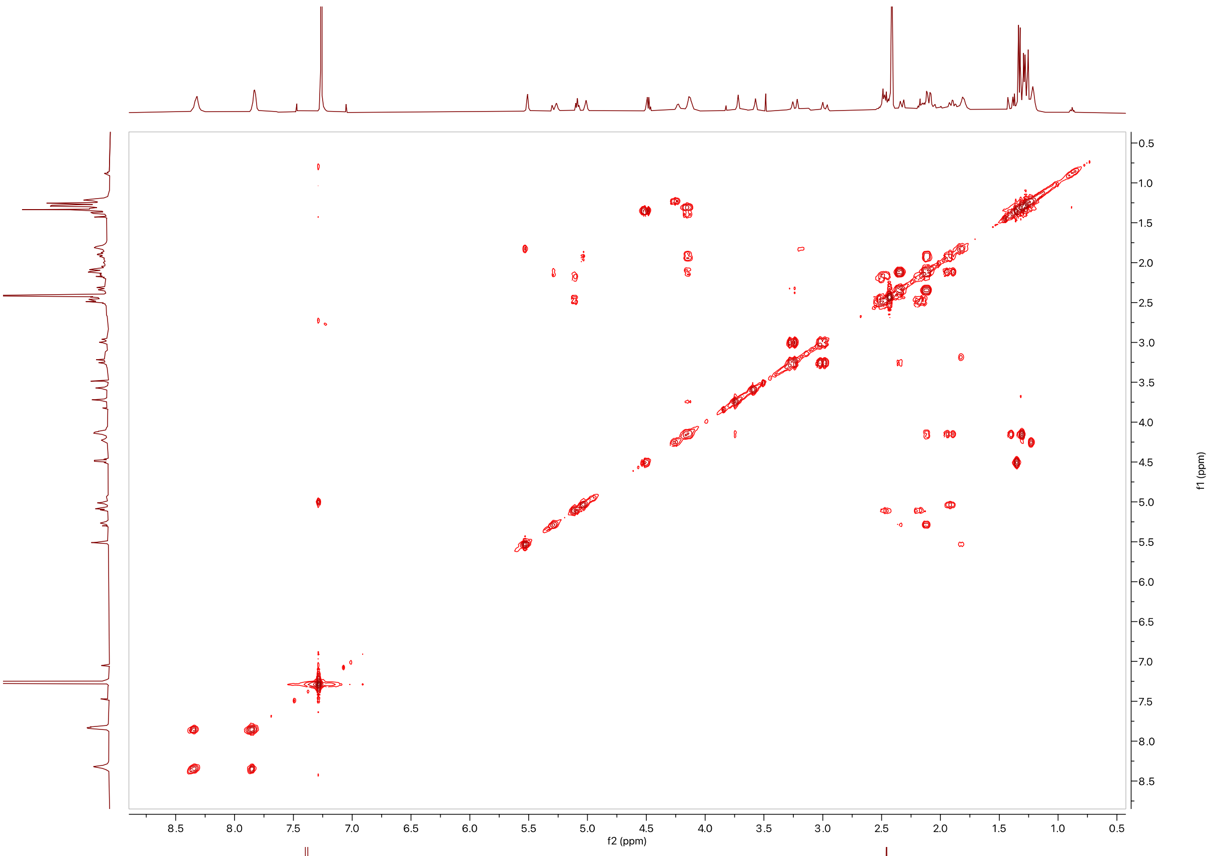
f2 (ppm)
f1 (ppm)

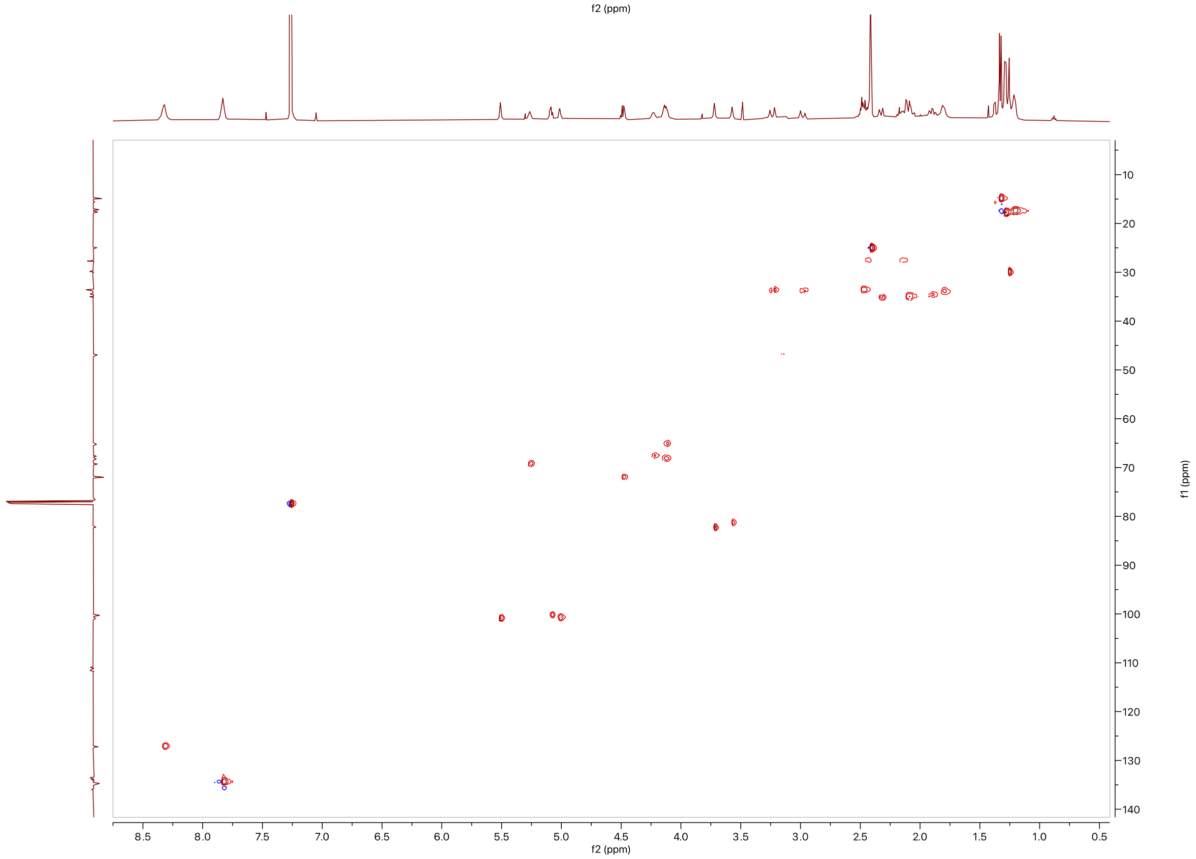
f2 (ppm)
f1 (ppm)

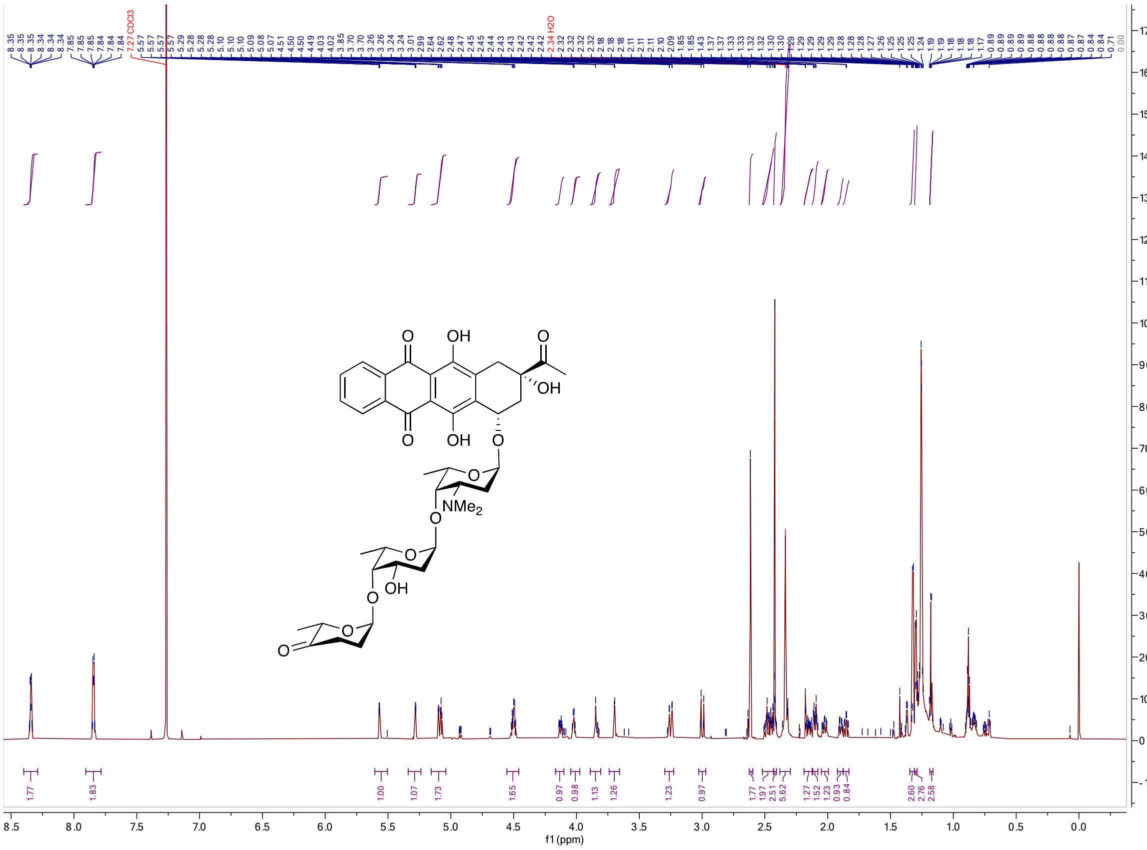
7.27 CDCl3
2.34 H2O
O
OH
OH
O
OH
O
O
NMe2
O
OH
O
O
O
1.77
1.83
1.00
1.07
1.73
1.65
0.97
0.98
1.13
1.26
1.23
0.97
1.77
1.97
2.51
5.62
1.27
1.52
1.23
0.93
0.84
2.60
2.76
2.58
f1 (ppm)

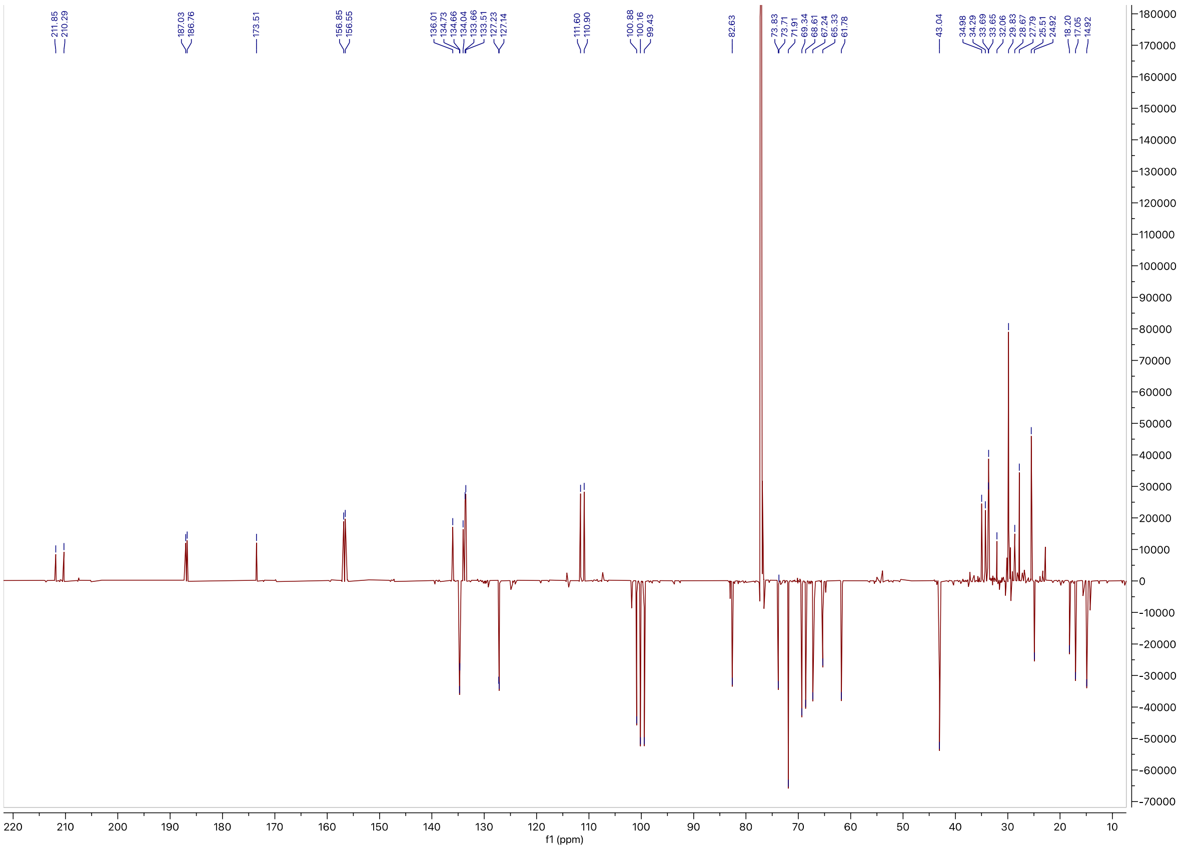
211.85
210.29
187.03
186.76
173.51
156.85
156.55
136.01
134.73
134.66
134.04
133.66
133.51
127.23
127.14
111.60
110.90
100.88
100.16
99.43
82.63
73.83
72.71
71.91
69.34
68.61
67.24
65.33
61.78
43.04
34.98
34.29
33.69
33.65
32.06
29.83
28.67
27.79
25.51
24.92
18.20
17.05
14.92
f1 (ppm)

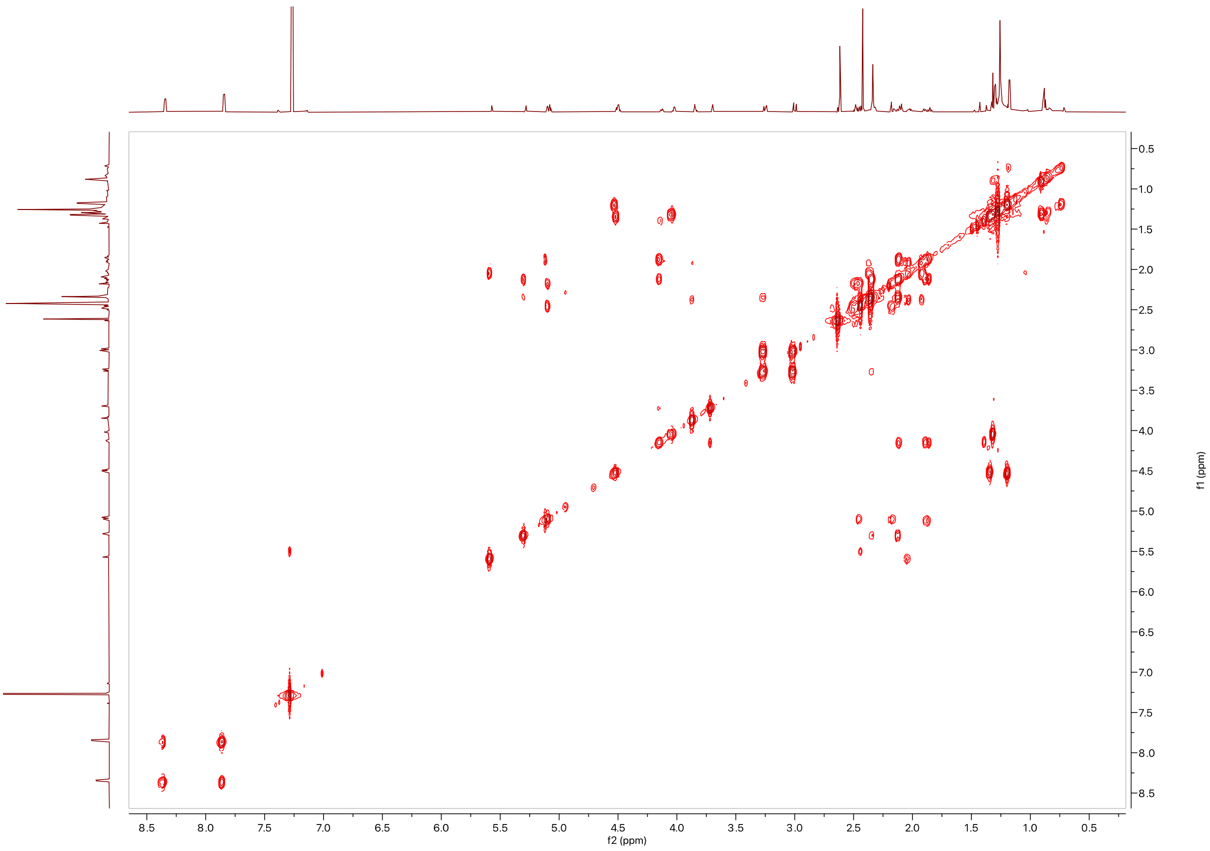
f2 (ppm)
f1 (ppm)

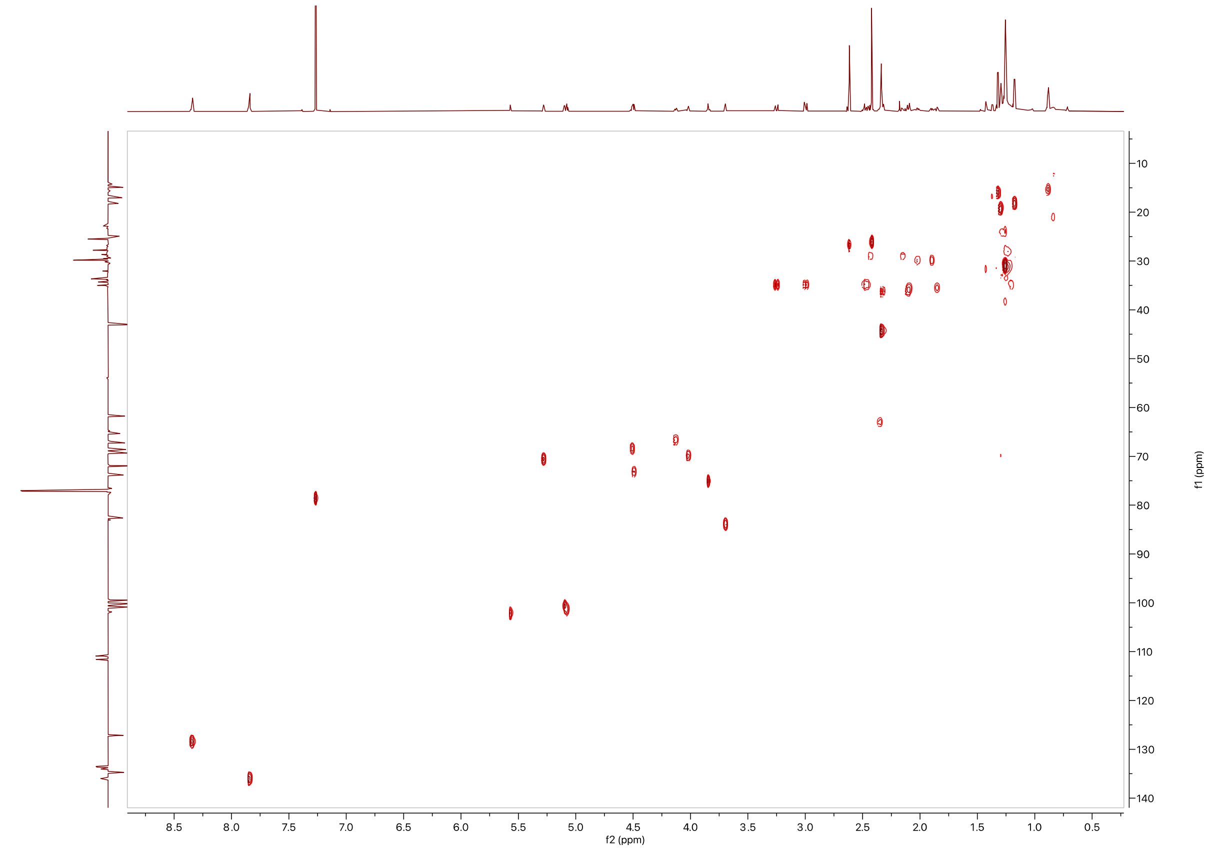
f2 (ppm)
f1 (ppm)

E: HRMS traces of compounds 6-26

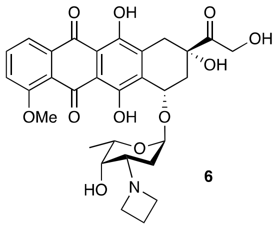

6

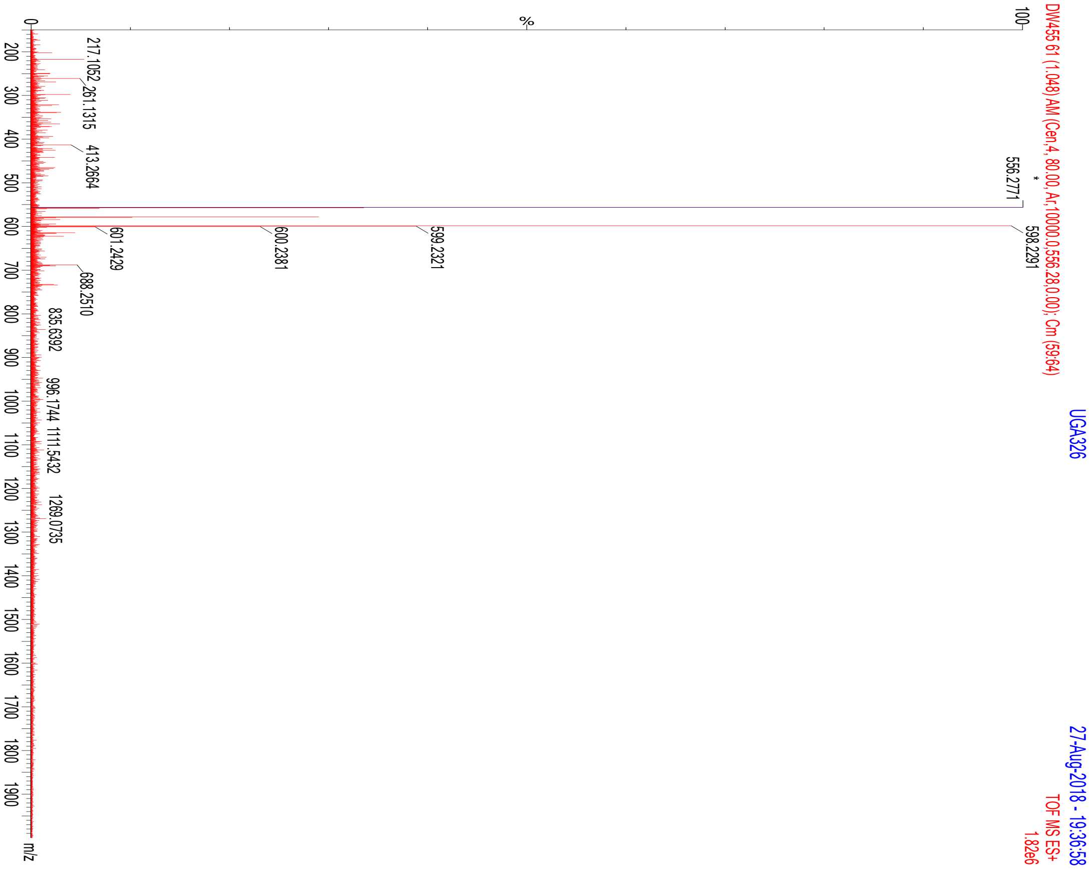

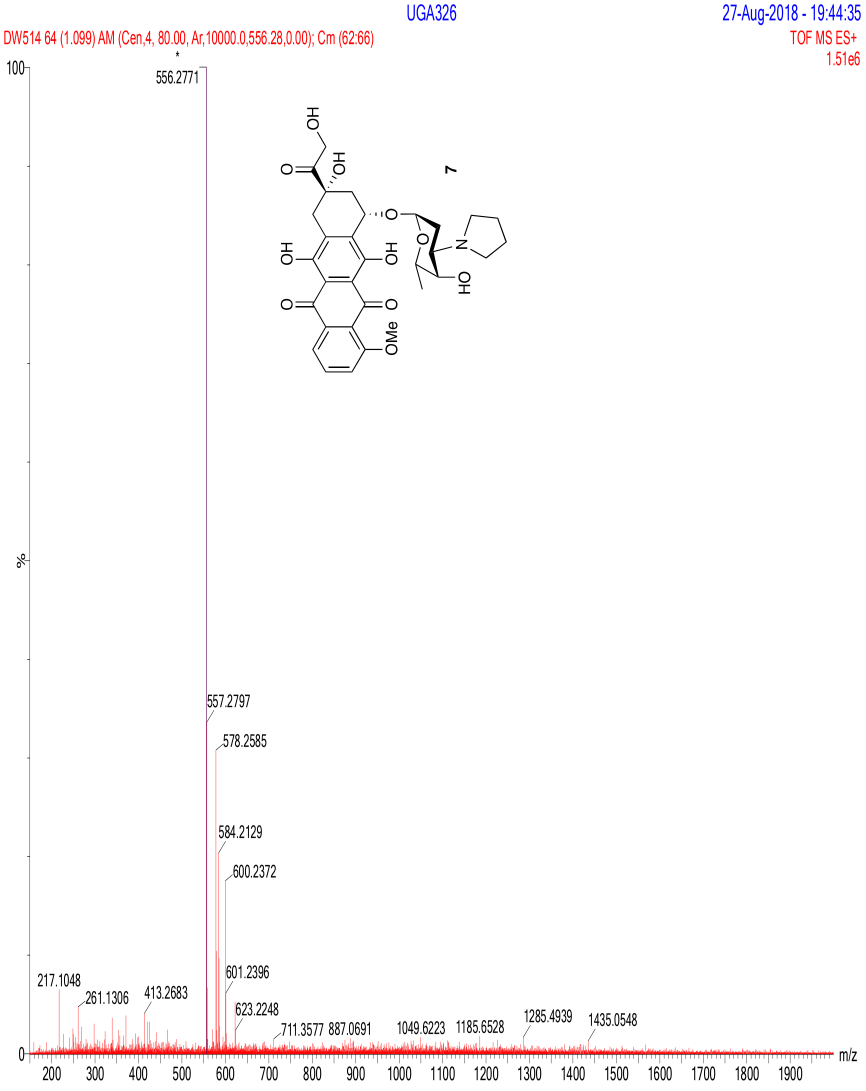
UGA326
27-Aug-2018 - 19:44:35
DW514 64 (1.099) AM (Cen,4, 80.00, Ar,10000.0,556.28,0.00); Cm (62:66)
TOF MS ES+
1.51e6
100
%
0
556.2771
557.2797
578.2585
584.2129
600.2372
601.2396
623.2248
217.1048
261.1306
413.2683
711.3577
887.0691
1049.6223
1185.6528
1285.4939
1435.0548
200
300
400
500
600
700
800
900
1000
1100
1200
1300
1400
1500
1600
1700
1800
1900
m/z
OH
O
OH
OH
O
OH
O
O
OMe
O
N
HO
7

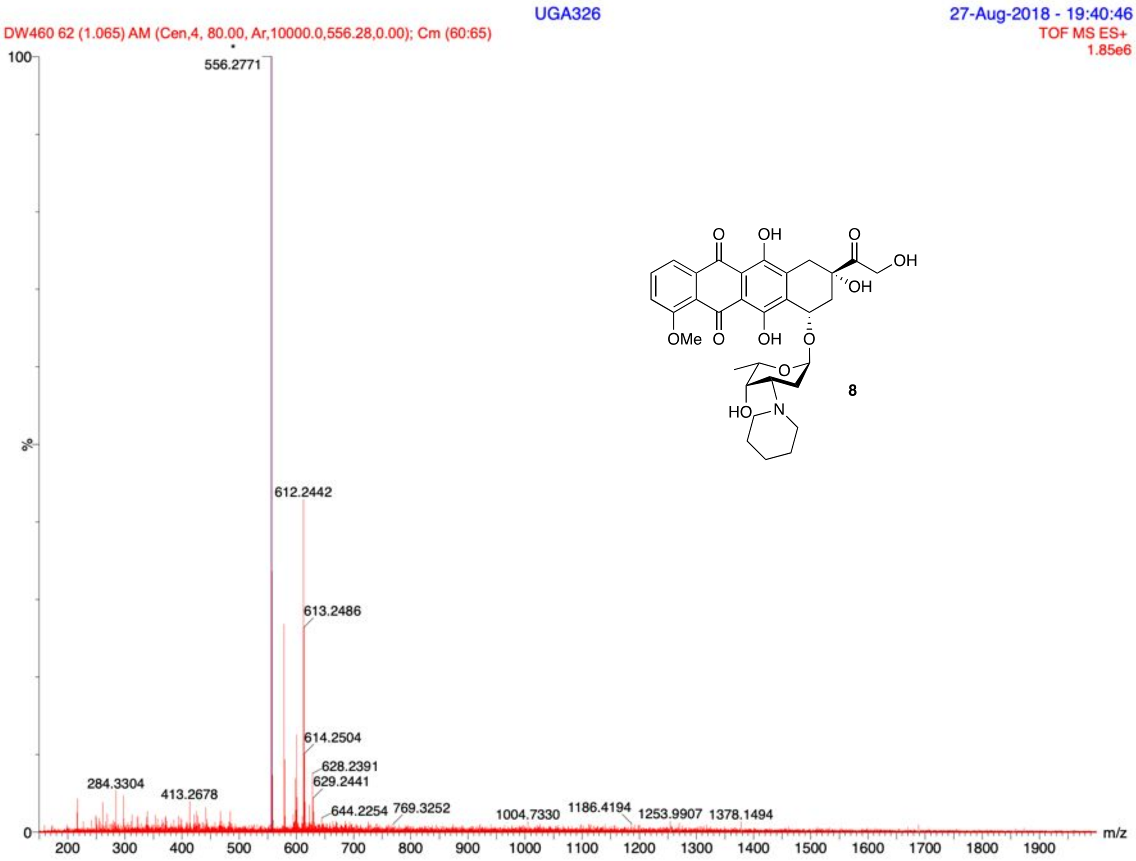
UGA326
27-Aug-2018 - 19:40:46
DW460 62 (1.065) AM (Cen,4, 80.00, Ar,10000.0,556.28,0.00); Cm (60:65)
TOF MS ES+
1.85e6
556.2771
612.2442
613.2486
614.2504
628.2391
629.2441
284.3304
413.2678
644.2254
769.3252
1004.7330
1186.4194
1253.9907
1378.1494
%
m/z
OMe
OH
HO
N
8

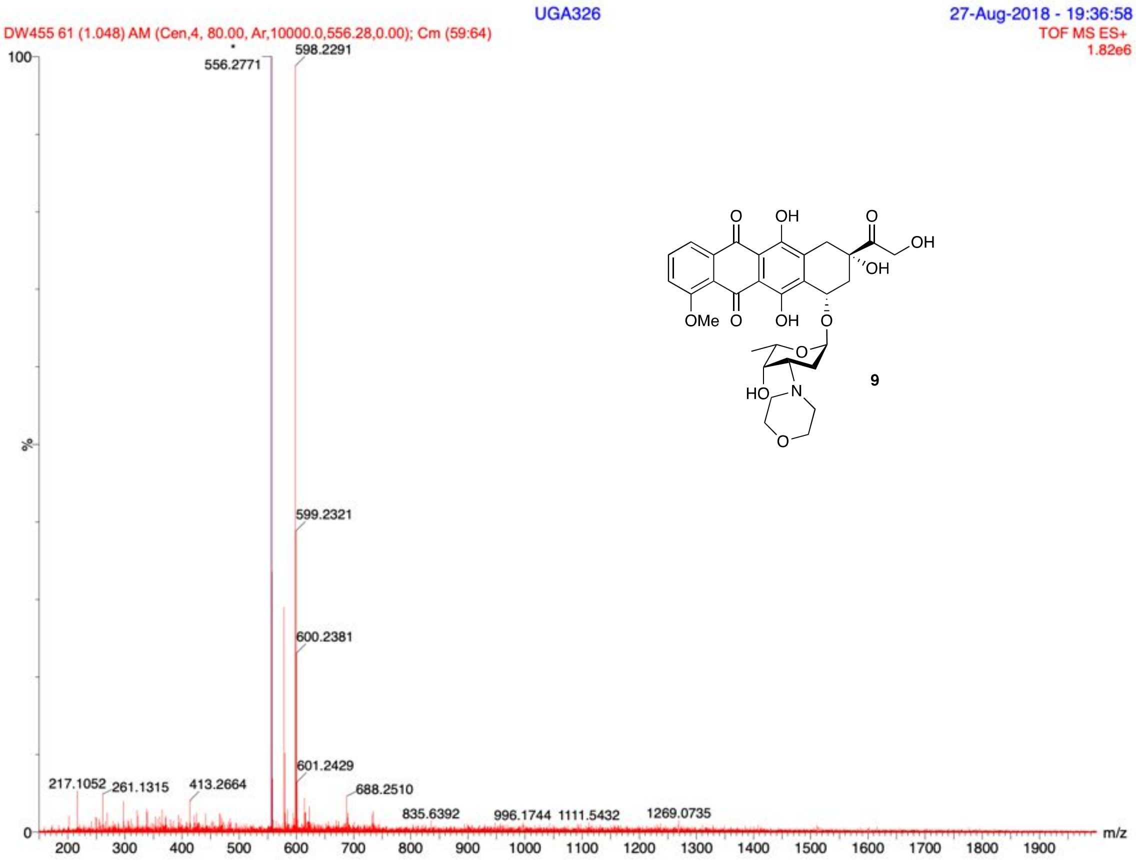
UGA326
27-Aug-2018 - 19:36:58
DW455 61 (1.048) AM (Cen,4, 80.00, Ar,10000.0,556.28,0.00); Cm (59:64)
TOF MS ES+
1.82e6
556.2771
598.2291
599.2321
600.2381
601.2429
688.2510
217.1052
261.1315
413.2664
835.6392
996.1744
1111.5432
1269.0735
%
m/z

9

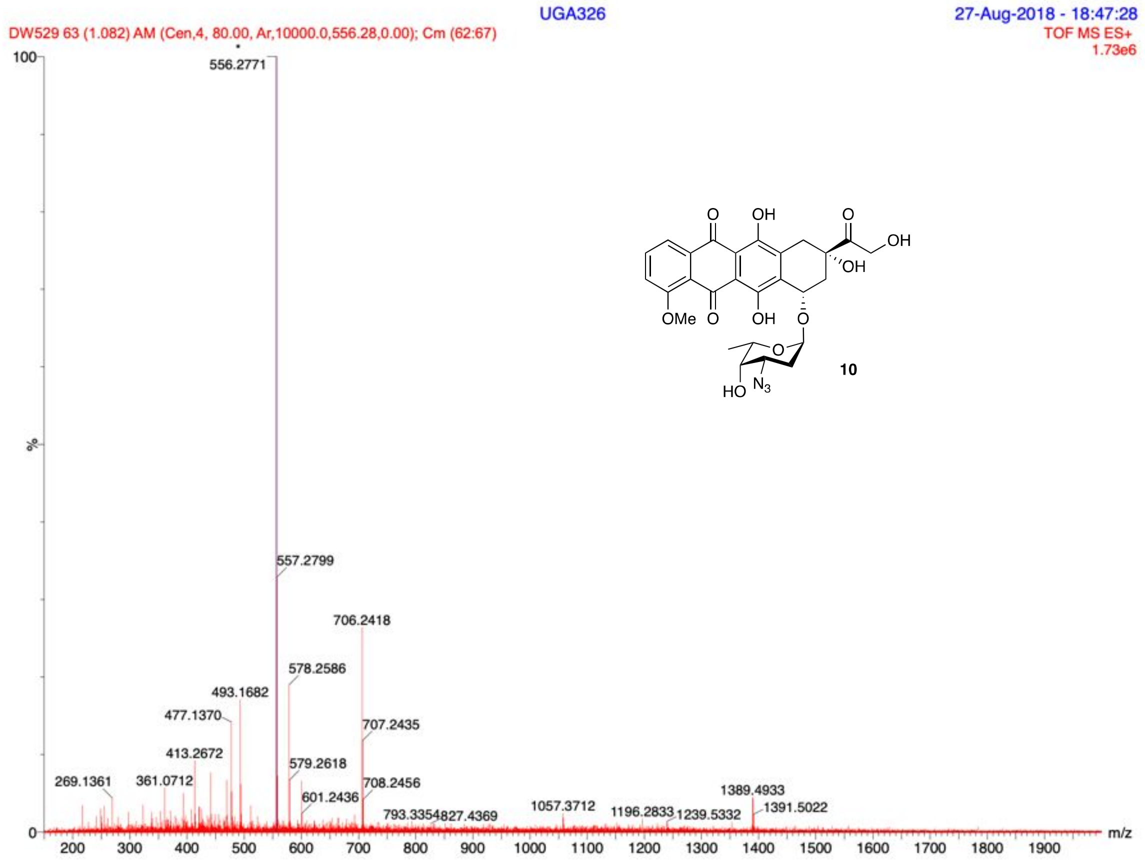
UGA326
27-Aug-2018 - 18:47:28
DW529 63 (1.082) AM (Cen,4, 80.00, Ar,10000.0,556.28,0.00); Cm (62:67)
TOF MS ES+
1.73e6
100
%
0
556.2771
557.2799
706.2418
578.2586
493.1682
477.1370
707.2435
413.2672
579.2618
269.1361
361.0712
708.2456
601.2436
793.3354
827.4369
1057.3712
1196.2833
1239.5332
1389.4933
1391.5022
m/z
200
300
400
500
600
700
800
900
1000
1100
1200
1300
1400
1500
1600
1700
1800
1900
O
OH
OH
OMe
O
OH
O
OH
O
O
HO
N3
10

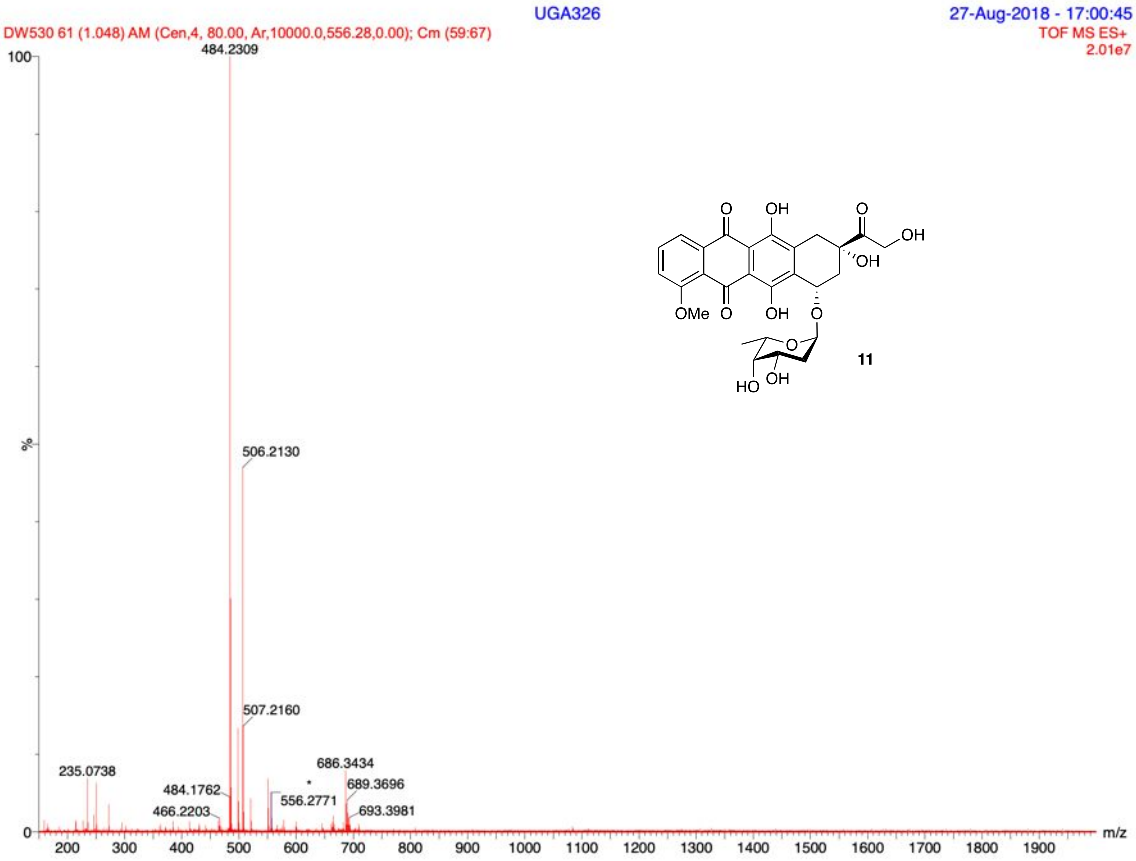
UGA326
27-Aug-2018 - 17:00:45
DW530 61 (1.048) AM (Cen,4, 80.00, Ar,10000.0,556.28,0.00); Cm (59:67)
TOF MS ES+
2.01e7
484.2309
506.2130
507.2160
235.0738
484.1762
466.2203
686.3434
689.3696
556.2771
693.3981
%
m/z
OMe
OH
HO
11

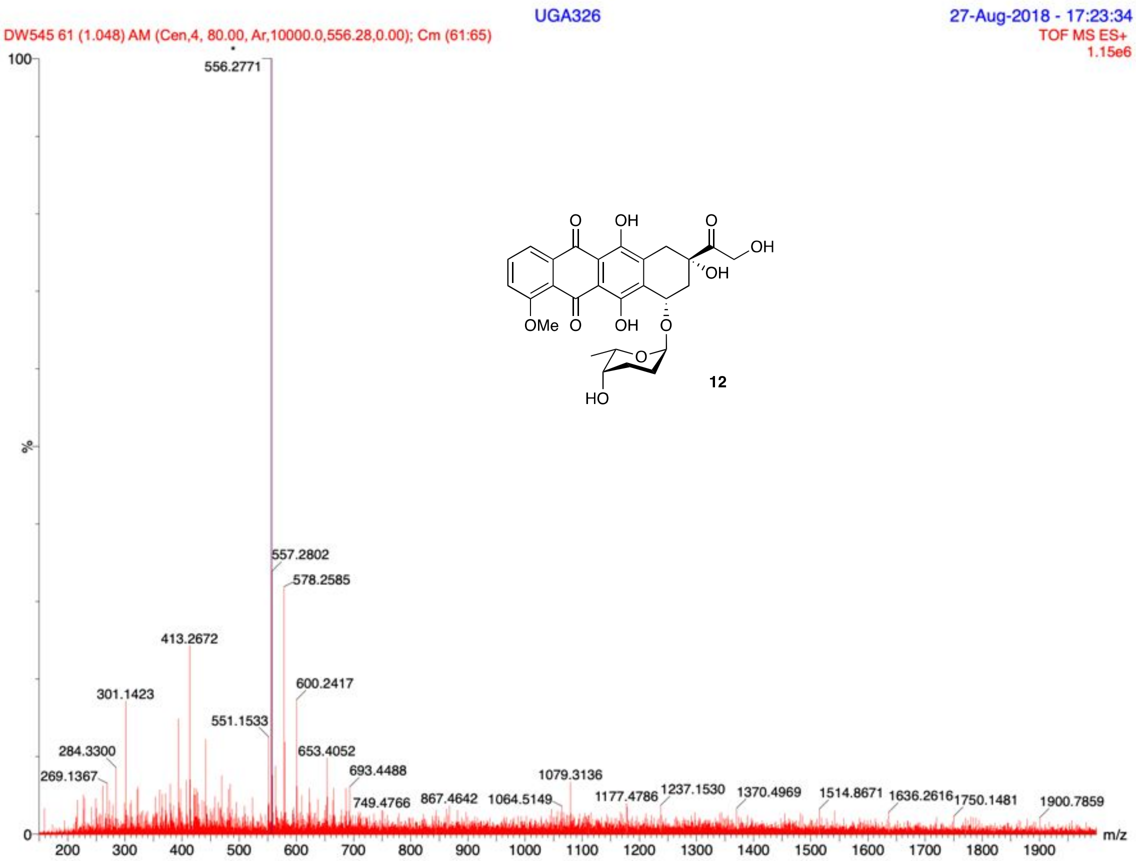

UGA326
DW545 61 (1.048) AM (Cen,4, 80.00, Ar,10000.0,556.28,0.00); Cm (61:65)
27-Aug-2018 - 17:23:34
TOF MS ES+
1.15e6
100
%
0
556.2771
557.2802
578.2585
413.2672
600.2417
301.1423
551.1533
284.3300
653.4052
269.1367
693.4488
749.4766
867.4642
1064.5149
1079.3136
1177.4786
1237.1530
1370.4969
1514.8671
1636.2616
1750.1481
1900.7859
m/z
200
300
400
500
600
700
800
900
1000
1100
1200
1300
1400
1500
1600
1700
1800
1900
OMe
OH
O
HO
12

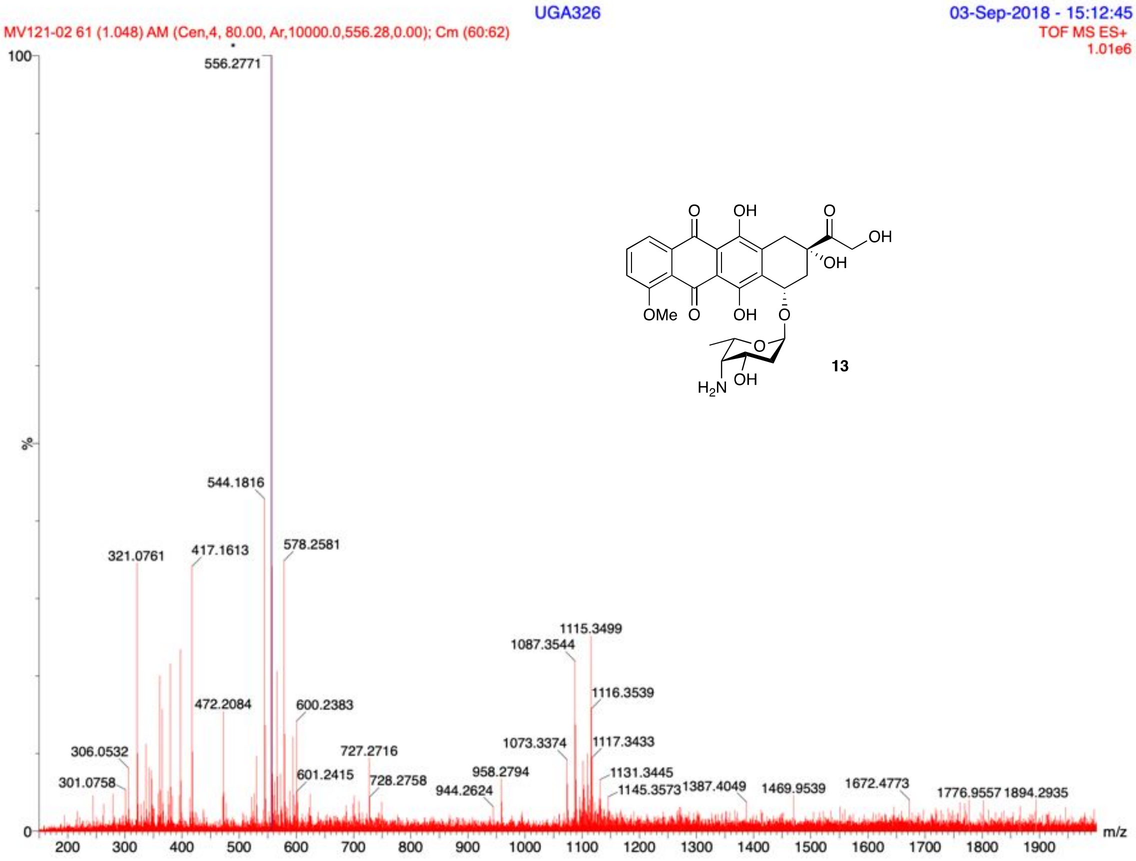
UGA326
MV121-02 61 (1.048) AM (Cen,4, 80.00, Ar,10000.0,556.28,0.00); Cm (60:62)
03-Sep-2018 - 15:12:45
TOF MS ES+
1.01e6
556.2771
544.1816
578.2581
321.0761
417.1613
1115.3499
1087.3544
1116.3539
472.2084
600.2383
1073.3374
1117.3433
306.0532
727.2716
958.2794
1131.3445
301.0758
601.2415
728.2758
944.2624
1145.3573
1387.4049
1469.9539
1672.4773
1776.9557
1894.2935
13
m/z
%

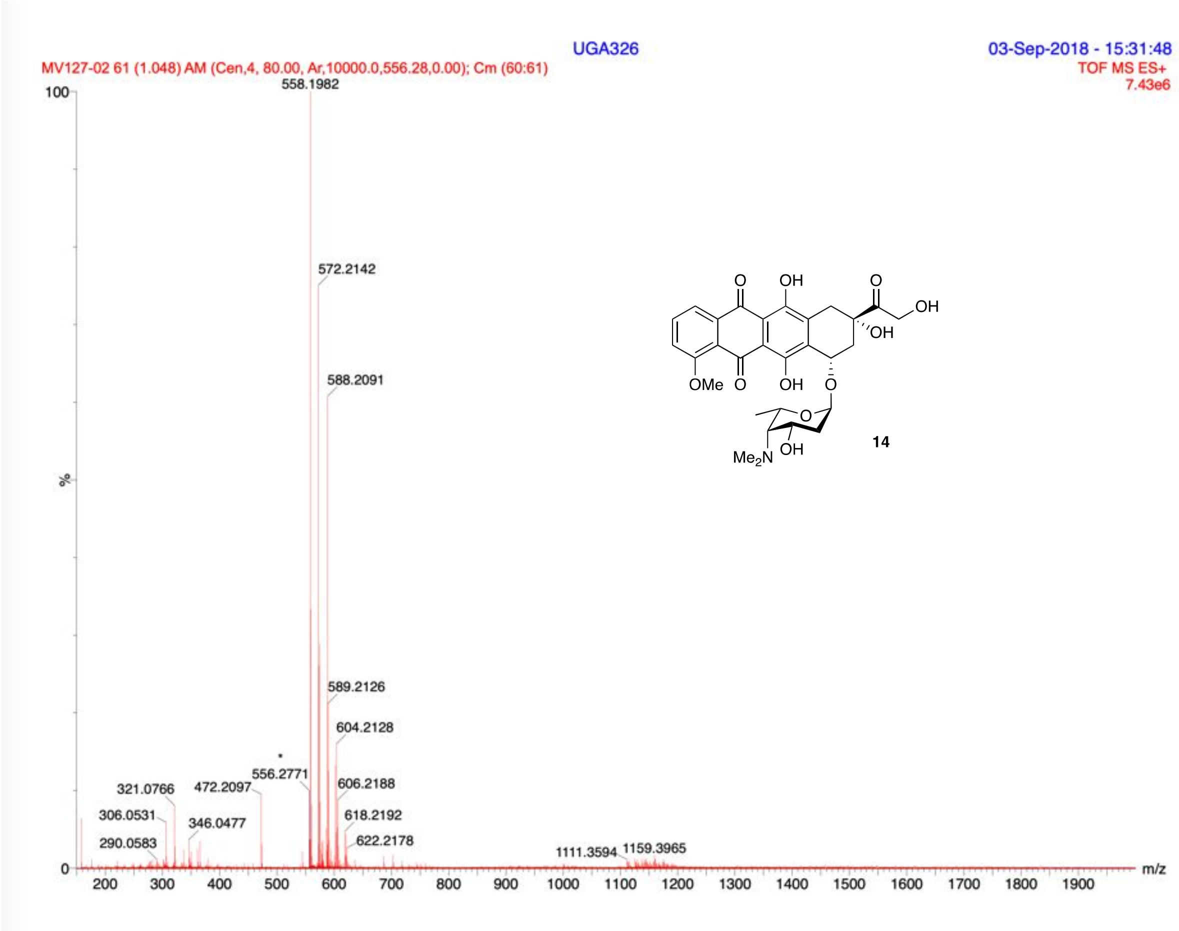
UGA326
03-Sep-2018 - 15:31:48
MV127-02 61 (1.048) AM (Cen,4, 80.00, Ar,10000.0,556.28,0.00); Cm (60:61)
TOF MS ES+
7.43e6
558.1982
572.2142
588.2091
589.2126
604.2128
606.2188
618.2192
622.2178
556.2771
472.2097
321.0766
306.0531
346.0477
290.0583
1111.3594
1159.3965
%
m/z
14

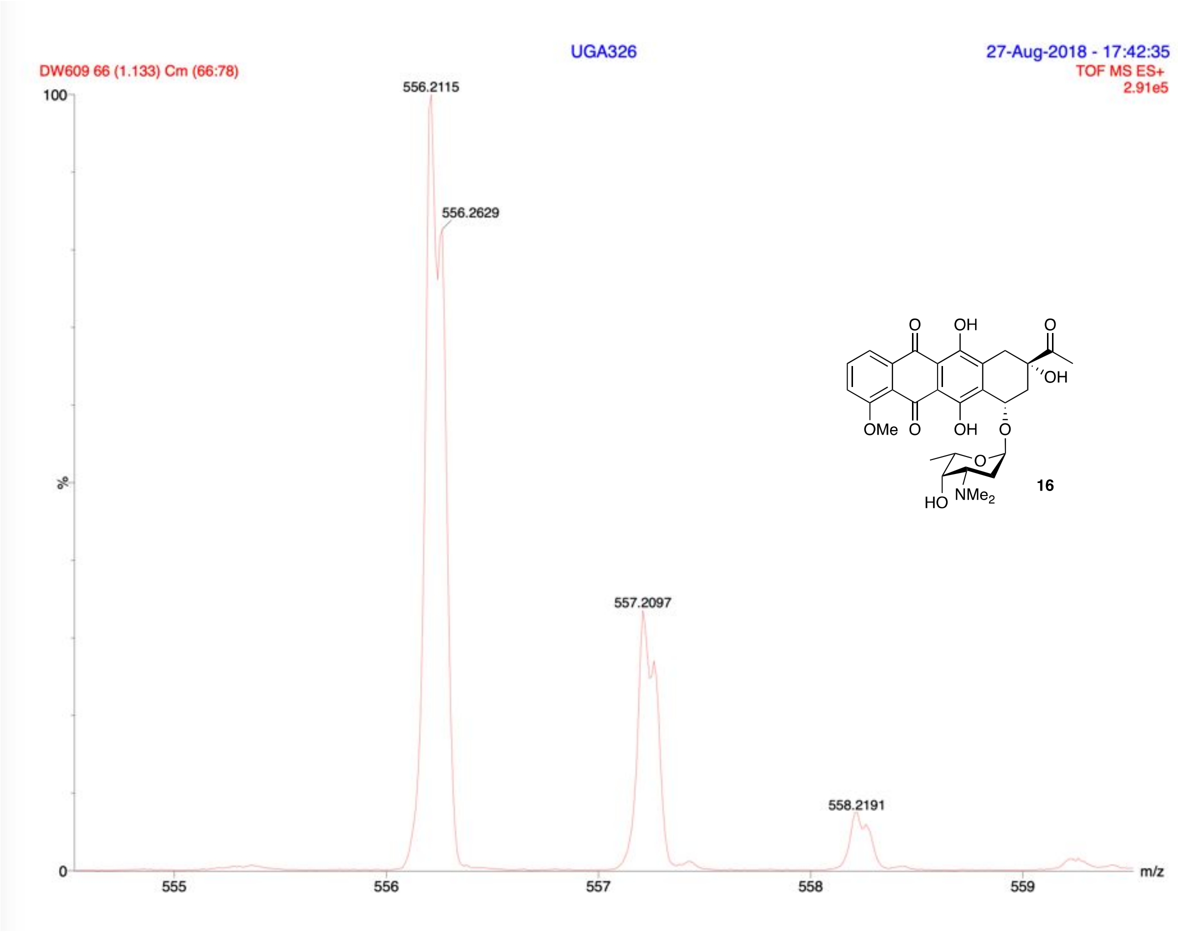
UGA326
27-Aug-2018 - 17:42:35
DW609 66 (1.133) Cm (66:78)
TOF MS ES+
2.91e5
100
%
0
556.2115
556.2629
557.2097
558.2191
555
556
557
558
559
m/z
OMe
OH
HO
NMe2
16

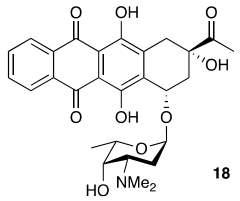
O
OH
O
OH
O
OH
O
O
HO
NMe2

18

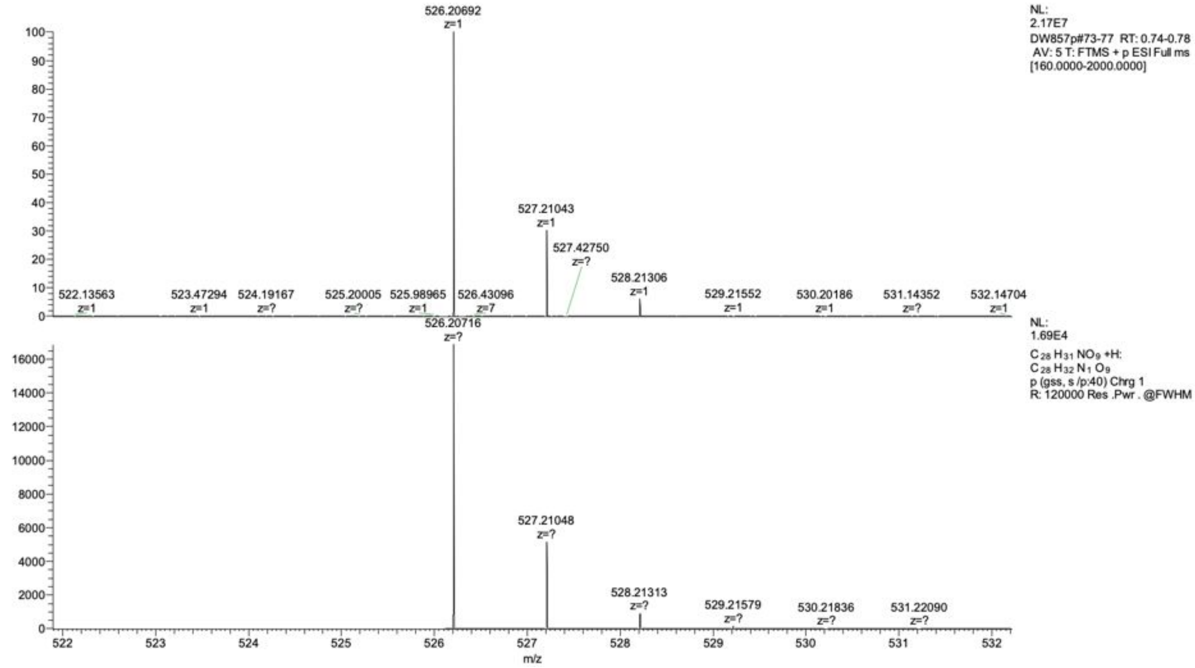
526.20692
z=1
NL:
2.17E7
DW857p#73-77 RT: 0.74-0.78
AV: 5 T: FTMS + p ESI Full ms
[160.0000-2000.0000]
527.21043
z=1
527.42750
z=?
528.21306
z=1
522.13563
z=1
523.47294
z=1
524.19167
z=?
525.20005
z=?
525.98965
z=1
526.43096
z=7
529.21552
z=1
530.20186
z=1
531.14352
z=?
532.14704
z=1
526.20716
z=?
NL:
1.69E4
C 28 H 31 NO 9 +H:
C 28 H 32 N 1 O 9
p (gss, s /p:40) Chrg 1
R: 120000 Res .Pwr . @FWHM
527.21048
z=?
528.21313
z=?
529.21579
z=?
530.21836
z=?
531.22090
z=?
m/z

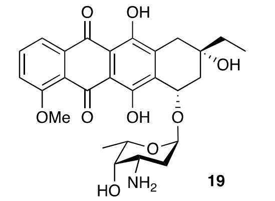
OH
OH
OMe
O
O
OH
O
O
HO
NH2

19

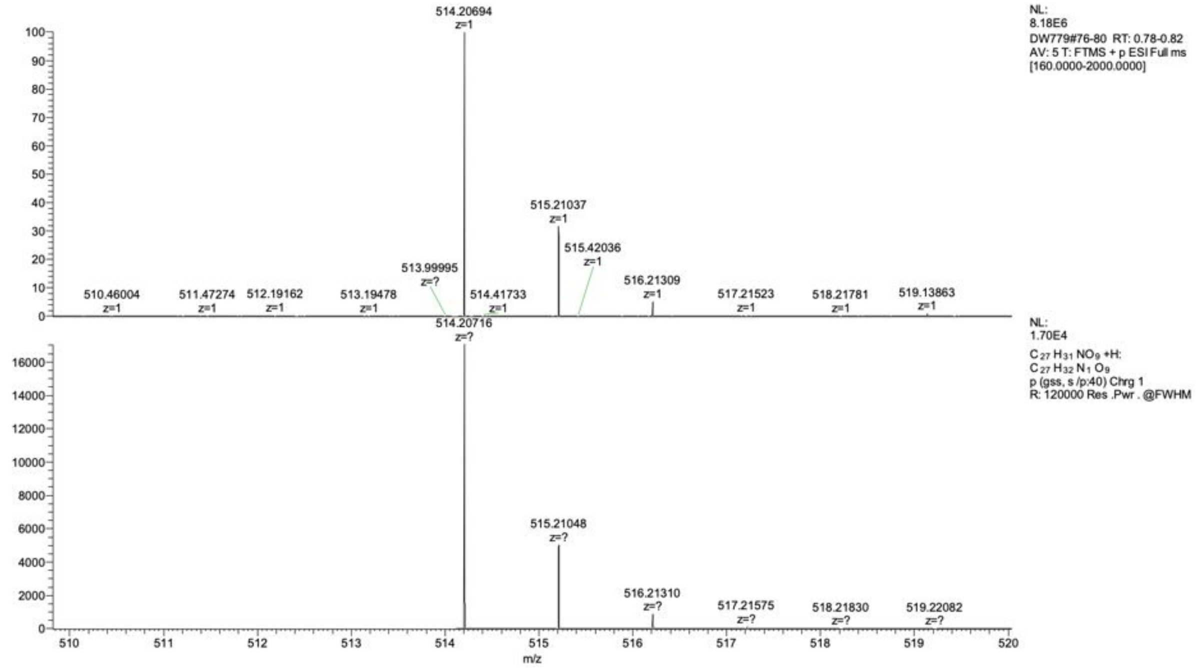
514.20694
z=1
515.21037
z=1
515.42036
z=1
513.99995
z=?
510.46004
z=1
511.47274
z=1
512.19162
z=1
513.19478
z=1
514.41733
z=1
516.21309
z=1
517.21523
z=1
518.21781
z=1
519.13863
z=1
NL:
8.18E6
DW779#76-80 RT: 0.78-0.82
AV: 5 T: FTMS + p ESI Full ms
[160.0000-2000.0000]
514.20716
z=?
NL:
1.70E4
C 27 H 31 NO 9 +H:
C 27 H 32 N 1 O 9
p (gss, s /p:40) Chrg 1
R: 120000 Res .Pwr . @FWHM
515.21048
z=?
516.21310
z=?
517.21575
z=?
518.21830
z=?
519.22082
z=?
m/z

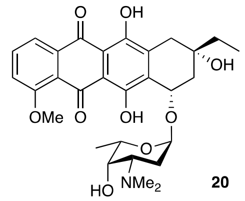

20

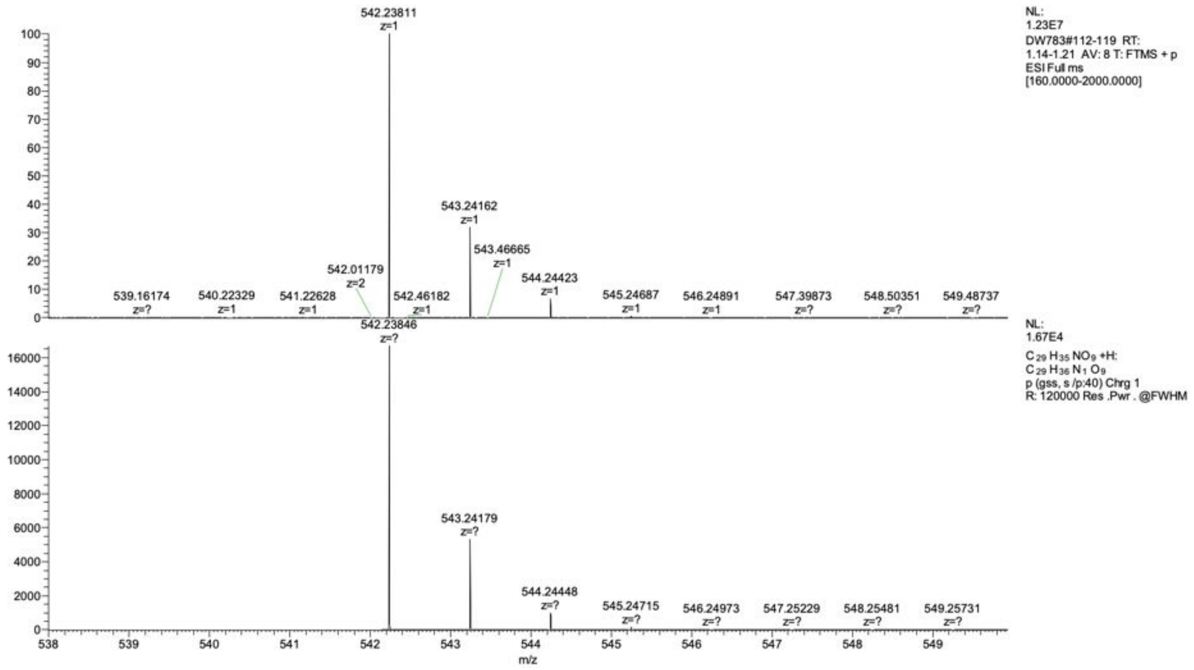

NL: 1.23E7
DW783#112-119 RT: 1.14-1.21 AV: 8 T: FTMS + p ESI Full ms [160.0000-2000.0000]
NL: 1.67E4
C 29 H 35 NO 9 +H: C 29 H 36 N 1 O 9
p (gss, s /p:40) Chrg 1
R: 120000 Res .Pwr . @FWHM
542.23811 z=1
543.24162 z=1
543.46665 z=1
542.01179 z=2
544.24423 z=1
539.16174 z=?
540.22329 z=1
541.22628 z=1
542.46182 z=1
545.24687 z=1
546.24891 z=1
547.39873 z=?
548.50351 z=?
549.48737 z=?
542.23846 z=?
543.24179 z=?
544.24448 z=?
545.24715 z=?
546.24973 z=?
547.25229 z=?
548.25481 z=?
549.25731 z=?
m/z

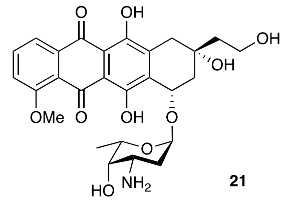

21

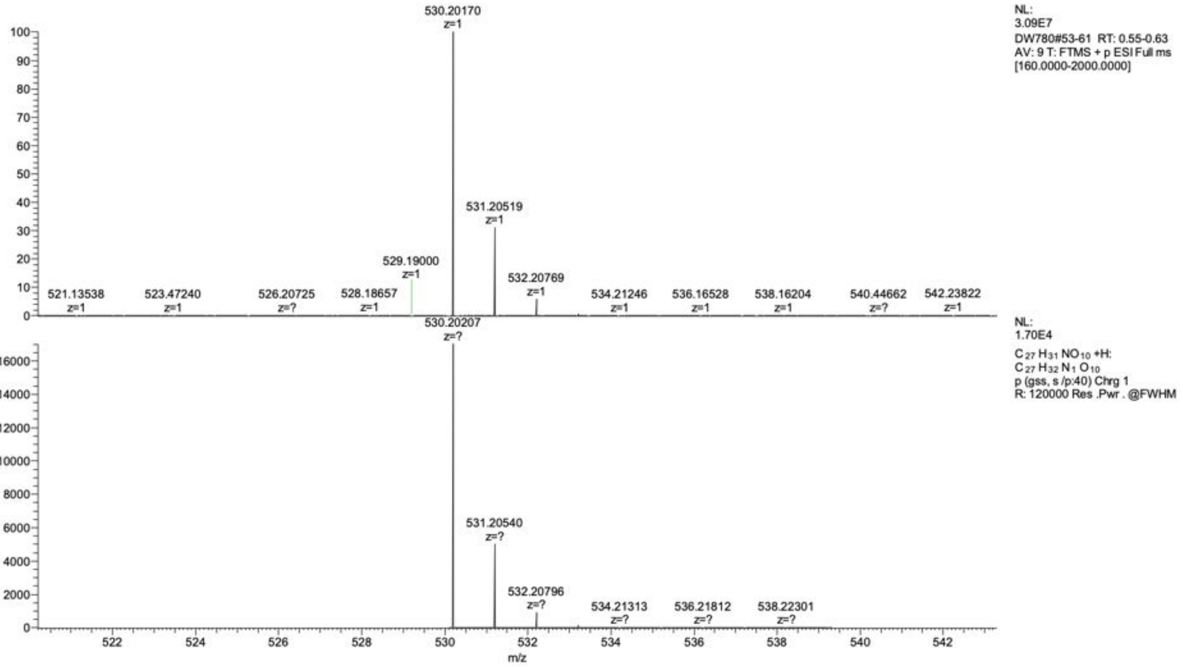

530.20170
z=1
531.20519
z=1
529.19000
z=1
532.20769
z=1
521.13538
z=1
523.47240
z=1
526.20725
z=?
528.18657
z=1
534.21246
z=1
536.16528
z=1
538.16204
z=1
540.44662
z=?
542.23822
z=1
NL:
3.09E7
DW780#53-61 RT: 0.55-0.63
AV: 9 T: FTMS + p ESI Full ms
[160.0000-2000.0000]
530.20207
z=?
531.20540
z=?
532.20796
z=?
534.21313
z=?
536.21812
z=?
538.22301
z=?
NL:
1.70E4
C 27 H 31 NO 10 +H:
C 27 H 32 N 1 O 10
p (gss, s /p:40) Chrg 1
R: 120000 Res .Pwr . @FWHM
m/z

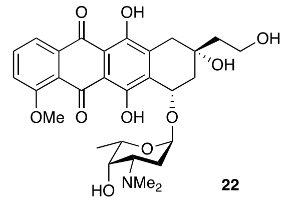

22

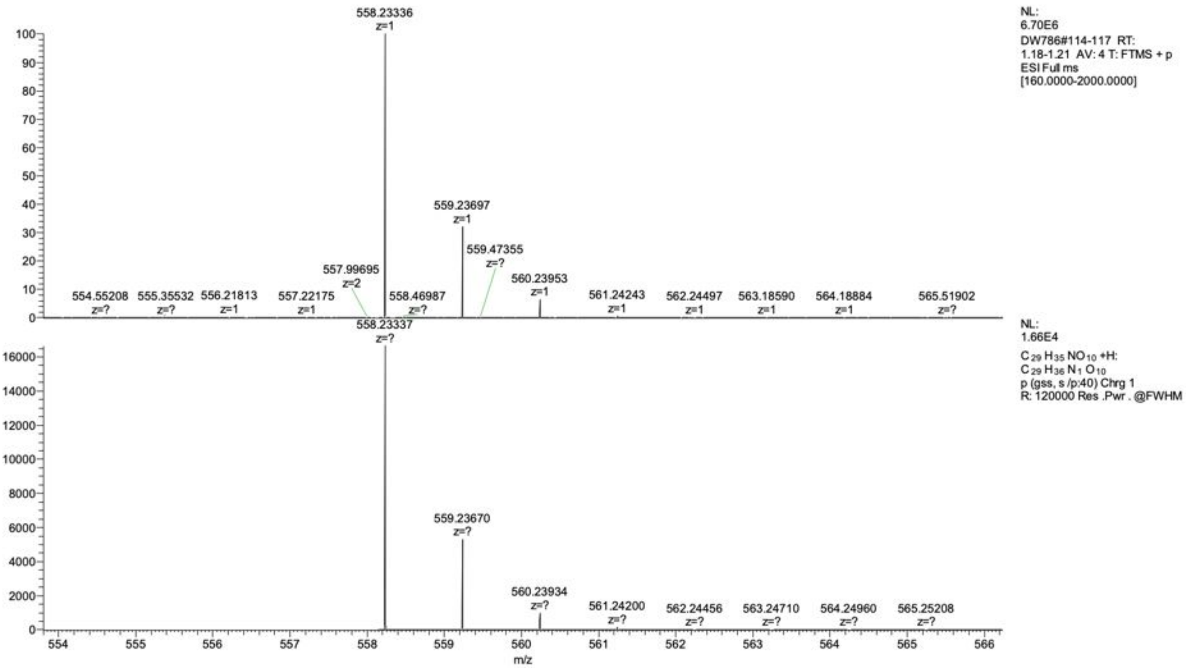

NL:
6.70E6
DW786#114-117 RT:
1.18-1.21 AV: 4 T: FTMS + p
ESI Full ms
[160.0000-2000.0000]
558.23336
z=1
559.23697
z=1
559.47355
z=?
557.99695
z=2
560.23953
z=1
554.55208
555.35532
556.21813
557.22175
558.46987
561.24243
562.24497
563.18590
564.18884
565.51902
NL:
1.66E4
C 29 H 35 NO 10 +H:
C 29 H 36 N 1 O 10
p (gss, s /p:40) Chrg 1
R: 120000 Res .Pwr . @FWHM
558.23337
z=?
559.23670
z=?
560.23934
z=?
561.24200
562.24456
563.24710
564.24960
565.25208
m/z

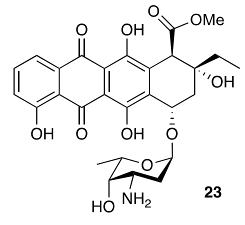

O
OMe
O
OH
OH
OH
O
OH
O
O
HO
NH2

23

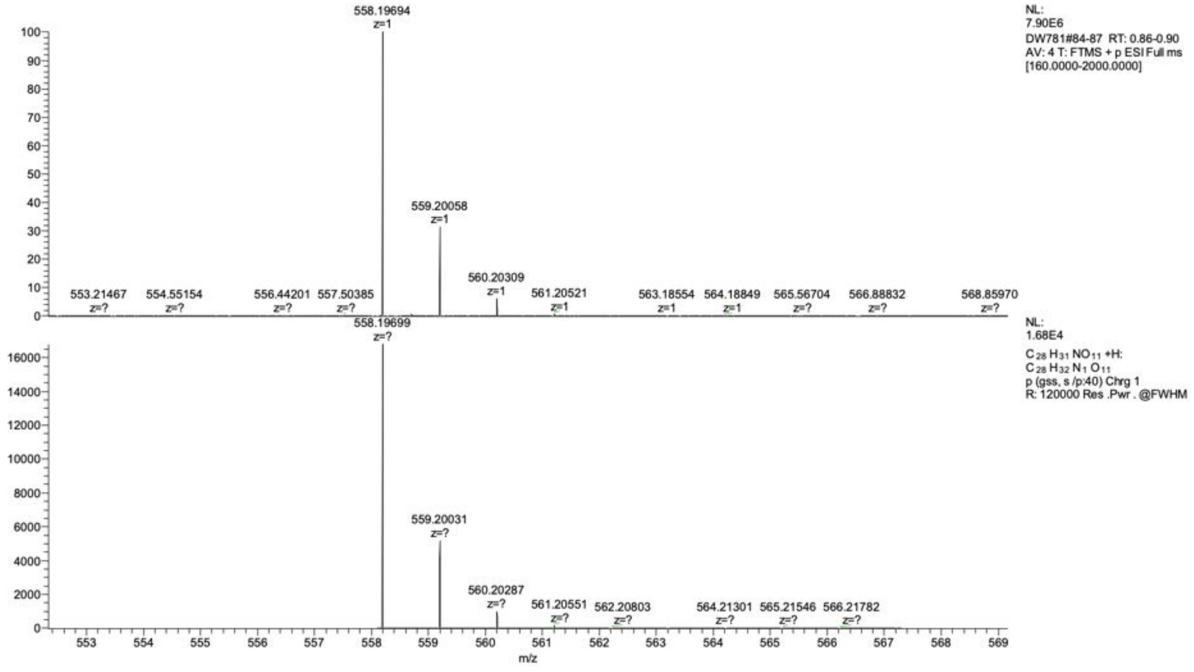

558.19694
z=1
559.20058
z=1
560.20309
z=1
553.21467
z=?
554.55154
z=?
556.44201
z=?
557.50385
z=?
561.20521
z=1
563.18554
z=1
564.18849
z=1
565.56704
z=?
566.88832
z=?
568.85970
z=?
NL:
7.90E6
DW781#84-87 RT: 0.86-0.90
AV: 4 T: FTMS + p ESI Full ms
[160.0000-2000.0000]
558.19699
z=?
559.20031
z=?
560.20287
z=?
561.20551
z=?
562.20803
z=?
564.21301
z=?
565.21546
z=?
566.21782
z=?
NL:
1.68E4
C 28 H 31 NO 11 +H:
C 28 H 32 N 1 O 11
p (gss, s /p:40) Chrg 1
R: 120000 Res .Pwr . @FWHM
m/z

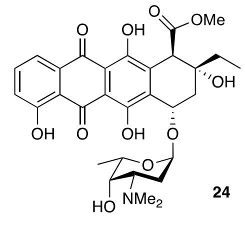
O
OH
OMe
OH
OH
O
OH
O
O
HO
NMe2

24

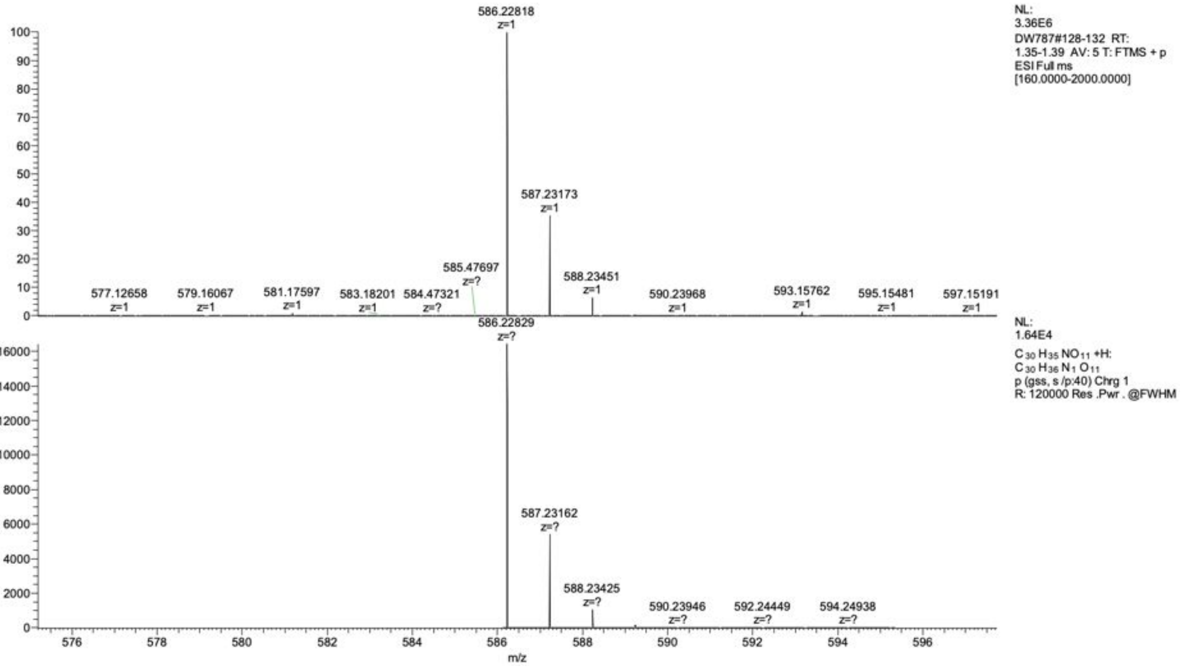
586.22818
z=1
NL:
3.36E6
DW787#128-132 RT:
1.35-1.39 AV: 5 T: FTMS + p
ESI Full ms
[160.0000-2000.0000]
587.23173
z=1
585.47697
z=?
588.23451
z=1
577.12658
z=1
579.16067
z=1
581.17597
z=1
583.18201
z=1
584.47321
z=?
590.23968
z=1
593.15762
z=1
595.15481
z=1
597.15191
z=1
586.22829
z=?
NL:
1.64E4
C 30 H 35 NO 11 +H:
C 30 H 36 N 1 O 11
p (gss, s /p:40) Chrg 1
R: 120000 Res .Pwr . @FWHM
587.23162
z=?
588.23425
z=?
590.23946
z=?
592.24449
z=?
594.24938
z=?
m/z

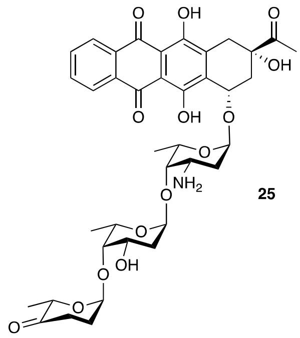
O
OH
O
OH
OH
O
OH
O
O
NH2
O
O
OH
O
O
O
O
25

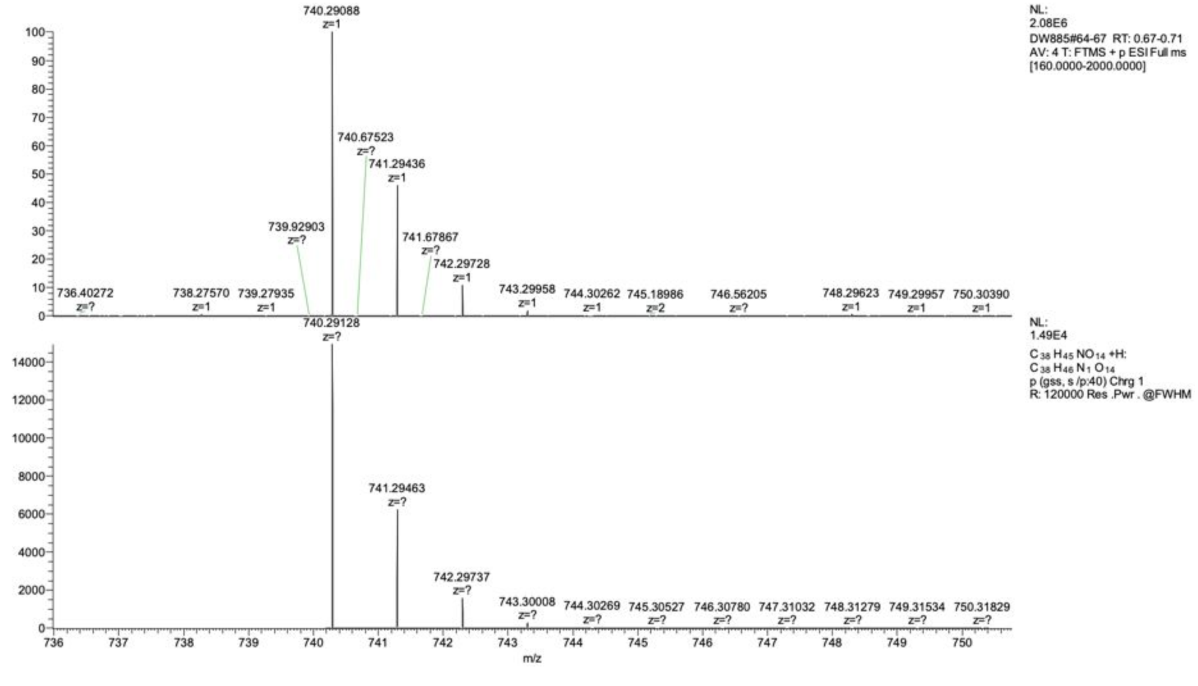
NL:
2.08E6
DW885#64-67 RT: 0.67-0.71
AV: 4 T: FTMS + p ESI Full ms
[160.0000-2000.0000]
NL:
1.49E4
C 38 H 45 NO 14 +H:
C 38 H 46 N 1 O 14
p (gss, s /p:40) Chrg 1
R: 120000 Res .Pwr . @FWHM
740.29088
z=1
740.67523
z=?
741.29436
z=1
739.92903
z=?
741.67867
z=?
742.29728
z=1
736.40272
z=?
738.27570
z=1
739.27935
z=1
743.29958
z=1
744.30262
z=1
745.18986
z=2
746.56205
z=?
748.29623
z=1
749.29957
z=1
750.30390
z=1
740.29128
z=?
741.29463
z=?
742.29737
z=?
743.30008
z=?
744.30269
z=?
745.30527
z=?
746.30780
z=?
747.31032
z=?
748.31279
z=?
749.31534
z=?
750.31829
z=?
m/z

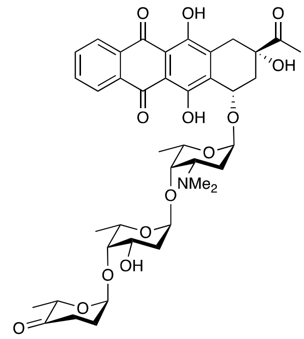
OH
O
OH
OH
O
O
NMe2
O
OH
O
O

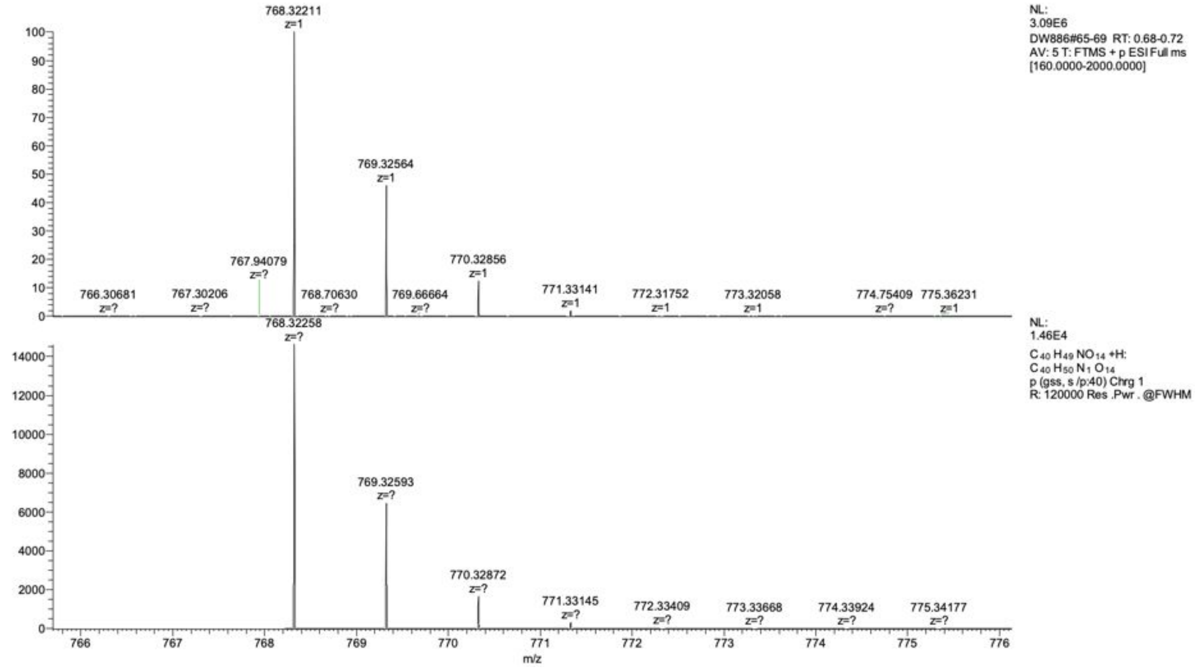
NL:
3.09E6
DW886#65-69 RT: 0.68-0.72
AV: 5 T: FTMS + p ESI Full ms
[160.0000-2000.0000]
768.32211
z=1
769.32564
z=1
767.94079
z=?
770.32856
z=1
766.30681
z=?
767.30206
z=?
768.70630
z=?
769.66664
z=?
771.33141
z=1
772.31752
z=1
773.32058
z=1
774.75409
z=?
775.36231
z=1
NL:
1.46E4
C 40 H 49 NO 14 +H:
C 40 H 50 N 1 O 14
p (gss, s /p:40) Chrg 1
R: 120000 Res .Pwr . @FWHM
768.32258
z=?
769.32593
z=?
770.32872
z=?
771.33145
z=?
772.33409
z=?
773.33668
z=?
774.33924
z=?
775.34177
z=?
m/z

F: HPLC traces

Compound 3

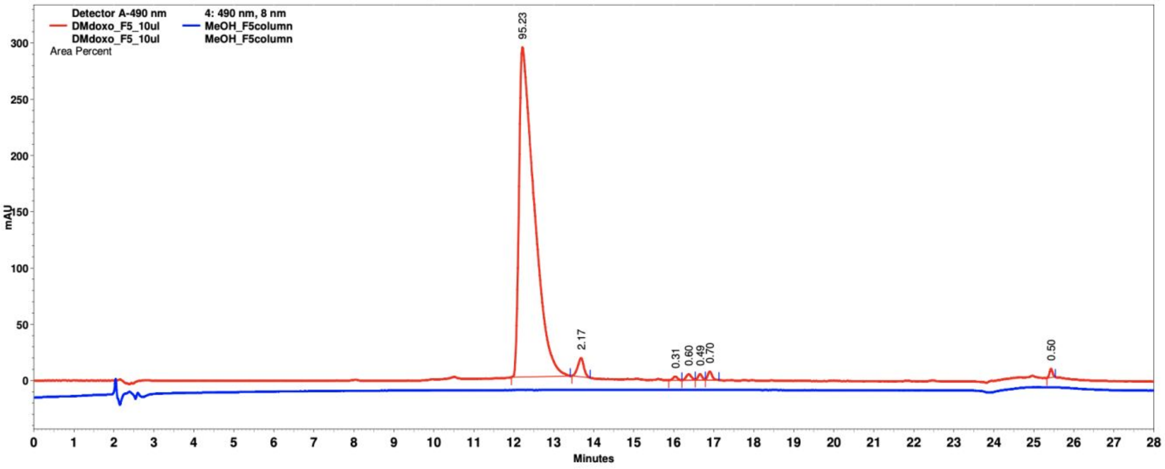

<u>Column description:</u>

Phenomenex Kinetex® F5 2.6µm 100 Å, LC Column 100 x 4.6 mm (cat no. 00D-4723-E0)

Blank run (MeOH) in blue

Flow rate: 0.5 mL/min; with following gradient elution:

time (min)	B%
0.1	15
19	40
20	100
24	100
24.1	15
28	15

<u>Buffer used for elution:</u>

Buffer A:

45 % ammonium acetate solution

5% ACN

50% MQ

Buffer B:

100% ACN

Compound 16

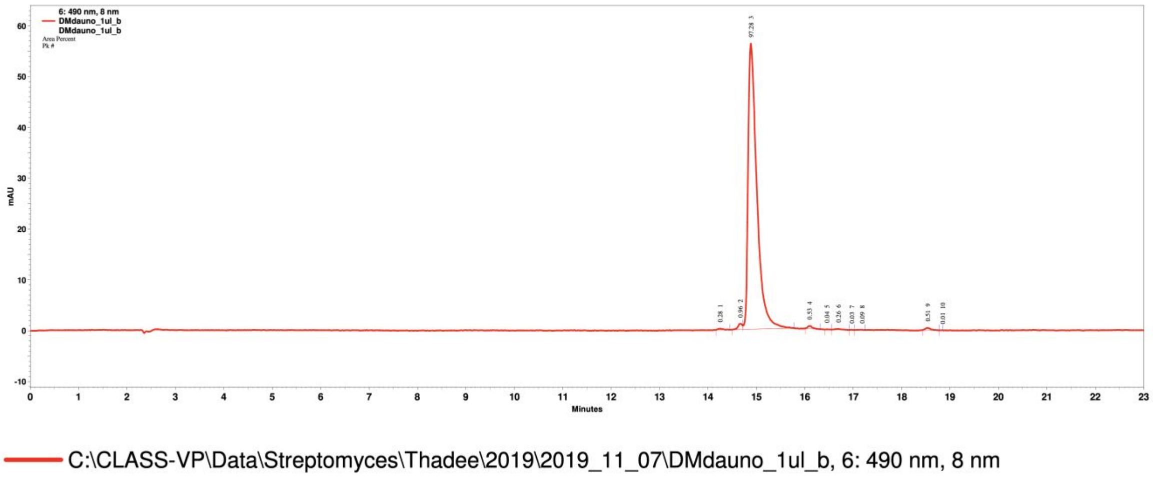

<u>Column description:</u>

Phenomenex Kinetex® F5 2.6μm 100 Å, LC Column 100 x 4.6 mm (cat no. 00D-4723-E0)

Flow rate: 0.5 mL/min; with following gradient elution:

time (min)	B%
0.1	15
19	40
20	100
24	100
24.1	15
28	15

<u>Buffer used for elution:</u>

Buffer A:

45 % ammonium acetate solution

5% ACN

50% MQ

Buffer B:

100% ACN

G: References

(1) Nielsen, D.; Maare, C.; Skovsgaard, T. Cellular Resistance to Anthracyclines. *General Pharmacology: The Vascular System* **1996**, *27* (2), 251–255. https://doi.org/10.1016/0306-3623(95)02013-6.

(2) Rayner, D. M.; Cutts, S. M. Anthracyclines. *Side Effects of Drugs Annual* **2014**, *36*, 683–694. https://doi.org/10.1016/B978-0-444-63407-8.00045-9.

(3) Wander, D. P. A.; van der Zanden, S. Y.; van der Marel, G. A.; Overkleeft, H. S.; Neefjes, J.; Codée, J. D. C. Doxorubicin and Aclarubicin: Shuffling Anthracycline Glycans for Improved Anticancer Agents. *J Med Chem* **2020**, *63* (21), 12814–12829. https://doi.org/10.1021/acs.jmedchem.0c01191.

(4) Kloc, K.; Mlochowski, J.; Syper, L. SYNTHESIS OF NOVEL QUINONES WITH SILVER (II) DIPICOLINATE AS A NEW SELECTIVE OXIDANT. *Chem Lett* **1980**, *9* (6), 725–728. https://doi.org/10.1246/cl.1980.725.

(5) Goddard-Borger, E. D.; Stick, R. V. An Efficient, Inexpensive, and Shelf-Stable Diazotransfer Reagent: Imidazole-1-Sulfonyl Azide Hydrochloride. *Org Lett* **2007**, *9* (19), 3797–3800. https://doi.org/10.1021/ol701581g.

(6) Wang, T.; Ng, D. Y. W.; Wu, Y.; Thomas, J.; TamTran, T.; Weil, T. Bis-Sulfide Bioconjugates for Glutathione Triggered Tumor Responsive Drug Release. *Chem. Commun.* **2014**, *50* (9), 1116–1118. https://doi.org/10.1039/C3CC47003B.

(7) Zhou, J.; Yang, L.; Hu, W. Stereoselective Synthesis of a Sulfated Tetrasaccharide Corresponding to a Rare Sequence in the Galactofucan Isolated from *Sargassum Polycystum*. *J Org Chem* **2014**, *79* (10), 4718–4726. https://doi.org/10.1021/jo500503r.

(8) HORTON, D.; PRIEBE, W.; VARELA, O. Synthesis and Antitumor Activity of 3’-Deamino-3’-Hydroxydoxorubicin. A Facile Procedure for the Preparation of Doxorubicin Analogs. *J Antibiot (Tokyo)* **1984**, *37* (8), 853–858. https://doi.org/10.7164/antibiotics.37.853.

(9) Noshita, T.; Sugiyama, T.; Kitazumi, Y.; Oritani, T. Reinvestigation of Phenolic Ferrier Reaction: Selective Synthesis of Aryl O-Δ 2 -Glycosides. *Biosci Biotechnol Biochem* **1995**, *59* (11), 2052–2055. https://doi.org/10.1271/bbb.59.2052.

(10) Noshita, T.; Sugiyama, T.; Kitazumi, Y.; Oritani, T. Phenolic Ferrier Reaction and Its Application to the Natural Product Synthesis. *Tetrahedron Lett* **1994**, *35* (44), 8259–8262. https://doi.org/10.1016/0040-4039(94)88297-5.

(11) Oberthür, M.; Leimkuhler, C.; Kruger, R. G.; Lu, W.; Walsh, C. T.; Kahne, D. A Systematic Investigation of the Synthetic Utility of Glycopeptide Glycosyltransferases. *J Am Chem Soc* **2005**, *127* (30), 10747–10752. https://doi.org/10.1021/ja052945s.

(12) Gui, C.; Mo, X.; Gu, Y.-C.; Ju, J. Elucidating the Sugar Tailoring Steps in the Cytorhodin Biosynthetic Pathway. *Org Lett* **2017**, *19* (20), 5617–5620. https://doi.org/10.1021/acs.orglett.7b02758.

(13) Kolar, C.; Kneissl, G.; Knödler, U.; Dehmel, K. Semisynthetic ε-(Iso)Rhodomycins: A New Glycosylation Variant and Modification Reactions. *Carbohydr Res* **1991**, *209* (C), 89–100. https://doi.org/10.1016/0008-6215(91)80147-F.

(14) Xuereb, H.; Maletic, M.; Gildersleeve, J.; Pelczer, I.; Kahne, D. Design of an Oligosaccharide Scaffold That Binds in the Minor Groove of DNA. *J Am Chem Soc* **2000**, *122* (9), 1883–1890. https://doi.org/10.1021/ja992513f.
